# Supplementary material for: Stream nitrate enrichment and increased light yet no algal response following forest harvest and experimental manipulation of headwater riparian zones
Source: PLoS One. 2023 Apr 20;18(4):e0284590. doi: 10.1371/journal.pone.0284590 (PMC10118188; doi:10.1371/journal.pone.0284590)
Supplement: S1 Table — Box-Cox transformations were used with the goal of improving model quality (See S1 File for details on Box-Cox transformations). An exponent of 1 indicates that raw data were used. For normality tests, bold indicates that the null hypothesis of normal residuals was rejected. For Levene’s test, bold indicates that the null hypothesis of a common variance across groups of a given factor was rejected. Significance was not reported in Table 2 when R2CV was negative for PLS. For Wu Jackknife and Wild Bootstrap, bold indicates regression coefficients were significantly different than zero. We did not assume the regressor rows were independent and consequently used the Wild Bootstrap and Wu Jackknife for discerning model parameter significance. Interaction predictions are PLS model predictions transformed back to the original scale of the data. Dissolved organic carbon (DOC), Soluble reactive phosphorus (SRP), Dissolved organic nitrogen (DON), Dissolved inorganic nitrogen (DIN), Chlorophyll a (Chl a). (DOCX) [file pone.0284590.s001.docx]

| **S1 Table. Details of PLS, Wu jacknife, Wild Bootstrap models for headwater treatment groups.** | | | | | | | | | | |
| --- | --- | --- | --- | --- | --- | --- | --- | --- | --- | --- |
|  |  | **Canopy closure** | **DOC** | **SRP** | **DON** | **DIN** | **DIN:SRP Molar Ratio** | **Epilithon**  **AFDM** | **Chl a** | **Surface sediment** |
| **Box_Cox Transformations** | Box-Cox Exponent | 1.00 | -15.00 | -1.25 | -5.00 | -10.00 | 0.09 | -2.94 | -2.62 | -0.01 |
|  | Box-Cox Offset | 1.00 | 5.70 | 0.00 | 0.26 | 2.34 | -0.58 | 9.16 | 8.25 | -0.12 |
| **Normality tests** | Anderson-Darling | 0.00 | 0.18 | 0.32 | 0.27 | 0.26 | 0.45 | **0.04*** | 0.13 | 0.86 |
|  | Jarque-Bera | 0.00 | 0.41 | 0.46 | 0.15 | 0.40 | 0.39 | 0.06 | 0.08 | 0.41 |
|  | Lillifors | 0.00 | 0.06 | 0.36 | 0.50 | 0.30 | 0.32 | 0.07 | 0.33 | 0.50 |
|  | Shapiro-Wilk | 0.00 | 0.83 | 0.46 | 0.26 | 0.27 | 0.58 | **0.02*** | 0.09 | 0.77 |
| **Levene Test (grouping factors)** | treatment | 0.02 | 0.14 | **0.00*** | 0.06 | **0.01*** | **0.00*** | 0.61 | **0.05*** | **0.02*** |
|  | period | 0.00 | 0.63 | 0.93 | 0.32 | **0.00*** | 0.36 | 0.16 | 0.41 | 0.12 |
|  | interaction | 0.00 | 0.14 | **0.00*** | **0.04*** | **0.00*** | **0.00*** | 0.58 | 0.11 | 0.08 |
| **PLS stats** | R^2^ | 0.48 | 0.06 | 0.59 | 0.13 | 0.37 | 0.28 | 0.10 | 0.16 | 0.04 |
|  | R^2^CV | 0.38 | -0.02 | 0.52 | 0.03 | 0.29 | 0.18 | 0.00 | 0.05 | -0.03 |
|  | Optimal number of latent variables | 3 | 1 | 3 | 1 | 3 | 2 | 1 | 1 | 1 |
| **Wu Jackknife p-values for PLS models** | Clearcut Variable | **0.03*** | 0.46 | **0.00*** | 0.18 | **0.01*** | **0.02*** | 0.35 | 0.35 | 0.34 |
|  | Clearcut Variable *Post | **0.00*** | 0.22 | 0.22 | 0.29 | **0.00*** | **0.00*** | 0.86 | 0.34 | 0.45 |
|  | Clearcut Uniform | 0.38 | 0.14 | **0.00*** | **0.02*** | 0.64 | **0.02*** | 0.24 | 0.59 | 0.34 |
|  | Clearcut Uniform *Post | 0.14 | 0.56 | 0.61 | **0.02*** | **0.00*** | 0.09 | **0.05*** | 0.34 | 0.96 |
|  | Reference | 0.35 | **0.04*** | **0.00*** | 0.33 | 0.10 | 0.27 | 0.67 | 0.24 | 0.09 |
|  | Post | **0.01*** | 0.64 | 0.18 | 0.06 | **0.00*** | **0.00*** | **0.01*** | **0.01*** | 0.30 |
|  | Reference * Post | **0.02*** | 0.09 | 0.99 | 0.54 | 0.17 | 0.70 | 0.15 | **0.00*** | **0.04*** |
| **Wild Bootstrap** | Clearcut Variable | **1** | 0 | **1** | 0 | **1** | **1** | 0 | 0 | 0 |
|  | Clearcut Variable *Post | **1** | 0 | 0 | 0 | **1** | **1** | 0 | 0 | 0 |
|  | Clearcut Uniform | 0 | 0 | **1** | 1 | 0 | **1** | 0 | 0 | 0 |
|  | Clearcut Uniform *Post | 0 | 0 | 0 | **1** | **1** | **1** | **1** | 0 | 0 |
|  | Reference | 0 | 0 | **1** | 0 | 0 | 0 | 0 | 0 | 0 |
|  | Post | **1** | 0 | 0 | **1** | **1** | **1** | **1** | **1** | 0 |
|  | Reference * Post | **1** | 0 | 0 | 0 | 0 | 0 | 0 | **1** | **1** |
| **Interaction Predictions** | Clearcut Variable *Pre | 92.28 | 0.53 | 0.00 | 0.03 | 0.04 | 25.08 | 7.54 | 6.56 | 51.25 |
|  | Clearcut Variable *Post | 54.44 | 0.55 | 0.00 | 0.03 | 0.18 | 110.69 | 6.66 | 5.24 | 49.75 |
|  | Clearcut Uniform *Pre | 94.36 | 0.55 | 0.01 | 0.02 | 0.07 | 9.93 | 6.77 | 7.25 | 52.67 |
|  | Clearcut Uniform *Post | 91.67 | 0.55 | 0.02 | 0.01 | 0.27 | 31.34 | 5.24 | 5.67 | 58.52 |
|  | Thinned *Pre | 95.59 | 0.52 | 0.00 | 0.03 | 0.06 | 16.89 | 7.17 | 6.99 | 58.08 |
|  | Thinned *Post | 89.86 | 0.51 | 0.00 | 0.03 | 0.12 | 33.04 | 6.26 | 5.99 | 64.95 |
|  | Reference *Pre | 94.29 | 0.49 | 0.01 | 0.03 | 0.09 | 13.80 | 7.32 | 6.53 | 68.24 |
|  | Reference *Post | 93.74 | 0.45 | 0.01 | 0.03 | 0.11 | 24.72 | 5.81 | 4.70 | 98.88 |
