# Supplementary material for: Stream nitrate enrichment and increased light yet no algal response following forest harvest and experimental manipulation of headwater riparian zones
Source: PLoS One. 2023 Apr 20;18(4):e0284590. doi: 10.1371/journal.pone.0284590 (PMC10118188; doi:10.1371/journal.pone.0284590)
Supplement: S3 Table — A) Kolmogorov-Smirnov two sample test p-values compare post- and pre- empirical cumulative distribution functions for downstream site groups (downstream of harvest and downstream of reference). B) Comparison of 95% Bias Corrected accelerated (BCa) Bootstrap confidence intervals (CI) for observed pre- and post-harvest treatment means for each response variable. "0" indicates the pre- and post-harvest CI overlapped; "Increase" indicates that the lower bound of the post-harvest CI was greater than the upper bound of the pre-harvest CI. "decrease" indicated where pre-harvest was greater than post-harvest. C) Pre/Post comparisons of significance for PLS model predictions for each attribute, based on 95% Wild Bootstrap confidence intervals. When R2CV was negative, significance was not reported, indicated by “- -“. Within a group, pre/post with the same letters are not significantly different: “A” denotes the largest value, “B” is second largest, etc, for that attribute. Box-Cox transformations were used with the goal of improving model quality. For Wu Jackknife and Wild Bootstrap, bold indicates regression coefficients were significantly different than zero. We did not assume the regressor rows were independent and consequently used the Wild Bootstrap and Wu Jackknife for discerning model parameter significance. Dissolved organic carbon (DOC), Soluble reactive phosphorus (SRP), Dissolved organic nitrogen (DON), Dissolved inorganic nitrogen (DIN), Chlorophyll a (Chl a). (DOCX) [file pone.0284590.s003.docx]

| **S3 Table. Statistical summaries and details of PLS, Wu jackknife and Wild Bootstrap models for downstream sites.** | | | | | | | | | |
| --- | --- | --- | --- | --- | --- | --- | --- | --- | --- |
| **Analysis** |  | **DOC** | **SRP** | **DON** | **DIN** | **DIN:SRP Molar Ratio** | **Epilithon**  **AFDM** | **Chl a** | **Surface Sediment** |
| **A. Kolmogorov-Smirnov two sample test p-values** | Downstream of harvested | 0.43 | 0.07 | 0.79 | 0.07 | 0.43 | **0.01*** | **0.04*** | 0.26 |
|  | Downstream-reference | 0.11 | 0.53 | 0.11 | **0.01*** | **0.01*** | 0.26 | 0.26 | 0.96 |
| **B. Comparison of 95% confidence intervals** | Downstream of harvested | 0 | 0 | 0 | **increase** | 0 | **decrease** | 0 | 0 |
|  | Downstream-reference | 0 | 0 | 0 | **decrease** | **decrease** | 0 | 0 | 0 |
| **C. Pre-Post comparison of PLS model predictions** | Downstream of harvested | -- | B/AB | -- | B/AB | -- | AB /A | AB /B | -- |
|  | Downstream-reference | -- | AB/A | -- | **A/B*** | -- | A/AB | A/A | -- |
| **Box-Cox Transformation** | Box-Cox exponent | -10.0 | -6.81 | 1.00 | -3.93 | 1.22 | 1.00 | 1.00 | -0.49 |
|  | Box-Cox offset | 0.44 | 0.012 | 0.000 | 0.26 | 10.0 | 0.000 | 0.000 | 21.2 |
| **PLS Stats** | R^2^ | 0.18 | 0.25 | 0.07 | 0.37 | 0.12 | 0.26 | 0.25 | 0.06 |
|  | R^2^CV | -0.11 | 0.11 | -0.10 | 0.23 | -0.06 | 0.15 | 0.16 | -0.09 |
|  | Optimal number of latent variables | 2 | 2 | 1 | 3 | 3 | 1 | 1 | 1 |
| **Wu Jackknife p-values** | Downstream of harvested | -- | **0.01*** | -- | **0.00*** | -- | 0.28 | **0.02** | -- |
|  | Post-harvest | -- | **0.01*** | -- | **0.00*** | -- | **0.00*** | **0.01*** | -- |
|  | Downstream of harvested * Post-harvest | -- | 0.13 | -- | **0.00*** | -- | **0.00*** | **0.00*** | -- |
| **Wild bootstrap** | Downstream of harvested | -- | **1** | -- | **1** | -- | 0 | **1** | -- |
|  | Post-harvest | -- | **1** | -- | **1** | -- | **1** | **1** | -- |
|  | Downstream of harvested * Post-harvest | -- | 0 | -- | **1** | -- | **1** | **1** | -- |
