# Supplementary material for: Stream nitrate enrichment and increased light yet no algal response following forest harvest and experimental manipulation of headwater riparian zones
Source: PLoS One. 2023 Apr 20;18(4):e0284590. doi: 10.1371/journal.pone.0284590 (PMC10118188; doi:10.1371/journal.pone.0284590)
Supplement: S4 Table — Concentrations of A) stream nitrate (NO3) and B) ammonium (NH4) at each site, each year at the time of early summer sampling. "--" indicates no sample collected. Additional Trask River Watershed study chemistry data are available through US Forest Service Research Data Archive: https://doi.org/10.2737/RDS-2022-0002. (DOCX) [file pone.0284590.s004.docx]

**S4 Table. Concentrations of instream A) nitrate (NO^3^) and B) ammonium (NH_4_) at each site, each year at the time of early summer sampling.**

| **A. NO_3_ Concentrations (mg N/L)** | | | | | | | | | | |
| --- | --- | --- | --- | --- | --- | --- | --- | --- | --- | --- |
| **Catchment** | **Watershed** | **Harvest treatment** | **2008** | **2009** | **2010** | **2011** | **2013** | **2014** | **2015** | **2016** |
| **Upper Main** | UM 1 | Reference | 0.009 | 0.018 | 0.011 | 0.005 | 0.013 | 0.019 | 0.040 | 0.027 |
|  | UM 2 | Clearcut variable buffer | 0.037 | 0.067 | 0.034 | 0.026 | 0.187 | 0.356 | 0.319 | 0.227 |
|  | UM 3 | Clearcut variable buffer | 0.052 | 0.071 | 0.052 | 0.022 | 0.037 | 0.069 | 0.092 | 0.059 |
|  | UM Downstream | Downstream of harvested | 0.156 | 0.188 | 0.154 | 0.113 | 0.107 | 0.140 | 0.189 | 0.131 |
| **Gus** | GS 1 | Reference | 0.162 | 0.343 | 0.364 | 0.253 | 0.295 | 0.336 | 0.441 | 0.436 |
|  | GS 2 | Thinned | 0.052 | 0.094 | 0.064 | 0.048 | 0.067 | 0.098 | 0.134 | 0.149 |
|  | GS 3 | Clearcut variable buffer | 0.006 | 0.017 | 0.004 | 0.006 | 0.030 | 0.250 | 0.369 | 0.688 |
|  | GS Downstream | Downstream of harvested | 0.103 | 0.160 | 0.144 | 0.105 | 0.150 | 0.176 | 0.217 | 0.270 |
| **Pothole** | PH 1 | Clearcut uniform buffer | 0.041 | 0.000 | 0.062 | 0.044 | 0.096 | 0.216 | 0.414 | 0.252 |
|  | PH 2 | Clearcut uniform buffer | 0.047 | 0.072 | 0.043 | 0.053 | 0.074 | 0.332 | 0.524 | 0.343 |
|  | PH 3 | Reference | 0.132 | 0.133 | 0.128 | 0.100 | 0.113 | 0.141 | 0.177 | 0.165 |
|  | PH 4 | Clearcut uniform buffer | 0.075 | 0.088 | 0.085 | 0.072 | 0.133 | 0.439 | 0.746 | 0.530 |
|  | PH Downstream | Downstream of harvested | 0.088 | 0.126 | 0.124 | 0.100 | 0.116 | 0.248 | 0.352 | 0.288 |
| **Rock** | RK 1 | Reference | 0.026 | 0.021 | 0.022 | 0.014 | 0.024 | 0.020 | 0.034 | 0.032 |
|  | RK 3 | Reference | 0.076 | 0.109 | 0.081 | 0.074 | 0.090 | 0.098 | 0.153 | 0.149 |
|  | RK Downstream | Downstream reference | 0.215 | 0.270 | 0.260 | 0.203 | 0.141 | 0.157 | 0.211 | 0.134 |
| **B. NH^4^ Concentrations (mg N/L)** | | | | | | | | | | |
| **Catchment** | **Watershed** | **Harvest treatment** | **2008** | **2009** | **2010** | **2011** | **2013** | **2014** | **2015** | **2016** |
| **Upper Main** | UM 1 | Reference | 0.001 | 0.002 | 0.009 | 0.002 | 0.002 | 0.004 | 0.004 | 0.002 |
|  | UM 2 | Clearcut variable buffer | 0.000 | 0.002 | 0.017 | 0.002 | 0.004 | 0.008 | 0.005 | 0.003 |
|  | UM 3 | Clearcut variable buffer | 0.009 | 0.001 | 0.025 | 0.003 | 0.005 | 0.001 | 0.004 | 0.006 |
|  | UM Downstream | Downstream of harvested | 0.005 | 0.003 | 0.013 | 0.002 | 0.004 | 0.003 | 0.002 | 0.002 |
| **Gus** | GS 1 | Reference | 0.002 | 0.004 | 0.008 | 0.002 | 0.003 | 0.003 | 0.001 | 0.001 |
|  | GS 2 | Thinned | 0.002 | 0.003 | 0.007 | 0.004 | 0.003 | 0.003 | 0.002 | 0.008 |
|  | GS 3 | Clearcut variable buffer | 0.004 | 0.003 | 0.009 | 0.003 | 0.008 | 0.008 | 0.011 | 0.002 |
|  | GS Downstream | Downstream of harvested | 0.003 | 0.005 | 0.007 | 0.006 | 0.008 | 0.001 | 0.002 | 0.006 |
| **Pothole** | PH 1 | Clearcut uniform buffer | 0.000 | -- | 0.002 | 0.003 | 0.017 | 0.005 | 0.003 | 0.003 |
|  | PH 2 | Clearcut uniform buffer | 0.029 | 0.003 | 0.008 | 0.001 | 0.001 | 0.006 | 0.002 | 0.005 |
|  | PH 3 | Reference | 0.005 | 0.006 | 0.011 | 0.004 | 0.003 | 0.004 | 0.004 | 0.004 |
|  | PH 4 | Clearcut uniform buffer | 0.000 | 0.005 | 0.008 | 0.002 | 0.002 | 0.001 | 0.004 | 0.004 |
|  | PH Downstream | Downstream of harvested | 0.001 | 0.006 | 0.013 | 0.008 | 0.003 | 0.006 | 0.003 | 0.003 |
| **Rock** | RK 1 | Reference | 0.002 | 0.003 | 0.004 | 0.003 | 0.004 | 0.001 | 0.002 | 0.004 |
|  | RK 3 | Reference | 0.000 | 0.002 | 0.009 | 0.003 | 0.006 | 0.001 | 0.001 | 0.001 |
|  | RK Downstream | Downstream reference | 0.000 | 0.003 | 0.020 | 0.035 | 0.008 | 0.001 | 0.002 | 0.003 |
