# Supplementary material for: Stream nitrate enrichment and increased light yet no algal response following forest harvest and experimental manipulation of headwater riparian zones
Source: PLoS One. 2023 Apr 20;18(4):e0284590. doi: 10.1371/journal.pone.0284590 (PMC10118188; doi:10.1371/journal.pone.0284590)
Supplement: S1 File — (DOCX) [file pone.0284590.s005.docx]

**S1 File. Details of Box-Cox transformations.**

Although normal residuals and homoscedasticity were not essential to our PLS analyses, we sought regression models with normally distributed residuals and homogeneity of variance using the Box-Cox transformation [1] and the program described below. The resultant models frequently achieved normality and homoscedasticity and often had higher R^2^CV than the untransformed response models. In the end, almost all of the final regression models used the Box-Cox transformation because of their higher R^2^CV.

The details of the procedure for the Box-Cox transformations are as follows:

Four tests were used to test for residual normality: Anderson-Darling [2], Jarque-Bera [3], Lilliefors [4], and Shapiro-Wilk [5]. The null hypothesis for the normality tests is that the input data is normally distributed i.e. a “small” p-value would lead one to reject the null hypothesis. A second assumption for regression models is homogeneity of variance (homoscedasticity). Levene’s test [6] was used to test for this, as it is more robust to departures from normality than Bartlett’s test [7]. The null hypothesis for Levene’s test is homogeneity.

Objective functions were then fashioned using the above tests so as to try to find the optimal Box-Cox 2-parameter transformation for each response variable. The transformation for response Y is (λ ≠ 0):

Y_λ,ν_ = ((Y+ν)^λ^ - 1)/ λ

For λ = 0, the transformation is log(Y + ν).

The optimization approach used genetic algorithms [8].

The objective function calculated p-values from the four normality tests and the p-values from three Levene’s tests.  There were three Levene’s tests as there were 3 grouping factors in every model, viz., two main effects plus the main effects interaction. Because the genetic algorithm we used is a minimizer, the final objective function was 1 minus the mean of the 7 p-values, unless one of two conditions was met. In the first condition, given our significance level of 0.05, if 3 of the 4 normality tests had p-values above 0.05 and the 3 Levene’s tests also had p-values above 0.05 the objective functions value was 0. The second condition differed from the first in that only 2 of the normality tests p-values were above 0.05. In this case the output value was 0.1.

1. Box GEP, Cox DR. An analysis of transformations. J R Stat Soc Ser B. 1964;26: 211–252.

2. Anderson TW, Darling DA. Asymptotic theory of certain “goodness of fit” criteria based on stochastic processes. Ann Math Stat. 1952;23: 193–212. doi:10.1214/aoms/1177729437

3. Jarque CM, Bera AK. Efficient tests for normality, homoscedasticity and serial independence of regression residuals. Econ Lett. 1980;6: 255–259. doi:10.1016/0165-1765(80)90024-5

4. Lilliefors HW. On the Kolmogorov-Smirnov test for normality with mean and variance unknown. J Am Stat Assoc. 1967;62: 399–402. doi:10.1080/01621459.1967.10482916

5. Shapire SS, Wilk MB. An analysis of variance test for normality (complete samples)†. Biometrika. 1965;52: 591–611. doi:10.1093/biomet/52.3-4.591

6. Levene H. Robust Tests for Equality of Variances. In: Olkin I, et al., editors. Contributions to Probability and Statistics: Essays in Honor of Harold Hotelling. Palo Alto, CA: Stanford University Press; 1960. pp. 278–292.

7. Snedecor GW, Cochran WG. Statistical Methods. 8th ed. Iowa: Iowa State University Press; 1989.

8. Michalewicz Z. Genetic Algorithms + Data Structures = Evolution Programs. 3rd ed. Berlin Heidelberg: Springer-Verlag; 1996. Available: https://doi.org/10.1007/978-3-662-03315-9
