# Supplementary material for: Stream nitrate enrichment and increased light yet no algal response following forest harvest and experimental manipulation of headwater riparian zones
Source: PLoS One. 2023 Apr 20;18(4):e0284590. doi: 10.1371/journal.pone.0284590 (PMC10118188; doi:10.1371/journal.pone.0284590)
Supplement: S2 File — (DOCX) [file pone.0284590.s006.docx]

**S2 File.** **Epilithon and chlorophyll *a* in June and July 2016.**

Sampling at a subset of sites occurred 30 days after regular June sampling in 2016 to examine if post-harvest responses of epilithon standing stocks and concentrations of chlorophyll a changed over the summer. A) Average epilithon AFDM in each study reach, plus standard deviation. B) Average chlorophyll *a* concentration in each study reach, plus standard deviation.


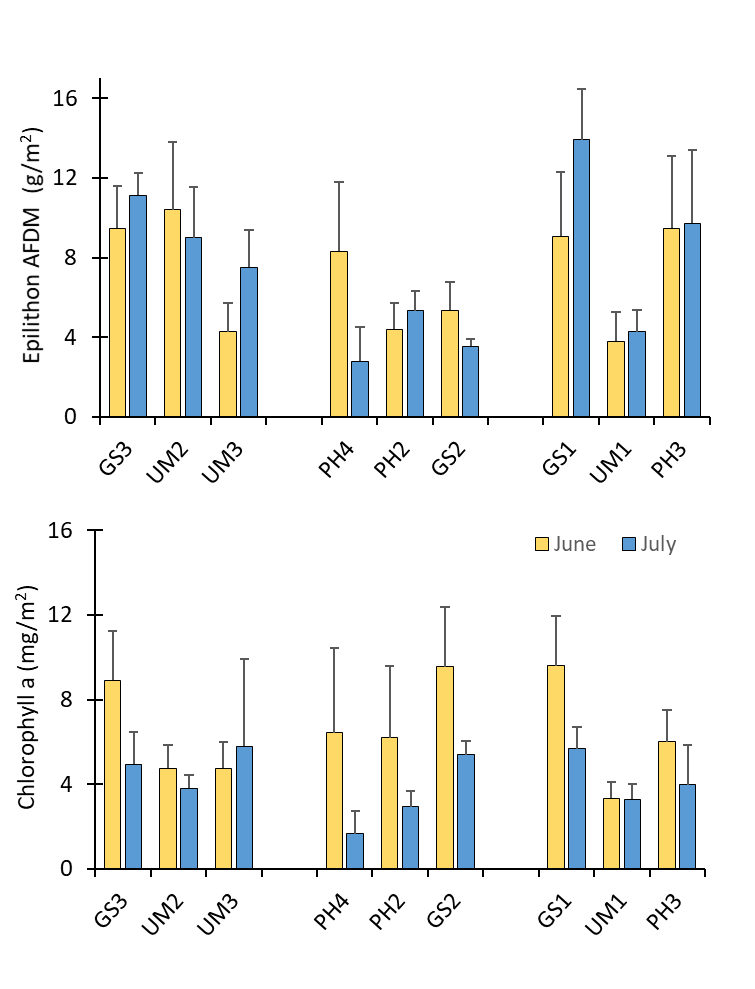


A

B

**Clearcut Clearcut Reference**

**Variable Uniform**
