# Supplementary material for: Stream nitrate enrichment and increased light yet no algal response following forest harvest and experimental manipulation of headwater riparian zones
Source: PLoS One. 2023 Apr 20;18(4):e0284590. doi: 10.1371/journal.pone.0284590 (PMC10118188; doi:10.1371/journal.pone.0284590)
Supplement: Attachment — Submitted filename: PONE-D-22-25727_R1_reviewer.pdf [file pone.0284590.s009.pdf]

PLOS ONE

Stream nitrate enrichment and increased light yet no algal response following forest harvest and experimental manipulation of headwater riparian zones

--Manuscript Draft--

**Manuscript Number:**	PONE-D-22-25727R1
**Article Type:**	Research Article
**Full Title:**	Stream nitrate enrichment and increased light yet no algal response following forest harvest and experimental manipulation of headwater riparian zones
**Short Title:**	Instream nutrient and algal responses to forest and riparian management
**Corresponding Author:**	Sherri L. Johnson, PhD<br>USDA Forest Service Pacific Northwest Research Station<br>Corvallis, Oregon UNITED STATES
**Keywords:**	Aquatic ecology; stream chemistry; nitrogen; epiliithon; forest harvest; disturbance; riparian; chlorophyll a; algae
**Abstract:**	Disturbances to forested watersheds often result in increases of nutrients and light to nearby streams. Such changes are generally expected to produce a shift to a more autotrophic aquatic ecosystem, with measurable increases in algae, and associated implications for food webs and fisheries. Although this paradigm is widely established, results from our 10-year study (2007-2016) in 12 headwater streams and four sites downstream in the Trask River Watershed (Oregon, USA), did not concur. In 2012, one watershed was thinned, three were clearcut harvested with variable buffers and three with uniform riparian buffers. After harvest, light to the stream surface significantly increased at the three watersheds with variable buffers while dissolved inorganic nitrogen (DIN) significantly increased in all of the clearcut harvested streams. Despite the increase in DIN and light, algal standing stocks and chlorophyll a concentrations did not significantly increase. The common assumption of increased autotrophic responses following increases of nitrogen and light was not supported here. We postulate the co-limitation of nutrients, driven by low phosphorus concentrations, which unlike DIN did not increase post-harvest, and the characteristics of the algal community, which were dominated by low light adapted diatoms rather than green algae, contributed to our findings of no responses for epilithon standing stocks of epilithic algae or chlorophyll a. The inclusion of multiple statistical analyses provided more certainty around our findings. This study documents responses to current forest practices and provides cautionary information for management and restoration activities aiming to increase abundances and standing stocks of fish by opening riparian canopies and adding nutrients.
**Order of Authors:**	Sherri L Johnson
	Alba Argerich
	Linda R Ashkenas
	Rebecca J Bixby
	David C Plaehn
**Response to Reviewers:**	I uploaded our response to reviewer and editor comments as a separate document. It is 25 pages of text, so best viewed as a document. I also addressed the editor comment about funding sources in our updated cover letter.
**Additional Information:**	
**Question**	**Response**
**Financial Disclosure**<br><br>Enter a financial disclosure statement that describes the sources of funding for the work included in this submission. Review	Support for the Trask River Watershed Study was primarily provided through the Oregon State University Watershed Research Cooperative, with long-term base funding from the Oregon Department of Forestry and Weyerhaeuser Corporation. Additional funding was provided by US Bureau of Land Management, US Forest Service Pacific Northwest Research Station, US Geological Survey Forest and

the submission guidelines for detailed requirements. View published research articles from *PLOS ONE* for specific examples.<br><br>This statement is required for submission and **will appear in the published article** if the submission is accepted. Please make sure it is accurate.<br><br>**Unfunded studies**<br>Enter: *The author(s) received no specific funding for this work.*<br><br>**Funded studies**<br>Enter a statement with the following details:<br>• Initials of the authors who received each award<br>• Grant numbers awarded to each author<br>• The full name of each funder<br>• URL of each funder website<br>• Did the sponsors or funders play any role in the study design, data collection and analysis, decision to publish, or preparation of the manuscript?<br>• **NO** - Include this sentence at the end of your statement: *The funders had no role in study design, data collection and analysis, decision to publish, or preparation of the manuscript.*<br>• **YES** - Specify the role(s) played.<br><br>* typeset	Rangeland Ecosystem Science Center, Oregon Department of Environmental Quality, Oregon Watershed Enhancement Board.<br><br>The funders provided support in the form of salaries for authors [SLJ, AA, LRA, RJB], but did not have any additional roles in the decisions about data collection, data analysis and interpretation, preparation of the manuscript and decision to publish.
**Competing Interests**<br><br>Use the instructions below to enter a competing interest statement for this submission. On behalf of all authors, disclose any competing interests that could be perceived to bias this work—acknowledging all financial support and any other relevant financial or non-financial competing interests.<br><br>This statement is required for submission and **will appear in the published article** if the submission is accepted. Please make sure it is accurate and that any funding sources listed in your Funding Information later in the submission form are also	The authors have declared that no competing interests exist.

Powered by Editorial Manager® and ProduXion Manager® from Aries Systems Corporation

declared in your Financial Disclosure statement.<br><br>View published research articles from *PLOS ONE* for specific examples.<br><br>**NO authors have competing interests**<br><br>Enter: *The authors have declared that no competing interests exist.*<br><br>**Authors with competing interests**<br><br>Enter competing interest details beginning with this statement:<br><br>*I have read the journal's policy and the authors of this manuscript have the following competing interests: [insert competing interests here]*<br><br>* typeset	
**Ethics Statement**<br><br>Enter an ethics statement for this submission. This statement is required if the study involved:<br><br>• Human participants<br>• Human specimens or tissue<br>• Vertebrate animals or cephalopods<br>• Vertebrate embryos or tissues<br>• Field research<br><br>Write "N/A" if the submission does not require an ethics statement.<br><br>General guidance is provided below. Consult the submission guidelines for detailed instructions. **Make sure that all information entered here is included in the Methods section of the manuscript.**	NA

**Format for specific study types**<br><br>**Human Subject Research (involving human participants and/or tissue)**<br>• Give the name of the institutional review board or ethics committee that approved the study<br>• Include the approval number and/or a statement indicating approval of this research<br>• Indicate the form of consent obtained (written/oral) or the reason that consent was not obtained (e.g. the data were analyzed anonymously)<br><br>**Animal Research (involving vertebrate animals, embryos or tissues)**<br>• Provide the name of the Institutional Animal Care and Use Committee (IACUC) or other relevant ethics board that reviewed the study protocol, and indicate whether they approved this research or granted a formal waiver of ethical approval<br>• Include an approval number if one was obtained<br>• If the study involved *non-human primates*, add *additional details* about animal welfare and steps taken to ameliorate suffering<br>• If anesthesia, euthanasia, or any kind of animal sacrifice is part of the study, include briefly which substances and/or methods were applied<br><br>**Field Research**<br><br>Include the following details if this study involves the collection of plant, animal, or other materials from a natural setting:<br>• Field permit number<br>• Name of the institution or relevant body that granted permission	
**Data Availability**<br><br>Authors are required to make all data underlying the findings described fully available, without restriction, and from the time of publication. PLOS allows rare exceptions to address legal and ethical concerns. See the PLOS Data Policy and FAQ for detailed information.	Yes - all data are fully available without restriction

Powered by Editorial Manager® and ProduXion Manager® from Aries Systems Corporation

A Data Availability Statement describing where the data can be found is required at submission. Your answers to this question constitute the Data Availability Statement and **will be published in the article**, if accepted.<br><br>**Important:** Stating 'data available on request from the author' is not sufficient. If your data are only available upon request, select 'No' for the first question and explain your exceptional situation in the text box.<br><br>Do the authors confirm that all data underlying the findings described in their manuscript are fully available without restriction?	
**Describe where the data may be found in full sentences. If you are copying our sample text, replace any instances of XXX with the appropriate details.**<br><br>• If the data are **held or will be held in a public repository**, include URLs, accession numbers or DOIs. If this information will only be available after acceptance, indicate this by ticking the box below. For example: *All XXX files are available from the XXX database (accession number(s) XXX, XXX.*).<br>• If the data are all contained **within the manuscript and/or Supporting Information files**, enter the following: *All relevant data are within the manuscript and its Supporting Information files.*<br>• If neither of these applies but you are able to provide **details of access elsewhere**, with or without limitations, please do so. For example:<br><br>*Data cannot be shared publicly because of [XXX]. Data are available from the XXX Institutional Data Access / Ethics Committee (contact via XXX) for researchers who meet the criteria for access to confidential data.*<br><br>*The data underlying the results presented in the study are available from (include the name of the third party*	These data are archived and publicly available through the US Forest Service Research Data Archive. Data and supporting documents are online and cited in the bibliography of the manuscript.<br><br>https://doi.org/10.2737/RDS-2022-0001<br><br>https://doi.org/10.2737/RDS-2022-0002<br><br>https://doi.org/10.2737/RDS-2022-0003

Powered by Editorial Manager® and ProduXion Manager® from Aries Systems Corporation

and contact information or URL).<br>• This text is appropriate if the data are owned by a third party and authors do not have permission to share the data.<br><br>* typeset	
Additional data availability information:	

Manuscript
Click here to access/download;Manuscript;Trask_manuscript_Nutrients_algae

Stream nitrate enrichment and increased light yet no algal response following forest harvest and experimental manipulation of headwater riparian zones

Sherri L. Johnson[1*], Alba Argerich[2], Linda R. Ashkenas[3], Rebecca J. Bixby[4], David C. Plaehn[5]

[1] Pacific Northwest Research Station, USDA Forest Service Corvallis, Oregon, United States of America

[2] School of Natural Resources, University of Missouri, Columbia, Missouri, United States of America

[3] Department of Fisheries and Wildlife, Oregon State University, Corvallis, Oregon, United States of America

[4] Department of Biology, University of New Mexico, Albuquerque, New Mexico, United States of America

[5] Pilot Records, Corvallis, Oregon, United States of America

*Corresponding author

E-mail: sherri.johnson2@usda.gov

Keywords

Stream chemistry, nitrogen, algae, epilithon, forest harvest, disturbance, riparian, buffers

Abstract

Disturbances to forested watersheds often result in increases of nutrients and light to nearby streams. Such changes are generally expected to produce a shift to a more autotrophic aquatic ecosystem, with measurable increases in algae, and associated implications for food webs and fisheries. Although this paradigm is widely established, results from our 10-year study (2007-2016) in 12 headwater streams and four sites downstream in the Trask River Watershed (Oregon, USA), did not concur. In 2012, one watershed was thinned, three were clearcut harvested with variable buffers and three with uniform riparian buffers. After harvest, light to the stream surface significantly increased at the three watersheds with variable buffers while dissolved inorganic nitrogen (DIN) significantly increased in all of the clearcut harvested streams. Despite the increase in DIN and light, algal standing stocks and chlorophyll *a* concentrations did not significantly increase. The common assumption of increased autotrophic responses in stream food webs following increases of nitrogen and light was not supported here. We postulate the co-limitation of nutrients, driven by low phosphorus concentrations, which unlike DIN did not increase post-harvest, and the characteristics of the algal community, which were dominated by low light adapted diatoms rather than green algae, contributed to our findings of no responses for epilithon standing stocks of epilithic algae or chlorophyll *a*. The inclusion of multiple statistical analyses provided more certainty around our findings. This study documents responses to current forest practices and provides cautionary information for management and restoration activities aiming to increase abundances and standing stocks of fish by opening riparian canopies and adding nutrients.

Introduction

Terrestrial disturbances in headwaters and the role of riparian forests in maintaining stream integrity

Researchers have long worked to understand instream responses associated with differing types of watershed disturbances, including forest harvest [1,2], fire [3,4], hurricanes [5], agriculture [6], and urbanization [7]. Forests and woodlands cover large portions of landscapes; across the Unites States, they comprise one-third of the land base [8]. Forested watersheds managed for timber production (including harvest) some 67% of the forested lands in the USA [8]. Evaluation of the effects of forest harvest on streams ecosystems is therefore an important and necessary focus of research and monitoring [9]. Streams flowing through forests provide habitat for a diverse array of aquatic organisms [10,11] as well as serve as sources of drinking water for downstream communities [12]. Because headwater streams represent 80-90% of miles of any given river network [13], reducing impacts from forest harvest to headwater streams can have broad implications locally as well as downstream [11,14].

Forested riparian zones provide a variety of functions [15,16] including buffering aquatic ecosystems from disturbances within their watersheds [9,17]. Intact riparian vegetation can stabilize stream banks [18], provide shade to maintain cool water and air temperatures [19,20], minimize direct solar radiation that can potentially stimulate algal blooms [2,21], intercept nutrients and sediment transported from hillslopes [22,23], and deliver allochthonous detrital material, including wood, leaves, and needles [24,25]. Changes to riparian vegetation and/or the widths of riparian buffers during natural or anthropogenic disturbances can result in shifts in some or all of these functions, potentially leading to impacts on multiple trophic levels of instream biotic communities [26–29] and water quality for downstream water users [9,30].

Management of riparian forests to reduce instream responses to harvest

The assumptions and observations of instream responses to riparian management are numerous and sometimes conflicting, which can make it challenging to identify best management practices and regulatory guidelines during forest harvest. Identifying the optimal widths of riparian buffers necessary for protection of instream functions is complicated by multiple site-specific factors capable of influencing riparian-stream interactions [23,31] as well as which stream response parameter or process is of most interest [17,32,33]. For example, headwater forested streams frequently have low background levels of stream nutrients [34,35] and following harvest in forested watersheds, increases in dissolved nitrogen, phosphorus and carbon have been observed [36–40]. Increases in stream chemistry concentrations have been associated with increased instream sediment [29,39,40], especially when riparian buffers were not retained during harvest. However, not all studies have observed increased stream nutrients or stream sediment following forest harvest [35,41,42]. Retention of vegetated riparian buffers during harvest has been suggested as a way to mitigate these increases [16,43,44].

The removal of riparian vegetation during harvest has also been frequently associated with increases in stream algae [45,46]. Riparian harvest, and the accompanying changes in instream light and possibly nutrients [45,47,48] can result in higher biomass of attached algae and increased concentrations of chlorophyll *a* [49,50]. In studies of forest streams, an increase in algae has been linked to higher densities of macroinvertebrates and fish [47,49,51–53]. Depending on site specific interactions, increases in nutrients and potential co-limitation between nitrogen and phosphorus can influence algal biomass [54,55]. The taxonomic composition of

algal assemblages can shift in response to changes in light and nutrients; several studies have documented a shift from diatom communities to filamentous green algae following clearcutting [45,46,56]. Although the relationships among reduced riparian cover, increased light and increased algal standing stocks are frequently assumed to be ubiquitous [21,57], a subset of research papers have noted that algal responses to increased light and nutrients can be negligible [46,58,59]. It has been suggested that null findings that do not support this common paradigm are less frequently published [58]; this would influence the information available for new studies as well as support for assumptions associated with instream responses to forest management.

Compounding the complexity of instream responses to forest harvest is the inflexibility of commonly used statistical tests to capture biological variability and statistical significance in dynamic systems such as streams. Traditional statistical analyses have generally been developed for controlled studies with high replication and constant variance. The statistical assumptions for parametric analyses of normally distributed, homoscedastic data, randomly assigned treatments and independent measurements may not be appropriate for field studies of landscape-scale experiments with low sample sizes. Yet the statistical significance associated with natural resource questions is highly relevant to forest planning and policy, which has led to multiple instances of controversies about the best statistics [60–63]. Given the potential complexity of types of statistical assessments, research that informs best management practices, including management of riparian vegetation during harvest, would ideally address these multifaceted dynamics with multiple, rigorous evaluations of responses to current forest harvest practices.

In this landscape-scale experimental study, we use multiple statistical tests to examine whether differences in the width of riparian buffers retained during clearcut harvest preserve water quality and whether stream ecosystems shift to include more autochthonous resources. Our

hypotheses were that removal of some or all riparian vegetation would lead to a) higher concentrations of instream nutrients and dissolved organic carbon, b) higher chlorophyll *a* concentration and algal biomass in response to more available light and nutrients, and c) increased instream sediment as a result of disturbances to hillslope soils from harvest activities. We also hypothesized that responses within a harvested area might propagate to downstream sites.

Methods

Site description and experimental design

Study sites were located in the Trask River Watershed Study area, within the upper 25 km^2 of the South Fork of the Trask River in Oregon, USA (Fig 1). The Trask River originates in the northern Oregon Coast Range and flows westward into the Pacific Ocean. The underlying geology is a mixture of sedimentary deposits and volcanic basalt intrusions [64]. Annual rainfall in the upper Trask study area averages 200 cm per year, primarily between October and May, with occasional snow at higher elevation sites. At the beginning of this study, the hillslope and riparian forests in the Trask River Watershed Study area were 45-60 year old Douglas-fir (*Pseudotsuga menziesii*) and red alder (*Alnus rubra*). Big leaf maple (*Acer macrophyllum*) occurred only in riparian areas. This landscape had been heavily impacted by prior disturbances; a series of wildfires occurred between 1933 and 1951 and following the fires, all burned and unburned trees were harvested and the area replanted. In addition to impacts from fires and harvest, land management legacies included a high density of logging roads, minimal downed wood in streams and riparian areas, and a simplified understory plant community.

The Trask River Watershed Study was conducted by researchers and natural resource managers as a multi-disciplinary, 10+ yr. collaborative study to understand and quantify the onsite effects of current timber harvest practices on physical, chemical, and biological communities in first-order headwater streams. We also evaluated whether responses to headwater harvest propagated downstream to third-order fish-bearing streams. This study was conducted using a paired study design of 12 small watersheds nested within four large catchments (Fig 1). Within each large catchment, two to three small headwater watersheds (17-67 ha) were clearcut harvested over as much of the watershed area as possible, while one headwater watershed remained as an unharvested reference site (Table 1). In addition, no harvests occurred in one of the larger catchments (Rock Creek) so that it could serve as a reference site for the downstream comparisons. The pre-harvest sampling occurred 2007-2011. In 2012, harvest took place in seven headwater watersheds and no sampling occurred. Post-harvest, sampling restarted in 2013 and continued through 2016.

Fig 1. Map of study watersheds in Trask River Watershed Study, western Oregon. Locations of headwater sampling reaches are shown as triangles and downstream sites are noted as squares. Map was created using ArcGIS Pro with LiDAR terrain (https://www.oregongeology.org/lidar/) and derived hydrography. Reference watersheds are shown in dark green, clearcut with variable buffers are shown as orange and clearcut with uniform buffers are show as blue. Light green watershed was thinned with wide unharvested buffers. Created using ArcGIS Pro with LiDAR terrain (https://www.oregongeology.org/lidar/) and LiDAR-derived hydrography.

Table 1. Study site characteristics.

Catchment	Watershed	Harvest treatment [1]	Ownership	Elevation Study Site (m)	WS Area (ha)	Percent WS harvested in 2012	Distance to downstream site (m)	Ave channel slope (%)	Ave wetted channel width (m)	Ave water depth (cm)	Ave discharge (L/s)[2]
Upper Main	UM 1	Reference	Private	658	44.2	0%	406	18.7	1.47	6.8	6.6
	UM 2	Clearcut variable buffer	Private	685	17.1	91%	1140	18.4	1.16	5.8	5.9
	UM 3	Clearcut variable buffer	Private	728	39.2	56%*	1581	11.8	1.34	6.9	5.2
	UM Downstream	Downstream of harvested	Private	608	278.8	25%	--	3.8	3.63	14.1	15.1
Gus	GS 1	Reference	Private	611	26.5	0%	1059	12.1	1.32	6.1	3.7
	GS 2	Thinned	Federal	638	39.0	91%	1355	14.5	1.82	7.0	5.6
	GS 3	Clearcut variable buffer	Private	789	20.4	92%	2297	22.4	1.20	4.2	1.2
	GS Downstream	Downstream of harvested	State	470	301.4	30%	--	10.2	4.16	15.4	27.6
Pothole	PH 1	Clearcut uniform buffer	State	531	67.1	87%	2352	13.6	2.00	10.1	-
	PH 2	Clearcut uniform buffer	State	493	20.4	82%	2159	13.7	1.00	6.2	4.9
	PH 3	Reference	State	390	45.4	0%	1044	12.8	0.93	5.5	4.3
	PH 4	Clearcut uniform buffer	State	370	23.6	92%	947	13.8	0.80	5.2	1.5
	PH Downstream	Downstream of harvested	State	324	324.6	44%	--	2.8	3.30	13.0	19.4
Rock	RK 1	Reference	Private	680	42.2	0%	4385	7.6	1.36	9.5	11.7
	RK 3	Reference	State	548	35.3	0%	2388	16.2	1.66	9.9	12.3
	RK Downstream	Downstream reference	State	334	669.9	0%	--	3.5	4.58	16.3	43.1

[1] **Harvest details:**

Clearcut variable buffer: A riparian buffer was not required on these sites. During harvest, leave trees remained in the downstream riparian areas of UM2 and UM3

Clearcut uniform buffer: Clearcut or modified clearcut with a uniform 12 m riparian buffers on each side of the stream

Thinned: Thinned watershed with a 15 m no-harvest riparian buffer on each side of the stream

Reference: Not harvested during this study period. Vegetation age was 45-60 yrs.

[2] Discharge was measured with conservative salt tracers at the same time as the water chemistry and epilithon sampling.

* A portion of this WS had been harvested 6 years previously

Riparian treatments during forest harvest differed by land ownership (Fig 1; Table 1). On private forest lands, riparian buffers were not required at the time of this study [65,66] and the watersheds owned by forest industry (UM3, UM2, and GS3), were clearcut with variable amounts of riparian vegetation retained. Forest harvest guidelines for protection of terrestrial wildlife require the equivalent of two live trees per acre not be harvested [65]. In two watersheds (UM2 and UM3), the trees designated for wildlife were grouped in the riparian areas for operational reasons including to avoid steep banks or wet soils. We termed this treatment group as "Clearcut variable".

During harvest on State Forest lands (PH1, PH2, PH4), the ODF Forest Management Plan required minimum 7.6 m buffers along each side of the perennial section of the stream channel [67]. In addition, some of the required trees designated for wildlife were grouped adjacent to the buffers which led to final buffers of approximately 12 m [19,68]. We characterized these sites as clearcut with uniform width buffers, and termed this treatment group as "Clearcut uniform".

The thinned watershed (GS2) was on federal land, managed by the Bureau of Land Management. Harvest practices involved thinning throughout the watershed using helicopter logging and retaining a 15 m, no-entry buffer on each side of the stream. This site is termed thinned with uniform buffer and abbreviated as "Thinned". No additional forest treatments beyond these described occurred during the study period. More information is available as supplementary material with the archived data.

The locations of the study reaches were designed to spatially coincide with collaborative studies, which included stream temperature [19,69], suspended sediment [64,70], stream chemistry during storms [71], stream discharge, macroinvertebrates [68], amphibians [72] and fish [73]. In the harvested watersheds, the study reaches were located within the downstream

most portion of the treated area. In addition, to assess potential cumulative impacts of harvest in upstream headwaters, four downstream study reaches were located in larger third-order fish-bearing streams, one in each of the catchments (Fig 1). Three study reaches were downstream of harvested watersheds, at distances of 947-2297 m (Table 1): UMDS was downstream of sites clearcut with variable buffers, PHDS was downstream of sites clearcut with uniform buffers and GSDS had mixture of harvest groups upstream. Rock Creek, RKDS, was the reference for the downstream sites.

Each year, the sampling for the research occurred in early summer to provide as coincident measurements of concentration, biomass, or abundance as possible for all of the study groups. In early summer, streamflow was sufficient at all headwater sites for consistent biotic sampling to occur. Sampling schedules were established to prevent unintended impacts of each type of sampling on subsequent projects. The Trask headwater sites did not contain fish, but all were inhabited by an amphibian predator, Coastal Giant salamander (*Dicamptodon tenebrosus*), and a subset of the sites contained an amphibian grazer, Coastal Tailed Frog tadpoles (*Ascaphus truei)*. At downstream sites, resident fish species included Cutthroat Trout (*Onchorhynchus clarkia clarkii*) and Sculpin (*Cottus* spp.). Two of the downstream sites also contained anadromous Coho Salmon (*Oncorhynchus kisutch*) and Steelhead (*Onchorhynchus mykiss*) on a seasonal basis.

Data collection

Stream characterization

Sampling occurred annually during the last two weeks of June between 2007-2011 (pre-harvest period) and 2013-2016 (post-harvest period). On each sampling date, at each of the 12

headwaters and 4 downstream sampling sites, we collected basic measurements of stream physical characteristics. Channel dimensions were measured at a series of 4-transects over a 30 m reach in headwaters and at 10 transects over 100 m reach at downstream sites. Discharge at the time of sampling was calculated using a constant addition of a conservative salt tracer [74]. We avoided sampling during rain events. Water temperatures were measured continuously from June-September using recording thermistors (Onset Computer Corporation, model U22-001) protected from direct radiation [19]. Reach slope was measured in the field using a stadia rod and clinometer (PM-5, Suunto) in 2008 and 2013. At each sampling time, the average percentage of canopy closure was measured at each transect from the center of the stream using a handheld concave densiometer [75]. In addition, in 2008 (pre-harvest) and 2013 (immediately after harvest), we quantified the amount of overstory light reaching the stream using hemispherical photos. The hemispherical photos were recorded using a self-leveling camera with hemispherical lens that was positioned mid-channel and 1m above the water surface. A total of five photos were taken per reach with photos spaced 25 m apart in headwater channels and 50 m apart at downstream sites. Photos were taken at dawn or dusk and when there were no clouds. In 2008, the position of camera point was carefully documented so that post-harvest images could be collected from the same position and height. The percent of overstory light reaching the stream was calculated for each image using a digital image analyzer (Metric used: percent total site factor; WinSCANOPY, Regent Instruments, Inc.) and the site average calculated.

Water chemistry

At each headwater and downstream site during the annual June sampling, we collected a grab sample of stream water for analyses of water chemistry. The samples were stored on ice in the

field, and then filtered through pre-combusted Whatman GF/F filters (pore size = 0.7μm) in the laboratory within 3 days of collection. After filtering, the samples were frozen for later analysis at the Oregon State University Cooperative Chemical Analytical Laboratory (see http://ccal.oregonstate.edu/methodology for description of analytical methods and detection limits). Analytes reported here include dissolved organic carbon (DOC), soluble phosphorus (SRP), dissolved inorganic nitrogen (DIN; sum of nitrate, nitrite, and ammonium), dissolved organic nitrogen (DON; calculated as the difference between total dissolved nitrogen and DIN), total dissolved nitrogen (TDN) and total dissolved phosphorus (TDP).

Algae

Each year in late June, four composite samples of epilithic algae, hereafter termed epilithon, were collected at each of the 12 headwater sites and six composite samples were collected at each of the four downstream sites. Epilithon is a complex matrix of algal cells on stream substrates; it potentially includes bacteria, fungi, and fine detrital matter. The epilithon samples were analyzed for photosynthetic pigments (chlorophyll *a* concentration) and standing stock (biomass). For each composite sample, we used a small wire brush to scrub a known area of randomly selected rocks from riffles, glides, and cascades, but not pools. Each composite contained material scrubbed from four to twelve rocks, with a total sample area of at least 50 cm^2. Each rock was gently lifted out of the water and a flexible plastic template with a known area was held onto the rock, exposing only the area to be scrubbed. After scrubbing, the loosened epilithic material within the template area was gently rinsed into the sample container. The total area scrubbed from multiple rocks was recorded for each composite sample. Sample containers were placed on dry ice in the field, frozen, and analyzed within one month of collection. In

2016, additional sampling for epilithon and chlorophyll *a* was conducted in late July at sites with variable buffer, uniform buffer, and a subset of reference sites.

In the lab, each sample was split into two aliquots after thawing; the aliquot for epilithon biomass, as ash free dry mass (AFDM), was sieved, using 2 mm mesh, to remove large, non-algal particles, and filtered onto pre-combusted and pre-weighed GF/F filters (Whatman glass microfiber filters). These filters were then dried at 60°C, weighed, combusted at 500°C, and reweighed. The second aliquot was analyzed for chlorophyll *a* and phaeophytin concentration by filtering a known volume onto pre-combusted GF/F filters, and using the heated ethanol methodology for pigment extraction [76]. All measurement of concentrations of pigments occurred using a Shimadzu UV-1700 UV-VIS spectrophotometer. Chlorophyll *a* pigment concentrations were corrected for acid strength and post-acidification time [77].

We also collected algal samples for analysis of algal taxonomic structure from sites. At each sampling time, a fifth composite sample was collected as described above and preserved in 0.5% formalin. Samples from 2011 (pre-harvest) and 2013 (post-harvest) at a subset of sites were selected for analysis. The dominant algal genera were qualitatively noted in wet mounts in a Palmer Maloney chamber [78]. To determine diatom densities, 4-ml aliquots were heated with 25 ml of 30% hydrogen peroxide for one hour and rinsed six times with distilled water to remove oxidation by-products. Processed subsamples were evaporated onto coverslips and mounted to microscope slides with Z-Rax mounting medium, making permanent slides. Specimens along transects were examined under oil immersion at 1000× magnification using a Leica DM5000 compound microscope with differential inference contrast microscopy. Five hundred valves were identified to species and enumerated from each sample. Diatoms were identified to the lowest taxonomic level (usually species), using standard taxonomic literature (https://diatoms.org/) and

regional literature [79,80]. Identifiable fragments were mathematically reconstituted to whole valve units when possible and included in total counts. The raw data were converted to densities and classified by growth forms, based on Rimet and Bouchez [81].

Benthic sediment

Each sampling period, three randomly located samples of fine benthic sediment were collected within the 30 m headwater sites following LINX II protocols [82]. Five samples were collected from the downstream sites. This benthic sediment sampling was sequenced before the algal sampling to avoid substrate disturbance and resuspension of sediment during other instream sampling. After collection, samples were refrigerated and returned to the laboratory. Sediment samples were dried and weighed for calculations of mass of fine surface sediment per square meter.

Data analyses

We compared multiple statistical approaches to examine whether forest harvest treatments resulted in differences in responses between the pre- and post-harvest periods. We grouped our headwater study sites by four treatments – clearcut with variable buffer width (variable buffer; 0-7.5 m), clearcut with uniform buffer width (uniform buffer; 12 m), thinned with uniform buffer width (thinned; 15 m), and reference (no harvest during this study). Downstream sites were characterized into two groups based on whether there was harvest in the headwaters or no harvest. As well as examining treatment groups, we also modelled differences in responses between pre- and post-harvest periods at individual headwater sites. Our analyses and models examined responses of chlorophyll *a* concentration, epilithon biomass, surface sediment,

concentrations of DOC, DON, DIN, and SRP, DIN:SRP molar ratio, and canopy cover following treatments relative to pre-harvest. We also calculated Pearson correlations between epilithon, chlorophyll *a*, and key instream factors. A significance level of 0.05 was used for all tests.

Kolmogorov-Smirnov test and Bootstrapped Means

An initial step was to compare the pre- and post-harvest cumulative distribution functions for each treatment group (clearcut variable, clearcut uniform, thinned, and reference) using the non-parametric Kolmogorov-Smirnov 2-sample test (KS2) [83]. This test identifies whether the distributions are different, but does not provide information about direction of difference. We next evaluated whether the 95% confidence intervals (CI) for the means for pre- and post-harvest responses within a treatment overlapped using a bias-corrected, accelerated (BCa) bootstrap [84]. We considered pre- and post-harvest means significantly different if their bootstrapped confidence intervals did not overlap and noted the direction of the post-harvest change. These analyses were only conducted for treatment groups, because sample sizes for individual watershed sites pre- and post-harvest were small.

Partial Least Squares and significance testing

Our third approach involved modeling the differences in responses between pre- and post-harvest periods using Partial Least Squares regression (PLS) [85] to further evaluate differences in instream responses. Partial Least Squares is a latent variable approach that maximizes regressor and regressand covariance and avoids problems with multi-collinearity. It creates multiple models for a given data set using latent variables, which can be thought of as similar to principal components. The criterion for selecting the “optimal” PLS model, essentially a notable change in slope of the root-mean-square-error of cross-validation (RMSECV) graph, which is usually at or

near the minimum RMSECV (maximum R^2CV). If the R^2CV of a given model was negative or "small," it was not used regardless of the R^2. Full (leave-one-out) cross-validation was used in the PLS modeling. Cross-validation allows for calculation of the R^2 of cross-validation (R^2CV), which we interpret as a more robust measure of model quality [86,87].

Categorical factors of treatment group and harvest period, or watershed and harvest period were converted to dummy variables and the last level then deleted to avoid linearly dependent regressors. The interactions were then created from these dummy variables. For the model examining effects of forest harvest treatments, Response = Treatment + Period + Treatment*Period, where "Period" refers to harvest pre-post and "*" indicates the interaction between factors. This resulted in a regressor array with three Treatments, one Period and three interactions and allowed seven PLS latent variables, and therefore, seven regression models to be created. The downstream model was the same symbolically as the headwater treatment model except that there were only two categories in the treatment factor. For the headwater watershed model (Response = Watershed + Period + Watershed*Period), the regressor array had eleven Watersheds, one Period and eleven Watershed-Period interactions.

We used Box-Cox transformations [88] to try to improve model quality as defined by R^2CV (S1 File). The R^2CV of the resulting models was typically better than those of models that did not use transformed responses or more standard transformations, such as the log transformation.

In a standard regression model, one would generally assume the sites were independent of one another. In this study, this assumption was likely violated due to annual sampling at each site as well as the spatial distribution of treatments across watersheds which was a function of forest ownership. To address this concern, we employed Wu's jackknife [89] and the wild bootstrap [90,91] for significance testing. For Wu's jackknife, we used one of Wu's best performing

methods (“full cross validation”). This provided a coefficient variance estimate, which was then used in a 2-sided t-test for each coefficient for each model. We also used the wild bootstrap method HC_3 to construct 95% confidence intervals from 2000 re-samplings. The wild bootstrap confidence intervals were used to compare model pre- and post- predictions, where two predictions were considered significantly different if their 95% confidence intervals did not intersect. For regression coefficient testing, if a given confidence interval did not include zero, then the null hypothesis (that the coefficient was zero) was rejected. Note that neither the semi-parametric Wu jackknife nor the non-parametric wild bootstrap require normally distributed model residuals.

For calculations involving degrees of freedom for PLS models, the method described in Krämer and Sugiyama [92] was employed. Both the regressor and response data were centered and scaled. All analyses were conducted in Matlab™ 2020b (Mathworks, 2020) using Matlab programs, except Box-Cox, genetic algorithm, wild bootstrap, and Wu jackknife analyses, which were written by D. Plaehn.

Results

Stream characterization

Headwater streams were steep (mean slope 14.6%) and shallow (mean depth 6.9 cm), with wetted widths ranging from 0.8 m to 2.0 m (Table 1). Downstream sites had lower gradients (mean slope 5.1%), and were wider (mean width 3.9 m) and deeper (mean depth 15.0 cm). At the time of sampling, streamflow ranged from 1.2 L/s to 6.6 L/s, in headwater streams, with slightly higher discharge in the two reference watersheds in Rock Creek, whereas downstream sites

showed larger discharge (ranging from 15 – 43 L/s). Streamflow across years was fairly constant during the three weeks preceding sampling with an average coefficient of variation of 22% among watersheds and years.

During the pre-harvest period, the percent of overstory light reaching the streams was low (average 15.5%) and comparatively uniform across sites (range 9-19%, Fig 2). After harvest in 2013, sites clearcut with variable buffers showed increased light reaching the stream. The annual measurements of percent canopy closure collected with a densiometer showed that canopy closure at clearcut sites with variable buffers was significantly different pre- and post-harvest in all three statistical approaches (Table 2, S1 Table). The three individual watersheds in the variable buffer group each showed significant changes in canopy closure pre- to post-harvest (Table 3). We did not observe significant changes in canopy closure for other treatments or for other individual watersheds.

Fig 2. Percent of light reaching streams pre- and post-harvest. The percent of overstory light reaching the streams, measured using hemispherical photos in 2008, pre-harvest, and in 2013, post-harvest. Sites are organized from left to right by percentage of overstory light reaching the stream post-harvest. Bars show site averages and standard deviation.

Pre-harvest, maximum June water temperatures ranged between 7.3^{o}C and 15.3^{o}C in headwater streams. In the post-harvest period, maximum June temperatures were warmer than pre-harvest across all sites. Streams clearcut with variable buffers showed greater increases (3.9^{o}C, 2.5^{o}C, and 3.1^{o}C) than streams with uniform buffers (1.1^{o}C, 0.7^{o}C, 1.0^{o}C), thinned (1.2^{o}C) or reference streams (0.4^{o}C ,1.1^{o}C, 0.9^{o}C, 0.7^{o}C, 1.4^{o}C) (Fig 3A). In mid-summer, post-

Fig 3. Stream temperature and fine surface sediment. Headwater sites are organized by harvest treatment and organized from left to right by the percentage of overstory light reaching the stream post-harvest. A) Average daily maximum stream temperatures in June during pre and post-harvest periods. B) Mass of fine surface sediment at annual sampling in June, for pre- and post-harvest periods. Bars show site averages and standard deviation.

harvest temperatures at streams that were clearcut with variable buffers were significantly warmer than pre-harvest [19].

In the headwater streams, substrates were primarily gravel to cobble with some areas of bedrock. The average mass of fine benthic sediment (Fig 3B) did not significantly change post-harvest in any of the treatment groups (Table 2), within individual headwater watersheds (Fig 3B, Table 3, S2 Table) or at downstream sites (S3 Table).

Table 2. Statistical differences between pre- and post-harvest periods for headwater treatment groups.

Treatment group	Canopy closure	DOC	SRP	DON	DIN	DIN:SRP Molar Ratio	Epilithon biomass	Chl a	Surface sediment
**A. Kolmogorov-Smirnov two sample test p-values**									
Clearcut variable	**0.00***	0.43	0.43	0.99	**0.00***	**0.02***	0.98	0.92	1.00
Clearcut uniform	0.57	0.86	0.96	0.24	**0.00***	**0.00***	0.17	**0.05***	0.54
Thinned	0.48	1.00	0.11	0.53	0.11	0.11	0.26	0.88	0.26
Reference	0.77	0.50	0.77	0.28	0.50	0.77	**0.04***	0.06	0.38
**B. Comparison of 95% BCa Bootstrap confidence intervals**									
Clearcut variable	**decrease**	0	0	0	**increase**	**increase**	0	0	0
Clearcut uniform	0	0	0	0	**increase**	**increase**	0	0	0
Thinned	0	0	0	0	0	0	0	0	0
Reference	0	0	0	0	0	0	0	**decrease**	0
**C. Pre-Post comparison of PLS Model predictions**									
Clearcut variable	**B/C***	--	C/C	A/AB	**C/AB***	**B/A***	--	AB/AB	--
Clearcut uniform	AB/AB	--	A/A	AB/A	**C/A***	**C/AB***	--	A/AB	--
Thinned	AB/B	--	BC/BC	AB/AB	**C/B***	BC/AB	--	A/AB	--
Reference	A/AB	--	B/AB	AB/AB	BC/BC	BC/ABC	--	AB/B	--

An asterisk and bold text indicates significant differences between pre- and post-harvest periods. B). "0" indicates the pre- and post-harvest confidence intervals (CI) overlapped; "increase" indicates that the lower bound of the post-harvest CI was greater than the upper bound of the pre-harvest CI. "decrease" where pre-harvest CI was greater than post-harvest. C). Pre-post comparisons of significance for PLS model predictions for each attribute were based on 95% confidence intervals using the wild bootstrap. Within an attribute, pre/post with the same letters are not significantly different. "A" denotes the largest value, "B" is second largest, etc. within a response variable. The lettering on PLS model predictions is based on comparing the Box-Cox model predictions, not transformed back the original scale. " --" indicates that the R^2CV was negative and therefore the significance was not reported. Dissolved organic carbon (DOC), Soluble reactive phosphorus (SRP), Dissolved organic nitrogen (DON), Dissolved inorganic nitrogen (DIN), Chlorophyll a (Chl a).

Water chemistry

During our annual early summer sampling, concentrations of dissolved nutrients and DOC in these forested headwater streams were low and varied among watersheds. Pre- and post-harvest responses were not significantly different for headwater treatment groups for DOC and DON (Table 2) or for individual headwater watersheds (Table 3). Average DOC ranged from 0.240 to 0.883 mg C/L (Fig 4A). Because the R^2 CV was negative for DOC in the treatment group PLS model (S1 Table), the significance for the PLS model was not reported for DOC (Table 2C). Average DON varied from 0.005 to 0.083 mg N/L (Fig 4B). DIN showed significant increases post-harvest at all clearcut treatment groups (Table 2[illegible]N concentrations post-harvest in clear-cut treatments were approximately six times greater than pre-harvest, relative to a slight increase at the reference and thinned watersheds post-harvest (1.3x and 1.7x, respectively) (Fig 4C). The greatest change pre- to post-harvest in DIN occurred at GS3 where average concentration increased from 0.008 mg N/L pre-harvest to 0.334 mg N/L in the fourth year post-harvest (S4 Table). DIN was predominately composed of nitrate (S4 Table); concentrations of nitrate in 2013, the first year post-harvest were not the highest observed in the post-harvest period. SRP concentrations showed very little change following harvest (Fig 4D, Table 2). Some watersheds, PH2, PH3, and RK1, had substantially higher concentrations than others. Pre- to post-harvest changes in DIN:SRP molar ratio mirrored the increases in DIN for watersheds clearcut with variable buffers, but showed much smaller increases post-harvest in the clearcut with uniform buffer group (Fig 4E). Molar ratios for both clearcut treatment groups were significantly greater post-harvest (Table 2), but in the analysis for individual watersheds, only GS3 and UM2 were

significantly higher (Table 3). The average molar ratios of DIN:SRP concentrations in the headwaters ranged from 2.5 to 161 pre-harvest and up to 377 post-harvest at GS3 (Fig 4E).

Fig 4. Average concentrations of dissolved nutrients and carbon at headwater sites. Sites are organized from left to right by percentage of overstory light reaching the stream post-harvest. A) Dissolved organic carbon (DOC), B) Dissolved organic nitrogen (DON). C) Dissolved inorganic nitrogen (DIN), D) Soluble reactive phosphorus (SRP), E) Molar ratios of DIN:SRP. Bars show averages plus standard deviation during early summer annual sampling. Asterisks indicate significant differences for watersheds using PLS analyses (Table 3).

Table 3. Statistical summary from PLS model comparing pre- to post-harvest responses of headwater watersheds.

Treatment group	Watershed	Canopy closure	DOC	SRP	DON	DIN	DIN:SRP Molar Ratio	Epilithon biomass	Chl *a*
Clearcut variable	GS3	**BC/D***	CD/CD	G/G	ABC/ABC	**D/AB***	**C/A***	AB/AB	AB/ABC
Clearcut variable	UM2	**AB/C***	BC/BC	G/FG	ABC/ABC	**CD/A***	**BC/A***	AB/AB	BC/C
Clearcut variable	UM3	**B/C***	BC/BC	E/E	BC/BC	CD/BC	BC/BC	B/BC	B/BC
Clearcut uniform	PH1	ABC/BC	AB/A	E/E	ABC/ABC	**CD/AB***	C/BC	BC/CD	AB/ABC
Clearcut uniform	PH2	AB/AB	BC/ABC	A/AB	BC/C	**CD/AB***	C/C	BC/CD	ABC/BC
Clearcut uniform	PH4	AB/AB	C/CD	DE/D	BC/C	**C/A***	B/BC	AB/ABC	BC/BC
Thinned	GS2	AB/ABC	BC/ABC	FG/FG	B/BC	CD/BC	BC/BC	BC/CD	AB/A
Reference	UM1	ABC/ABC	**D/E***	FG/FG	BC/ABC	D/D	C/C	**C/D***	C/C
Reference	GS1	AB/AB	AB/AB	FG/F	AB/A	A/A	AB/A	AB/AB	AB/AB
Reference	PH3	A/A	CD/CD	B/B	ABC/BC	B/B	C/C	A/AB	AB/ABC
Reference	RK1	BC/BC	CD/CD	C/C	ABC/ABC	D/D	C/C	BC/CD	BC/C
Reference	RK3	AB/AB	B/AB	FG/FG	BC/C	BC/BC	BC/B	BC/C	BC/C

Significance in PLS models is based on 95% confidence intervals calculated using Wild Bootstrap. Within a site, pre/post comparisons with the same letters are not significantly different. An asterisk and bold text indicates significant differences between pre- and post-harvest periods. "A" denotes the largest value, "B" is second largest, etc within a response variable. The lettering on PLS model predictions is based on comparing the Box-Cox model predictions, not transformed back the original scale. Dissolved organic carbon (DOC), Soluble reactive phosphorus (SRP), Dissolved organic nitrogen (DON), Dissolved inorganic nitrogen (DIN), Chlorophyll *a* (Chl *a*).

The PLS model for individual watersheds showed significant post-harvest increases in DIN in four of the six clearcut watersheds (Table 3). The thinned watershed, GS2, showed a significant increase in DIN post-harvest in the PLS model of treatment groups but not in the watershed PLS model. Because the watershed PLS model had a higher R^2CV than the associated model of treatment groups, the watershed model could be viewed as more reliable.

Nutrient and DOC concentrations at downstream sites were very similar to those in the headwaters (Fig 5). As observed at headwater sites, downstream DOC, SRP, and DON did not show significant differences between pre- and post-harvest periods (S3 Table). The increased concentration of DIN post-harvest in clearcut headwaters did not result in a consistent significant increase of DIN at their downstream study sites; the analysis of overlapping 95% confidence intervals showed DIN to be significantly greater post-harvest period downstream of harvested headwaters, but the other analyses did not (S3 Table). Interestingly, the downstream of reference site showed significant differences for DIN and DIN:SRP molar ratios post-harvest. The comparison of 95% confidence intervals and the PLS Models indicated that the DIN concentrations decreased post-harvest at the reference site, possibly related to dryer and warmer conditions. Comparisons from the PLS model were not reported for DOC, DON, or molar ratio; the R^2CVs were negative, indicating that the model quality was poor (S3 Table).

Fig 5. Average concentrations of nutrients at downstream sites during pre- and post-harvest periods. A) Dissolved organic carbon (DOC), B) Dissolved organic nitrogen (DON), C) Dissolved inorganic nitrogen (DIN), D) Soluble reactive phosphorus (SRP), E) Molar ratio DIN:SRP. Bars show averages plus standard deviation during early summer annual sampling. RKDS is the reference for the downstream sites. UMDS is downstream of variable buffer sites, PHDS is downstream of uniform buffer sites and GSDS has mixture of harvest groups upstream.

Epilithic algal biomass and chlorophyll *a*

Average epilithon biomass at headwater sites ranged from 3.8-14.3 g AFDM/m^2 (Fig 6A). Post-harvest, epilithon biomass was not significantly different from pre-harvest levels in any of the harvested headwater groups (Table 2) or individual headwater watersheds that were harvested(Table 3). A reference watershed (UM1) had significantly lower biomass post-harvest (Table 3) and likely influenced KS2 test that showed significantly different distribution for the reference group (Table 2). In the PLS watershed model, the multi-comparison lettering (Table 3) based on wild bootstrap prediction confidence intervals, was indicative of a general decrease in epilithon during the post-harvest period, in agreement with Fig 6. The R^2 CV was zero for epilithon in the PLS model for headwater treatments (S1 Table), therefore the model significance was not reported (Table 2C).

Fig 6. Average epilithon biomass and chlorophyll *a* concentration at headwater sites. Sites are organized from left to right by percentage of overstory light reaching the stream post-harvest. A) Average epilithon biomass as ash free dry mass (AFDM) plus standard deviation; B) Average chlorophyll *a* concentration plus standard deviation. Samples were collected annually in early summer. Asterisks indicate significant differences using PLS analyses (Table 3).

Average chlorophyll *a* concentration ranged from 3.3-13.0 mg/m^2 (Fig 6B). There were no significant differences in chlorophyll *a* pre- to post-harvest in clearcut with variable buffer group or in the thinned treatment (Table 2). The KS2 analyses, which compares distributions but not means, was the only test that suggested significant differences for post-harvest chlorophyll *a* in the clearcut with uniform buffer treatment (Table 2A). The Reference group showed a significant decrease in the comparison of 95% BCa confidence internals (Table 2B). Although PLS analysis of Treatment groups showed no significant change for any group (Table 2C), the PLS model

R^2CV for chlorophyll *a* was low, suggesting weak predictive power (S1 Table). No significant differences were identified for individual headwater watersheds (Table 3).

An analysis of the algal cell counts from a subset of headwater sites found that the June algal community was primarily diatoms in pre-harvest and post-harvest samples (Table 4); no filamentous algae were present. *Achnanthidium minutissimum* was the most common taxon in the epilithic algal samples at three of the five sites where taxa were identified pre- and post-harvest. At four of the five sites, one of the two most common species was the same in pre- and post-harvest periods. No clear shifts in growth form were noted among the treatments.

Table 4. The most common and second most common algal taxa in selected headwater streams, based on cell counts of epilithon samples.

Treatment group	Watershed	Period	Most Common Algal Taxon [1]	Second Most Common Algal Taxon [1]
Clearcut variable	GS3	Pre	*Gomphonema minutum* (H)	*Meridion circulare* (L)
		Post	*Achnanthidium minutissimum* (L)	---
Clearcut variable	UM2	Pre	*Planothidium lanceolatum* (L)	*Achnanthidium minutissimum* (L)
		Post	*Achnanthidium minutissimum* (L)	*Achnanthidium cf. linearis* (L)
Clearcut variable	UM3	Pre	*Achnanthidium minutissimum* (L)	*Achnanthidium cf. deflexum* (L)
		Post	*Achnanthidium minutissimum* (L)	*Achnanthidium cf. linearis* (L)
Clearcut uniform	PH4	Pre	*Rhoicosphenia abbreviatum* (L)	*Nitzschia inconspicua* (L)
		Post	*Rhoicosphenia abbreviatum* (L)	*Planothidium lanceolatum* (L)
Reference	UM1	Pre	*Gomphonema minutum* (H)	*Planothidium lanceolatum* (L)
		Post	*Planothidium lanceolatum* (L)	*Gomphonema minutum* (H)

[1] Growth forms are indicated as low (L) and high (H) profiles, based on classification by Rimet and Bouchez, 2012 [81].

At the downstream sites, epilithon biomass and chlorophyll *a* concentration in the downstream of harvested group were generally lower in the post-harvest period (Fig 7). The group of sites ‘downstream of harvested” showed significantly different distributions for both epilithon mass and chlorophyll *a* concentration between pre- and post-harvest periods using the KS2 test (S3 Table) and epilithon biomass significantly decreased in the analysis of 95% confidence intervals. Using the PLS model of pre- to post-harvest comparisons, neither epilithon nor chlorophyll *a* showed significant differences across these time periods.

Fig 7. Average epilithon biomass and concentration of chlorophyll a at downstream sites during pre- and post-harvest periods. A) Epilithon biomass as ash free dry mass (AFDM); B) Chlorophyll *a* concentration. Bars show early summer average plus standard deviation. RKDS was the reference for the downstream sites. UMDS was downstream of variable buffer sites, PHDS was downstream of uniform buffer sites and GSDS had mixture of harvest groups upstream.

Chlorophyll *a* concentrations were significantly correlated with epilithon biomass during pre-harvest period (R^2 =0.446, Fig 8a; R^2=0.521, Fig 8B). However, during the post-harvest period, the correlations were significant only for reference sites (R^2 =0.444, Fig 8B). Chlorophyll *a* was not significantly correlated with canopy closure at reference or harvested sites, pre- or post-harvest (Fig 8C, D). Epilithon was not correlated with DIN at harvested sites (Fig 8E); only at reference sites during post-harvest period was the correlation between epilithon and DIN significant (R^2=0.300, Fig 8F). The correlation between epilithon and DIN:SRP molar ratios was significant for harvested sites in the post-harvest period (R^2 =0.149, Fig 8G) and likely greatly influenced by several high molar ratios. No correlations for epilithon and ratios at reference sites (Fig 8H).

Fig 8. Correlations between chlorophyll *a*, epilithon and instream factors for headwater sites. These correlations compare site averages for each year and site during pre- and post-harvest periods. Headwater sites that were harvested are in the left column (A, C, E, G) and those were reference sites are in the right column (B, D, F, H). Chlorophyll *a* versus epilithon (A, B); Chlorophyll *a* versus canopy closure (C, D); Epilithon versus DIN (E, F); Epilithon versus molar ratios of DIN:SRP (Dissolved Inorganic Nitrogen: Soluble Reactive Phosphorus) (G, H). Data from pre-harvest period are indicated as dark green circles and from the post-harvest period are shown as triangles. R^2 values are included for the correlations where p-values equal to or less than 0.05.

Discussion

Retention of vegetated riparian buffers can reduce impacts of forest harvest on stream ecosystems. In the Trask River Watershed study, all streams in harvested forests showed increased DIN regardless of the width of riparian buffers, and no change in concentrations of dissolved organic carbon, soluble reactive phosphorus, or mass of benthic sediment. Canopy closure remained similar to the pre-harvest period at the sites with uniform 12 m buffers but dramatically increased at the sites with variable riparian buffers. Despite increased levels of both light and dissolved inorganic nitrogen in clearcut watersheds with variable riparian buffers, we did not observe increased standing stocks of epilithon or higher concentrations of chlorophyll *a*, in contrast to the common expectation of increased autotrophic resources in response to increased light and nutrients. Headwater streams have been overlooked in many prior regulatory discussions; therefore these findings are valuable in expanding our understanding of what conditions and factors are major drivers of responses in small headwater streams. Though our use of multiple analyses, we have greater confidence in the statistical significance of these relevant management practices.

The challenges of distinguishing statistical significance in landscape experiments

There are multiple challenges to distinguishing statistical significance in field experiments. Research into responses to landscape treatments are conducted over a large area and for a number of years. These large-scale field experiments are expensive and labor-intensive and consequently have low sample sizes [37,39,93], and the focal metrics often exhibit high spatial and temporal variability [54]. Our study design planned that individual watersheds would be replicates of a specific harvest treatment, but study watersheds are never true replicates [34]. Our study sites within each treatment group showed variability in site conditions and responses [19,68]; and in this study, we explored this variability by including examination of pre- and post-harvest responses within each watershed. In addition to treatments, we included reference watersheds to control for the influence of variable climatic conditions during the 10 years of the study. The analyses of treatment groups were crucial for evaluation of forestry best management practices, while the analyses of individual watersheds addressed the variability in responses across sites.

Our results from this complex landscape-scale experiment have potentially broad implications for forest management and policy issues. We recognize that statistical significance becomes a major factor for the incorporation of research findings into policy and management; To address the need of statistical robustness, we used multiple statistical approaches to assess pre-and post-harvest differences with the belief that consensus among methods would provide stronger evidence than any one method by itself. The use of multiple methods was further warranted given the data challenges of spatial and temporal dependency, small sample sizes, and the potential of not finding an acceptable PLS model given the stringent quality criterion of

R^2CV. Multiple statistical tests allowed us to evaluate means (BCa bootstrap) and distributions (KS2) for pre-post comparisons, and simultaneously assess responses across all treatments or watersheds using PLS models. The non-parametric KS2 and BCa bootstrap both used small sample sizes which can be problematic [94–96], but by bootstrapping, the confidence intervals were considered conservative (i.e. larger) for non-independent data [97]. Our multi-analysis approach leveraged the study design of pre-post, reference-treatment data, but unlike most Before-After-Control-Intervention (BACI) and repeated measures tests; our analyses of significance did not depend upon normally distributed data for comparisons.

Stream chemistry responses to riparian and watershed harvest

The increase in DIN concentrations concur with results from other studies of stream chemistry responses to forest management [35,37,98,99]. Early studies in non-nitrogen limited regions found extremely high concentrations of nitrate following harvest [37,42,100], especially during storm flows. Our sampling timing was focused on early summer, non-storm periods, which in these oligotrophic ecosystems, is a time when nutrients are generally at their minimum concentrations [101,102]. The magnitude of DIN increase in this study was substantial relative to the pre-harvest period, but the absolute concentrations were relatively low and comparable to studies in western US that documented small increases in nitrogen after forest harvest without riparian buffers [35,39,93].

Significant increases in DIN concentrations occurred even where riparian buffers were retained. Maintaining forested riparian zones during watershed harvest is a widely recognized best management practice for protecting water quality by reducing nutrient and sediment delivery to streams [9,16]. Stream nitrogen increases following removal of vegetation have been

hypothesized to be driven by a reduced demand for nitrate where there is less vegetation, coupled with mobilization of nitrate related to soil disturbance, decaying organic matter and microbial nitrification [38,99]. The effectiveness of riparian buffers to intercept, retain and process nutrients has been related to the width of the buffer, with the recognition that multiple other landscape factors, including slope, soil type, and depth of groundwater also provide key functions [16,23,43]. Our high-gradient watersheds have a mix of soil types [64] and a legacy of soil disturbances related to historical wildfire and subsequent salvage logging [19,70] that may have altered connections between subsurface hillslope flow paths and rooting zones of riparian vegetation [16]. Although transport of inorganic nitrogen to the Trask streams may not have been fully intercepted in watersheds with wider buffers, the wider buffers provided other water quality functions, including shade that prevented stream temperature increases [19].

Responses of carbon and nutrients other than DIN to forest harvest and removal of riparian buffers have been mixed. Similar to findings from other studies in the Pacific Northwest [42,100,104], we did not see increased concentrations of DON, DOC, or phosphorus. However, in other regions such as boreal forests, researchers have observed increased concentrations and export of DON and DOC following harvest [40,101]. Although increases of stream phosphorus following harvest are not common [42], adsorption of P to soil and DOC can enhance its mobility and export [101], with site specific differences in soil and vegetation type likely controlling these dynamics. Even where phosphorus occurs naturally at high concentrations, including western Cascade Mountains [42,106], an increase in phosphorus concentrations has not generally been observed following forest harvest or watershed disturbances [35,101,107], unless substantial transport of sediment occurred [35,39].

Downstream propagation of responses to headwater disturbances can be a concern [11] and has been observed for water quality [103,104], especially during stormflows [39]. In nitrogen-limited ecosystems, nitrogen spiraling, uptake, and transformation would be expected to reduce instream concentrations of N as the water flows downstream [105,106]. At the Trask sites downstream of harvest, downstream propagation of upstream DIN increases was not definitive; our statistics showed mixed results, albeit with small sample sizes.

Lack of epilithic algal responses

Increased nutrients [45,78,107,108] as well as increased light [29,109] have long been recognized to stimulate autotrophic responses in aquatic systems. When increased nutrients coincide with increased light, many studies have documented an increase in autochthonous resources and productivity instream [21,29,48,55]. Therefore, at our sites where both DIN and light increased post-harvest, we expected but did not observe increased standing stocks of epilithon and/or changes in chlorophyll *a*. We surmised that lack of response might be related to limitation of individual nutrients and/or co-limitation of nitrogen and phosphorus [58,59,107]. Although the Trask DIN concentrations increased after harvest and approached proposed "breakpoint" and saturation concentrations associated with high chlorophyll values[110], phosphorus concentrations in the Trask (except in PH2, PH3, and RK1) were generally lower than those noted by other studies and used for predicting algal standing stocks and chlorophyll *a* [110,111]. Historically, stream water N:P ratios (evaluated here as DIN:SRP) have been proposed as predictors of algal responses and have been included regularly in comparisons [55,110,112], while others have suggested that they might not be strong indicators of nutrient limitation [108]. Across our wide range of N:P ratios across sites, some of which were in

optimum ranges for algal growth, epilithic algal stocks didn't increase following harvest. Our correlations between algae responses and potential drivers of increased algae showed few relationships. In very small streams, co-limitation may involve multiple mechanisms including nutrients [58,107] in combination with light [113] plus other factors.

The taxonomic composition of algal assemblages can influence their responses to disturbance [108]. Multiple studies of responses to forest harvest have observed taxonomic shifts and increased filamentous green algae following the opening of the riparian canopy [2,45,46] and deposition of benthic sediment [56], yet the Trask algal assemblages were consistently dominated by small, low growing diatoms during pre-and post-harvest periods. *Achnanthidium minutissimum* is considered an early successional species, that competes well as in shaded headwater streams with low nutrients and high water velocities [79,114] but can be replaced by other taxa when inorganic nutrients and light increase [115]. Yet our small study streams at the upper tips of the stream network had minimal green algal taxa to take advantage of post-harvest changes. Diatoms are frequently used as indicators of water quality and watershed condition [79,114,116]; the diatoms in the Trask streams were indicators of high water quality and cold waters. Some diatom taxa are more responsive than others to changes in nutrients; Stelzer and Lamberti [112] noted that *Achnanthidium minutissimum* showed sensitivity to N:P ratios while other diatom taxa, such as *Gomphonema* sp., were sensitive to only increases in nitrate. Increased light availability can lead to shifts in community composition from low, prostrate to high upright algal growth forms (e.g., Bixby et al. [117]), yet the Trask algal assemblages showed no shifts in growth forms associated with increased light availability. Light flecks [57] in shaded stream beds, including reference sites, may have supported microhabitats of algal assemblages with higher profile taxa (e.g., *Gomphonema minutum*).

We wondered if the lack of observed responses in epilithon could be due to the timing of our samplings (i.e., early summer), as seasonality can be a major factor for composition of diatom assemblages as well as green algae and summer is the time of highest activity and biomass [59,108]. To assess if the timing of our sampling affected the results about epilithic responses, we examined the algal standing stocks and chlorophyll *a* concentrations later in the summer when temperatures were warmer and flows lower. However, additional sampling in July showed generally lower chlorophyll *a* and similar biomass of epilithon as in June (S2 Text), thereby confirming that we were not missing substantial algal responses post-harvest.

A final consideration of influences of autochthonous response to disturbances involves the potential influences of instream grazers [55,109,115]. The Trask stream food webs communities had low abundances of grazers and macroinvertebrate scrapers and grazers did not increase post-harvest [68], as might have been expected if more autochthonous resources became available. Tailed frog tadpoles were present in only 3 of our 12 headwaters sites (UM2, PH1 and PH2) [72], and their presence did not explain differences in chlorophyll *a* or standing stocks of algae as has been observed elsewhere [109].

Harpole et al. [58] noted that minimal or null responses of autotrophic community to increased light and nutrients "are rarely discussed in the nutrient limitation literature". In our review of the primary literature related to algal responses to disturbances, we found only few studies where increased nitrogen or combination of light and nitrogen did not lead to increased algal standing stocks and chlorophyll *a* [59,118,119]. The lack of studies showing null responses to increased light and nutrients could be partially attributed to a bias in the published literature towards studies that show responses after disturbances or treatments [54,55,107,120]. The few studies where null responses to increased nutrients and light were documented have suggested

the lack of observed response was due to low phosphorus [118] or seasonally shifting N:P ratios [108,121]. Frequently, natural resource managers and some researchers have expectations that removal of forest cover or increases in nutrients will result in straightforward and predictable algal increases that transfer to upper trophic levels [55] for more productive fisheries [49,51–53,122], based on the general paradigm that light and nutrients stimulate instream primary producers. Meta-analyses [54,55] have suggested responses to nutrient enrichment are contingent on background concentrations of inorganic N and P with interactions among nutrients, light and turbidity.

Conclusions and management implications

The evolution of forest harvest methods and improvements in Best Management Practices have resulted in smaller magnitude of post-harvest impacts than historically observed [9,18,19,100]. However, the stakes to quantify the impacts of forest management accurately and to inform best management practices and policies continue to be high and regularly associated with controversy [32,61,123], especially in landscapes with mixed ownerships and management objectives. Our inclusion of multiple analytical tests allowed us to examine the variability in responses and the concurrence of statistical responses. This provided more certainty around our findings and better information for managers and policy makers in their planning and evaluations of responses to management.

Disturbance from forest harvest in these small watersheds led to increased instream dissolved nitrogen broadly while the wider riparian buffers maintained lower light levels representative of forested headwater streams. However, this study reminds us that not all streams respond similarly to forest harvest and removal of riparian vegetation; our results did not fit the general

expectation that increased light and or increased stream nitrogen after watershed disturbances stimulate algal responses. We postulate that multiple factors, including co-limitation of nutrients, driven by low phosphorus concentrations, which did not change, plus an algal community dominated by low light adapted diatoms rather than green algae, contributed to our findings of no change in biomass of epilithon or concentrations of chlorophyll *a*. Management and restoration activities which aim to increase abundances and standing stocks of fish by opening riparian canopies and adding nutrients would benefit from prior evaluation of algal community composition and co-limitation of nutrients before undertaking major modifications.

Acknowledgements

Bob Bilby, Liz Dent, Maryanne Reiter, Jason Dunham, Judy Li, Janel Sobota, Arne Skaugsat, and Bill Gerth were invaluable research partners. We thank Rich Van Driesche, Kylie Meyer, Brent Morrissette, Tim Glidden, Jenn King, Mark Meleason, and Katie Whitehead for multi-year field assistance plus many others too numerous to list. We appreciate the analyses, field maps and Fig 1 by David Hockman-Wert. Any use of trade, firm, or product names is for descriptive purposes only and does not imply endorsement by the U.S. Government.

References

1. Moore RD, Richardson JS. Natural disturbance and forest management in riparian zones: comparison of effects at reach, catchment, and landscape scales. Freshw Sci. 2012;31: 239–247. doi:10.1899/11-030.1

2. Sabater S, Butturini A, Munoz I, Romani A, Wray J. Effects of removal of riparian vegetation on algae and heterotrophs in a Mediterranean stream. J Aquat Ecosyst Stress Recovery. 1998;6: 129–140.

3. Bixby RJ, Cooper SD, Gresswell RE, Brown LE, Dahm CN, Dwire KA. Fire effects on aquatic ecosystems: an assessment of the current state of the science. Freshw Sci. 2015;34: 1340–1350. doi:10.1086/684073

4. Rodriguez-Cardona BM, Coble AA, Wymore AS, Kolosov R, Podgorski DC, Zito P, et al. Wildfires lead to decreased carbon and increased nitrogen concentrations in upland arctic streams. Sci Rep. 2020;10: 8722. doi:10.1038/s41598-020-65520-0

5. Schaefer DA, McDowell WH, Scatena FN, Asbury CE. Effects of hurricane disturbance on stream water concentrations and fluxes in eight tropical forest watersheds of the Luquillo Experimental Forest, Puerto Rico. J Trop Ecol. 2000;16: 189–207. Available: https://www.jstor.org/stable/3068756

6. Kaushal SS, Mayer PM, Vidon PG, Smith RM, Pennino MJ, Newcomer TA, et al. Land use and climate variability amplify carbon, nutrient, and contaminant pulses: a review with management implications. J Am Water Resour Assoc. 2014;50: 585–614. doi:10.1111/jawr.12204

7. Gardiner EP, Sutherland AB, Bixby RJ, Scott MC, Meyer JL, Helfman GS, et al. Linking stream and landscape trajectories in the southern Appalachians. Environ Monit Assess. 2009;156: 17–36. doi:10.1007/s10661-008-0460-x

8. Oswalt SN, Smith WB, Miles PD, Pugh SA. Forest Resources of the United States, 2017: a technical document supporting the Forest Service 2020 RPA Assessment. Gen Tech Rep WO-97 Wash DC US Dep Agric For Serv Wash Off. 2019;97. doi:10.2737/WO-GTR-97

9. Cristan R, Aust WM, Bolding MC, Barrett SM, Munsell JF, Schilling E. Effectiveness of forestry best management practices in the United States: Literature review. For Ecol Manag. 2016;360: 133–151. doi:10.1016/j.foreco.2015.10.025

10. Penaluna BE, Olson DH, Flitcroft RL, Weber MA, Bellmore JR, Wondzell SM, et al. Aquatic biodiversity in forests: a weak link in ecosystem services resilience. Biodivers Conserv. 2016; 1–31.

11. Freeman MC, Pringle CM, Jackson CR. Hydrologic connectivity and the contribution of stream headwaters to ecological integrity at regional scales1. JAWRA J Am Water Resour Assoc. 2007;43: 5–14. doi:10.1111/j.1752-1688.2007.00002.x

12. Liu N, Dobbs GR, Caldwell PV, Miniat CF, Sun G, Duan K, et al. Quantifying the role of National Forest System and other forested lands in providing surface drinking water supply for the

conterminous United States. Gen Tech Rep WO-100 Wash DC US Dep Agric For Serv Wash Off. 2022;100. doi:10.2737/WO-GTR-100

13. Downing JA, Cole JJ, Duarte CM, Middelburg JJ, Melack JM, Prairie YT, et al. Global abundance and size distribution of streams and rivers. Inland Waters. 2012;2: 229–236. doi:10.5268/IW-2.4.502

14. Roon DA, Dunham JB, Torgersen CE. A riverscape approach reveals downstream propagation of stream thermal responses to riparian thinning at multiple scales. Ecosphere. 2021;12: e03775. doi:10.1002/ecs2.3775

15. Riis T, Kelly-Quinn M, Aguiar FC, Manolaki P, Bruno D, Bejarano MD, et al. Global overview of ecosystem services provided by riparian vegetation. BioScience. 2020;70: 501–514. doi:10.1093/biosci/biaa041

16. Sweeney BW, Newbold JD. Streamside forest buffer width needed to protect stream water quality, habitat, and organisms: a literature review. JAWRA J Am Water Resour Assoc. 2014;50: 560–584. doi:10.1111/jawr.12203

17. Gregory SV, Swanson FJ, McKee WA, Cummins KW. An ecosystem perspective of riparian zones: focus on links between land and water. BioScience. 1991;41: 540–551. doi:10.2307/1311607

18. Hatten JA, Segura C, Bladon KD, Hale VC, Ice GG, Stednick JD. Effects of contemporary forest harvesting on suspended sediment in the Oregon Coast Range: Alsea Watershed Study Revisited. For Ecol Manag. 2018;408: 238–248. doi:10.1016/j.foreco.2017.10.049

19. Reiter M, Johnson SL, Homyack J, Jones JE, James PL. Summer stream temperature changes following forest harvest in the headwaters of the Trask River watershed, Oregon Coast Range. Ecohydrology. 2020;13: e2178. doi:10.1002/eco.2178

20. Roon DA, Dunham JB, Groom JD. Shade, light, and stream temperature responses to riparian thinning in second-growth redwood forests of northern California. Rossi S, editor. PLOS ONE. 2021;16: e0246822. doi:10.1371/journal.pone.0246822

21. Gregory SV. Effects of light, nutrients and grazers on periphyton communities in streams. Oregon State University. 1980.

22. Baker ME, Weller DE, Jordan TE. Improved methods for quantifying potential nutrient interception by riparian buffers. Landsc Ecol. 2006;21: 1327–1345. doi:10.1007/s10980-006-0020-0

23. Vidon PGF, Hill AR. Landscape controls on nitrate removal in stream riparian zones. Water Resour Res. 2004;40. doi:10.1029/2003WR002473

24. Gregory S, Boyer KL, Gurnell AM, International Conference of Wood in World R. The ecology and management of wood in world rivers. Bethesda, Md.: Bethesda, Md. : American Fisheries Society; 2003.

25. Benfield EF. Comparison of litterfall input to streams. J North Am Benthol Soc. 1997;16: 104–108. doi:10.2307/1468242

26. Dudley MP, Solomon K, Wenger S, Jackson CR, Freeman M, Elliott KJ, et al. Do crayfish affect stream ecosystem response to riparian vegetation removal? Freshw Biol. 2021;66: 1423–1435. doi:10.1111/fwb.13728

27. Erdozain M, Kidd KA, Emilson EJS, Capell SS, Kreutzweiser DP, Gray MA. Forest management impacts on stream integrity at varying intensities and spatial scales: Do abiotic effects accumulate spatially? Sci Total Environ. 2021;753: 141968. doi:10.1016/j.scitotenv.2020.141968

28. Richardson JS, Béraud S. Effects of riparian forest harvest on streams: a meta-analysis. J Appl Ecol. 2014;51: 1712–1721. doi:10.1111/1365-2664.12332

29. Murphy ML, Hawkins CP, Anderson NH. Effects of canopy modification and accumulated sediment on stream communities. Trans Am Fish Soc. 1981;110: 469–478.

30. Dodds WK, Bouska WW, Eitzmann JL, Pilger TJ, Pitts KL, Riley AJ, et al. Eutrophication of u.s. freshwaters: analysis of potential economic damages. Environ Sci Technol. 2009;43: 12–19. doi:10.1021/es801217q

31. Luke SH, Luckai NJ, Burke JM, Prepas EE. Riparian areas in the Canadian boreal forest and linkages with water quality in streams. Environ Rev. 2007;15: 79–97. doi:10.1139/A07-001

32. Richardson JS, Naiman RJ, Bisson PA. How did fixed-width buffers become standard practice for protecting freshwaters and their riparian areas from forest harvest practices? Freshw Sci. 2012;31: 232–238. doi:10.1899/11-031.1

33. Graham EB, Averill C, Bond-Lamberty B, Knelman JE, Krause S, Peralta AL, et al. Toward a generalizable framework of disturbance ecology through crowdsourced science. Front Ecol Evol. 2021;9. Available: https://www.frontiersin.org/articles/10.3389/fevo.2021.588940

34. Argerich A, Johnson SL, Sebestyen SD, Rhoades CC, Greathouse E, Knoepp JD, et al. Trends in stream nitrogen concentrations for forested reference catchments across the USA. Environ Res Lett. 2013;8: 014039. doi:10.1088/1748-9326/8/1/014039

35. Binkley D, Ice GG, Kaye J, Williams CA. Nitrogen and phosphorus concentrations in forest streams of the United States. JAWRA J Am Water Resour Assoc. 2004;40: 1277–1291. doi:10.1111/j.1752-1688.2004.tb01586.x

36. Swank WT. Stream Chemistry Responses to Disturbance. In: Swank WT, Crossley DA, editors. Forest Hydrology and Ecology at Coweeta. New York, NY: Springer; 1988. pp. 339–357. doi:10.1007/978-1-4612-3732-7_25

37. Likens GE, Bormann FH, Johnson Noye M, Fisher DW, Pierce Robert S. Effects of forest cutting and herbicide treatment on nutrient budgets in the Hubbard Brook Watershed Ecosystem. Ecol Monogr. 1970;40: 23–47. doi:10.2307/1942440

38. Feller M. Forest harvesting and streamwater inorganic chemistry in western North America: A review. JAWRA J Am Water Resour Assoc. 2005;41: 785–811. doi:10.1111/j.1752-1688.2005.tb03771.x

39. Gravelle JA, Ice G, Link TE, Cook DL. Nutrient concentration dynamics in an inland Pacific Northwest watershed before and after timber harvest. For Ecol Manag. 2009;257: 1663–1675. doi:10.1016/j.foreco.2009.01.017

40. Schelker J, Eklöf K, Bishop K, Laudon H. Effects of forestry operations on dissolved organic carbon concentrations and export in boreal first-order streams. J Geophys Res Biogeosciences. 2012;117. doi:10.1029/2011JG001827

41. Martin CW, Harr RD. Logging of mature Douglas-fir in western Oregon has little effect on nutrient output budgets. Can J For Res. 1989;19: 35–43. doi:10.1139/x89-005

42. Aubertin GM, Patric JH. Water quality after clearcutting a small watershed in West Virginia. J Environ Qual. 1974;3: 243–249. doi:10.2134/jeq1974.00472425000300030012x

43. Mayer PM, Reynolds Jr. SK, McCutchen MD, Canfield TJ. Meta-analysis of nitrogen removal in riparian buffers. J Environ Qual. 2007;36: 1172–1180. doi:10.2134/jeq2006.0462

44. Hill AR. Nitrate removal in stream riparian zones. J Environ Qual. 1996;25: 743–755. doi:10.2134/jeq1996.00472425002500040014x

45. Lowe RL, Golladay SW, Webster JR. Periphyton response to nutrient manipulation in streams draining clearcut and forested watersheds. J North Am Benthol Soc. 1986;5: 221–229. doi:10.2307/1467709

46. Shortreed KS, Stockner JG. Periphyton biomass and species composition in a coastal rainforest stream in British Columbia: Effects of environmental changes caused by logging. Can J Fish Aquat Sci. 1983;40: 1887–1895. doi:10.1139/f83-219

47. Hill WR, Knight AK. Nutrient and light limitation of algae in two Northern California streams. J Phycol. 1988;24: 125–132.

48. Fanta SE, Hill WR, Smith TB, Roberts BJ. Applying the light:nutrient hypothesis to stream periphyton. Freshw Biol. 2010;55: 931–940. doi:10.1111/j.1365-2427.2009.02309.x

49. Murphy ML, Hall JD. Varied effects of clear-cut logging on predators and their habitat in small streams of the Cascade Mountains, Oregon. Can J Fish Aquat Sci. 1981;38: 137–145. doi:10.1139/f81-018

50. Kiffney PM, Richardson JS, Bull JP. Responses of periphyton and insects to experimental manipulation of riparian buffer width along forest streams. J Appl Ecol. 2003;40: 1060–1076.

51. Kaylor MJ, Warren DR. Linking riparian shade and the legacies of forest management to fish and vertebrate biomass in forested streams. Ecosphere. 2017;8: 1–19.

52. Hawkins CP, Murphy ML, Anderson NH, Wilzbach MA. Density of fish and salamanders in relation to riparian canopy and physical habitat in streams of the Northwestern United States. Can J Fish Aquat Sci. 1983;40: 1173–1185. doi:10.1139/f83-134

53. Wootton JT. River food web response to large-scale riparian zone manipulations. PLOS ONE. 2012;7: e51839. doi:10.1371/journal.pone.0051839

54. Francoeur SN. Meta-analysis of lotic nutrient amendment experiments: detecting and quantifying subtle responses. J North Am Benthol Soc. 2001;20: 358–368. doi:10.2307/1468034

55. Ardón M, Zeglin LH, Utz RM, Cooper SD, Dodds WK, Bixby RJ, et al. Experimental nitrogen and phosphorus enrichment stimulates multiple trophic levels of algal and detrital-based food webs: a global meta-analysis from streams and rivers. Biol Rev. 2021;96: 692–715. doi:10.1111/brv.12673

56. Hansmann EW, Phinney HK. Effects of logging on periphyton in coastal streams of Oregon. Ecology. 1973;54: 194–199.

57. Warren DR, Keeton WS, Bechtold HA, Rosi-Marshall EJ. Comparing streambed light availability and canopy cover in streams with old-growth versus early-mature riparian forests in western Oregon. Aquat Sci. 2013;75: 547–558. doi:10.1007/s00027-013-0299-2

58. Harpole WS, Ngai JT, Cleland EE, Seabloom EW, Borer ET, Bracken MES, et al. Nutrient co-limitation of primary producer communities. Ecol Lett. 2011;14: 852–862. doi:10.1111/j.1461-0248.2011.01651.x

59. Greenwood JL, Rosemond AD. Periphyton response to long-term nutrient enrichment in a shaded headwater stream. Can J Fish Aquat Sci. 2005;62: 2033–2045.

60. Alila Y, Kuraś PK, Schnorbus M, Hudson R. Forests and floods: A new paradigm sheds light on age-old controversies. Water Resour Res. 2009;45. doi:10.1029/2008WR007207

61. Biggs R, Carpenter SR, Brock WA. Spurious certainty: how ignoring measurement error and environmental heterogeneity may contribute to environmental controversies. BioScience. 2009;59: 65–76. doi:10.1525/bio.2009.59.1.10

62. Jones JA, Grant GE. Peak flow responses to clear-cutting and roads in small and large basins, Western Cascades, Oregon. Water Resour Res. 1996;32: 959–974. doi:10.1029/95WR03493

63. Beschta RL, Pyles MR, Skaugset AE, Surfleet CG. Peakflow responses to forest practices in the western cascades of Oregon, USA. J Hydrol. 2000;233: 102–120. doi:10.1016/S0022-1694(00)00231-6

64. Bywater-Reyes S, Segura C, Bladon KD. Geology and geomorphology control suspended sediment yield and modulate increases following timber harvest in temperate headwater streams. J Hydrol. 2017;548: 754–769. doi:10.1016/j.jhydrol.2017.03.048

65. ODF. Oregon Forest Practices Act Water Protection Rules: Riparian Management Areas and Protection Measures for Significant Wetlands. Salem, Oregon, Oregon Department of Forestry. 2010.

66. Boisjolie BA, Santelmann MV, Flitcroft RL, Duncan SL. Legal ecotones: A comparative analysis of riparian policy protection in the Oregon Coast Range, USA. J Environ Manage. 2017;197: 206–220. doi:10.1016/j.jenvman.2017.03.075

67. ODF. Northwest Oregon State Forests Management Plan, Appendix J: Management Standards for Aquatic and Riparian Areas. 2010.

68. Johnson SL, Li JL, Sobota JB, Ashkenas LR, Pollock AM, Meleason MA, et al. Macroinvertebrate responses to differing riparian treatments following forest harvest in the headwaters of Trask River watershed. For Ecol Manag. 2022;508: 119999. doi:10.1016/j.foreco.2021.119999

69. Bladon KD, Segura C, Cook NA, Bywater-Reyes S, Reiter M. A multicatchment analysis of headwater and downstream temperature effects from contemporary forest harvesting. Hydrol Process. 2018;32: 293–304. doi:10.1002/hyp.11415

70. Arismendi I, Groom JD, Reiter M, Johnson SL, Dent L, Meleason M, et al. Suspended sediment and turbidity after road construction/improvement and forest harvest in streams of the Trask River Watershed Study, Oregon. Water Resour Res. 2017;53: 6763–6783. doi:10.1002/2016WR020198

71. Steadman CL. Natural variability of nitrogen and phosphorus in a forested headwater stream system in the oregon coast range. Masters Thesis, Oregon State University. 2017.

72. Chelgren ND, Adams MJ. Inference of timber harvest effects on survival of stream amphibians Is complicated by movement. Copeia. 2017;105: 712–725. doi:10.1643/CE-16-573

73. Benjamin JR, Dunham JB, Johnson SL, Ashkenas L, Penaluna BE, Bilby RE, et al. Pathways of productivity and influences on top consumers in forested streams. For Ecol Manag. 2022;508: 120046. doi:10.1016/j.foreco.2022.120046

74. Webster JR, Valett HM. CHAPTER 8 - Solute Dynamics. In: Hauer FR, Lamberti GA, editors. Methods in Stream Ecology (Second Edition). San Diego: Academic Press; 2007. pp. 169–185. doi:10.1016/B978-012332908-0.50010-3

75. Platts WS, Armour C, Booth GD, Bryant M, Bufford JL, Cuplin P, et al. Methods for evaluating riparian habitats with applications to management. US Dept. of Agriculture, Forest Service, Intermountain Research Station; 1987.

76. Sartory DP, Grobbelear JU. Extraction of chlorophyll a from freshwater phytoplankton for spectrophotometric analysis. Hydrobiologia. 1984;114: 177–187.

77. Parker SP, Bowden WB, Flinn MB. The effect of acid strength and postacidification reaction time on the determination of chlorophyllain ethanol extracts of aquatic periphyton. Limnol Oceanogr Methods. 2016;14: 839–852. doi:10.1002/lom3.10130

78. Lowe RL, LaLiberte GD. Chapter 11 - Benthic Stream Algae: Distribution and Structure. In: Hauer FR, Lamberti GA, editors. Methods in Stream Ecology, Volume 1 (Third Edition). Boston: Academic Press; 2017. pp. 193–221. doi:10.1016/B978-0-12-416558-8.00011-1

79. Weilhoefer CL, Pan Y. Diatom assemblages and their associations with environmental variables in Oregon Coast Range streams, USA. Hydrobiologia. 2006;561: 207–219. doi:10.1007/s10750-005-1615-1

80. Edwards PM, Pan Y, Mork L, Thorne C. Using diatoms to assess river restoration: A pilot study in Whychus Creek, Oregon, USA. River Res Appl. 2020;36: 2089–2095. doi:10.1002/rra.3712

81. Rimet F, Bouchez A. Life-forms, cell-sizes and ecological guilds of diatoms in European rivers. Knowl Manag Aquat Ecosyst. 2012 [cited 9 Dec 2022]. doi:10.1051/kmae/2012018

82. Mulholland PJ. Lotic Intersite Nitrogen eXperiment II (LINX II): a cross-site study of the effects of anthropogenic land use change on nitrate uptake and retention in 72 streams across 8 different biomes (2003 – 2006). Environmental Data Initiative; 2015. doi:10.6073/PASTA/EAC34B6C1CFB0268DD76EF68A7A90BFC

83. Massey FJ. The Kolmogorov-Smirnov test for goodness of fit. J Am Stat Assoc. 1951;46: 68–78. doi:10.1080/01621459.1951.10500769

84. DiCiccio TJ, Efron B. Bootstrap confidence intervals. Stat Sci. 1996;11: 189–228. doi:10.1214/ss/1032280214

85. Martens H, Næs T. Multivariate Calibration. John Wiley & Sons; 1992.

86. Efron B. Estimating the error rate of a prediction rule: Improvement on cross-validation. J Am Stat Assoc. 1983;78: 316–331. doi:10.2307/2288636

87. Breiman L. Statistical modeling: The two cultures (with comments and a rejoinder by the author). Stat Sci. 2001;16: 199–231. doi:10.1214/ss/1009213726

88. Box GEP, Cox DR. An analysis of transformations. J R Stat Soc Ser B. 1964;26: 211–252.

89. Wu CFJ. Jackknife, bootstrap and other resampling methods in regression analysis. Ann Stat. 1986;14: 1261–1295. doi:10.1214/aos/1176350142

90. Davidson R, Flachaire E. The wild bootstrap, tamed at last. J Econom. 2008;146: 162–169. doi:10.1016/j.jeconom.2008.08.003

91. Liu RY. Bootstrap Procedures under some Non-I.I.D. Models. Ann Stat. 1988;16: 1696–1708. doi:10.1214/aos/1176351062

92. Krämer N, Sugiyama M. The degrees of freedom of partial least squares regression. J Am Stat Assoc. 2011;106: 697–705. doi:10.1198/jasa.2011.tm10107

93. Sollins P, Cromack Jr. K, Mc Corison FM, Waring RH, Harr RD. Changes in nitrogen cycling at an old-growth Douglas-fir site after disturbance. J Environ Qual. 1981;10: 37–42. doi:10.2134/jeq1981.00472425001000010007x

94. Hesterberg T. Bootstrap. WIREs Comput Stat. 2011;3: 497–526. doi:10.1002/wics.182

95. Büning H. Kolmogorov-Smirnov- and Cramèr-Von Mises Type two-sample tests with various weight functions. Commun Stat - Simul Comput. 2001;30: 847–865. doi:10.1081/SAC-100107784

96. Büning H. Robustness and power of modified Lepage, Kolmogorov-Smirnov and Crame´r-von Mises two-sample tests. J Appl Stat. 2002;29: 907–924. doi:10.1080/02664760220136212

97. Liu RY, Singh K. Using i.i.d. bootstrap inference for general non-i.i.d. models. J Stat Plan Inference. 1995;43: 67–75. doi:10.1016/0378-3758(94)00008-J

98. Webster JR, Knoepp JD, Swank WT, Miniat CF. Evidence for a regime shift in nitrogen export from a forested watershed. Ecosystems. 2016;19: 881–895. doi:10.1007/s10021-016-9974-1

99. Deval C, Brooks ES, Gravelle JA, Link TE, Dobre M, Elliot WJ. Long-term response in nutrient load from commercial forest management operations in a mountainous watershed. For Ecol Manag. 2021;494: 119312. doi:10.1016/j.foreco.2021.119312

100. Kreutzweiser DP, Hazlett PW, Gunn JM. Logging impacts on the biogeochemistry of boreal forest soils and nutrient export to aquatic systems: A review. Environ Rev. 2008;16: 157–179. doi:10.1139/a08-006

101. Ashkenas LR, Johnson SL, Gregory SV, Tank JL, Wollheim WM. A stable isotope tracer study of nitrogen uptake and transformation in an old-growth forest stream. Ecology. 2004;85: 1725–1739. doi:10.1890/03-0032

102. Vanderbilt KL, Lajtha K, Swanson FJ. Biogeochemistry of unpolluted forested watersheds in the Oregon Cascades: temporal patterns of precipitation and stream nitrogen fluxes. Biogeochemistry. 2003;62: 87–117. doi:10.1023/A:1021171016945

103. Gomi T, Sidle RC, Richardson JS. Understanding processes and downstream linkages of headwater systems. BioScience. 2002;52. doi:10.1641/0006-3568

104. MacDonald LH, Coe D. Influence of headwater streams on downstream reachs in forest areas. For Sci. 2007;53: 148–168.

105. Sobota DJ, Johnson SL, Gregory SV, Ashkenas LR. A stable isotope tracer study of the influences of adjacent land use and riparian condition on fates of nitrate in streams. Ecosystems. 2012;15: 1–17. doi:10.1007/s10021-011-9489-8

106. Bernhardt ES, Likens GE, Buso DC, Driscoll CT. In-stream uptake dampens effects of major forest disturbance on watershed nitrogen export. Proc Natl Acad Sci. 2003;100: 10304–10308. doi:10.1073/pnas.1233676100

107. Elser JJ, Bracken MES, Cleland EE, Gruner DS, Harpole WS, Hillebrand H, et al. Global analysis of nitrogen and phosphorus limitation of primary producers in freshwater, marine and terrestrial ecosystems. Ecol Lett. 2007;10: 1135–1142. doi:10.1111/j.1461-0248.2007.01113.x

108. Francoeur SN, Smith RA, Lowe RL. Nutrient limitation of algal biomass accrual in streams: seasonal patterns and a comparison of methods. J North Am Benthol Soc. 1999;18: 242–260. doi:10.2307/1468463

109. Kiffney PM, Richardson JS, Bull JP. Establishing light as a causal mechanism structuring stream communities in response to experimental manipulation of riparian buffer width. J North Am Benthol Soc. 2004;23: 542–555. doi:10.1899/0887-3593(2004)023<0542:ELAACM>2.0.CO;2

110. Dodds WK, Smith VH, Lohman K. Nitrogen and phosphorus relationships to benthic algal biomass in temperate streams. Can J Fish Aquat Sci. 2002;59: 865–874. doi:10.1139/f02-063

111. Hill WR, Fanta SE, Roberts BJ. Quantifying phosphorus and light effects in stream algae. Limnol Oceanogr. 2009;54: 368–380. doi:10.4319/lo.2009.54.1.0368

112. Stelzer RS, Lamberti GA. Effects of N: P ratio and total nutrient concentration on stream periphyton community structure, biomass, and elemental composition. Limnol Oceanogr. 2001;46: 356–367. doi:10.4319/lo.2001.46.2.0356

113. Warren DR, Collins SM, Purvis EM, Kaylor MJ, Bechtold HA. Spatial variability in light yields colimitation of primary production by both light and nutrients in a forested stream ecosystem. Ecosystems. 2017;20: 198–210. doi:10.1007/s10021-016-0024-9

114. Naymik J, Pan Y, Ford J. Diatom assemblages as indicators of timber harvest in coastal Oregon streams. J North Am Benthol Soc. 2005;24: 569–581.

115. Biggs BJF, Stevenson RJ, Lowe RL. A habitat matrix conceptual model for stream periphyton. Fundam Appl Limnol. 1998;143: 21–56. doi:10.1127/archiv-hydrobiol/143/1998/21

116. Stevenson RJ, Pan Y, Manoylov KM, Parker CA, Larsen DP, Herlihy AT. Development of diatom indicators of ecological conditions for streams of the western US. J North Am Benthol Soc. 2008;27: 1000–1016. doi:10.1899/08-040.1

117. Bixby RJ, Benstead JP, Douglas MM, Pringle CM. Relationships of stream algal community structure to catchment deforestation in eastern Madagascar. J North Am Benthol Soc. 2009;28: 466–479. doi:10.1899/07-171.1

118. Stockner JG, Shortreed KRS. Enhancement of autotrophic production by nutrient addition in a coastal rainforest stream on vancouver island. J Fish Res Board Can. 1978;35: 28–34. doi:10.1139/f78-004

119. McIntyre AP, Hayes MP, Ehinger WJ, Estrella SM, Schuett-Hames DE, Quinn, Timothy. (Technical Coordinators). Effectiveness of Experimental Riparian Buffers on Perennial Non-fish-bearing Streams on Competent Lithologies in Western Washington. Cooperative Monitoring, Evaluation and Research Report CMER 18-100, Washington State Forest Practices Adaptive Management Program, Washington Department of Natural Resources, Olympia, WA.; 2018.

120. Bennett MG, Lee SS, Schofield KA, Ridley CE, Washington BJ, Gibbs DA. Response of chlorophyll a to total nitrogen and total phosphorus concentrations in lotic ecosystems: a systematic review. Environ Evid. 2021;10: 1–25. doi:10.1186/s13750-021-00238-8

121. Biggs BJF. Eutrophication of streams and rivers: dissolved nutrient-chlorophyll relationships for benthic algae. J North Am Benthol Soc. 2000;19: 17–31. doi:10.2307/1468279

122. Murphy ML. Forestry Impacts on Freshwater Habitat of Anadromous Salmonids in the Pacific Northwest and Alaska: requirements for protection and restoration. NOAA/ National Centers for Coastal Ocean Science; 1995. Available: Forestry Impacts on Freshwater Habitat of Anadromous Salmonids in the Pacific Northwest and Alaska: Requirements for Protection and Restoration

123. Groom JD, Johnson SL, Seeds JD, Ice GG. Evaluating links between forest harvest and stream temperature threshold exceedances: the value of spatial and temporal data. JAWRA J Am Water Resour Assoc. 2017;53: 761–773. doi:10.1111/1752-1688.12529

Supporting Information

S1 Table. Details of PLS, Wu jacknife, Wild Bootstrap models for headwater treatment groups. Box-Cox transformations were used with the goal of improving model quality. See S1 File for details on Box-Cox transformations. An exponent of 1 indicates that raw data were used. For normality tests, bold indicates that the null hypothesis of normal residuals was rejected. For Levene's test, bold indicates that the null hypothesis of a common variance across groups of a given factor was rejected. Significance was not reported in Table 2 when R^2CV was negative for PLS. For Wu Jackknife and Wild Bootstrap, bold indicates regression coefficients were significantly different than zero. We did not assume the regressor rows were independent and consequently used the Wild Bootstrap and Wu Jackknife for discerning model parameter significance. Interaction predictions are PLS model predictions transformed back to the original scale of the data. Dissolved organic carbon (DOC), Soluble reactive phosphorus (SRP), Dissolved organic nitrogen (DON), Dissolved inorganic nitrogen (DIN), Chlorophyll a (Chl a).

S2 Table. Details of PLS, Wu jackknife and Wild Bootstrap models for headwater watersheds. Box-Cox transformations were used with the goal of improving model quality. See S1 File for details on Box-Cox transformations. An exponent of 1 indicates that raw data were used. For normality tests, bold indicates that the null hypothesis of normal residuals was rejected. For Levene's test, bold indicates that the null hypothesis of a common variance across groups of a given factor was rejected. For Wu Jackknife and Wild Bootstrap, bold indicates regression coefficients were significantly different than zero. We did not assume the regressor rows were independent and consequently used the Wild Bootstrap and Wu Jackknife for discerning model parameter significance. Interaction predictions are PLS model predictions transformed back to the original scale of the data. Dissolved organic carbon (DOC), Soluble reactive phosphorus (SRP), Dissolved organic nitrogen (DON), Dissolved inorganic nitrogen (DIN), Chlorophyll a (Chl a).

S3 Table. Statistical summaries and details of PLS, Wu jackknife and Wild Bootstrap models for downstream sites. A) Kolmogorov-Smirnov two sample test p-values compare post- and pre- empirical cumulative distribution functions for downstream site groups (downstream of harvest and downstream of reference). **B)** Comparison of 95% Bias Corrected accelerated (BCa) Bootstrap confidence intervals (CI) for observed pre- and post-harvest treatment means for each response variable. "0" indicates the pre- and post-harvest CI overlapped; "Increase" indicates that the lower bound of the post-harvest CI was greater than the upper bound of the pre-harvest CI. "decrease" where pre-harvest was greater than post-harvest. **C)** Pre/Post comparisons of significance for PLS model predictions for each attribute, based on 95% Wild Bootstrap confidence intervals. When R^2CV was negative, significance was not reported, indicated by "- -" . Within a group, pre/post with the same letters are not significantly different: "A" denotes the largest value, "B" is second largest, etc, for that attribute. Box-Cox transformations were used

with the goal of improving model quality. For Wu Jackknife and Wild Bootstrap, bold indicates regression coefficients were significantly different than zero. We did not assume the regressor rows were independent and consequently used the Wild Bootstrap and Wu Jackknife for discerning model parameter significance. Dissolved organic carbon (DOC), Soluble reactive phosphorus (SRP), Dissolved organic nitrogen (DON), Dissolved inorganic nitrogen (DIN), Chlorophyll a (Chl a).

S4 Table. Concentrations of A) stream nitrate (NO^3) and B) ammonium (NH_4) at each site, each year at the time of early summer sampling. "--" indicates no sample collected. Additional Trask River Watershed study chemistry data are available through US Forest Service Research Data Archive: https://doi.org/10.2737/RDS-2022-0002

S1 File. Details of Box-Cox transformations.

S2 File. Epilithon biomass and chlorophyll *a* concentrations in June and July 2016.

Figure 1

Click here to access/download;Figure;Fig1.tif

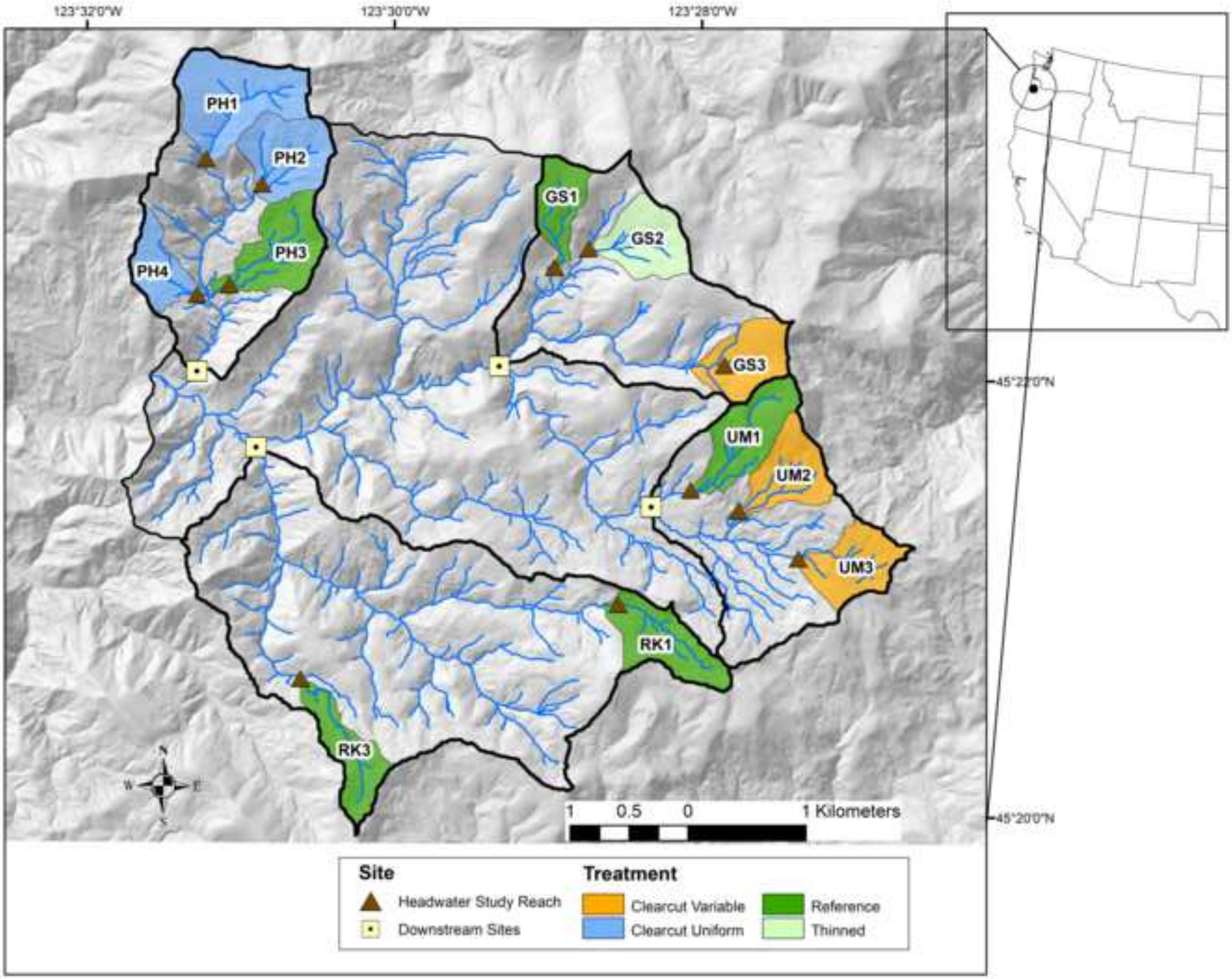

Click here to access/download;Figure;Fig2.tif

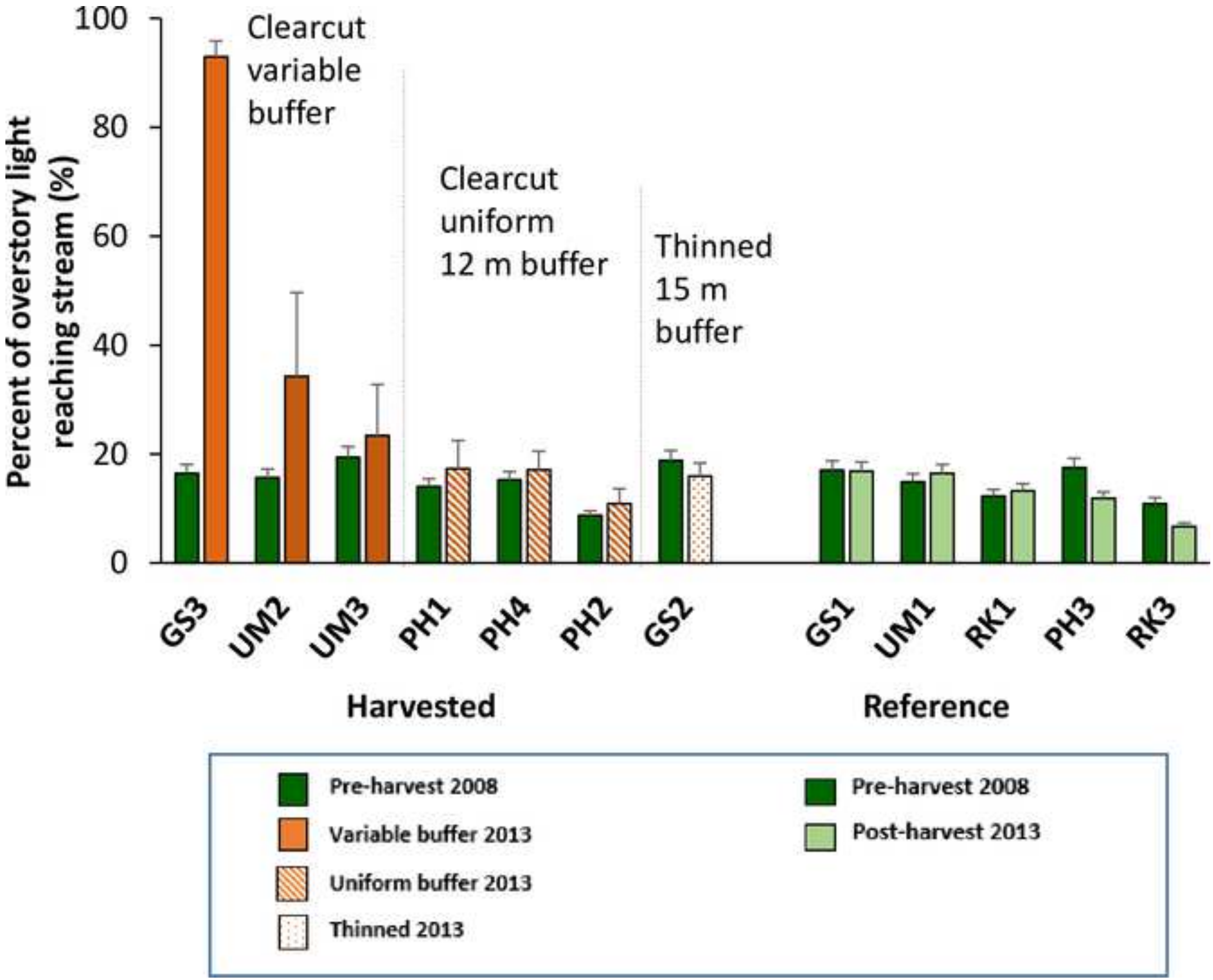

Click here to access/download;Figure;Fig3.tif

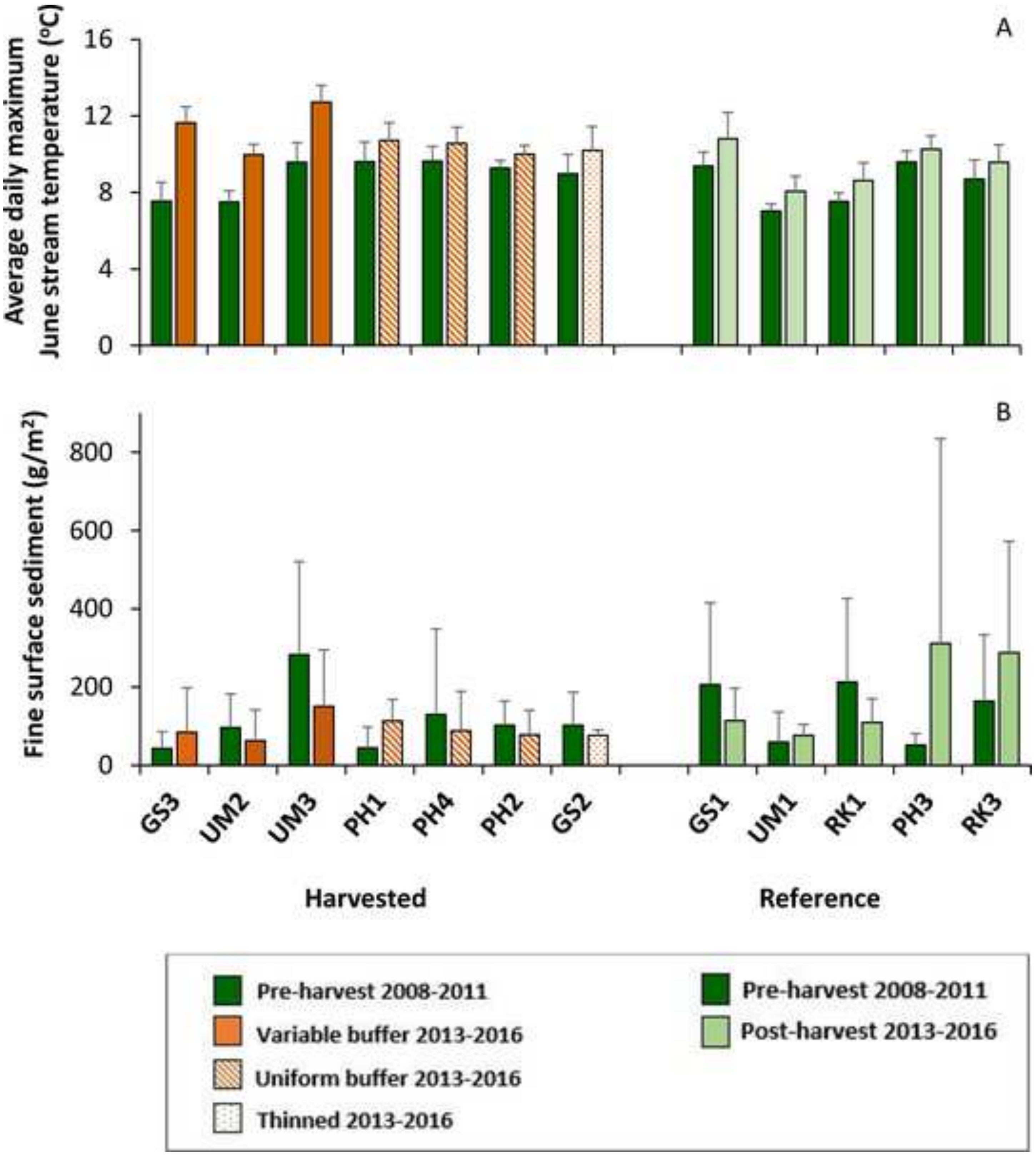

Click here to access/download;Figure;Fig4.tif

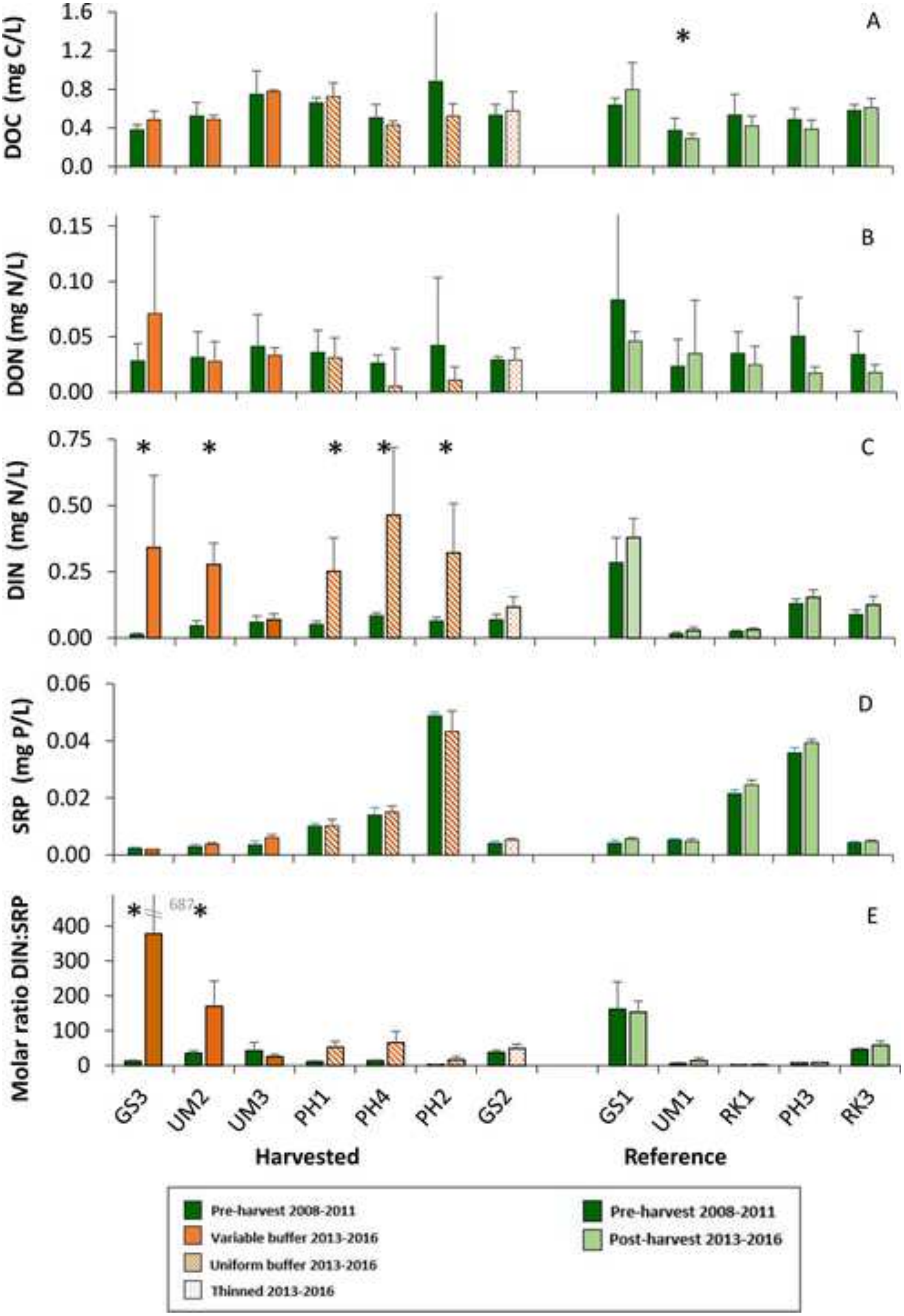

Click here to access/download;Figure;Fig5.tif

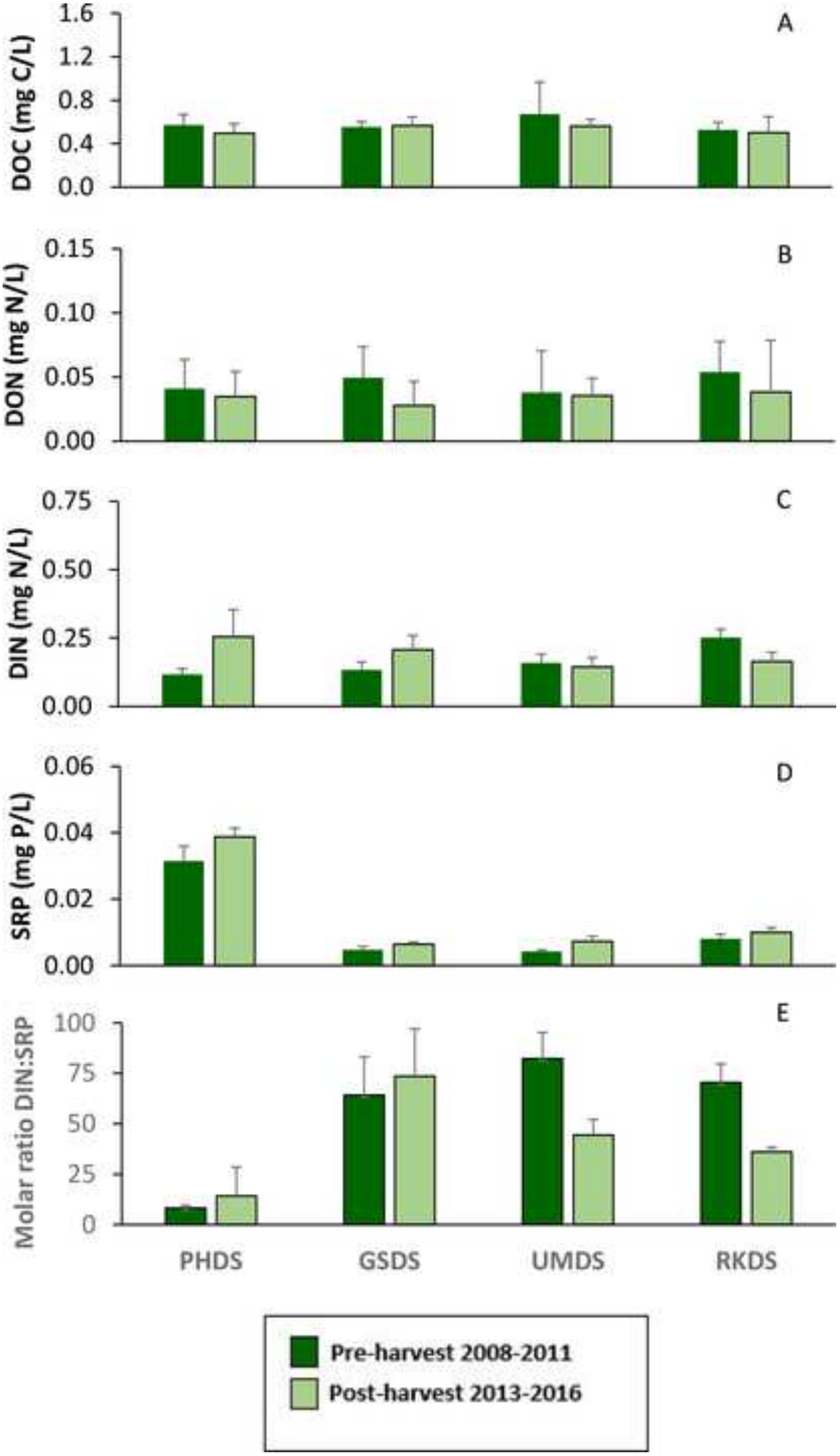

Click here to access/download;Figure;Fig6.tif

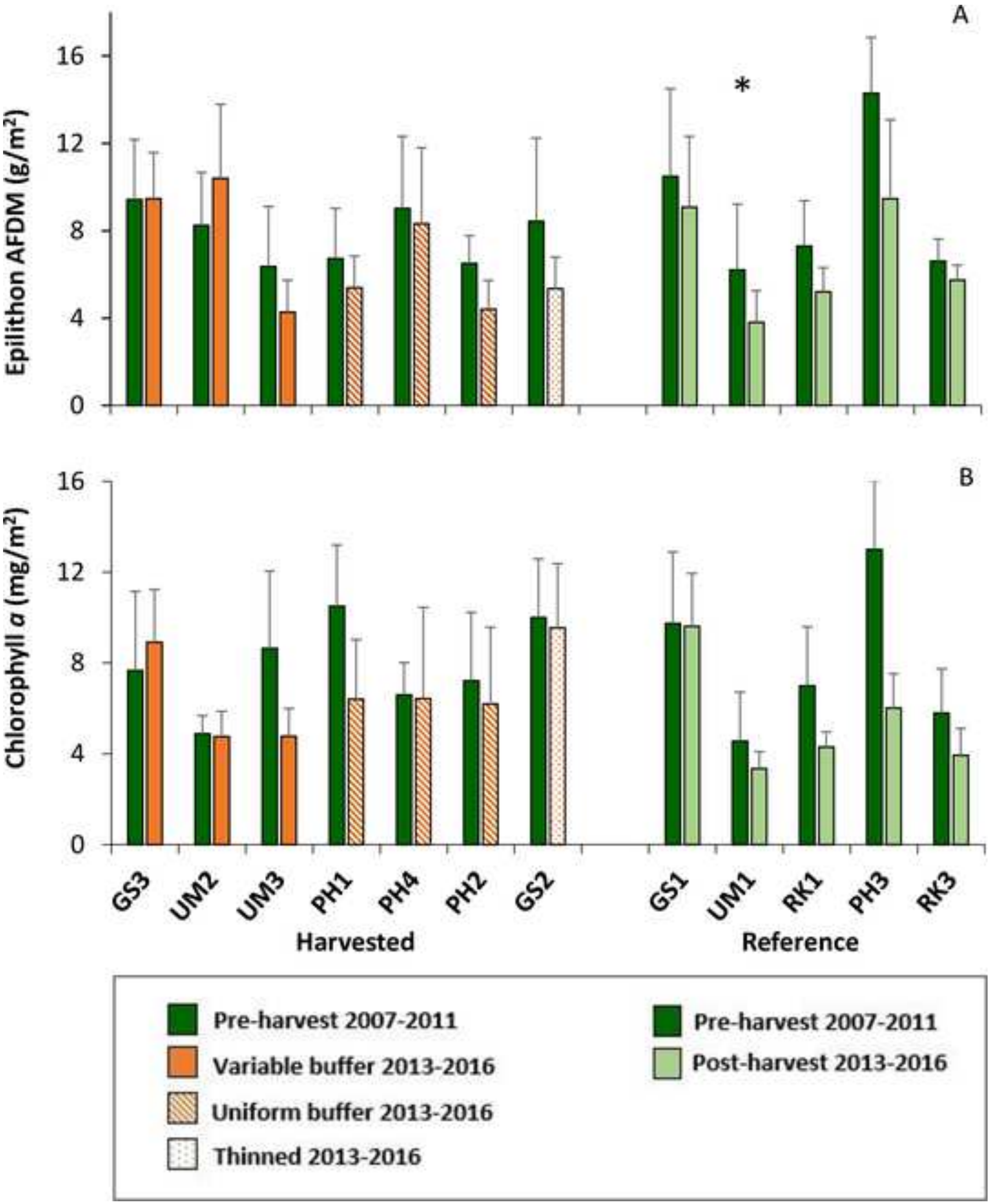

Click here to access/download;Figure;Fig7.tif

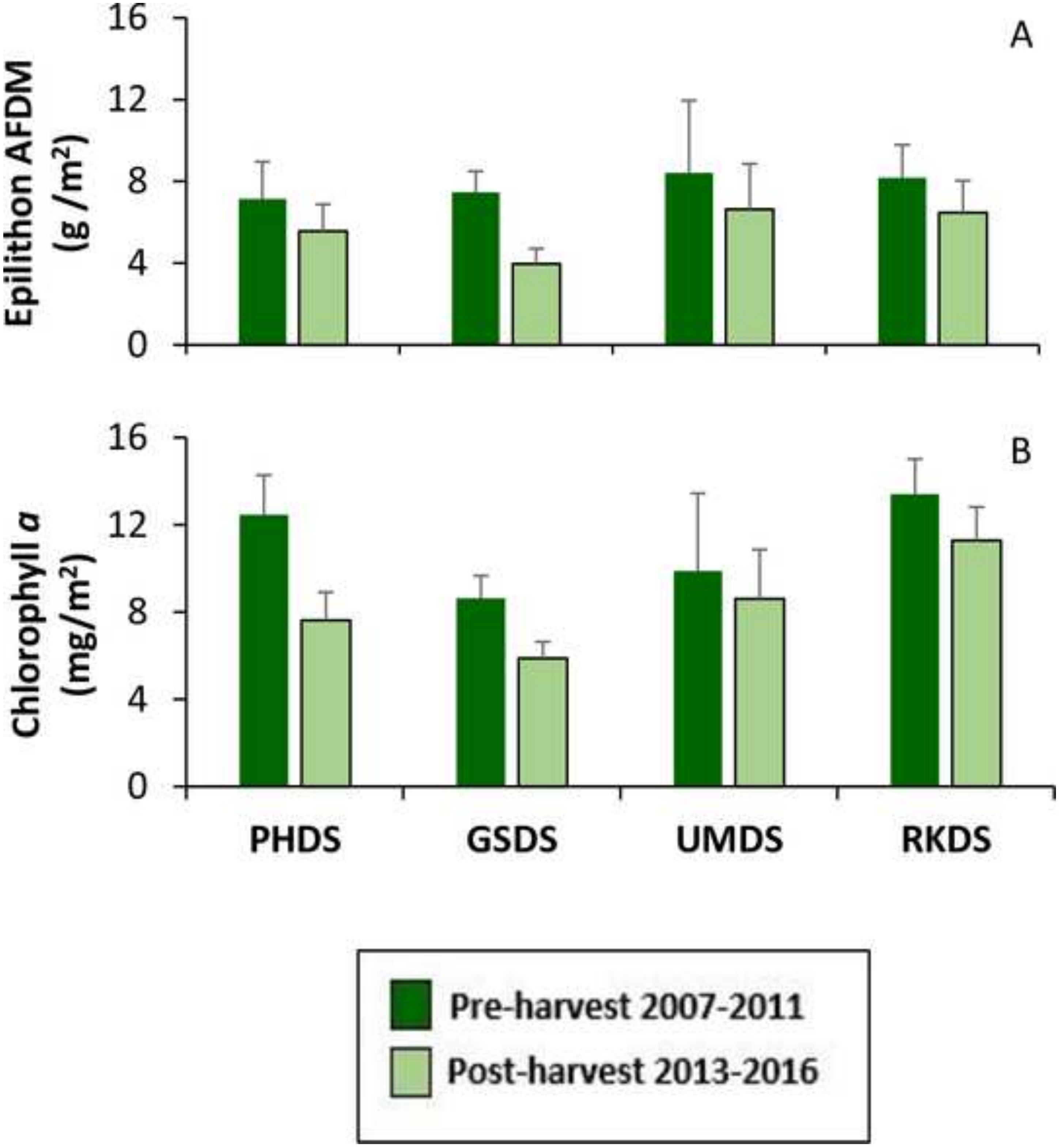

Click here to access/download;Figure;Fig8.tif

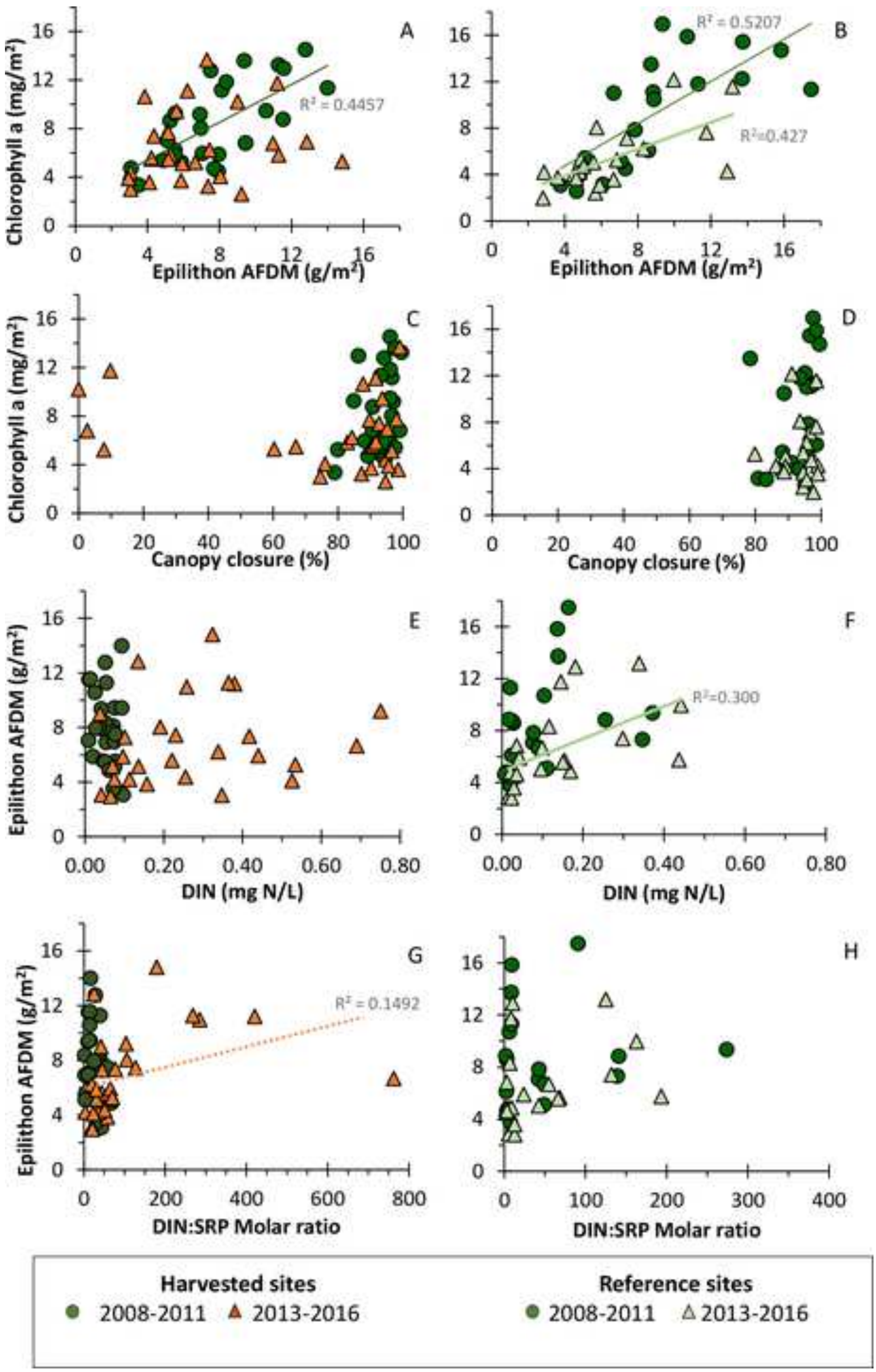

Click here to access/download
Supporting Information
S1_File.docx

Click here to access/download
Supporting Information
S2_File.docx

Click here to access/download
Supporting Information
S1_Table.docx

Click here to access/download
Supporting Information
S4_Table.docx

Click here to access/download
Supporting Information
S2_Table.docx

Click here to access/download
Supporting Information
S3_Table.docx

~~The paradox of~~S ~~s~~tream nitrate enrichment and increased light yet no algal response following experimental manipulation of headwater riparian zones and forested watersheds

Sherri L. Johnson[1*], Alba Argerich[2], Linda R. Ashkenas[3], Rebecca J. Bixby[4], David C. Plaehn[5]

[1] Pacific Northwest Research Station, USDA Forest Service Corvallis, Oregon, United States of America

[2] School of Natural Resources, University of Missouri, Columbia, Missouri, United States of America

[3] Department of Fisheries and Wildlife, Oregon State University, Corvallis, Oregon, United States of America

[4] Department of Biology, University of New Mexico, Albuquerque, New Mexico, United States of America

[5] Pilot Records, Corvallis, Oregon, United States of America

*Corresponding author

E-mail: sherri.johnson2@usda.gov

Keywords

Stream chemistry, algae, epilithon, forest harvest, disturbance, riparian, buffers~~,~~ ~~stoichiometry~~

Abstract

~~Forest d~~Disturbances to forested watersheds often result in~~have been shown to~~ increases ~~a release~~ of nutrients ~~to the streams draining the landscape and in an increase of in-stream~~ and light to nearby streams. Such~~availability. Tose~~ changes are generally expected to produce a shift to a more autotrophic aquatic ecosystem, with~~with~~ measurable increases in ~~primary producers'~~algae ~~biomass~~, ~~and activity, with~~ and associated implications for food webs and fisheries. Although this paradigm is widely established~~accepted~~, ~~-~~results from our 10-year study (2007-2016) in 12 headwater streams and four sites downstream ~~sites~~ in~~at~~ the Trask River Watershed (Oregon, USA), did~~o~~ not ~~support it~~concur. In 2012, one watershed was thinned, three~~six~~ ~~of the headwater watersheds~~ were clearcut harvested with variable buffers and three with uniform ~~two types of~~ riparian buffers. After harvest, light to the stream surface significantly increased at the three watersheds with variable buffers while ~~-~~dissolved inorganic nitrogen (DIN) significantly increased ~~up to 5 times~~ in all of the clearcut harvested streams~~, whether or not forested riparian buffers were retained~~. Despite the increase in DIN and light, standing stocks of epilithon and concentration of chlorophyll *a* did not significantly increase. ~~-~~While this dynamic has been well documented in many systems, there are complex interactions that can lead to differing responses. We postulate ~~that~~ the ~~the stoichiometry~~co-limitation of nutrients, driven by low phosphorus concentrations, which unlike DIN did not change~~ before and after harvest~~, plus the

characteristics of the algal community, which were~~as~~ dominated by low light adapted diatoms rather than green algae, ~~resulted~~ contributed to our findings of ~~in little~~ no responses for standing stocks of epilithic algae~~on~~ or chlorophyll *a* ~~concentration~~. The ~~The~~ common assumption~~-~~ of increased autotrophic responses in stream food webs following increases of nitrogen and light was not supported here. The inclusion of multiple statistical analyses provided more certainty around our findings~~allowed us to explore the variability in responses and to examine the concurrence of differing analytical tests~~. This study documents responses to current forest practices and provides~~provided more certainty around our findings and better~~ cautionary information for ~~managers and policy makers in their planning and evaluations of responses to management activities.~~management and restoration activities aiming to increase abundances and standing stocks of fish by opening riparian canopies and adding nutrients.

Introduction

Terrestrial disturbances in headwaters and the role of riparian forests in maintaining stream integrity

Researchers have long worked to understand instream responses associated with differing types of watershed disturbances, including forest harvest [1,2], fire [3,4], hurricanes [5], agriculture [6], and urbanization [7]. Forests and woodlands cover large portions of landscapes; across the Unites States, they comprise one-third of the land base [8]. Forested watersheds managed for timber production (including harvest) some 67% of the forested lands in the USA [8]. ~~E~~Evaluation of the effects of forest harvest on streams ecosystems is therefore ~~has been a~~an

important and necessary ~~frequent~~ focus of ~~study~~ research and monitoring [9]. ~~because many forested watersheds are managed for timber. The s~~Streams ~~that~~ flowing through forests provide habitat for a diverse array of aquatic organisms [10,11] as well as serve as sources of drinking water for downstream communities [12]. Because headwater streams represent 80-90% of miles of any give~~the~~n river network [13], reducing impacts from forest harvest to headwater streams can have broad implications ~~not only~~ locally as well as~~but~~ downstream [11,14].

Forested riparian zones~~, especially on small streams,~~ provide a variety of ~~ecosystem services~~functions [15,16] including buffering aquatic ecosystems from disturbances within their watersheds [9,17]~~[9,17]~~. Intact riparian vegetation can stabilize stream banks [18], provide shade ~~headwater streams~~ to ~~minimize increases~~maintain cool ~~in~~ water and air ~~stream~~ temperatures [19,20], ~~or~~ minimize direct solar radiation that can potentially stimulate algal blooms [2,21], intercept nutrients and sediment transported from hillslopes [22,23], and deliver allochthonous detrital material, including wood, leaves, and needles [24,25]. Changes to riparian vegetation and/or the widths of riparian buffers during natural or anthropogenic disturbances can result in shifts in some~~any~~ or all of these functions, ~~and ecosystem services~~ potentially leading to impacts on ~~can influence~~multiple trophic levels of instream biotic communities [26–29] and water quality for downstream water users ~~through changes in water quality~~ [9,30].

Formatted: Font: (Default) +Body (Calibri), 11 pt

Management of riparian forests to reduce instream responses to harvest ~~disturbances~~

The assumptions and observations of instream responses to riparian management are numerous and sometimes conflicting, which can make it challenging to identify ~~Establishment of~~ best management practices and regulatory guidelines during forest harvest. ~~can be complex because~~

~~assumptions and observations of the responses of the lower trophic levels of food webs to forest management and riparian disturbances are numerous and sometimes conflicting.~~ Identifi~~i~~ying the ~~cation of the~~ optimal widths of ~~for~~ riparian buffers necessary for ~~a~~ protection of instream functions ~~functional riparian zone~~ is complicated by multiple site-specific factors ~~that~~ capable of influencing~~e~~ riparian-stream interactions [23,31] as well as which stream response parameter or process is of most interest [17,32,33]. For example, h~~H~~eadwater forested streams frequently have low background levels of ~~nutrients~~ stream nutrients [34,35] and following harvest ~~and fire related disturbances~~in forested watersheds, increases in dissolved nitrogen, phosphorus and carbon ~~nutrients~~ have been observed [36–40]. Increases in stream chemistry concentrations have been associated with increased instream sediment [29,39,40], ~~[40]~~especially when riparian buffers were not retained during harvest. However, not all studies have observed increased stream nutrients or stream sediment following forest harvest [35,41,42]. ~~[4]~~~~However, not all studies have observed increased stream nutrients following forest disturbances [35,41,42].~~ ~~Maintenance~~ Retention of vegetated riparian buffers during harvest ha~~ve~~s been suggested as a way to mitigate these increases [16,43,44]. ~~However, not all studies have observed increased stream nutrients following forest disturbances [35,42,43].~~

The removal of riparian vegetation during harvest has also been frequently associated with increases in stream algae [45,46]. Riparian harvest, and the accompanying changes in instream light and possibly nutrients [45,47,48]~~,~~ can result in ~~Likewise,~~ higher biomass of attached algae and increased concentrations of chlorophyll *a* [49,50]. In studies of forest streams, an increase in algae has been linked to higher densities of macroinvertebrates and fish [47,49,51–53]. Depending on site specific interactions, increases in nutrients and potential co-limitation between nitrogen and phosphorus can influence algal biomass [54,55]~~.~~. T~~In addition,~~

twhether through removal of riparian vegetation [39,40] or natural gaps in canopy cover [41]. The amount of light reaching the stream affehe taxonomic composition and form of algalepilithic assemblages can shift in response to changes in is influenced by light and nutrients; s and several studies have documented a shift from diatom communities to filamentous green algae following clearcutting (e.g., [45,46,56]). [58]. surface, edassociated with higher instream productivity from [46,48,55 57]Although the relationships among reduced riparian cover, increased light and increased algal standing stocks are frequently assumed to be ubiquitous [21,57], Although other a subset of researcher paperss have noted that algal responses to increased light and nutrients can be negligible [46,58,59]., depending on site specific interactions, including the stochiometry between nitrogen and phosphorus ( ). The relationship between riparian cover and increased light and increased algal standing stocks are frequently assumed to be ubiquitous [21,61] ( ). It has been suggested that nNull findings that do not support this common paradigm [61] are less frequently published [58]; this would influence the information available for new studies as well as support for assumptions associated with instream responses to forest management.

Compounding the complexity of instream responses to forest harvest is the inflexibility of commonly used statistical tests to capture biological variability and statistical significance in dynamic systems such as streams.focus given by both resource managers and researchers to the importance of statistically significant findings. Traditional statistical analyses have generally been developed for controlled studies with high replication and constant variance. The statistical assumptions for parametric analyses of normally distributed, homoscedastic data, randomly assigned treatments and independent measurements may not be appropriate for field supported in studies of using field data and landscape-scale experiments with low sample sizes. Yet the sOn tatistical significance associated with natural resource issues questions is highly relevant to forest

planning and policy, which has led to multiple instances of controversies about the best ~~nd~~ statistics ~~dueling statistics are not uncommon~~ [60–63]. Given the~~is~~ potential complexity ~~of responses~~of types of statistical assessments, research that informs best management practices, including management of riparian vegetation during harvest, would ideally address these multifaceted dynamics with multiple, ~~requires our best understanding of~~rigorous evaluations ~~multifaceted dynamics in conjunction with multiple evaluations~~ of responses to current forest harvest practices.

In this landscape-scale experimental study, we use multiple statistical tests to examine whether differences in the ~~extent~~ width of riparian buffers retained during clearcut harvest preserve water quality ~~, as measured by nutrient concentration,~~ and whether stream ecosystems shift to include more autochthonous resources~~, measured as standing stock of epilithic algae and chlorophyll *a*~~. Our hypotheses were~~were~~ that ~~more~~ removal of some or all riparian vegetation would lead to a) higher concentrations of instream nutrients and dissolved organic carbon, b) higher chlorophyll *a* concentration and algal biomass in response to more available light and nutrients~~ to the streams~~, ~~which would result in higher chlorophyll *a* concentration and epilithon biomass, , which would result in higher concentrations of instream nutrients and dissolved organic carbon~~and c) increased instream sediment as a result of disturbances to hillslope soils from harvest activities~~, which would result in increased instream sediment~~. We also hypothesized that responses within a harvested area might propagate to downstream sites.

~~We specifically ask:~~

- ~~Following watershed disturbance from forest harvest, do instream carbon and nutrient concentrations increase? Does the presence of forested riparian buffers mitigate increases in stream chemistry concentrations?~~

- ~~What is the temporal and spatial context of nutrient responses? If there are changes, are they consistent across years and do they propagate downstream?~~
- ~~Do algal standing stocks and chlorophyll concentrations respond to potential changes in nutrients and increases in light reaching streams after harvest?~~

Methods

Site description and experimental design

Study sites were located in the Trask River Watershed Study area, within the upper 25 km^2 of the South Fork of the Trask River in Oregon, USA (Fig 1). The Trask River originates in the northern Oregon Coast Range and flows westward into the Pacific Ocean. The underlying geology is a mixture of sedimentary deposits and volcanic basalt intrusions [64]. Annual rainfall in the upper Trask study area averages 200 cm per year, primarily between October and May, with occasional snow at higher elevation sites. At the beginning of this study, the hillslope and riparian forests in the Trask River Watershed Study area were 45-60 year old Douglas-fir (*Pseudotsuga menziesii*) and red alder (*Alnus rubra*). Big leaf maple (*Acer macrophyllum*) occurred only in riparian areas. This landscape had been heavily impacted by prior disturbances; a series of wildfires occurred between 1933 and 1951 and following the fires, all burned and unburned trees were harvested and the area replanted. In addition to impacts from fires and harvest, land management legacies included a high density of logging roads, minimal downed wood in streams and riparian areas, and a simplified understory plant community.

The Trask River Watershed Study was conducted by researchers and natural resource managers as a multi-disciplinary, 10+ yr. collaborative study to understand and quantify the onsite effects of current timber harvest practices on physical, chemical, and biological

communities in first-order headwater streams. We also evaluated whether responses to headwater harvest propagated downstream to third-order fish-bearing streams. This study was conducted using a paired~~, nested~~ study design of 12 ~~treatment and reference~~ small watersheds nested within four large catchments (Fig 1). Within each large catchment, two to three small headwater watersheds (17-67 ha) were clearcut harvested over as much of the watershed area as possible, while one headwater watershed remained as an unharvested reference site (Table 1). In addition, no harvests occurred in one of the larger catchments (Rock Creek) so that it could serve as a reference site for the downstream comparisons. The pre-harvest sampling occurred 2007-2011. In 2012, harvest took place in seven headwater watersheds and no sampling occurred. Post-harvest, sampling restarted in 2013 and continued through 2016.

Fig 1. Study watersheds in Trask River Watershed Study, western Oregon. A) Reference watersheds are shown in dark green, clearcut with variable buffers are shown as orange and clearcut with uniform buffers are show as blue. Light green watershed was thinned with wide unharvested buffers. Headwater sampling locations are shown as triangles and downstream sites are noted as squares. B) Imagery from 2013, the first year post-harvest, with outlines of the study watersheds. (Reprinted from Johnson et al. 2022 [71] under a CC BY license with permission of Elsevier, 2022) ~~shown.~~

Table 1. Study site characteristics.

Catchment	Watershed	Harvest treatment [1]	Ownership	Elevation Study Site (m)	WS Area (ha)	Percent WS harvested in 2012	Distance to downstream site (m)	Ave channel slope (%)	Ave wetted channel width (m)	Ave water depth (cm)	Ave discharge (L/s)[2]
**Upper Main**	UM 1	Reference	Private	658	44.2	0%	406	18.7	1.47	6.8	6.6
	UM 2	Clearcut variable buffer	Private	685	17.1	91%	1140	18.4	1.16	5.8	5.9
	UM 3	Clearcut variable buffer	Private	728	39.2	56%*	1581	11.8	1.34	6.9	5.2
	UM Downstream	Downstream of harvested	Private	608	278.8	25%	--	3.8	3.63	14.1	15.1
**Gus**	GS 1	Reference	Private	611	26.5	0%	1059	12.1	1.32	6.1	3.7
	GS 2	Thinned	Federal	638	39.0	91%	1355	14.5	1.82	7.0	5.6
	GS 3	Clearcut variable buffer	Private	789	20.4	92%	2297	22.4	1.20	4.2	1.2
	GS Downstream	Downstream of harvested	State	470	301.4	30%	--	10.2	4.16	15.4	27.6
**Pothole**	PH 1	Clearcut uniform buffer	State	531	67.1	87%	2352	13.6	2.00	10.1	-
	PH 2	Clearcut uniform buffer	State	493	20.4	82%	2159	13.7	1.00	6.2	4.9
	PH 3	Reference	State	390	45.4	0%	1044	12.8	0.93	5.5	4.3
	PH 4	Clearcut uniform buffer	State	370	23.6	92%	947	13.8	0.80	5.2	1.5
	PH Downstream	Downstream of harvested	State	324	324.6	44%	--	2.8	3.30	13.0	19.4
**Rock**	RK 1	Reference	Private	680	42.2	0%	4385	7.6	1.36	9.5	11.7
	RK 3	Reference	State	548	35.3	0%	2388	16.2	1.66	9.9	12.3
	RK Downstream	Downstream reference	State	334	669.9	0%	--	3.5	4.58	16.3	43.1

[1] **Harvest details:**

Clearcut variable buffer: A riparian buffer was not required on these sites. During harvest, leave trees remained in the downstream riparian areas of UM2 and UM3

Clearcut uniform buffer: Clearcut or modified clearcut with a uniform 12 m riparian buffers on each side of the stream

Thinned: Thinned watershed with a 15 m no-harvest riparian buffer on each side of the stream

Reference: Not harvested during this study period. Vegetation age was 45-60 yrs.

[2] Discharge was measured with conservative salt tracers at the same time as the water chemistry and epilithon sampling.

* An additional portion of this WS had been harvested 6 years previously

Riparian treatments during forest harvest differed by land ownership (Fig 1; Table 1). On private forest lands, riparian buffers were not required at the time of this study [65,66] and the watersheds owned by forest industry (UM3, UM2, and GS3), were clearcut with variable amounts of riparian vegetation retained. Forest harvest guidelines for protection of terrestrial wildlife require the equivalent of two live trees per acre not be harvested [65]. In two watersheds (UM2 and UM3)~~)~~, the ~~“leave~~ trees designated for wildlife~~”~~ were grouped in the riparian areas for operational reasons including to avoid steep banks or wet soils. We termed this treatment group as “Clearcut variable”.

During harvest on State Forest lands (PH1, PH2, PH4), the ODF Forest Management Plan required minimum of 7.6 m buffers along each side of the perennial section of the stream channel [67]. In addition,~~However,~~ some of the required trees designated for wildlife~~“leave trees”~~ were grouped adjacent to the buffers which led to final buffers of approximately 12 m [19,68]. We characterized these sites as clearcut with uniform width buffers, and termed this treatment group as “Clearcut uniform”.

The thinned ~~One~~ watershed (GS2) was on federal land, managed by the Bureau of Land Management. Harvest practices involved thinning throughout the watershed using helicopter logging and retaining a 15 m, no-entry buffer on each side of the stream. This site is termed thinned with uniform buffer and abbreviated as “Thinned”. No additional forest treatments beyond these described occurred during the study period. More information is available as supplementary material with the archived data ~~[57]~~.

~~Study reaches of 30 m length were established in twelve small headwater watersheds (Table 1).~~ The locations of the study reaches were designed to ~~overlap~~ spatially coincide with collaborative studies, which ~~that~~ included stream temperature [19,69], suspended sediment

[64,70], stream chemistry during storms [71], stream discharge, macroinvertebrates [68], amphibians [72] and fish [73]. In the harvested watersheds, the study reaches were located within ~~at~~ the downstream most portion of the treated area. In addition, ~~order~~ to assess potential cumulative impacts of harvest in upstream headwaters, four downstream study reaches ~~of 100 m~~ were located in larger third-order fish-bearing streams, one in each of the catchments (Fig 1). Three study reaches were downstream of harvested watersheds, at distances of 947-2297 m (Table 1): UMDS was downstream of sites clearcut with variable buffers, PHDS was downstream of sites clearcut with uniform buffers and GSDS had mixture of harvest groups upstream. Rock Creek, RKDS, was the reference for the downstream sites.

Each year, the sampling for the research occurred in early summer to provide as coincident measurements of concentration, biomass, or abundance as possible for all of the study groups. In early summer, streamflow was sufficient at all headwater sites for consistent biotic sampling to occur. Sampling schedules were established to prevent unintended impacts of each type of sampling on subsequent projects. The Trask headwater sites did not contain fish, but all were inhabited by an amphibian predator, Coastal Giant salamander (*Dicamptodon tenebrosus*), and a subset of the sites contained an amphibian grazer, Coastal Tailed Frog tadpoles (*Ascaphus truei)*. At downstream sites, resident fish species included Cutthroat Trout (*Onchorhynchus clarkia clarkii*) and Sculpin (*Cottus* spp.). Two of the downstream sites also contained anadromous Coho Salmon (*Oncorhynchus kisutch*) and Steelhead (*Onchorhynchus mykiss*) on a seasonal basis.

Data collection

Stream characterization

Sampling occurred annually during the last two weeks of June between 2008-2011 (pre-harvest period) and 2013-2106 (post-harvest period). On each sampling date, at each of the 12 headwaters and 4 downstream sampling sites, we collected basic measurements of stream physical characteristics including wetted stream width, water depth, discharge, water temperature, fine benthic sediment, and canopy closure. Channel dimensions were measured at a series of 4-transects spaced 10 meters apart over a 30 min each reach in headwaters and at 10 transects over 100 m reach at downstream sites. Discharge at the time of during sampling was calculated using a constant addition of a conservative salt tracer [74]. We avoided sampling during rain events. Water temperatures were measured continuously from June-September using recording thermistors (Onset Computer Corporation, model U22-001) protected from direct radiation [19]. Reach slope was measured in the field using a stadia rod and clinometer (PM-5, Suunto) in 2008 and 2013. At each sampling time, the aAverage percentage of canopy closure was measured at each transect from the center of the stream using a handheld concave densiometer [75] at transects. In addition, in 2008 (pre-harvest) and 2013 (immediately after harvest), we quantified the amount of overstory light reaching the stream using hemispherical photos., in addition to the annual measurements of canopy closure. We also conducted more intensive channel and riparian measurements within the 30 m benthic sampling reach and over an additional 70 m upstream at headwater sites. Downstream channel and riparian measurements covered 200 m reach. The hHemispherical photos were recorded using a self-leveling camera with hemispherical lens that was positioned mid-channel and 1m above the water surface. A total of five photos were taken per reach withP photos were taken everyspaced 25 m apart in

headwater channels and ~~every~~ 50 m apart at downstream sites ~~for a total of five photos per reach~~. Photos were taken at dawn or dusk and when there were no cloudes. In 2008, the position of ~~each~~ camera point~~osition~~ was carefully documented so that post-harvest images could be collected from the same position and height. The percent of overstory light ~~Incoming radiation~~ reaching the stream ~~(percent total site factor)~~ was calculated for each image using a digital image analyzer (Metric used: percent total site factor; WinSCANOPY, Regent Instruments, Inc.) and ~~;~~ the site average calculated ~~and termed percent of full light reaching the stream~~. ~~Reach slope was measured in the field using a stadia rod and clinometer (PM-5, Suunto) in 2008 and 2013.~~

Water chemistry

At each headwater and downstream site during the annual June sampling, we collected a grab sample of stream water for analyses of water chemistry. The samples were stored on ice in the field, and then filtered through pre-combusted Whatman GF/F filters (pore size = 0.7μm) in the laboratory within 3 days of collection. After filtering, the samples were frozen for later analysis at the Oregon State University Cooperative Chemical Analytical Laboratory (see http://ccal.oregonstate.edu/methodology for description of analytical methods and detection limits). Analytes reported here include dissolved organic carbon (DOC), soluble phosphorus (SRP), dissolved inorganic nitrogen (DIN; sum of nitrate, nitrite, and ammonium), dissolved organic nitrogen (DON; calculated as the difference between total dissolved nitrogen and DIN), total dissolved nitrogen (TDN) and total dissolved phosphorus (TDP).

Algae

Each year in late June, fFour composite samples of epilithic algae, hereafter termed epilithon, were collected at each of the 12 headwater sites and s and six composite samples were collected at each of at the four downstream sites ineach June. Epilithon is a complex matrix of algal cells on stream substrates; it potentially includes bacteria, fungi, and fine detrital matter. The epilithon samples were for the analyzeds for of photosynthetic pigments (chlorophyll *a* concentration) and algal standing stock (biomass).s. For each composite sample, we used a small wire brush to scrubbed a known areas of randomly selected rocks from riffles, glides, and cascades, but not pools. Each composite contained material scrubbed from four to twelve (4-12 rocks, with thae fortotal sample area of at least 50 cm^2) within a 30 m reach. Each rock was gently lifted out of the water and aA flexible plastic template with a known area was held onto eachthe rock, exposing only the area to be scrubbed. After scrubbing, the loosened epilithic material within the template area was gently rinsed into the sample container. The total area scrubbed from multiple rocks was recorded for each composite sample. , for a total of area of at least 50 cm^2, and collected the slurry in a container. Sample containers were placed on dry ice in the field, frozen, and analyzed within one month of collection. In 2016, an additional sampling for epilithon and chlorophyll *a* periodwas conducted in late July was added at sites with variable buffer, uniform buffer, and a subset of reference sites(Supplemental).

In the labAfter thawing, each sample was split into two aliquots after thawing; the aliquot for epilithon standing stockbiomass, as ash free dry mass (AFDM), was sieved, using 2 mm mesh, to remove large, non-algal particles, and filtered onto pre-combusted and pre-weighed GF/F filters (Whatman glass microfiber filters). These filters were then dried at 60ºC, weighed, combusted at 500ºC, and reweighed. The second aliquot was analyzed for chlorophyll *a* and phaeophytin concentration by filtering a known volume onto pre-combusted GF/F filters, and using the heated

ethanol methodology for pigment extraction [76]. All measurement of concentrations of pigments occurred using a Shimadzu UV-1700 UV-VIS spectrophotometer. Chlorophyll *a* pigment concentrations were corrected for acid strength and post-acidification time [77].

We also collected ~~A separate composite~~ algal samples ~~was also collected~~ for ~~later~~ analysis of algal taxonomic structure from sites. At each sampling time, a fifth composite sample was collected ~~Known areas of rocks were scrubbed~~ as described above and ~~and the total area recorded. The composite sample was~~ preserved in 0.5% formalin. Samples from 2011 (pre-harvest) and 2013 (post-harvest) at a~~A~~ subset of sites ~~from 2011 (pre-harvest) and 2013 (post-harvest)~~ were selected for analysis. The dominant algal genera were qualitatively noted in wet mounts in a Palmer Maloney chamber [78]. To determine diatom densities, 4-ml aliquots were heated with 25 ml of 30% hydrogen peroxide for one hour and rinsed six times with distilled water to remove oxidation by-products. Processed subsamples were evaporated onto coverslips and mounted to microscope slides with Z-Rax mounting medium, making permanent slides. Specimens along transects were examined under oil immersion at 1000× magnification using a Leica DM5000 compound microscope with differential inference contrast microscopy. Five hundred valves were identified to species and enumerated from each sample. Diatoms were identified to the lowest taxonomic level (usually species), using standard taxonomic literature (~~e.g.,~~ https://diatoms.org/) and regional literature [79,80]. Identifiable fragments were mathematically reconstituted to whole valve units when possible and included in total counts. The raw data were converted to densities and classified by growth forms, based on Rimet and Bouchez [81].

In addition to the annual late June sampling, in July 2016 we collected post-harvest epilithic algal samples on rocks and unglazed clay tiles to evaluate a) whether more algae were accruing mid-summer in comparison to early summer and b) whether epilithon standing stock and chlorophyll *a* differed on tiles in comparison to natural rocks. At a subset of the headwater streams, we placed six clean clay tiles (58 cm^2) into each site during sampling in June. One month later[53], four tiles from each site were scrubbed, and four standard rock samples (using methods above) were collected for analysis of epilithon standing stock and chlorophyll *a*.

Benthic sediment

Each sampling period, three randomly located samples of fine benthic sediment were collected within the 30 m headwater sites following LINX II protocols [82]. Five samples were collected from the downstream sites. This benthic sediment sampling was sequenced before the algal sampling to avoid substrate disturbance and resuspension of sediment during other instream sampling. After collection, samples were refrigerated and returned to the laboratory. Sediment samples were dried and weighed for calculations of mass of fine surface sediment per square meter.

Data analyses

We compared multiple statistical approaches to examine whether forest harvest treatments resulted in differences in responses between the pre- and post-harvest periods. We grouped our headwater study sites by four treatments – clearcut with variable buffer width (variable buffer; 0-7.5 m), clearcut with uniform buffer width (uniform buffer; 12 m), thinned with uniform buffer width (thinned; 15 m), and reference (no harvest during this study). Downstream sites were characterized into two groups based on whether there was harvest in the headwaters or no

harvest. As well as examining treatment groups, we also modelled differences in responses between pre- and post-harvest periods at individual headwater sites. Our analyses and models examined responses of chlorophyll *a* concentration, epilithon biomass, surface sediment, concentrations of DOC, DON, DIN, and SRP, DIN:SRP molar ratio, and canopy cover following treatments relative to pre-harvest. We also calculated Pearson correlations between epilithon, chlorophyll *a*, and key instream factors. A significance level of 0.05 was used for all tests.

Kolmogorov-Smirnov~~S2~~ test and Bootstrapped Means

An initial step was to compare the pre- and post-harvest cumulative distribution functions for each treatment group (~~clearcut_variable~~clearcut variable, ~~clearcut_uniform~~clearcut uniform, thinned, and reference) using the non-parametric Kolmogorov-Smirnov 2-sample test (KS2) [83]. This test identifies whether the distributions are different, but does not provide information about direction of difference. We next evaluated whether the 95% confidence intervals (CI) for the means for pre- and post-harvest responses within a treatment overlapped using a bias-corrected, accelerated (BCa) bootstrap [84]. We considered pre- and post-harvest means significantly different if their bootstrapped confidence intervals did not overlap and noted the direction of the post-harvest change. These analyses were only conducted for treatment groups, because sample sizes for individual watershed sites pre- and post-harvest were small.

Partial Least Squares and significance testing

Our third approach involved modeling the differences in responses between pre- and post-harvest periods using Partial Least Squares regression (PLS) [85] to further evaluate differences in instream responses. Partial Least Squares is a latent variable approach that maximizes regressor and regressand covariance and avoids problems with multi-collinearity. It creates multiple

models for a given data set using latent variables, which can be thought of as similar to principal components. ~~The “optimal” PLS model, essentially a notable change in slope of the root-mean-square-error of cross-validation (RMSECV) graph, which is usually at or near the minimum RMSECV (maximum R^2CV).~~ The criterion for selecting the “optimal” PLS model, essentially a notable change in slope of the root-mean-square-error of cross-validation (RMSECV) graph, which is usually at or near the minimum RMSECV (maximum R^2CV). If the R^2CV of a given model was negative or “small,” it was not used regardless of the R^2. Full (leave-one-out) cross-validation was used in the PLS modeling. Cross-validation allows for calculation of the R^2 of cross-validation (R^2CV), which we interpret as a more robust measure of model quality [86,87].

Categorical factors of ~~T~~treatment group and ~~H~~harvest period, or w~~W~~atershed and h~~H~~arvest period were converted to dummy variables and the last level then deleted to avoid linearly dependent regressors. The interactions were then created from these dummy variables. For the model examining effects of forest harvest treatments, Response = Treatment + Period + Treatment*Period, where “Period” refers to harvest pre-post and “*” indicates the interaction between factors. This resulted in a regressor array with three Treatments, one Period and three interactions and allowed seven PLS latent variables, and therefore, seven regression models to be created. The downstream model was the same symbolically as the headwater treatment model except that there were only two categories in the treatment factor. For the headwater watershed model (Response = Watershed + Period + Watershed*Period), the regressor array had eleven Watersheds, one Period and eleven Watershed-Period interactions.

We used Box-Cox transformations [88] to try to improve model quality as defined by R^2CV (S1 File). The R^2CV of the resulting models was typically better than those of models that did not use transformed responses or more standard transformations, such as the log transformation.

In a standard regression model, one would generally assume the ~~various measurements~~sites were independent of one another. In this study, this assumption was likely violated due to annual sampling at each site as well as the spatial distribution of treatments across watersheds which was a function of forest ownership. To address this concern, we employed Wu's jackknife [89] and the wild bootstrap [90,91] for significance testing. For Wu's jackknife, we used one of Wu's best performing methods ("full cross validation"). This provided a coefficient variance estimate, which was then used in a 2-sided t-test for each coefficient for each model. ~~To address this concern, we employed Wu's jackknife [89] and the wild bootstrap [90,91] for significance testing. For Wu's jackknife, we used one of Wu's best performing methods ("full cross validation"). This provided a coefficient variance estimate, which was then used in a 2-sided t-test for each coefficient for each model.~~ We also used the wild bootstrap method HC_3 to construct 95% confidence intervals from 2000 re-samplings. The wild bootstrap confidence intervals were used to compare model pre- and post- predictions, where two predictions were considered significantly different if their 95% confidence intervals did not intersect. For regression coefficient testing, if a given confidence interval did not include zero, then the null hypothesis (that the coefficient was zero) was rejected. Note that neither the semi-parametric Wu jackknife nor the non-parametric wild bootstrap require normally distributed model residuals.

For calculations involving degrees of freedom for PLS models, the method described in Krämer and Sugiyama [92] was employed. Both the regressor and response data were centered and scaled. All analyses were conducted in Matlab™ 2020b (Mathworks, 2020) using Matlab programs, except Box-Cox, genetic algorithm, wild bootstrap, and Wu jackknife analyses, which were written by D. Plaehn.

Results

Stream characterization

Headwater streams were steep (mean slope 14.6%) and shallow (mean depth 6.9 cm), with wetted widths ranging from 0.8 m to 2.0 m (Table 1). Downstream sites had lower gradients (mean slope 5.1%), and were wider (mean width 3.9 m) and deeper (mean depth 15.0 cm). At the time of sampling, streamflow ranged from 1.2 L/s to 6.6 L/s, in headwater streams, with slightly higher discharge in the two reference watersheds in Rock Creek, whereas downstream sites showed larger discharge (ranging from 15 – 43 L/s). Streamflow across years was fairly constant during the three weeks preceding sampling with an average coefficient of variation of 22% among watersheds and years. ~~Substrates were primarily gravel to cobble with some areas of bedrock. Pre-harvest, maximum June water temperatures ranged between 7.3 °C and 15.3 °C in headwater streams. After harvest, maximum June temperatures increased depending on the riparian treatment. Streams clearcut with variable buffers showed greater increases than streams with uniform buffers, thinned and reference streams (Fig 2B). In mid-summer periods, post-harvest temperatures at streams that were clearcut with variable buffers were significantly warmer than pre-harvest [16].~~

During the pre-harvest period, the percent of overstory light reaching the streams was low (average 15.5%) and comparatively uniform across sites (range 9-19%, Fig 2~~A~~). After harvest in 2013, sites clearcut with variable buffers showed increased light reaching the stream. ~~-~~The ~~Differences in~~ annual measurements of percent canopy closure collected with a densiometer showed that canopy closure at clearcut sites with variable buffers ~~pre- and post-harvest~~ ~~were~~as significantly different pre- and post-harvest in~~in~~ all three statistical approaches (Table 2, S1 Table). The three individual ~~individual~~ watersheds in the variable buffer group ~~all~~ each showed significant ~~decreases~~ changes in canopy closure pre- to post-harvest (Table 3). We did not observe significant changes in canopy closure ~~pre- to post-harvest~~ for other treatments or for other individual watersheds.

Fig 2. Changes in percent of light reaching streams. The percent of overstory light reaching the streams, measured using hemispherical photos in 2008, pre-harvest, and in 2013, post-harvest. ~~**Site conditions during pre- and post-harvest periods at h**eadwater sites.~~ Sites are organized from left to right by percentage of overstory light reaching the stream post-harvest. Bars show site averages and standard deviation. ~~A) Percent of light reaching the stream, measured using hemispherical photos and calculated as incoming radiation reaching the stream; B) Average daily maximum stream temperature plus standard deviation during June of each study year.~~

Pre-harvest, maximum June water temperatures ranged between 7.3~~-~~°C and 15.3~~-~~°C in headwater streams. ~~Afte~~In the post~~r~~-harvest period, maximum June temperatures were warmer than pre-harvest across all sites. ~~increased depending on the riparian treatment.~~ Streams clearcut with variable buffers showed greater increases (3.9°C, 2.5°C, and 3.1°C) than streams with uniform buffers (1.1°C, 0.7°C, 1.0°C)~~;~~, thinned (1.2°C) ~~and~~or reference streams (0.4°C ,1.1°C, 0.9°C, 0.7°C, 1.4°C) ~~-~~(Fig 3~~2~~A~~B~~). In mid-summer ~~periods~~, post-harvest temperatures at streams that were clearcut with variable buffers were significantly warmer than pre-harvest [19].

In the headwater streams, sSubstrates were primarily gravel to cobble with some areas of bedrock. The average mass of fine benthic sediment (Fig 3B) did not significantly change post-harvest in any of the treatment groups (Table 2), within individual headwater watersheds (Fig 3B, Table 3, S2 Table) or at downstream sites (S3 Table).

Table 2. Statistical differences between pre- and post-harvest periods for headwater treatment groups.

Treatment group	Canopy closure	DOC	SRP	DON	DIN	DIN:SRP Molar Ratio	Epilithon biomass	Chl a	Surface sediment
**A. Kolmogorov-Smirnov two sample test p-values**									
~~Clearcut_Variable~~Clearcut variable	**0.00***	0.43	0.43	0.99	**0.00***	**0.02***	0.98	0.92	1.00
~~Clearcut_Uniform~~Clearcut uniform	0.57	0.86	0.96	0.24	**0.00***	**0.00***	0.17	**0.05***	0.54
Thinned	0.48	1.00	0.11	0.53	0.11	0.11	0.26	0.88	0.26
Reference	0.77	0.50	0.77	0.28	0.50	0.77	**0.04***	0.06	0.38
**B. Comparison of 95% BCa Bootstrap confidence intervals**									
~~Clearcut_Variable~~Clearcut variable	**decrease**	0	0	0	**increase**	**increase**	0	0	0
~~Clearcut_Uniform~~Clearcut uniform	0	0	0	0	**increase**	**increase**	0	0	0
Thinned	0	0	0	0	0	0	0	0	0
Reference	0	0	0	0	0	0	0	**decrease**	0
**C. Pre-Post comparison of PLS Model predictions**									
~~Clearcut_Variable~~Clearcut variable	**B/C***	--	C/C	A/AB	**C/AB***	**B/A***	--	AB/AB	--
~~Clearcut_Uniform~~Clearcut uniform	AB/AB	--	A/A	AB/A	**C/A***	**C/AB***	--	A/AB	--
Thinned	AB/B	--	BC/BC	AB/AB	**C/B***	BC/AB	--	A/AB	--
Reference	A/AB	--	B/AB	AB/AB	BC/BC	BC/ABC	--	AB/B	--

~~Bold text~~An asterisk and bold text indicates significant differences between pre- and post-harvest periods. B). "0" indicates the pre- and post-harvest confidence intervals (CI) overlapped; "increase" indicates that the lower bound of the post-harvest CI was greater than the upper bound of the pre-harvest CI. "decrease" where pre-harvest CI was greater than post-harvest. C). Pre-Post comparisons of significance for PLS model predictions for each attribute were based on 95% confidence intervals using the wild bootstrap. Within an attribute, pre/post with the same letters are not significantly different. “A” denotes the largest value, “B” is second largest, etc. within a response variable. The lettering on PLS model predictions is based on comparing the Box-Cox model predictions, not transformed back the original scale. " --" indicates that the R^2CV was negative and therefore the significance was not reported. Dissolved organic carbon (DOC), Soluble reactive phosphorus (SRP), Dissolved organic nitrogen (DON), Dissolved inorganic nitrogen (DIN), Chlorophyll a (Chl a).

Water chemistry

During our annual early summer sampling, concentrations of dissolved nutrients and DOC in these forested headwater streams were low and varied among watersheds. Pre- and post-harvest responses were not significantly different for headwater treatment groups for DOC and DON (Table 2) or for individual headwater watersheds (Table 3). Average DOC ranged from 0.240 to 0.883 mg C/L and average DON from 0.005 to 0.083 mg N/L (Fig 34A, 3C). Because t; concentrations did not significantly differ between pre- and post-harvest for headwater treatment groups (Table 2A, 2B) or for individual headwater watersheds (Table 3). The R^2 CV was negative for DOC in the treatment group PLS model (S1 Table), ; therefore, the significance for the PLS model was not reported for DOC (Table 2C). ; Average DON varied from 0.005 to 0.083 mg N/L (Fig 4B). pre- and post-harvest responses were not significantly different for headwater treatment groups (Table 2) or for individual headwater watersheds (Table 3). DIN showed significant increases post-harvest at all clearcut treatment groups (Table 2) DIN concentrations post-harvest in clear-cut treatments were approximately six times greater than pre-harvest, relative to a slight increase at the reference and thinned watersheds post-harvest (1.3x and 1.7x, respectively) (Fig 5A). The greatest change pre- to post-harvest in DIN occurred at GS3 where average concentration increased from 0.008 mg N/L pre-harvest to 0.334 mg N/L in the fourth year post-harvest (S4 Table). DIN was predominately composed of nitrate (S4 Table); c mg N/L.oncentrations of nitrate in 2013, the first year post-harvest were not the highest observed in the post-harvest period. SRP concentrations showed very little change following harvest (Fig 4D, Table 2). Some watersheds, PH2, PH3, and RK1, had substantially higher concentrations than others. Pre- to post-harvest changes in DIN:SRP molar ratio mirrored the increases in DIN for watersheds clearcut with variable buffers, but showed much smaller

increases post-harvest in the clearcut with uniform buffer group (Fig 4E). Molar ratios for both clearcut treatment groups were significantly greater post-harvest (Table 2), but in the analysis for individual watersheds, only GS3 and UM2 were significantly higher (Table 3). The average molar ratios of DIN:SRP concentrations in the headwaters ranged from 2.5 to 161 pre-harvest and up to 377377 post-harvest at GS3 (Fig 4E4).

Fig 43. Average concentrations of dissolved nutrients and carbon at headwater sites. Sites are organized from left to right by percentage of overstory light reaching the stream post-harvest. A) Dissolved organic carbon (DOC), B) Dissolved organic nitrogen (DON). C) Dissolved inorganic nitrogen (DIN), D) Soluble reactive phosphorus (SRP), E) Molar ration DIN:SRP. Bars show averages plus standard deviation during early summer annual sampling.

Table 3. Statistical summary from PLS model comparing pre- to post-harvest responses for individual headwater watersheds.

Treatment group	Watershed	Canopy closure	DOC	SRP	DON	DIN	DIN:SRP Molar Ratio	Epilithon biomass	Chl *a*
~~Clearcut_Variable~~Clearcut variable	GS3	**BC/D***	CD/CD	G/G	ABC/ABC	**D/AB***	**C/A***	AB/AB	AB/ABC
~~Clearcut_Variable~~Clearcut variable	UM2	**AB/C***	BC/BC	G/FG	ABC/ABC	**CD/A***	**BC/A***	AB/AB	BC/C
~~Clearcut_Variable~~Clearcut variable	UM3	**B/C***	BC/BC	E/E	BC/BC	CD/BC	BC/BC	B/BC	B/BC
~~Clearcut_Uniform~~Clearcut uniform	PH1	ABC/BC	AB/A	E/E	ABC/ABC	**CD/AB***	C/BC	BC/CD	AB/ABC
~~Clearcut_Uniform~~Clearcut uniform	PH2	AB/AB	BC/ABC	A/AB	BC/C	**CD/AB***	C/C	BC/CD	ABC/BC
~~Clearcut_Uniform~~Clearcut uniform	PH4	AB/AB	C/CD	DE/D	BC/C	**C/A***	B/BC	AB/ABC	BC/BC
Thinned	GS2	AB/ABC	BC/ABC	FG/FG	B/BC	CD/BC	BC/BC	BC/CD	AB/A
Reference	UM1	ABC/ABC	**D/E***	FG/FG	BC/ABC	D/D	C/C	**C/D***	C/C
Reference	GS1	AB/AB	AB/AB	FG/F	AB/A	A/A	AB/A	AB/AB	AB/AB
Reference	PH3	A/A	CD/CD	B/B	ABC/BC	B/B	C/C	A/AB	AB/ABC
Reference	RK1	BC/BC	CD/CD	C/C	ABC/ABC	D/D	C/C	BC/CD	BC/C
Reference	RK3	AB/AB	B/AB	FG/FG	BC/C	BC/BC	BC/B	BC/C	BC/C

Significance in PLS models is based on 95% confidence intervals calculated using Wild Bootstrap. Within a site, pre/post comparisons with the same letters are not significantly different. An asterisk and bold text indicates significant differences between pre- and post-harvest periods. "A" denotes the largest value, "B" is second largest, etc within a response variable. The lettering on PLS model predictions is based on comparing the Box-Cox model predictions, not transformed back the original scale. Dissolved organic carbon (DOC), Soluble reactive phosphorus (SRP), Dissolved organic nitrogen (DON), Dissolved inorganic nitrogen (DIN), Chlorophyll *a* (Chl *a*).

The PLS model for individual watersheds showed significant post-harvest increases in DIN in four of the six clearcut watersheds (Table 3). The thinned watershed, GS2, showed a significant increase in DIN post-harvest in the PLS model of treatment groups but not in the watershed PLS model. Because the watershed PLS model had a higher R^2CV than the associated model of treatment groups, the watershed model could be viewed as more reliable. The molar ratios of DIN:SRP concentrations ranged from 2.5 to 377 (Table 4).

Table 4. Molar ratios of DIN to SRP.

Catchment	Watershed	DIN:SRP Molar Ratio			
		Pre-harvest	SD Pre	Post-harvest	SD Post
Upper Main	UM 1	5.9	2.6	13.8	7.7
	UM 2	35.2	7.4	170.0	72.4
	UM 3	42.5	24.2	25.0	6.6
	UM Downstream	82.3	12.9	44.5	7.6
Gus	GS 1	161.2	78.6	153.2	31.4
	GS 2	37.5	6.4	48.4	13.5
	GS 3	12.5	2.4	377.6	300.8
	GS Downstream	64.2	19.0	73.6	23.4
Pothole	PH 1	11.2	2.6	52.1	17.7
	PH 2	2.9	0.7	16.9	9.1
	PH 3	8.0	1.3	8.6	1.5
	PH 4	13.5	2.3	65.7	32.3
	PH Downstream	8.2	1.4	14.3	14.3
Rock	RK 1	2.5	0.5	2.7	2.7
	RK 3	45.8	4.0	57.9	11.9
	RK Downstream	70.5	9.1	36.1	2.4

DIN is dissolved inorganic nitrogen. SRP is soluble reactive phosphorus.
SD is standard deviation.

Although this is not the full characterization of N:P ratios, the DIN:SRP molar ratio can be considered as a characterization of relationships between N and P. Pre- to post-harvest changes in molar ratio mirrored the increases in DIN; both clearcut treatment groups were significantly

greater post-harvest (Table 2). In the watershed models, molar ratios were significantly greater for five of the six clearcut watersheds (Table 3).

Nutrient and DOC concentrations at downstream sites were very similar to those in the headwaters (Fig 64). As observed at headwater sites, downstream DOC, SRP, and DON did not show significant differences between pre- and post-harvest periods (S3 Table). The increased concentration of DIN post-harvest in clearcut headwaters did not result in a consistent significant increase of DIN at their downstream study sites; the analysis of overlapping 95% confidence intervals showed DIN to be significantly greater post-harvest period downstream of harvested headwaters, but the other analyses did not (S3 Table). Interestingly, the downstream of reference site showed significant differences for DIN and DIN:SRP molar ratios post-harvest. The comparison of 95% confidence intervals and the PLS Models indicated that the DIN concentrations decreased post-harvest at the reference site, possibly related to dryer and warmer conditions. Comparisons from the PLS model were not reported for DOC, DON, or molar ratio; the R^2CVs were negative, indicating that the model quality was poor (S3 Table).

Fig 54. Average concentrations of nutrients at downstream sites during pre- and post-harvest periods. A) Dissolved organic carbon (DOC), B) Dissolved organic nitrogen (DON), C) Dissolved inorganic nitrogen (DIN), D) Soluble reactive phosphorus (SRP), E) Molar ratio DIN:SRP. Bars show averages plus standard deviation during early summer annual sampling. RKDS is the reference for the downstream sites. UMDS is downstream of variable buffer sites, PHDS is downstream of uniform buffer sites and GSDS has mixture of harvest groups upstream.

Epilithic algal biomass and chlorophyll *a*

Average epilithon ~~standing stock~~biomass at headwater sites ranged from 3.8-14.3 g AFDM/m^2 (Fig 6~~5~~A). Post-harvest, epilithon biomass was not significantly different from pre-harvest levels in any of the harvested headwater groups (Table 2) or individual headwater watersheds that were harvested(Table 3). A reference watershed (UM1) had significantly lower biomass post-harvest (Table 3) and likely influenced KS2 test that showed significantly different distribution for the reference group (Table 2). In the PLS watershed model, the multi-comparison lettering (Table 3) based on wild bootstrap prediction confidence intervals, was indicative of a general decrease during the post-harvest period, in agreement with Fig 6~~5~~. The R^2 CV was zero for epilithon in the PLS model for headwater treatments (S1 Table), therefore the model significance was not reported (Table 2C).

Fig 6~~5~~. Average epilithon ~~standing stock~~biomass and ~~f~~ chlorophyll *a* concentration~~s~~ at headwater sites. Sites are organized from left to right by percentage of overstory light reaching the stream post-harvest. A) Average epilithon ~~standing stock~~biomass as ash free dry mass (AFDM) plus standard deviation; B) Average chlorophyll *a* concentration plus standard deviation. Samples were collected annually in early summer.

Average chlorophyll *a* concentration ranged from 3.3-13.0 mg/m^2 (Fig ~~5~~6B). There were no significant differences in chlorophyll *a* pre- to post-harvest in clearcut with variable buffer group or in the thinned treatment (Table 2). The KS2 analyses, which compares distributions but not means, was the only test that suggested significant differences for post-harvest chlorophyll *a* in the clearcut with uniform buffer treatment (Table 2A). The Reference group showed a significant decrease in the comparison of 95% BCa confidence internals (Table 2B). Although PLS analysis of Treatment groups showed no significant change for any group (Table 2C), the PLS model

R^2CV for chlorophyll *a* was low, suggesting weak predictive power (S1 Table). No significant differences were identified for individual headwater watersheds (Table 3).

An analysis of the algal cell counts from a subset of headwater sites found that the June algal community was primarily diatoms in pre-harvest and post-harvest samples (Table 54); no

Fig 7. Chlorophyll *a* on rocks versus tiles. Chlorophyll *a* concentration on native rocks in June and July 2016 and on tiles in July 2016, after 30 days of colonization in the streams. This subset of sites is similarly organized from left to right by percentage of overstory light reaching the stream post-harvest. PH1, RK1 and RK3 were not sampled. Bars show average plus standard deviation.

filamentous algae were present. *Achnanthidium minutissimum* was the most common taxon in the epilithic algal samples at three of the five sites where taxa were identified pre- and post-harvest. At four of the five sites, oOne of the two most common species was the same during in pre- and post-harvest periods at four of the five sites. No clear shifts in growth form were noted among the treatments.

Table 54. The most common and second most common algal taxa in selected headwater streams, based on cell counts of epilithon samples. Growth forms are indicated as low (L) and high (H) profiles, based on classification by Rimet and Bouchez (2012).

Treatment group	Watershed	Period	Most Common Algal Ttaxon	Second Most Common Algal Taxon
Clearcut_variableClearcut variable	GS3	Pre	*Gomphonema minutum* (H)	*Meridion circulare* (L)*Meridion circulare*
		Post	*Achnanthidium minutissimum* (L)	---
Clearcut_variableClearcut variable	UM2	Pre	*Planothidium lanceolatum* (L)	*Achnanthidium minutissimum* (L)

		Post	*Achnanthidium minutissimum* (L)	*Achnanthidium cf. linearis* (L)
~~Clearcut_variable~~Clearcut variable	UM3	Pre	*Achnanthidium minutissimum* (L)	*Achnanthidium cf. deflexum* (L)
		Post	*Achnanthidium minutissimum* (L)	*Achnanthidium cf. linearis* (L)
~~Clearcut_uniform~~Clearcut uniform	PH4	Pre	*Rhoicosphenia abbreviatum* (L)	*Nitzschia inconspicua* (L)
		Post	*Rhoicosphenia abbreviatum* (L)	*Planothidium lanceolatum* (L)
Reference	UM1	Pre	*Gomphonema minutum* (H)	*Planothidium lanceolatum* (L)
		Post	*Planothidium lanceolatum* (L)	*Gomphonema minutum* (H)

At the downstream sites, epilithon ~~standing stock~~biomass and chlorophyll *a* concentration in the downstream of harvested group were generally lower in the post-harvest period~~similar to headwater values~~ (Fig 7~~8~~). The group of sites ‘downstream of harvested” showed ~~y had~~ significantly different distributions for both epilithon mass and chlorophyll *a* concentration between pre- and post-harvest periods using ~~in~~ the KS2 test (S3 Table) and e~~ and e~~pilithon biomass ~~showed~~ significantly decreased in the analysis of 95% confidence intervals ~~(S3 Table)~~. ~~In analyses of pre- and post-harvest responses~~ U~~u~~sing the ~~confidence intervals and~~ PLS model of

Fig 7~~8~~. Average epilithon ~~standing stock~~biomass and concentration of chlorophyll a at downstream sites during pre- and post-harvest periods. A) Epilithon ~~standing stock~~biomass as ash free dry mass (AFDM); B) Chlorophyll *a* concentration. Bars show early summer average plus standard deviation. RKDS was the reference for the downstream sites. UMDS was downstream of variable buffer sites, PHDS was downstream of uniform buffer sites and GSDS had mixture of harvest groups upstream.

pre- to post-harvest comparisons, neither epilithon nor chlorophyll *a* ~~did not~~ showed significant differences across these time periods.~~s.~~

C~~C~~hlorophyll *a* concentrations were significantly correlated with epilithon biomass during pre-harvest period (R^2 =0.446, Fig 8a; R^2=0.521, Fig 8B). However, during the post-harvest period, the correlations were significant only for reference sites (R^2 =0.444, Fig 8B).~~,~~ Chlorophyll *a* was not significantly correlated with canopy closure at reference or harvested sites, pre- or post-harvest (Fig 8C, D). Epilithon was not correlated with DIN at harvested sites ~~not correlated with~~ (Fig 8E); only at reference sites during post-harvest period was the correlation between epilithon and DIN significant (R^2=0.300, Fig 8F). The correlation between epilithon and DIN:SRP molar ratios was significant for harvested sites in the post-harvest period (R^2 =0.149, Fig 8G) and likely greatly influenced by several high molar ratios. No correlations for epilithon and ratios at reference sites (Fig 8H). ~~the amount of light reaching the stream, concentrations of DIN or TDN:TDP molar ratios (Fig 6). Our comparison of the influence of natural or artificial substrate and seasonality on chlorophyll *a* noted lower concentrations of chlorophyll *a* in July than in June on rocks, except at UM3 (Fig 7). The tiles, which had been in the streams for 30 days, had much lower chlorophyll *a* than the existing instream rocks.~~

Formatted: Not Highlight

Formatted: Not Highlight

Fig ~~6~~8. Correlations ~~with~~ between ~~chlorophyll *a*~~chlorophyll *a*, epilithon and instream factors for headwater sites. These correlations compare site averages for each year and site during pre- and post-harvest periods. Headwater sites that were harvested are in the left column (A, C, E, G) and those were reference sites are in the right column (B, D, F, H). C~~C~~hlorophyll *a* versus ~~light~~ epilithon (A, B); Chlorophyll *a* versus canopy closure (C, D); Epilithon versus ~~nitrate~~ DIN (E, F); Epilithon ~~(C, D)~~ versus molar ratios of ~~TDN:TDP~~DIN:SRP (~~molar ratios~~ Dissolved Inorganic Nitrogen:Soluble Reactive Phosphorus) (G, H). Data from ~~during~~ pre-harvest period~~s~~ are indicated as~~(~~ dark green circles~~)~~ and from the post-harvest period~~s~~ are shown as ~~(~~triangles~~)~~. R^2 values for the correlations where p-values equal to or less than 0.05. ~~A, C and E: Clearcut sites with variable buffers and uniform buffers. B, D, and F: Reference sites. Dissolved inorganic nitrogen (DIN), Total dissolved nitrogen (TDN), Total dissolved phosphorus (TDP).~~

Discussion

~~Following forest harvest~~Retention of vegetated riparian buffers can reduce impacts of forest harvest on stream ecosystems. In the Trask River Watershed study, regardless of width of riparian buffer treatments, stream chemistry responses showed increased DIN, with no change in concentrations of dissolved organic carbon, soluble reactive phosphorus, or benthic sediment. The sites with uniform 12 m buffers maintained canopy closure similar to the pre-harvest period, and stream algal biomass and chlorophyll *a* did not change post-harvest. Despite increased levels of both light and dissolved inorganic nitrogen in clearcut watersheds with variable riparian buffers, we did not observe increased standing stocks of epilithon or higher concentrations of chlorophyll *a*, in contrast to the common expectation of increased autotrophic resources in response to increased light and nutrients. Headwater streams have been overlooked in many prior regulatory discussions, therefore these findings are valuable in expanding our understanding of what conditions and factors are major drivers of responses in small headwater streams. Though our use of multiple analyses, we have greater confidence in the statistical significance of these relevant management practices.

-and disturbances to watersheds and riparian areas, a common expectation is higher concentrations of instream nutrients. Where riparian vegetation has been removed and light limitation eliminated, increased standing stocks of algal epilithon and higher concentrations of chlorophyll *a* are also expected. In this study, we observed increased concentrations of dissolved inorganic nitrogen during summer low flows but found no change in concentrations of dissolved organic carbon, soluble reactive phosphorus or benthic sediment. DIN increased both where riparian buffers were harvested and where 15 m uniform buffers remained. Despite increased levels of both light and dissolved inorganic nitrogen in streams with variable buffers during the post-harvest period, we did not observe greater standing stocks of epilithon or higher concentrations of chlorophyll *a*. Propagation of any responses downstream was negligible.

The challenges of distinguishing statistical significance in landscape experiments

There are multiple challenges to distinguishing statistical significance in field experiments. Research into responses to landscape treatments are conducted over a large area and for a number of years. These large-scale field experiments are expensive and labor-intensive and consequently have low sample sizes [37,39,93], and the focal metrics often exhibit high spatial and temporal variability [54]. Our study design planned that individual watersheds would be replicates of a specific harvest treatment, but study watersheds are never true replicates [34]. Our study sites within each treatment group showed variability in site conditions and responses [19,68]; and in this study, we explored this variability by including examination of pre- and post-harvest responses within each watershed. The conditions in reference watersheds were not static

and drivers of ecosystem processes can vary among reference watersheds as well as between reference and treated watersheds. The analyses of treatment groups were crucial for evaluation of forestry best management practices, while the analyses of individual watersheds addressed the variability in responses across siteschemistry, stream [19,34,69]. Climatic events added to the complexity of disentangling responses to experimental treatments, and highlighted the importance of reference sites and sampling pre- and post-disturbance.

Our findings from this complex landscape-scale experiment have potentially broad implications for forest management and policy issues and wWe recognize that statistically significant findings can havebe a major influencefactor for incorporation of the findings to informing management and policy. ,The multiple analyses were applied to the question of pre-post differences with the belief that consensus among methods would provide a stronger result than any one method by itself. The use of multiple methods was further warranted given the data challenges of spatial and temporal dependency, small sample sizes, and the potential of not finding an acceptable PLS model given the stringent quality criterion of R^2CV. and therefore we conducted mMultiple statistical tests allowed us to so as to be able to examine evaluate means (BCa bootstrap) and distributions (KS2) for pre-post comparisons, and simultaneously assess responses across all treatments or watersheds using PLS models. The non-parametric KS2 and BCa bootstrap both used small sample sizes which can be problematic [94–96]

, but by bootstrapping, the confidence intervals were considered conservative (i.e. larger) for non-independent data [97]. concurrence among the analyses. We recognize that statistically significant findings have a major influence to informing management and policy. We also acknowledge that statistical responses can be subjective to the types of analyses conducted and the assumptions within those analyses. Our multi-analysis approach leveraged the study design

of pre-post, reference-treatment data, but unlike most Before-After-Control-Intervention (BACI) and repeated measures tests;, our analyses of significance did not depend upon normally distributed or independent responses data for comparisons.

The challenges of distinguishing statistical significance of field experiments are multiple. Research into landscape treatments are conducted over a large area and for number of years, and therefore the focal metrics often exhibit high spatial and temporal variability. These large-scale field experiments are expensive and labor-intensive and consequently have low sample sizes [78]. Study watersheds are never true replicates, even if they are adjacent, and have similar overstory vegetation and legacies of prior disturbance or treatments; geology, soils, substrates, aspect, stream temperatures, and other factors differ. In addition, the conditions in reference watersheds are not static – trees continue to grow, understory vegetation shows successional changes – and drivers of ecosystem processes vary among reference watersheds as well as between reference and treated watersheds. Finally, high year-to-year variability in weather, coupled with projections of increased frequency of extreme events, adds to the complexity of disentangling responses to experimental treatments, and highlights the importance of reference sites and sampling pre- and post-disturbance.

Stream chemistry responses to riparian and watershed harvest

The increased in instream concentrations of DIN concur with results from other studies of stream chemistry responses to forest management in nitrogen-limited ecosystems [35,37,98,99]. Early studies in non-nitrogen limited regions found extremely high concentrations of nitrate following harvest [37,42,100], especially during storm flows. Our sampling timing was focused

on early summer, non-storm periods, which in these oligotrophic ecosystems, is a time when nutrients are generally at their minimum concentrations [101,102]. The magnitude of DIN increase in this study was substantial relative to the pre-harvest period, but the absolute concentrations values were relatively low concentrations still and comparable to studies in western US that documented small increases in nitrogen after forest harvest without riparian buffers [35,39,93]. Our sampling timing was focused on early summer, non-storm periods [], which in these oligotrophic ecosystems, is a time when limiting nutrients are at their minimum concentrations [102,103].

Responses of other nutrients and carbon to forest harvest and removal of riparian buffers has shown mixed results. As observed for some studies in the Pacific Northwest [42,100,104], forest harvest in the Trask did not result in increased concentrations of additional analytes, including DON, DOC, or phosphorus. However, in other regions, such as boreal forests, increased increased export of DON and DOC following harvest has occurred [40,101]. Although responses of stream phosphorus to forest harvest have been less studied[42], adsorption of P to soil and DOC can enhance its mobility and export [101], with site specific differences in soil and vegetation type likely controlling these dynamics. Although we observed a wide range of background phosphorus concentrations across Trask sites, our study did not show changes in instream phosphorus concentrations post-harvest at any sites. The Oregon Coast Range, where the Trask River watershed is located, is considered to be comparatively nitrogen rich, in part related to inputs from nitrogen-fixing alder vegetation [105] and likely phosphorus limited as a function of geology and weathering [106]. Even where phosphorus normally occurs at higher concentration related to recent volcanic history, including western Cascade Mountains [42,106],

Formatted: Highlight

Field Code Changed

Formatted: Font: (Default) Times New Roman, 12 pt

increased phosphorus concentrations have not generally been observed following forest harvest or watershed disturbances [35,101,107] unless substantial transport sediment occurred [35,39].

The magnitude of DIN increase in this study was low but comparable to studies in western US that documented small increases in nitrogen after forest harvest [35,39,93]. Nutrient increases following removal of vegetation have been hypothesized to be driven by a reduced demand for nitrate where there is less vegetation, coupled with mobilization of nitrate related to soil disturbance, decaying organic matter and microbial nitrification [38,99]. However, we were surprised the increased DIN concentrations in our streams occurred even where riparian buffers were retained.

HoweverIt is interesting thSignificant e increases in d DIN concentrations in our streams occurred even where riparian buffers were retained. Maintaining forested riparian zones during watershed harvest is a widely recognized best management practice for protecting water quality by reducing nutrient and sediment delivery to streams [9,16]. -Stream nutrient increases following removal of vegetation have been hypothesized to be driven by a reduced demand for nitrate where there is less vegetation, coupled with mobilization of nitrate related to soil disturbance, decaying organic matter and microbial nitrification [38,99]. The effectiveness of riparian buffers to intercept, retain and process nutrients has been related to the width of the buffer, with the recognition that multiple other landscape factors, including slope, soil type, and depth of groundwater also provide key functions [16,23,43]. These Our high-gradient Trask watersheds have a mix of soil types [64] and a legacy of soil disturbances related to historical wildfire and subsequent salvage logging [19,70] that may have resulted in altereddisconnection of the connections between -subsurface hillslope flow paths from and rooting zones of riparian

Formatted: Not Highlight

vegetation [16]. Although transport of inorganic nitrogen to the Trask streams ~~wa~~may not have been fully ~~s not~~ intercepted in watersheds with ~~by~~ wider buffers, the wider buffers provided other water quality functions, including shade that ~~o limit~~prevented stream temperature increases [19].

Responses of other nutrients and carbon to forest harvest and removal of riparian buffers have been mixed. Similar to findings from other studies in the Pacific Northwest [42,100,104], we did not see increased concentrations of DON, DOC, or phosphorus. However, i~~I~~n other regions such as boreal forests, ~~however,~~ researchers have observed increased concentrations and export of DON and DOC following harvest [40,101]. Although increases of stream phosphorus following harvest are not common [42], adsorption of P to soil and DOC can enhance its mobility and export [101], with site specific differences in soil and vegetation type likely controlling these dynamics. Even where phosphorus occurs naturally at high concentrations, including western Cascade Mountains [42,106], an increase in phosphorus concentrations has not generally been observed following forest harvest or watershed disturbances [35,101,107], unless substantial transport of sediment occurred [35,39].

Downstream propagation of responses to headwater disturbances can be a concern [11] and has been observed ~~elsewhere, including changes to~~for ~~drinking~~ water quality [103,104], especially during stormflows [39]. ~~and macroinvertebrate assemblages [88]. Mixed responses have been noted for transfer of increased stream temperatures to downstream reaches [10,58]. Here we observed increased DIN only in the headwater study sites.~~ In nitrogen-limited ecosystems, nitrogen spiraling, uptake, and transformation would be expected~~can~~ to reduce instream concentrations of N as the water flows downstream [105,106]. At the Trask sites downstream of harvest, downstream propagation of upstream DIN increases ~~impacts~~ was not

definitive; our statistics showed mixed results, albeit with small sample sizes. which effectively focused the DIN responses to forest harvest in the Trask Watersheds to harvest-adjacent stream sites.

Formatted: Not Highlight

As observed in other studies in the Pacific Northwest [42,99,107], forest harvest in the Trask did not result in increased concentrations of additional analytes, including DON, DOC, or phosphorus. However, other studies have noted increased export of DON and DOC following harvest, reviewed by Kreutzweiser et al. [100]. Although responses of stream phosphorus to forest harvest have been less studied, adsorption of P to soil and DOC can enhance its mobility and export [100], with site specific differences in soil and vegetation type likely controlling these dynamics. Although we observed a wide range of background phosphorus concentrations across Trask sites, our study did not show changes in instream phosphorus concentrations post-harvest at any sites. The Oregon Coast Range, where the Trask River watershed is located, is considered to be comparatively nitrogen rich, in part related to inputs from nitrogen-fixing alder vegetation [108] and likely phosphorus limited as a function of geology and weathering [109]. Even where phosphorus normally occurs at higher concentration related to recent volcanic history, including western Cascade Mountains [42,109], increased phosphorus concentrations have not generally been observed following forest harvest or watershed disturbances [35,100,110] unless substantial transport sediment occurred [35].

Lack of epilithic algal responses

Increased nutrients [45,78,107,108] as well as increased light [29,109] have long been recognized to stimulate autotrophic responses in aquatic systems. W[46,79,108,109] hen increased nutrients coincide with increased light, many studies have documented an increase in

autochthonous resources and productivity instream [21,29,48,55]. Therefore, at our sites where both DIN and light increased post-harvest, we expected but did not observe to see increased standing stocks of epilithon and/or changes in chlorophyll *a*. However, we did not observe this expected response. Although we observed significant correlations between chlorophyll a and epilithon significant correlations between We surmised that lack of response it might be due to related to limitation of individual nutrients and/or co-limitation of nitrogen and phosphorus [58,59,107]. Our correlations showed limited relationships between Although the Trask DIN concentrations increased after harvest and approached proposed "breakpoint" and saturation concentrations associated with high chlorophyll values[110], phosphorus concentrations in the Trask (except in PH2, and PH3, and RK1) were generally lower than those notedproposed by other studies and used for stimulation predicting algal standing stocks and chlorophyll *a* of primary production [110,111]. Historically, stream water N:P ratios (evaluated here as DIN:SRP) have beenwere proposed as predictors of algal responses and have been included regularly in comparisons [55,110,112], while others have suggested that they might not be strong indicators of nutrient limitation [108]. In the Trask, P limitation may have been a major constraint at sites with high DIN:SRP molar ratios. HoweverAcross our , we documented a wide range of N:P ratios across sites, some in optimum ranges, epilithic algal stocks didn't increase change following harvest and correlations showed few relationships. In very small streams, co-limitation may involve multiple mechanisms i[59]ncluding nutrients [58,107] in combination with light [113] plus other factors.

.

The taxonomic composition of algal assemblages, seasonal and successional dynamics and grazing pressure can also influence their responses to disturbance [108]. Multiple Although

Formatted: Not Highlight

multiple studies of responses to forest harvest have observed taxonomic shifts and increased filamentous green algae following opening of the riparian canopy [2,45,46] and deposition of benthic sediment [56], yet after harvest [2,46,57], the Trask algal assemblages continued towere consistently dominated by small, low growing diatoms in pre-and post-harvest periods. *Achnanthidium minutissimum* is considered an early successional species, that competes well as in shaded headwater streams with low nutrients and high water velocities [79,114] but can be replaced by other taxa when inorganic nutrients and light increase [115]. Yet our small study streams at the upper tips of the stream network had minimal green algal taxa to take advantage of post-harvest changes. Diatoms are frequently used as indicators of water quality and watershed condition [79,114,116]; the diatoms in the Trask streams were indicators of high water quality and cold waters. Some diatom taxa are more responsive than others to changes in nutrients; Stelzer and Lamberti [112] noted that *Achnanthidium minutissimum* showed sensitivity to N:P ratios while other diatom taxa, such as *Gomphonema* sp., were sensitive to only increases in nitrate. be dominated by cold water diatoms post- harvest, even though in summertime, the streams with variable buffers warmed several degrees [19]. Filamentous algae were not present in these streams, unlike other studies where opening the riparian canopy [46] or deposition of benthic sediment [57] stimulated shifts in algal composition. Additionally, while Iincreased light availability can result lead toin shifts in community composition from low, prostrate to high upright algal growth forms (e.g., Bixby et al. Bixby et al. 2009[117]), yet the Trask algal assemblages showed no broad patterns shifts in growth forms associated with increased light availability related to more open canopy. and watershed condition[80,115,116] (cite).

Field Code Changed

~~*Achnanthidium minutissimum*is considered an early successional species, that competes well s with low nutrients and high water velocities [80,115]~~

Field Code Changed

Light flecks [57]~~(Warren et al. 2013)~~ in shaded stream beds, including reference sites, may have supported microhabitats of algal assemblages with higher profile taxa ~~members~~ (e.g., *Gomphonema minutum*). ~~Finally, some taxa are more responsive than others to changes in nutrients; Stelzer and Lamberti [113] noted that the diatom *Achnanthidium minutissimum* showed sensitivity to N:P ratios while other taxa, such as *Gomphonema* sp., were sensitive to only increases in nitrate.~~

Field Code Changed

Seasonality can be a major factor for composition of diatom assemblages as well as green algae and summer is the time of highest activity and biomass [59,108]. Because our annual sampling occurred in early summer, we examined algal responses later in the summer when temperatures were warmer and flows lower. However, additional sampling in July showed generally lower chlorophyll *a* and similar biomass of epilithon as in June (S2 Text), thereby confirming that we were not missing substantial algal responses post-harvest.

A~~Seasonality can be a major factor for green algae as well as diatom assemblages; summer is the time of highest activity and biomass [60,109]. Because our annual sampling occurred in early summer, we initially speculated that the epilithon responses might have been different later in the summer when temperatures were warmer and flows were lower. However, additional sampling in July showed lower chlorophyll *a* and epilithon standing stocks than in June, and verified that we were not missing substantial algal responses post-harvest.~~

~~The type of sampling substrates must be considered when comparing studies of algal responses to increased light and or nutrients. Many studies have used tiles or glass slides or nutrient diffusing substrates to sample chlorophyll a or standing stocks. Algal assemblages~~

~~sampled on tiles or other introduced substrates can be substantially different than the mixed or late successional communities on native rocks. Although Dodds et al. noted that their meta-analysis did not demonstrate a difference in chlorophyll on artificial substrates versus native rocks, our incubated tiles showed much lower standing stocks and chlorophyll *a* than we observed on natural substrates. This additional evidence confirmed that our findings were not biased by seasonality or substrate type.~~

~~A~~ final consideration of ~~possible~~ influences of ~~lack of~~ autochthonous response to disturbances ~~could b~~involves~~e~~ the potential influences of instream ~~due to~~ grazersing [55,109,115]. The Trask stream food webs communities had low abundances of grazers and macroinvertebrate scrapers and grazers did not increase post-harvest [68], as might have been expected if more autochthonous resources became available. ~~and reduction of epilithon standing stocks by invertebrates or amphibians. In these streams,~~ Ttailed frog tadpoles~~, which are active grazers, were~~ were present in only 3 of our 12 headwaters sites ~~(present in three of the headwater sites (one harvested site with variable buffers (~~UM2~~) and two sites with uniform buffers (~~, PH1 and PH2) [72], and their presence did not explain ~~. The streams without tadpoles~~ differences in chlorophyll *a* or ~~did not show greater~~ standing stocks of ~~epilithon~~algae as has been observed elsewhere [109] ~~post-harvest~~. ~~And the Trask stream invertebrate communities had low proportions of scrapers which did not increase post-harvest [69], as might have been expected if more resources became available.~~

Harpole et al. [58] noted that minimal or null responses of ~~primary producers~~autotrophic community to increased light and nutrients “are rarely discussed in the nutrient limitation literature”. In our review of the primary literature related to algal responses to disturbances, we found ~~a~~ only few studies where increased nitrogen or combination of light and nitrogen did not

lead to increased algal standing stocks and chlorophyll *a* -[59,118,119]. Underrepresentation of null responses in the literature could also bias meta-analyses [54,55,107,120]. Other studies have suggested the lack of observed response was due to low phosphorus [118] or seasonally shifting N:P ratios [108,121,122]. Meta-analyses [54,55] have suggested responses to nutrient enrichment were contingent on background concentrations of inorganic N and P with interactive effects of nutrients, light and turbidity. Yet, fFrequently, researchers and managers have expectations that removal of forest cover or increases in nutrients will result in straightforward and predictable algal increases that transfer to upper trophic levels [55] for more productive fisheries [49,51,52,123,124], - increase resources and abundances of (e.g. [124], http://www.ramp-alberta.org/resources/forestry/potential+effects.aspx) that , based on the general paradigm that light and nutrients stimulate instream primary producers [21,125].

Conclusions and management implications

Over time, an evolution of harvest methods and treatments and improvements of Best Management Practices have resulted in smaller magnitude of post-harvest impacts than historically observed [9,18,19,100] have resulted in smaller magnitude of post-harvest impacts than historically observed. Meanwhile, the stakes to quantify the impacts of forest management accurately and to inform best management practices and policies continue to be high and are regularly associated with controversy [32,61,125], especially in landscapes with mixed ownerships and objectives and increasing events of extreme weather. The Our inclusion of multiple analytical tests multiple analyses allowed us to examineplore the variability in responses and to examine the concurrence of statistical responsesof several analytical tests. This provided more certainty around our findings and better information for managers and policy makers in

Formatted: Font color: Text 1

their planning and evaluations of responses to management~~ ~~.~~treatments. A legacy of disturbance may have simplified Trask ecosystems, as evidenced by vegetation and habitat surveys, and reflected in the stream communities [69]. Although these highly modified forest ecosystems may not have been studied extensively, they are likely increasingly common in our anthropogenic landscape.~~

~~In conclusion, d~~Disturbance from forest harvest in these small watersheds led to increased ~~-~~instream dissolved nitrogen broadly while ~~and light~~ the wider riparian buffers maintained lower light levels representative of forested headwater streams. ~~but~~ However, this study reminds us that not all streams respond similarly to forest harvest and removal of riparian vegetation; our results did not fit the general expectation that increased light and or increased stream nitrogen after watershed disturbances stimulate algal responses. We postulate that multiple factors, including co-limitation of nutrients, driven by low phosphorus concentrations, which did not change, plus an algal community dominated by low light adapted diatoms rather than green algae, contributed to our findings of no change in biomass of epilithon or concentrations of chlorophyll *a*. Management and restoration activities which aim to increase abundances and standing stocks of fish by opening riparian canopies and adding nutrients would benefit from prior evaluation of algal community composition and co-limitation of nutrients before undertaking major modifications.

~~did not increase epilithic algal standing stocks or chlorophyll *a* concentration, regardless of width of riparian buffer. This study did not fit the general expectation that increases of light and stream nitrogen after watershed disturbances stimulate algal responses. While this paradigm has been~~

Formatted: Not Highlight

~~well documented in some systems, there are complex interactions that result in lack of predictable responses for all streams.~~

Acknowledgements

Bob Bilby, Liz Dent, Maryanne Reiter, Jason Dunham, Judy Li, Janel Sobota, Arne Skaugsat, and Bill Gerth were invaluable research partners. We thank Rich Van Driesche, Kylie Meyer, Brent Morrissette, Tim Glidden, Jenn King, Mark Meleason, and Katie Whitehead for multi-year field assistance plus many others too numerous to list. We appreciate the field and publication maps created by David Hockman-Wert. Any use of trade, firm, or product names is for descriptive purposes only and does not imply endorsement by the U.S. Government.

References

1. Moore RD, Richardson JS. Natural disturbance and forest management in riparian zones: comparison of effects at reach, catchment, and landscape scales. Freshw Sci. 2012;31: 239–247. doi:10.1899/11-030.1

2. Sabater S, Butturini A, Munoz I, Romani A, Wray J. Effects of removal of riparian vegetation on algae and heterotrophs in a Mediterranean stream. J Aquat Ecosyst Stress Recovery. 1998;6: 129–140.

3. Bixby RJ, Cooper SD, Gresswell RE, Brown LE, Dahm CN, Dwire KA. Fire effects on aquatic ecosystems: an assessment of the current state of the science. Freshw Sci. 2015;34: 1340–1350. doi:10.1086/684073

4. Rodriguez-Cardona BM, Coble AA, Wymore AS, Kolosov R, Podgorski DC, Zito P, et al. Wildfires lead to decreased carbon and increased nitrogen concentrations in upland arctic streams. Sci Rep. 2020;10: 8722. doi:10.1038/s41598-020-65520-0

5. Schaefer DA, McDowell WH, Scatena FN, Asbury CE. Effects of Hurricane Disturbance on Stream Water Concentrations and Fluxes in Eight Tropical Forest Watersheds of the Luquillo Experimental Forest, Puerto Rico. J Trop Ecol. 2000;16: 189–207. Available: https://www.jstor.org/stable/3068756

6. Kaushal SS, Mayer PM, Vidon PG, Smith RM, Pennino MJ, Newcomer TA, et al. Land use and climate variability amplify carbon, nutrient, and contaminant pulses: a review with management implications. J Am Water Resour Assoc. 2014;50: 585–614. doi:10.1111/jawr.12204

7. Gardiner EP, Sutherland AB, Bixby RJ, Scott MC, Meyer JL, Helfman GS, et al. Linking stream and landscape trajectories in the southern Appalachians. Environ Monit Assess. 2009;156: 17–36. doi:10.1007/s10661-008-0460-x

8. Oswalt SN, Smith WB, Miles PD, Pugh SA. Forest Resources of the United States, 2017: a technical document supporting the Forest Service 2020 RPA Assessment. Gen Tech Rep WO-97 Wash DC US Dep Agric For Serv Wash Off. 2019;97. doi:10.2737/WO-GTR-97

9. Cristan R, Aust WM, Bolding MC, Barrett SM, Munsell JF, Schilling E. Effectiveness of forestry best management practices in the United States: Literature review. For Ecol Manag. 2016;360: 133–151. doi:10.1016/j.foreco.2015.10.025

10. Penaluna BE, Olson DH, Flitcroft RL, Weber MA, Bellmore JR, Wondzell SM, et al. Aquatic biodiversity in forests: a weak link in ecosystem services resilience. Biodivers Conserv. 2016; 1–31.

11. Freeman MC, Pringle CM, Jackson CR. Hydrologic connectivity and the contribution of stream headwaters to ecological integrity at regional scales1. JAWRA J Am Water Resour Assoc. 2007;43: 5–14. doi:10.1111/j.1752-1688.2007.00002.x

12. Liu N, Dobbs GR, Caldwell PV, Miniat CF, Sun G, Duan K, et al. Quantifying the role of National Forest System and other forested lands in providing surface drinking water supply for the

conterminous United States. Gen Tech Rep WO-100 Wash DC US Dep Agric For Serv Wash Off. 2022;100. doi:10.2737/WO-GTR-100

13. Downing JA, Cole JJ, Duarte CM, Middelburg JJ, Melack JM, Prairie YT, et al. Global abundance and size distribution of streams and rivers. Inland Waters. 2012;2: 229–236. doi:10.5268/IW-2.4.502

14. Roon DA, Dunham JB, Torgersen CE. A riverscape approach reveals downstream propagation of stream thermal responses to riparian thinning at multiple scales. Ecosphere. 2021;12: e03775. doi:10.1002/ecs2.3775

15. Riis T, Kelly-Quinn M, Aguiar FC, Manolaki P, Bruno D, Bejarano MD, et al. Global overview of ecosystem services provided by riparian vegetation. BioScience. 2020;70: 501–514. doi:10.1093/biosci/biaa041

16. Sweeney BW, Newbold JD. Streamside forest buffer width needed to protect stream water quality, habitat, and organisms: a literature review. JAWRA J Am Water Resour Assoc. 2014;50: 560–584. doi:10.1111/jawr.12203

17. Gregory SV, Swanson FJ, McKee WA, Cummins KW. An Ecosystem Perspective of Riparian Zones: Focus on links between land and water. BioScience. 1991;41: 540–551. doi:10.2307/1311607

18. Hatten JA, Segura C, Bladon KD, Hale VC, Ice GG, Stednick JD. Effects of contemporary forest harvesting on suspended sediment in the Oregon Coast Range: Alsea Watershed Study Revisited. For Ecol Manag. 2018;408: 238–248. doi:10.1016/j.foreco.2017.10.049

19. Reiter M, Johnson SL, Homyack J, Jones JE, James PL. Summer stream temperature changes following forest harvest in the headwaters of the Trask River watershed, Oregon Coast Range. Ecohydrology. 2020;13: e2178. doi:10.1002/eco.2178

20. Roon DA, Dunham JB, Groom JD. Shade, light, and stream temperature responses to riparian thinning in second-growth redwood forests of northern California. Rossi S, editor. PLOS ONE. 2021;16: e0246822. doi:10.1371/journal.pone.0246822

21. Gregory SV. Effects of light, nutrients and grazers on periphyton communities in streams. Oregon State University. 1980.

22. Baker ME, Weller DE, Jordan TE. Improved methods for quantifying potential nutrient interception by riparian buffers. Landsc Ecol. 2006;21: 1327–1345. doi:10.1007/s10980-006-0020-0

23. Vidon PGF, Hill AR. Landscape controls on nitrate removal in stream riparian zones. Water Resour Res. 2004;40. doi:10.1029/2003WR002473

24. Gregory S, Boyer KL, Gurnell AM, International Conference of Wood in World R. The ecology and management of wood in world rivers. Bethesda, Md.: Bethesda, Md. : American Fisheries Society; 2003.

25. Benfield EF. Comparison of litterfall input to streams. J North Am Benthol Soc. 1997;16: 104–108. doi:10.2307/1468242

26. Dudley MP, Solomon K, Wenger S, Jackson CR, Freeman M, Elliott KJ, et al. Do crayfish affect stream ecosystem response to riparian vegetation removal? Freshw Biol. 2021;66: 1423–1435. doi:10.1111/fwb.13728

27. Erdozain M, Kidd KA, Emilson EJS, Capell SS, Kreutzweiser DP, Gray MA. Forest management impacts on stream integrity at varying intensities and spatial scales: Do abiotic effects accumulate spatially? Sci Total Environ. 2021;753: 141968. doi:10.1016/j.scitotenv.2020.141968

28. Richardson JS, Béraud S. Effects of riparian forest harvest on streams: a meta-analysis. J Appl Ecol. 2014;51: 1712–1721. doi:10.1111/1365-2664.12332

29. Murphy ML, Hawkins CP, Anderson NH. Effects of canopy modification and accumulated sediment on stream communities. Trans Am Fish Soc. 1981;110: 469–478.

30. Dodds WK, Bouska WW, Eitzmann JL, Pilger TJ, Pitts KL, Riley AJ, et al. Eutrophication of u.s. freshwaters: analysis of potential economic damages. Environ Sci Technol. 2009;43: 12–19. doi:10.1021/es801217q

31. Luke SH, Luckai NJ, Burke JM, Prepas EE. Riparian areas in the Canadian boreal forest and linkages with water quality in streams. Environ Rev. 2007;15: 79–97. doi:10.1139/A07-001

32. Richardson JS, Naiman RJ, Bisson PA. How did fixed-width buffers become standard practice for protecting freshwaters and their riparian areas from forest harvest practices? Freshw Sci. 2012;31: 232–238. doi:10.1899/11-031.1

33. Graham EB, Averill C, Bond-Lamberty B, Knelman JE, Krause S, Peralta AL, et al. Toward a generalizable framework of disturbance ecology through crowdsourced science. Front Ecol Evol. 2021;9. Available: https://www.frontiersin.org/articles/10.3389/fevo.2021.588940

34. Argerich A, Johnson SL, Sebestyen SD, Rhoades CC, Greathouse E, Knoepp JD, et al. Trends in stream nitrogen concentrations for forested reference catchments across the USA. Environ Res Lett. 2013;8: 014039. doi:10.1088/1748-9326/8/1/014039

35. Binkley D, Ice GG, Kaye J, Williams CA. Nitrogen and phosphorus concentrations in forest streams of the United States. JAWRA J Am Water Resour Assoc. 2004;40: 1277–1291. doi:10.1111/j.1752-1688.2004.tb01586.x

36. Swank WT. Stream Chemistry Responses to Disturbance. In: Swank WT, Crossley DA, editors. Forest Hydrology and Ecology at Coweeta. New York, NY: Springer; 1988. pp. 339–357. doi:10.1007/978-1-4612-3732-7_25

37. Likens GE, Bormann FH, Johnson Noye M, Fisher DW, Pierce Robert S. Effects of forest cutting and herbicide treatment on nutrient budgets in the Hubbard Brook Watershed Ecosystem. Ecol Monogr. 1970;40: 23–47. doi:10.2307/1942440

38. Feller M. Forest harvesting and streamwater inorganic chemistry in western North America: A review. JAWRA J Am Water Resour Assoc. 2005;41: 785–811. doi:10.1111/j.1752-1688.2005.tb03771.x

39. Gravelle JA, Ice G, Link TE, Cook DL. Nutrient concentration dynamics in an inland Pacific Northwest watershed before and after timber harvest. For Ecol Manag. 2009;257: 1663–1675. doi:10.1016/j.foreco.2009.01.017

40. Schelker J, Eklöf K, Bishop K, Laudon H. Effects of forestry operations on dissolved organic carbon concentrations and export in boreal first-order streams. J Geophys Res Biogeosciences. 2012;117. doi:10.1029/2011JG001827

41. Martin CW, Harr RD. Logging of mature Douglas-fir in western Oregon has little effect on nutrient output budgets. Can J For Res. 1989;19: 35–43. doi:10.1139/x89-005

42. Aubertin GM, Patric JH. Water quality after clearcutting a small watershed in west virginia. J Environ Qual. 1974;3: 243–249. doi:10.2134/jeq1974.00472425000300030012x

43. Mayer PM, Reynolds Jr. SK, McCutchen MD, Canfield TJ. Meta-analysis of nitrogen removal in riparian buffers. J Environ Qual. 2007;36: 1172–1180. doi:10.2134/jeq2006.0462

44. Hill AR. Nitrate removal in stream riparian zones. J Environ Qual. 1996;25: 743–755. doi:10.2134/jeq1996.00472425002500040014x

45. Lowe RL, Golladay SW, Webster JR. Periphyton response to nutrient manipulation in streams draining clearcut and forested watersheds. J North Am Benthol Soc. 1986;5: 221–229. doi:10.2307/1467709

46. Shortreed KS, Stockner JG. Periphyton biomass and species composition in a coastal rainforest stream in British Columbia: Effects of environmental changes caused by logging. Can J Fish Aquat Sci. 1983;40: 1887–1895. doi:10.1139/f83-219

47. Hill WR, Knight AK. Nutrient and light limitation of algae in two Northern California streams. J Phycol. 1988;24: 125–132.

48. Fanta SE, Hill WR, Smith TB, Roberts BJ. Applying the light:nutrient hypothesis to stream periphyton. Freshw Biol. 2010;55: 931–940. doi:10.1111/j.1365-2427.2009.02309.x

49. Murphy ML, Hall JD. Varied effects of clear-cut logging on predators and their habitat in small streams of the Cascade Mountains, Oregon. Can J Fish Aquat Sci. 1981;38: 137–145. doi:10.1139/f81-018

50. Kiffney PM, Richardson JS, Bull JP. Responses of periphyton and insects to experimental manipulation of riparian buffer width along forest streams. J Appl Ecol. 2003;40: 1060–1076.

51. Kaylor MJ, Warren DR. Linking riparian shade and the legacies of forest management to fish and vertebrate biomass in forested streams. Ecosphere. 2017;8: 1–19.

52. Hawkins CP, Murphy ML, Anderson NH, Wilzbach MA. Density of Fish and Salamanders in Relation to Riparian Canopy and Physical Habitat in Streams of the Northwestern United States. Can J Fish Aquat Sci. 1983;40: 1173–1185. doi:10.1139/f83-134

53. Wootton JT. River Food Web Response to Large-Scale Riparian Zone Manipulations. PLOS ONE. 2012;7: e51839. doi:10.1371/journal.pone.0051839

54. Francoeur SN. Meta-analysis of lotic nutrient amendment experiments: detecting and quantifying subtle responses. J North Am Benthol Soc. 2001;20: 358–368. doi:10.2307/1468034

55. Ardón M, Zeglin LH, Utz RM, Cooper SD, Dodds WK, Bixby RJ, et al. Experimental nitrogen and phosphorus enrichment stimulates multiple trophic levels of algal and detrital-based food webs: a global meta-analysis from streams and rivers. Biol Rev. 2021;96: 692–715. doi:10.1111/brv.12673

56. Hansmann EW, Phinney HK. Effects of logging on periphyton in coastal streams of Oregon. Ecology. 1973;54: 194–199.

57. Warren DR, Keeton WS, Bechtold HA, Rosi-Marshall EJ. Comparing streambed light availability and canopy cover in streams with old-growth versus early-mature riparian forests in western Oregon. Aquat Sci. 2013;75: 547–558. doi:10.1007/s00027-013-0299-2

58. Harpole WS, Ngai JT, Cleland EE, Seabloom EW, Borer ET, Bracken MES, et al. Nutrient co-limitation of primary producer communities. Ecol Lett. 2011;14: 852–862. doi:10.1111/j.1461-0248.2011.01651.x

59. Greenwood JL, Rosemond AD. Periphyton response to long-term nutrient enrichment in a shaded headwater stream. Can J Fish Aquat Sci. 2005;62: 2033–2045.

60. Alila Y, Kuraś PK, Schnorbus M, Hudson R. Forests and floods: A new paradigm sheds light on age-old controversies. Water Resour Res. 2009;45. doi:10.1029/2008WR007207

61. Biggs R, Carpenter SR, Brock WA. Spurious certainty: how ignoring measurement error and environmental heterogeneity may contribute to environmental controversies. BioScience. 2009;59: 65–76. doi:10.1525/bio.2009.59.1.10

62. Jones JA, Grant GE. Peak Flow Responses to Clear-Cutting and Roads in Small and Large Basins, Western Cascades, Oregon. Water Resour Res. 1996;32: 959–974. doi:10.1029/95WR03493

63. Beschta RL, Pyles MR, Skaugset AE, Surfleet CG. Peakflow responses to forest practices in the western cascades of Oregon, USA. J Hydrol. 2000;233: 102–120. doi:10.1016/S0022-1694(00)00231-6

64. Bywater-Reyes S, Segura C, Bladon KD. Geology and geomorphology control suspended sediment yield and modulate increases following timber harvest in temperate headwater streams. J Hydrol. 2017;548: 754–769. doi:10.1016/j.jhydrol.2017.03.048

65. ODF. Oregon Forest Practices Act Water Protection Rules: Riparian Management Areas and Protection Measures for Significant Wetlands. Salem, Oregon, Oregon Department of Forestry. 2010.

66. Boisjolie BA, Santelmann MV, Flitcroft RL, Duncan SL. Legal ecotones: A comparative analysis of riparian policy protection in the Oregon Coast Range, USA. J Environ Manage. 2017;197: 206–220. doi:10.1016/j.jenvman.2017.03.075

67. ODF. Northwest Oregon State Forests Management Plan, Appendix J: Management Standards for Aquatic and Riparian Areas. 2010.

68. Johnson SL, Li JL, Sobota JB, Ashkenas LR, Pollock AM, Meleason MA, et al. Macroinvertebrate responses to differing riparian treatments following forest harvest in the headwaters of Trask River watershed. For Ecol Manag. 2022;508: 119999. doi:10.1016/j.foreco.2021.119999

69. Bladon KD, Segura C, Cook NA, Bywater-Reyes S, Reiter M. A multicatchment analysis of headwater and downstream temperature effects from contemporary forest harvesting. Hydrol Process. 2018;32: 293–304. doi:10.1002/hyp.11415

70. Arismendi I, Groom JD, Reiter M, Johnson SL, Dent L, Meleason M, et al. Suspended sediment and turbidity after road construction/improvement and forest harvest in streams of the Trask River Watershed Study, Oregon. Water Resour Res. 2017;53: 6763–6783. doi:10.1002/2016WR020198

71. Steadman CL. Natural variability of nitrogen and phosphorus in a forested headwater stream system in the oregon coast range. Masters Thesis, Oregon State University. 2017.

72. Chelgren ND, Adams MJ. Inference of timber harvest effects on survival of stream amphibians Is complicated by movement. Copeia. 2017;105: 712–725. doi:10.1643/CE-16-573

73. Benjamin JR, Dunham JB, Johnson SL, Ashkenas L, Penaluna BE, Bilby RE, et al. Pathways of productivity and influences on top consumers in forested streams. For Ecol Manag. 2022;508: 120046. doi:10.1016/j.foreco.2022.120046

74. Webster JR, Valett HM. CHAPTER 8 - Solute Dynamics. In: Hauer FR, Lamberti GA, editors. Methods in Stream Ecology (Second Edition). San Diego: Academic Press; 2007. pp. 169–185. doi:10.1016/B978-012332908-0.50010-3

75. Platts WS, Armour C, Booth GD, Bryant M, Bufford JL, Cuplin P, et al. Methods for evaluating riparian habitats with applications to management. US Dept. of Agriculture, Forest Service, Intermountain Research Station; 1987.

76. Sartory DP, Grobbelear JU. Extraction of chlorophyll a from freshwater phytoplankton for spectrophotometric analysis. Hydrobiologia. 1984;114: 177–187.

77. Parker SP, Bowden WB, Flinn MB. The effect of acid strength and postacidification reaction time on the determination of chlorophyllain ethanol extracts of aquatic periphyton. Limnol Oceanogr Methods. 2016;14: 839–852. doi:10.1002/lom3.10130

78. Lowe RL, LaLiberte GD. Chapter 11 - Benthic Stream Algae: Distribution and Structure. In: Hauer FR, Lamberti GA, editors. Methods in Stream Ecology, Volume 1 (Third Edition). Boston: Academic Press; 2017. pp. 193–221. doi:10.1016/B978-0-12-416558-8.00011-1

79. Weilhoefer CL, Pan Y. Diatom assemblages and their associations with environmental variables in Oregon Coast Range streams, USA. Hydrobiologia. 2006;561: 207–219. doi:10.1007/s10750-005-1615-1

80. Edwards PM, Pan Y, Mork L, Thorne C. Using diatoms to assess river restoration: A pilot study in Whychus Creek, Oregon, USA. River Res Appl. 2020;36: 2089–2095. doi:10.1002/rra.3712

81. Rimet F, Bouchez A. Life-forms, cell-sizes and ecological guilds of diatoms in European rivers. Knowl Manag Aquat Ecosyst. 2012 [cited 9 Dec 2022]. doi:10.1051/kmae/2012018

82. Mulholland PJ. Lotic Intersite Nitrogen eXperiment II (LINX II): a cross-site study of the effects of anthropogenic land use change on nitrate uptake and retention in 72 streams across 8 different biomes (2003 – 2006). Environmental Data Initiative; 2015. doi:10.6073/PASTA/EAC34B6C1CFB0268DD76EF68A7A90BFC

83. Massey FJ. The Kolmogorov-Smirnov test for goodness of fit. J Am Stat Assoc. 1951;46: 68–78. doi:10.1080/01621459.1951.10500769

84. DiCiccio TJ, Efron B. Bootstrap confidence intervals. Stat Sci. 1996;11: 189–228. doi:10.1214/ss/1032280214

85. Martens H, Næs T. Multivariate Calibration. John Wiley & Sons; 1992.

86. Efron B. Estimating the error rate of a prediction rule: Improvement on cross-validation. J Am Stat Assoc. 1983;78: 316–331. doi:10.2307/2288636

87. Breiman L. Statistical Modeling: The Two Cultures (with comments and a rejoinder by the author). Stat Sci. 2001;16: 199–231. doi:10.1214/ss/1009213726

88. Box GEP, Cox DR. An analysis of transformations. J R Stat Soc Ser B. 1964;26: 211–252.

89. Wu CFJ. Jackknife, bootstrap and other resampling methods in regression analysis. Ann Stat. 1986;14: 1261–1295. doi:10.1214/aos/1176350142

90. Davidson R, Flachaire E. The wild bootstrap, tamed at last. J Econom. 2008;146: 162–169. doi:10.1016/j.jeconom.2008.08.003

91. Liu RY. Bootstrap Procedures under some Non-I.I.D. Models. Ann Stat. 1988;16: 1696–1708. doi:10.1214/aos/1176351062

92. Krämer N, Sugiyama M. The degrees of freedom of partial least squares regression. J Am Stat Assoc. 2011;106: 697–705. doi:10.1198/jasa.2011.tm10107

93. Sollins P, Cromack Jr. K, Mc Corison FM, Waring RH, Harr RD. Changes in nitrogen cycling at an old-growth douglas-fir site after disturbance. J Environ Qual. 1981;10: 37–42. doi:10.2134/jeq1981.00472425001000010007x

94. Hesterberg T. Bootstrap. WIREs Comput Stat. 2011;3: 497–526. doi:10.1002/wics.182

95. Büning H. Kolmogorov-Smirnov- and Cramèr-Von Mises Type Two-Sample Tests with Various Weight Functions. Commun Stat - Simul Comput. 2001;30: 847–865. doi:10.1081/SAC-100107784

96. Büning H. Robustness and power of modified Lepage, Kolmogorov-Smirnov and Crame´r-von Mises two-sample tests. J Appl Stat. 2002;29: 907–924. doi:10.1080/02664760220136212

97. Liu RY, Singh K. Using i.i.d. bootstrap inference for general non-i.i.d. models. J Stat Plan Inference. 1995;43: 67–75. doi:10.1016/0378-3758(94)00008-J

98. Webster JR, Knoepp JD, Swank WT, Miniat CF. Evidence for a regime shift in nitrogen export from a forested watershed. Ecosystems. 2016;19: 881–895. doi:10.1007/s10021-016-9974-1

99. Deval C, Brooks ES, Gravelle JA, Link TE, Dobre M, Elliot WJ. Long-term response in nutrient load from commercial forest management operations in a mountainous watershed. For Ecol Manag. 2021;494: 119312. doi:10.1016/j.foreco.2021.119312

100. Kreutzweiser DP, Hazlett PW, Gunn JM. Logging impacts on the biogeochemistry of boreal forest soils and nutrient export to aquatic systems: A review. Environ Rev. 2008;16: 157–179. doi:10.1139/a08-006

101. Ashkenas LR, Johnson SL, Gregory SV, Tank JL, Wollheim WM. A stable isotope tracer study of nitrogen uptake and transformation in an old-growth forest stream. Ecology. 2004;85: 1725–1739. doi:10.1890/03-0032

102. Vanderbilt KL, Lajtha K, Swanson FJ. Biogeochemistry of unpolluted forested watersheds in the Oregon Cascades: temporal patterns of precipitation and stream nitrogen fluxes. Biogeochemistry. 2003;62: 87–117. doi:10.1023/A:1021171016945

103. Gomi T, Sidle RC, Richardson JS. Understanding processes and downstream linkages of headwater systems. BioScience. 2002;52. doi:10.1641/0006-3568

104. MacDonald LH, Coe D. Influence of headwater streams on downstream reachs in forest areas. For Sci. 2007;53: 148–168.

105. Sobota DJ, Johnson SL, Gregory SV, Ashkenas LR. A stable isotope tracer study of the influences of adjacent land use and riparian condition on fates of nitrate in streams. Ecosystems. 2012;15: 1–17. doi:10.1007/s10021-011-9489-8

106. Bernhardt ES, Likens GE, Buso DC, Driscoll CT. In-stream uptake dampens effects of major forest disturbance on watershed nitrogen export. Proc Natl Acad Sci. 2003;100: 10304–10308. doi:10.1073/pnas.1233676100

107. Elser JJ, Bracken MES, Cleland EE, Gruner DS, Harpole WS, Hillebrand H, et al. Global analysis of nitrogen and phosphorus limitation of primary producers in freshwater, marine and terrestrial ecosystems. Ecol Lett. 2007;10: 1135–1142. doi:10.1111/j.1461-0248.2007.01113.x

108. Francoeur SN, Smith RA, Lowe RL. Nutrient limitation of algal biomass accrual in streams: seasonal patterns and a comparison of methods. J North Am Benthol Soc. 1999;18: 242–260. doi:10.2307/1468463

109. Kiffney PM, Richardson JS, Bull JP. Establishing light as a causal mechanism structuring stream communities in response to experimental manipulation of riparian buffer width. J North Am Benthol Soc. 2004;23: 542–555. doi:10.1899/0887-3593(2004)023<0542:ELAACM>2.0.CO;2

110. Dodds WK, Smith VH, Lohman K. Nitrogen and phosphorus relationships to benthic algal biomass in temperate streams. Can J Fish Aquat Sci. 2002;59: 865–874. doi:10.1139/f02-063

111. Hill WR, Fanta SE, Roberts BJ. Quantifying phosphorus and light effects in stream algae. Limnol Oceanogr. 2009;54: 368–380. doi:10.4319/lo.2009.54.1.0368

112. Stelzer RS, Lamberti GA. Effects of N: P ratio and total nutrient concentration on stream periphyton community structure, biomass, and elemental composition. Limnol Oceanogr. 2001;46: 356–367. doi:10.4319/lo.2001.46.2.0356

113. Warren DR, Collins SM, Purvis EM, Kaylor MJ, Bechtold HA. Spatial Variability in Light Yields Colimitation of Primary Production by Both Light and Nutrients in a Forested Stream Ecosystem. Ecosystems. 2017;20: 198–210. doi:10.1007/s10021-016-0024-9

114. Naymik J, Pan Y, Ford J. Diatom assemblages as indicators of timber harvest in coastal Oregon streams. J North Am Benthol Soc. 2005;24: 569–581.

115. Biggs BJF, Stevenson RJ, Lowe RL. A habitat matrix conceptual model for stream periphyton. Fundam Appl Limnol. 1998;143: 21–56. doi:10.1127/archiv-hydrobiol/143/1998/21

116. Stevenson RJ, Pan Y, Manoylov KM, Parker CA, Larsen DP, Herlihy AT. Development of diatom indicators of ecological conditions for streams of the western US. J North Am Benthol Soc. 2008;27: 1000–1016. doi:10.1899/08-040.1

117. Bixby RJ, Benstead JP, Douglas MM, Pringle CM. Relationships of stream algal community structure to catchment deforestation in eastern Madagascar. J North Am Benthol Soc. 2009;28: 466–479. doi:10.1899/07-171.1

118. Stockner JG, Shortreed KRS. Enhancement of autotrophic production by nutrient addition in a coastal rainforest stream on vancouver island. J Fish Res Board Can. 1978;35: 28–34. doi:10.1139/f78-004

119. McIntyre AP, Hayes MP, Ehinger WJ, Estrella SM, Schuett-Hames DE, Quinn, Timothy. (Technical Coordinators). Effectiveness of Experimental Riparian Buffers on Perennial Non-fish-bearing Streams on Competent Lithologies in Western Washington. Cooperative Monitoring, Evaluation and Research Report CMER 18-100, Washington State Forest Practices Adaptive Management Program, Washington Department of Natural Resources, Olympia, WA.; 2018.

120. Bennett MG, Lee SS, Schofield KA, Ridley CE, Washington BJ, Gibbs DA. Response of chlorophyll a to total nitrogen and total phosphorus concentrations in lotic ecosystems: a systematic review. Environ Evid. 2021;10: 1–25. doi:10.1186/s13750-021-00238-8

121. Taulbee WK, Cooper SD, Melack JM. Effects of nutrient enrichment on algal biomass across a natural light gradient. Arch Für Hydrobiol. 2005;164: 449–464. doi:10.1127/0003-9136/2005/0164-0449

122. Biggs BJF. Eutrophication of streams and rivers: dissolved nutrient-chlorophyll relationships for benthic algae. J North Am Benthol Soc. 2000;19: 17–31. doi:10.2307/1468279

123. Wootton JT. River food web response to large-scale riparian zone manipulations. PLOS ONE. 2012;7: e51839. doi:10.1371/journal.pone.0051839

124. Murphy ML. Forestry Impacts on Freshwater Habitat of Anadromous Salmonids in the Pacific Northwest and Alaska: requirements for protection and restoration. NOAA/ National Centers for Coastal Ocean Science; 1995. Available: Forestry Impacts on Freshwater Habitat of Anadromous Salmonids in the Pacific Northwest and Alaska: Requirements for Protection and Restoration

125. Groom JD, Johnson SL, Seeds JD, Ice GG. Evaluating Links Between Forest Harvest and Stream Temperature Threshold Exceedances: The Value of Spatial and Temporal Data. JAWRA J Am Water Resour Assoc. 2017;53: 761–773. doi:10.1111/1752-1688.12529

Supporting Information

S1 Table. Details of PLS, Wu jacknife, Wild Bootstrap models for headwater treatment groups. Box-Cox transformations were used with the goal of improving model quality. See S1 File for details on Box-Cox transformations. An exponent of 1 indicates that raw data were used. For normality tests, bold indicates that the null hypothesis of normal residuals was rejected. For Levene's test, bold indicates that the null hypothesis of a common variance across groups of a given factor was rejected. Significance was not reported in Table 2 when R^2CV was negative for PLS. For Wu Jackknife and Wild Bootstrap, bold indicates regression coefficients were significantly different than zero. We did not assume the regressor rows were independent and consequently used the Wild Bootstrap and Wu Jackknife for discerning model parameter significance. Interaction predictions are PLS model predictions transformed back to the original scale of the data. Dissolved organic carbon (DOC), Soluble reactive phosphorus (SRP), Dissolved organic nitrogen (DON), Dissolved inorganic nitrogen (DIN), Chlorophyll a (Chl a).

S2 Table. Details of PLS, Wu jackknife and Wild Bootstrap models for headwater watersheds. Box-Cox transformations were used with the goal of improving model quality. See S1 File for details on Box-Cox transformations. An exponent of 1 indicates that raw data were used. For normality tests, bold indicates that the null hypothesis of normal residuals was rejected. For Levene's test, bold indicates that the null hypothesis of a common variance across groups of a given factor was rejected. For Wu Jackknife and Wild Bootstrap, bold indicates regression coefficients were significantly different than zero. We did not assume the regressor rows were independent and consequently used the Wild Bootstrap and Wu Jackknife for discerning model parameter significance. Interaction predictions are PLS model predictions transformed back to the original scale of the data. Dissolved organic carbon (DOC), Soluble reactive phosphorus (SRP), Dissolved organic nitrogen (DON), Dissolved inorganic nitrogen (DIN), Chlorophyll a (Chl a).

S3 Table. Statistical summaries and details of PLS, Wu jackknife and Wild Bootstrap models for downstream sites. A) Kolmogorov-Smirnov two sample test p-values compare post- and pre- empirical cumulative distribution functions for downstream site groups (downstream of harvest and downstream of reference). **B)** Comparison of 95% Bias Corrected accelerated (BCa) Bootstrap confidence intervals (CI) for observed pre- and post-harvest treatment means for each response variable. "0" indicates the pre- and post-harvest CI overlapped; "Increase" indicates that the lower bound of the post-harvest CI was greater than the upper bound of the pre-harvest CI. "decrease" where pre-harvest was greater than post-harvest. **C)** Pre/Post comparisons of significance for PLS model predictions for each attribute, based on 95% Wild Bootstrap confidence intervals. When R^2CV was negative, significance was not reported, indicated by "- -" . Within a group, pre/post with the same letters are not significantly different: "A" denotes the largest value, "B" is second largest, etc, for that attribute. Box-Cox transformations were used

with the goal of improving model quality. For Wu Jackknife and Wild Bootstrap, bold indicates regression coefficients were significantly different than zero. We did not assume the regressor rows were independent and consequently used the Wild Bootstrap and Wu Jackknife for discerning model parameter significance. Dissolved organic carbon (DOC), Soluble reactive phosphorus (SRP), Dissolved organic nitrogen (DON), Dissolved inorganic nitrogen (DIN), Chlorophyll a (Chl a).

S4 Table. Concentrations of A) stream nitrate (NO^3) and B) ammonium (NH_4) at each site, each year at the time of early summer sampling. "--" indicates no sample collected. Additional Trask River Watershed study chemistry data are available through US Forest Service Research Data Archive: https://doi.org/10.2737/RDS-2022-0002

S1 File. Details of Box-Cox transformations.

S2 File. Epilithon biomass and chlorophyll *a* concentrations in June and July 2016.

PONE-D-22-25727
The paradox of stream nitrate enrichment and increased light yet no algal response following experimental manipulation of riparian zones and forested watersheds
PLOS ONE

Dear Dr. Johnson,

Thank you for submitting your manuscript to PLOS ONE. After careful consideration, we feel that it has merit but does not fully meet PLOS ONE's publication criteria as it currently stands. Therefore, we invite you to submit a revised version of the manuscript that addresses the points raised during the review process.
I encourage to carefully consider all the comments of the reviewers, especially those from reviewers 2 and 3.

Please submit your revised manuscript by Dec 12 2022 11:59PM. If you will need more time than this to complete your revisions, please reply to this message or contact the journal office at plosone@plos.org. When you're ready to submit your revision, log on to https://www.editorialmanager.com/pone/ and select the 'Submissions Needing Revision' folder to locate your manuscript file.

Please include the following items when submitting your revised manuscript:

- A rebuttal letter that responds to each point raised by the academic editor and reviewer(s). You should upload this letter as a separate file labeled 'Response to Reviewers'.
- A marked-up copy of your manuscript that highlights changes made to the original version. You should upload this as a separate file labeled 'Revised Manuscript with Track Changes'.
- An unmarked version of your revised paper without tracked changes. You should upload this as a separate file labeled 'Manuscript'.

If you would like to make changes to your financial disclosure, please include your updated statement in your cover letter. Guidelines for resubmitting your figure files are available below the reviewer comments at the end of this letter.

If applicable, we recommend that you deposit your laboratory protocols in protocols.io to enhance the reproducibility of your results. Protocols.io assigns your protocol its own identifier (DOI) so that it can be cited independently in the future. For instructions see: https://journals.plos.org/plosone/s/submission-guidelines#loc-laboratory-protocols. Additionally, PLOS ONE offers an option for publishing peer-reviewed Lab Protocol articles, which describe protocols hosted on protocols.io. Read more information on sharing protocols at https://plos.org/protocols?utm_medium=editorial-email&utm_source=authorletters&utm_campaign=protocols.

We look forward to receiving your revised manuscript.

Kind regards,

Clara Mendoza-Lera
Academic Editor
PLOS ONE

Journal Requirements:

When submitting your revision, we need you to address these additional requirements.

1. Please ensure that your manuscript meets PLOS ONE's style requirements, including those for file naming. The PLOS ONE style templates can be found at https://journals.plos.org/plosone/s/file?id=wjVg/PLOSOne_formatting_sample_main_body.pdf and https://journals.plos.org/plosone/s/file?id=ba62/PLOSOne_formatting_sample_title_authors_affiliations.pdf

***Response*: Corrected**

2. We note that the grant information you provided in the 'Funding Information' and 'Financial Disclosure' sections do not match. When you resubmit, please ensure that you provide the correct grant numbers for the awards you received for your study in the 'Funding Information' section.

***Response*: We revised Funding Information and Financial Disclosure. There were no grant numbers for the funding provided for this study**

- 3. We note that Figure 1 in your submission contain [map/satellite] images which may be copyrighted. All PLOS content is published under the Creative Commons Attribution License (CC BY 4.0), which means that the manuscript, images, and Supporting Information files will be freely available online, and any third party is permitted to access, download, copy, distribute, and use these materials in any way, even commercially, with proper attribution. For these reasons, we cannot publish previously copyrighted maps or satellite images created using proprietary data, such as Google software (Google Maps, Street View, and Earth). For more information, see our copyright guidelines: http://journals.plos.org/plosone/s/licenses-and-copyright.

  We require you to either (1) present written permission from the copyright holder to publish these figures specifically under the CC BY 4.0 license, or (2) remove the figures from your submission:

  a. You may seek permission from the original copyright holder of Figure 1 [#] to publish the

content specifically under the CC BY 4.0 license. We recommend that you contact the original copyright holder with the Content Permission Form (http://journals.plos.org/plosone/s/file?id=7c09/content-permission-form.pdf) and the following text:

"I request permission for the open-access journal PLOS ONE to publish XXX under the Creative Commons Attribution License (CCAL) CC BY 4.0 (http://creativecommons.org/licenses/by/4.0/). Please be aware that this license allows unrestricted use and distribution, even commercially, by third parties. Please reply and provide explicit written permission to publish XXX under a CC BY license and complete the attached form." Please upload the completed Content Permission Form or other proof of granted permissions as an "Other" file with your submission.

In the figure caption of the copyrighted figure, please include the following text: "Reprinted from [ref] under a CC BY license, with permission from [name of publisher], original copyright [original copyright year]."

***Response*: We removed any potential copyright question with our updated Fig 1. Our map was made by us using ArcGIS Pro with publicly available data layers of LiDAR terrain (https://www.oregongeology.org/lidar/) and with the hydrography layer derived from the LiDAR. The aerial image that was part B was removed. We had permission from Elsevier to cite this image which was previously published in our manuscript in Forest Ecology and Management, but Elsevier would only provide their signed copyright permission and was not able to sign your copyright permission form.**

.

---

5. Review Comments to the Author

Reviewer #1: This paper tests the response of algal biomass to different forest harvest, and riparian buffer practices. This study was conducted over ten-years and twelve streams, which is quite impressive and unusual. This work is interesting and high quality. While the work underling this manuscript is of good quality I have some questions about the statistics used to reach the conclusions of this work. Additionally, I think this manuscript could be more useful to other scientists and managers if it was shortened and simplified significantly. Please see my detailed comments below.

Major issues:

• Lines 257-263 and subsequent sections of results and discussion: This section describes the methods

used to measure extra samples in July of 2016 and compare the measurements of algae on rocks to algae on tiles. Because the goals of this are not mentioned in the introduction (e.g., you do not have hypotheses stated in the introduction) this feels out of place here – particularly the comparison of between rocks and tiles. From the discussion section I understand that part of the motivation for this was to understand whether you were sampling too early in the season to see an effect of the treatments – but I don't understand how the tiles fit into the goals of the present study. Additionally, the results from this don't seem to really shed light on the findings of the main part of the study. I suggest you move all of this material to an appendix, and briefly reference the findings in the discussion.
Response: **We removed the data from the sampling of tiles and our comparison of biomass on rocks in July is now in the Supplemental material. We briefly reference it in the discussion. We had included the tile comparison in year 4 post-harvest because we were surprised by not seeing any response in years 1-3. Tiles were added because many of the studies in the literature used tiles rather than sampling natural substrates and we had questions if our sampling of natural substrates might be influencing the lack of responses of algal biomass and chlorophyll *a* that we were observing. We added the July sampling to examine possible later summer responses, because the literature contains multiple examples where algal responses were studied only later in the summer.**

• Statistical methods: This paper uses multiple statistical methods to test the same questions, about how the forest harvest/ buffer treatments effects in-stream variables. The inclusion of multiple statistical methods add considerable length to the manuscript, and complexity to an experimental design that is quite simple. This manuscript would be much clearer if there was a single method used – and less statistical tests generally.
Response: **We think the three methods are a strength of the paper, especially, where consensus among the methods occurs. We acknowledge that their importance was not clear enough in the original manuscript, so we have modified the discussion and the introduction to clarify the purpose of doing multiple statistical tests. The three analyses were applied to the question of pre-post differences with the belief that consensus among two or more of the methods gives a stronger result than any one method by itself. The use of multiple methods is further warranted given the data challenges: spatial and temporal dependency, small sample sizes and the possibility of not finding an acceptable PLS model given the more stringent quality criterion using the R^2CV. The Kolmogorov-Smirnov 2 sample test (KS2) compares distributions of each pre-post pair. The BCa bootstrap calculates the 95% confidence intervals for the means, which can be compared for each pre-post pair. The PLS models are the only cases where all the data for a specific response are compared, and because of our stringent model acceptance criterion, not all PLS models were valid. We feel these three techniques provide value and are standard applications available in many statistical packages. PLS with Box-Cox transformations and wild bootstrap does add some complexity but allowed us to get better models and to deal with lack of independence in the data.**

• Line 307: Your hypotheses are all related to the effect of forest treatment across the watersheds which are essentially replicates. I don't understand the need to look at changes in each watershed individually as you describe here.
Response: **Although we started with a study design where watersheds within a treatment were planned to be replicates, the study sites within a treatment group showed variability in site conditions and responses and we were interested in exploring that variability. In addition, 'real world'**

implementation of treatments was not uniform, especially where no buffers were required (variable buffer sites). This led us to include examination of responses within each watershed. The evaluation of treatment categories is important for evaluation of forest plans and forest practices act, while the evaluation of individual watersheds addressed the variability observed in the forests and streams.

• Line 437-439: Describes correlations between chlorophyll-a and light, I don't see where these regressions are described in the methods.

Response: **Thank you for pointing this out. We now include details about the correlations and statistics for them.**

• Figures: The goal of this manuscript is to investigate the instream responses to forest and riparian zone management. In the context of this, each stream is essentially a replicate – and I think it would make more sense to present them this way in the figures. Many of the figures show multiple bars for each of the streams. Presenting bar graphs grouped by treatments would fit better with your experimental design and would give the readers much less extraneous information. The figures in the present version could be moved to an appendix if you would like to keep that information available to a reader.

Response: **The bars in the figures are consistently displayed and grouped by treatment so that readers can best see the variability of responses and which sites might be contributing to the treatment group response, rather than only presenting treatment averages. To help readers see the treatment groups, we used a specific fill pattern consistently for each treatment group. As you note below, watersheds are not true replicates, so presenting details by individual watershed can help readers understand what was influencing the observed responses.**

• Line 312-325. You describe the use of Wu's jackknife to account for non-independence of measurements. I am more familiar with non-independence being addressed with random effects in mixed-effects models (e.g., as described in the books by Zuur 2009, or Wu 2009), than through bootstrapping. Reading Wu 1986 they seems to specifically caution against using their method in situations with non-independence. From Wu; "Our analysis of the bootstrap …. encounters similar difficulties in handling complex survey samples, where neither the independence nor the exchangeability assumption is satisfied (Rao and Wu (1985a))." From my own exploration of your data it does appear that there is non-independence within dates and sites, with roughly half of the variation in the chlorophyll-a data explained by random effects for date and site. Please clarify if, and how the bootstrapping addresses these forms of non-independence in your data.

Response: **Wu (1986) proposed a weighted bootstrap which did very well in the tests given the Wu's Table 1, especially, compared to the other (unweighted) bootstrap. However, Liu (1988) improved on Wu's weighted bootstrap with what became known as the wild bootstrap. Other researchers made further improvements and/or proved additional results, especially for non-independence. See, for example, the Davidson & Flachaire reference for the wild bootstrap. (R. Liu (1988), Bootstrap Procedures Under Some Non-I.I.D. Models, Annals of Statistics, 16, 4, 1696-1708).**

• Lines 484-496: This whole paragraph could be cut. I suspect any reader of this paper will understand that watersheds are not true replicates, and that environmental conditions are variable.

Response: **Although readers understand that watersheds are not true replicates, many statistical analyses assume that the sites are replicates. We feel that a contribution of this paper, in addition to documenting responses to riparian harvest, is to suggest that multiple analyses could provide more**

solid foundation of statistical responses, given the multiple assumptions of statistical designs and that watersheds are not replicates and data are not normally distributed.

• Line 581-589: This paragraph seems very tangential to the stated goals of this paper and could be cut entirely or moved to an appendix.

Response: **As suggested, we removed tile comparison and this paragraph.**

• Line 612: The subheading for this section is "conclusions and management implications". The management implications are not clear in the text of this section, or elsewhere in the paper. Please clarify what a manager should do with the information presented in this study.

Response: **We have rephrased this paragraph to clarify what a manager should do with the information presented in the study.**

• Line 617-620: I am not convinced that doing multiple statistical tests of the same data provides better information or more certainty for managers and policy makers. At the very least including this extra information makes the paper longer, and harder for a reader to understand and I don't think that the added value is really there. I think the paper would be better with a single type of analysis or with additional analyses relegated to the supplement.

Response: **We revised these lines to more fully explain our reasoning for presenting multiple statistical tests. Please see above for our responses to the prior similar suggestion.**

Minor issues:

• Line 1: The use of the phrase "The paradox" doesn't feel like it is lived up to by the body of the manuscript. I think the phrase sets an expectation that 1.) This is a commonly observed phenomena, and 2.) You provide evidence that explains how this happens. I suggest a simpler title: "No in-stream algal response to forest and riparian zone management despite increases in light and nitrate"

Response: **Thank you for this thoughtful comment. We removed "paradox" from the manuscript including the title.**

• Line 39-41: I don't really understand why the community being dominated by diatoms would explain the results.

Response: **Sorry that it wasn't clear. We revised the sentence to include that green algae were not abundant.**

• Line 41: Is the word "dynamics" needed here?

Response: **No, it isn't and the sentence was revised.**

• Line 59: The topic sentence suggests that this is a list of ecosystem services provided by riparian zones. Ecosystem services are the benefits to humans provided by an ecosystem. Much of this list is benefits of riparian zones to streams which are only loosely tied back to humans in the last sentence of this paragraph. I suggest dropping the phrase "ecosystem services" from this paragraph.

Response: **Thank you for this suggestion. "Ecosystem services" were dropped and replaced by "ecosystem functions".**

• Line 31: You use the word "paradigm" in the abstract to describe the expected increase in nutrients, light, and shift towards autotrophy. However, on lines 79-80, and lines 87-89 you list numerous studies have found contrary findings. If there are already more than half a dozen studies that contradicted what you describe as the current "paradigm" I am not sure it is correct to describe it as a paradigm at all.

Response: **That is an interesting philosophical question about terminology. I wonder if we are using different definitions of paradigm? We looked up multiple definitions of paradigm and those definitions included: 'a standard perspective or set of ideas; a pattern, especially an outstandingly clear or typical example or archetype'. In our field, I would say that this shift to autotrophy is being taught as a typical example (or paradigm), even though there are some published examples that contradict. Especially in our work with natural resource managers and teachers, we see it being used as an assumption and it shows up in classrooms, forest management plans and many gray literature documents. However, we accept your concern and have rephrased the title and revised the Abstract and Introduction to change how we mention paradigm.**

• Line 96: I am not sure what the phrase "dueling statistics" means.

Response: **We are referring to the back and forth that occurring in the literature when authors rebut publications by other authors using differing analyses to yield differing results, followed by responses by original authors justifying the original analyses. There are multiple instances of this in literature around topics that have implications for resource management, such as the effects of logging on peak stream flows. Sorry for the jargon. We have revised these sentences and removed "dueling statistics".**

• Line 104-117: The formatting of questions and hypotheses could be streamlined.

Response: **Thank you for this suggestion. This section was streamlined.**

• Line 312: When you say "various measurements" in this sentence I don't know if you mean measurements of different parameters within the same stream and date, or a single parameter among dates and streams.

Response: **Thank you. We revised the sentence and clarified that sites were not independent.**

• Lines 485-486: I think there is an extra word in this sentence.

Response: **Corrected**

• Line 502-504: Including self-citation of unpublished work is not necessary to make this point.

Response: **These citations point to articles in the journal titled "*Forest Ecology and Management*" and are included here to support the statement about multiple trophic levels being studied at these same sites. We have included wording to make this connection more clear.**

• Line 499-502: Forest harvest increased nitrogen concentrations in both nitrogen limited and non-nitrogen limited ecosystems. If the response is the same why draw a distinction between nitrogen limited and non-limited?

Response: **Revised to remove this distinction**

• Line 519-522: This should be modified slightly. The buffers could have taken up some nitrogen. Perhaps modify this sentence to say "Although not all transport of inorganic nitrogen…"

Response: **Revised as suggested**

• When working with your data I had to refer back to the manuscript to figure out which sites had which treatment. It would be helpful if there were columns indicating treatments, and treatment periods in the data.

Response: **The archived data in the USFS Research Data Archive are formatted as you describe. Tables 1, 3, and 5 had this information and it has been added to Table S4. Table 4 is now a figure.**

Reviewer #2: The manuscript addresses a relevant issue about limiting primary productivity in headwater streams, often limited by light, facing deforestation. It has an exciting approach, including experimental deforestation and long-term monitoring, potentially responding to relevant ecological questions, and dissertating about environmental management.

I noticed a potential issue concerning the work's message that seems dissonant with the research findings—for example, assessing the standing stock of biomass or pigments is not a direct measurement of primary productivity. Net primary production is strictly defined as the difference between the energy fixed by autotrophs and their respiration, and it is most commonly equated to increments in biomass per unit of surface and time, e.g., g C m-2 d-1 (https://www.sciencedirect.com/topics/earth-and-planetary-sciences/net-primary-production).

- Could authors calculate NPP based on epileptic mass over time?
  Response: **We agree that reporting changes of biomass over time could be a valuable way to synthesize and inform changes in productivity. We acknowledge that algal stocks are not a proxy for productivity and we make this point more clear in text. Since we only have one measurement of biomass per year at each site, we are not able to calculate NPP based on changes in biomass per time. In streams, NPP is calculated from measurements of stream metabolism and respiration (*Methods in Stream Ecology*, Hauer FR, Lamberti GA, editors. Boston: Academic Press; 2017. doi:10.1016/B978-0-12-416558-8.00011-1) and is a very different metric than measurement of epilithic biomass or chlorophyll *a*. Although we do have data on in-stream metabolism (of which primary production is one component) and nutrient spiraling from the Trask River Watershed Study at a subset of the sampling times, we feel that the descriptions of metabolism, respiration, and nutrient spiraling and uptake rates are complex, lengthy subjects better suited to a separate publication.**
- NPP may not be associated with biomass or pigments. For example, epilithon/periphyton is a matrix formed by a stock of autotrophic and heterotrophic biomass. Whether the heterotrophic compartment decrease and the autotrophic one increases, the balance is no biomass gain, and other factors may influence autotrophic and heterotrophic proportion (Guariento et al., 2011, https://link.springer.com/article/10.1007/s10452-011-9377-5). In addition, it is known that primary producers can increase or decrease their cell proportion of pigments depending on light conditions (Ma et al., 2022, https://doi.org/10.1016/j.biortech.2022.126777).
  Response: **We agree and more fully describe epilithon as a matrix. We present chlorophyll *a* concentration to evaluate activity and greenness. We also note that autotrophic algal taxa did not become more common after harvest.**
- It is worth mentioning that monitoring data is precious, including the immediate and long-term impacts, and this research could show interesting time series. The way authors present 10 years of data pulled in before and after deforestation, using repeated measurements and ignoring system dynamics.
  Response: **Yes, it is challenging to conduct large scale studies over time. While deciding what to include in this paper, we explored year to year and variability of responses as a function of**

time since treatment. Only DIN showed some lags in responses and therefore we presented data by site and year in Table S4 for the two forms of dissolved inorganic nitrogen. However, with regard to time series we believe there are too little data for this approach. Our analyses provide validation statistics which we feel makes our results more robust; we added a reference that highlights the importance of cross-validation or test set validation (Breiman, L. 2001. Statistical Modeling: The Two Cultures, Statistical Science, 16, 3, 199-231), in addition to the existing Efron reference.

- In addition, I believe that the manuscript should be restructured textually. For example, part of the assumptions is in the methods, and part of the methods is in the introduction. I find in the scientific literature a structure in which the introduction takes the scientific question and presents the hypotheses and the expected results.
  Response: **We agree that what you describe is optimal and we present our scientific questions and hypotheses in the Introduction. We double checked that methods are not in the Introduction. We mention some assumptions in the Data Analysis section because those are required to explain how we selected our analyses.**
- I also suggest including a sub-item about management and relocating textual sections accordingly.
  Response: **We have revised our statements about managers potential use of these findings, based on Reviewer #1's comments.**
- Similarly to the first comment, it is unclear what processes were changed or not during the removal of the forest. What ecological processes and functions lead to changes and maintenance in the system? Is there evidence for what limitations of local N and P? Does species composition show any exciting pattern?
  Response: **We feel this paper provides foundational information about instream responses to forest harvest and clearly shows what changed and what did not change. We are not attempting to quantify the ecosystem processes here and we do not present primary production results. The rates and processes can be informative but in different ways than what we focus on in this manuscript. We also provide the taxonomic information for the most common species of diatoms and note that there were not substantial changes.**
- Finally, I believe the authors can tailor their questions to the results and respond to other ecological questions not addressed in this manuscript, such as biomass and species dynamics.
  Response: **We have revised the manuscript extensively, based on all three reviewer comments. We discuss epilithon biomass and algal species dynamics. Beyond that, I am not sure how we might "tailor questions to the results"? Or how or why we might "respond to ecological questions not addressed in this manuscript"? We revised our study questions and hypotheses in the Introduction and now they are more streamlined, as recommended, we fully discuss our finding related to these hypotheses in the Discussion section. We more fully address forest harvest effects on biomass and species dynamics for epilithon as well as responses of nutrients and carbon. Thank you for suggestions.**

Reviewer #3: Thank you for giving me the opportunity to review this paper submitted to PLOS One. I think that the dataset analyzed in this paper has many strengths, including the longevity of the research with multiple years of pre-harvest controls, numerous watershed pairings, consideration of spatial complexities when studying stream ecosystems, extensive complementary datasets from a multi-disciplinary team, consistent annual sampling design, and algal composition data reviewed by a co-author who leads the field in diatom identification. While I could not speak specifically to the nuances of the partial least squares regression method due to my lack of familiarity with this type of analysis, I recognize the strength of using multiple non-parametric statistical methods to analyze field data characterized by a high degree of natural variability. Consequently, these data offer an important contribution to our understanding of how forest management affects in-stream primary producers and I sincerely hope that these data are published. However, I did feel that there were numerous and substantial issues with the paper as written that lead me to recommend that this paper be categorized as needing "major revisions."

- My concerns with the paper tend to fall into three categories. First, while there were many strengths in the methodologies used in this study, I do not feel that the authors provided enough detail on the specifics of how algae were collected from the stream in the methods and did not do enough to recognize the limitations of some of the methods that they used in the discussion.
  Response: **We now more fully describe the sampling protocol for attached epilithon in the Methods, as you suggest. In the Discussion, we added text about the limitations of algae sampling so that readers would recognize that epilithon is of mixed origin, including autotrophic and heterotrophic materials, and spatially variable -hence the number of rocks that were sampled for each composite sample with multiple composite samples per reach.**
- Second, while many of the figures were relatively visually clear, I found some inconsistencies with labeling to make it difficult to compare among figures. There was also a substantial amount of information that I felt was missing from the figure legends pertaining to the statistical analyses. I also disagreed with the use of standard deviations for error bars when the authors calculated 95% confidence intervals as part of their statistical analyses (and are more easily interpreted for statistical relevance in bar graphs).
  Response: **Thank you for pointing out the inconsistencies. We clarified legends, regrouped panels and added figures as suggested (below). It would be nice to be able to incorporate the lettering of significance for watershed comparisons in each figure. However, we could not figure out how to include visual notation about significant differences at the treatment scale since what we are presenting are individual sites and because we have multiple significant tests. And the confidence intervals we calculated as part of the statistical analyses are for treatment groups. For watersheds, we feel that the variability in data for the pre and post-harvest periods is best shown as standard deviation. If we were to show standard error, we would be presenting the error around the means, not of the data. Interestingly, given our small sample sizes (n=4 or 6 years pre and post), the error bars for the standard deviation are almost identical to 95% confidence intervals because confidence intervals are calculated as**

standard error * 1.96. We added statements to the text that the current error bars are almost identical to 95% confidence intervals.

- Finally, I felt that there were some substantial issues with the writing, particularly related to the framing of the data analysis and the literature used to support their findings. In particular, there were many areas of the writing that were too broad and drew from a broader literature source; focusing more specifically on riparian studies in headwater streams would allow the authors to make more direct comparisons and strengthen their claims.
  Response: **We appreciate your suggestions. We revised our framing of data analyses and narrowed the focus in the Introduction and Discussion to more specifically focus on harvesting and riparian studies.**

<u>Specific Reviewer Comments</u>

- 34: This description does not seem to account for the "thinned" treatment – is there a better way to write a succinct description of the treatments to make it clearer?
  Response: **Yes, good point and we revised to try to succinctly include treatment descriptions in the Abstract**
- 39: I am not sure you discuss the importance of the algal community enough to include it as a major point in your abstract as written. I would like to see you spend more time talking about the algal species composition in the paper to justify it here.
  Response: **As you suggest, we revised the sections about algal species composition within the paper and also reduced discussion of it in the Abstract.**
- 49: This may be more of a stylistic comment, but I am not sure I like the use of sub-titles within the introduction in a non-review style paper
  Response: **Subtitles were included to try to summarize main points of Introduction and Discussion. We feel they may be helpful to readers or we can remove them. We will defer to the Editor's recommendations for this.**
- 53: I think your argument about the importance of studying harvest would be strengthened if you could provide some kind of estimate of the percent of forests harvested in your study region, the US or globally
  Response: **Good suggestion. We now include statements about the amount of forested lands across the US and percentage of forests where harvesting can occur to provide context for readers.**
- 54: This statement would also be strengthen with some citations about both the diversity of aquatic organisms and the use of headwater streams as upstream sources of water for downstream communities
  Response: **Yes, thank you for that suggestion. We added citations about aquatic biodiversity diversity and domestic use of headwater streams for drinking water.**
- 59: I think it would be helpful to have a more direct tie from the effects of harvest to the use of riparian buffers as a management strategy
  Response: **We added text about functions of riparian buffers and then we provide details in the paragraphs addressing management of riparian forests relative to these functions.**

- 62: Rather than saying "minimize algal blooms," it seems this sentence should focus on the effect on light directly
  Response: **This sentence was revised as suggested.**
- 64: This part of the sentence is a little unclear: "Changes to any or all of these functions and ecosystem services" – Changes due to what? Functions and ecosystem services provided by forested riparian areas?
  Response: **Thank you for pointing out that this sentence was not clear. We revised it to and addressed ecosystem processes rather than services.**
- 70: I found this sentence to be very unclear – I was not sure what exactly you meant by "lower trophic levels of food webs" – Is this stream food webs or forest food webs? Is it primary producers or are herbivores also included? Is the focus here on removal of riparian vegetation or forest management at large? Because this is the first sentence in the paragraph, I am having a hard time figuring out the exact direction that this paragraph is going.
  Response: **We revised this sentence and narrowed the topic for this paragraph. We are focusing on aquatic ecosystems and responses to removal of riparian vegetation associated with forest harvest.**
- 73: You talk about the functions of the riparian buffer, but think it would be helpful in the paragraph above to talk about how the function of the riparian buffer vary with width – then this sentence will make more sense
  Response: **Good suggestion; revisions were included in previous paragraph.**
- 75: when you talk about stream parameter or process is of most interest, are you referring to protection of these processes as part of the establishment of riparian buffers?
  Response: **Yes, we revised as suggested.**
- 75: The focus on nitrogen in this paragraph seems to have not been a logically led in by the initial sentences in this paragraph. It might be helpful to start with this sentence, and then take the information from the first two sentences to make them more specific to nitrogen, since this seems to be the focus
  Response: **Good suggestion, we revised this paragraph to start broadly and then identify nutrient and sediment responses. (Nitrogen is not the only focus). Algal responses have been moved to their own paragraph.**
- 77: I would remove the information about fire-related disturbances, since you have narrowed to forest harvest in the first part of your intro
  Response: **We removed references pertaining to riparian disturbance by fire in the text.**
- 79: Again, I think focusing just on the effect of harvesting on nitrogen, rather than on "forest disturbance" would be more focused here
  Response: **We clarified that we are focusing on forest harvest and that we are studying responses of nutrients (N and P) as well as DOC, not just nitrogen.**
- 80: Will the general readership of PLOS one know the term "epilithon" or should it be defined?
  Response: **Good point; sentence was revised and algal biomass is used in the Introduction. We define epilithic algae/epilithon in the Methods and come back to the definition in the Discussion**.

- 80: Here is another place that I feel the writing is disorganized – the preceding sentences were about nitrogen, but then we jump to epilithon. It seems that this whole paragraph could be more focused by having a general subject of thinking about the effect of riparian buffers on abiotic conditions in streams, and then having a separate paragraph that talks bout the connection to the biotic
  Response: **Thank you, we revised and reorganized these paragraphs**
- 82: It seems like you should not include natural gaps in the canopy cover – it takes away the focus on riparian buffers to protect from harvest
  Response: **We removed natural gaps.**
- 83: It might make sense to first start by talking about the expected effects of nitrogen and light on biomass of epilithon, then get into the taxonomic effects
  Response: **We now start this paragraph referencing increased biomass.**
- 85: I have a problem with starting the sentence by saying that light changes composition of algae in the stream, but then supporting that with clearcutting (because there are many other abiotic factors that are affected by clearcutting)
  Response: **We revised this section to include nutrients and light, as supported by the citations.**
- 87: Can you be more specific on the ways that diatoms and filamentous algae affect consumers? Talking about which group is more palatable and/or which consumers benefit or don't benefit?
  Response: **Because we are being encouraged to shorten this paper, we don't feel that we should go into those details, which are off topic for this study. We include this sentence about food web responses to help readers have context that the type of algae matters.**
- 89: I think that the last sentence about not supporting the common paradigm is a bit bold, and think maybe there should be more support provided to support this
  Response: **We revised the Introduction and now mention paradigm as part of a citation about null findings being less frequently published.**
- 90: What do you mean by "this complexity of responses"?
  Response: **We added clarification that we are referring to multiple instream responses to forest harvest.**
- 91: I wonder if the issue is not so much the focus of resource managers and research on statistically significant findings, but rather the inflexibility of commonly used statistical tests to capture biological variability and significance in dynamic systems like streams
  Response: **Thank you for suggesting a more clear way to say this.**
- 95: I think that you need to be a little clearer than stating "may not be supported" – Do you mean that the assumptions might not be met? That the methods might not be appropriate?
  Response: **Yes, good suggestions. We revised as suggested.**
- 96: Can you be more specific in what you mean by "dueling statistics" Do you mean using multiple statistical approaches?
  Response: **Yes, we are referring to the back and forth that occurring in the literature when authors rebut publications by other authors using differing analyses to yield differing results, followed by responses by original authors justifying the original analyses. There are multiple**

instances of this in literature around topics that have implications for resource management, such as the effects of logging on peak stream flows. Sorry for the jargon.

- 96: I think you lost me on this final sentence – it seems to relate more to how managers manage riparian vegetation, rather than its relationship to the discussion about statistics
  Response: **We revised this sentence and this section so that the paragraph addresses statistics more directly and not how managers deal with riparian vegetation. Thank you.**
- 101: I am not sure what you mean by "extent" of riparian buffers – do you mean width? Length? Quality?
  Response: **We removed 'extent' and replaced it with width.**
- 101-104: This sentence is a little long, and might benefit from being shortened or turned into two sentences. Also, are you just measuring standing stock of algae or composition as well? If not measuring composition, I think that information can be removed from above.
  Response: **We are evaluating community composition as well as standing stock of algae. We simplified this sentence.**
- 104: Again, because you don't specify what you mean by "extent," in the first sentence, it is hard to know what you mean by "more removal" of riparian vegetation
  Response: **We revised and clarified this sentence**
- 105: I think it would be better to separate the effect on light and the effect on algal biomass
  Response: **We think of these as related. It is generally well accepted that removal of riparian vegetation changes light to a stream and what we are exploring in this paper are the related responses - or not- of epilithon.**
- 106: I would rephrase this part of the hypothesis to start with the effect you expect on nitrogen and then say why
  Response: **We revised all the hypotheses as you suggest so that they all have similar structure.**
- 108: I am a little confused here because you talk about nutrients and dissolved organic carbon, but the intro really just focuses on nitrogen. Which do you measure?
  Response: **We measure nutrients and DOC in the streams. We added mention of responses of dissolved carbon to forest harvest in the Introduction.**
- 108: You don't really discuss sediment at all in the intro, which I think is problematic to bring it in here in the hypothesis
  Response: **We now have expanded discussion of sediment responses in the Introduction.**
- 111: Is this dissolved carbon or are you measuring other forms of carbon? I also think you could change this to be one sentence by saying "Do forested riparian buffers reduce increases in in-stream concentrations of dissolved organic carbon and nutrients in harvested forest watersheds?"
  Response: **Thank you, we clarified that we are studying DOC. And because we simplified this section, we no longer have the question format.**
- 114: This question also does not line up with anything in the introduction or in the hypotheses, because you don't mention temporal or spatial extent anywhere else.

***Response:* As suggested by previous reviewer, we removed the questions to simplify this final section of the Introduction**

- 116: Should this say “chlorophyll a”?
  ***Response:* Yes, corrected.**
- I also don’t think you can bring in light here if you don’t mention light as something that you are directly measuring as a question.
  ***Response:* This section of questions were deleted.**
- 120: Should there be a reference to a figure at the end of this sentence?
  ***Response:* Reference to Fig 1 added**
- 128: Rather than saying “prior disturbances, including…” I think you should just say “disturbance due to wildfire” because you don’t give examples of any other types of disturbances
  ***Response:* We removed ‘including’. The next sentences note additional disturbances from forest harvest and replanting and roads.**
- 129: I think it would be helpful to know the extent of this burning and logging within the watershed – ideally a map, but even just report of percentages would be helpful.
  ***Response:* We edited this slightly. The extent of burning and logging was 100%, hence the vegetation was 45-60 yr old at the start of the study.**
- 136: did you evaluate downstream effects in this study, or was this just an objective of the overall study?
  ***Response:* The study of headwater and downstream responses occurred across all of the Trask River Watershed studies (stream temperature, discharge, sediment, nutrients, algae, stream metabolism, insects, instream vertebrates)**.
- 138: Were there 12 treatment and 12 reference or 6 treatment and 6 reference streams?
  ***Response:* We revised this to remove confusion.**
- 140: Were there riparian buffers left in these? Since this is a key point in the study, it seems like it would be good to mention it here
  ***Response:* The next paragraphs go into extensive detail about where riparian buffers were retained.**
- 146: I found it really difficult to read the legend on your figure. I am also not if you need both the satellite image and the map here. I also think it is problematic to introduce the reader to your treatment types for the first time in the Figure legend. I don’t know what you mean by “variable buffers’ or “uniform buffers” – a more specific description of the buffers that were left and the harvesting strategy would be helpful. Also in your figure legend, it would be helpful to give context for the 2013 year (first year post-harvest could be added).
  ***Response:* We revised this figure and the legends should be clearer in the new upload. We provided context for why the photo from 2013. This figure serves multiple purposes and the paragraphs following the figure go into extensive detail about our study design. Because this figure serves to show the location of the study watershed in western US, we cite it at the beginning of Site Description, but we could remove the reference to Fig 1 from the beginning of the Study Description? However, wherever it is first cited, there will be the issue of more descriptions following the presentation of the figure.**

- Table 1: I think it would look better in the harvest treatment to remove the underscore from the description. Do you think it is necessary to state the ownership of the watershed? You might instead just want to indicate if it was private or public. Also, is it possible to rename your watersheds so that all of the references are 1 and the clearcuts are the remaining numbers? I know this might not be possible if you are working in a large group. Did you run any statistics to compare the differences among your reference and treatment watersheds to make sure they were similar in some of the physical characteristics? Also, what is a "15m no riparian buffer?"
  Response: **We revised this table and all others to remove the underscore in the treatment names. We include the missing word in the description of the thinned watershed. We changed the ownership columns also – thank you for that suggestion. We do not want to renumber the watersheds because these are the names being used across all of our Trask publications. We did not run statistics in advance of the decision for which watersheds would be harvested and which would be reference; these decisions were made at the beginning of the study and based on harvest guidelines, adjacent treatments, and with input from our research and management group. However, in our PLS analyses, the pre-post predicted responses can be compared within a given watershed as well as compared across all the watersheds for each response variable. Therefore one can compare the post-hoc lettering across watersheds and identify which watersheds have unique lettering and therefore are significantly different.**
- 151: I am a little unclear as to whether these guidelines apply to the private or public lands or both
  Response: **The guidelines differ for state and private forests and we clarified this in the text.**
- 153: Can you provide a definition of "leave trees" in the prior sentence by using this term so that it is more familiar when you state what happened in the UM2 and UM3 watersheds
  Response: **We removed the term leave trees and further explained that these were trees identified for wildlife and required to be left unharvested.**
- 156-158: A few points of clarification about this treatment are needed – the first is how "perennial stream" is being defined by the logging company and if that is consistent with aquatic ecologist definition of the streams in this area. It is also unclear how even the 12m of buffer were in this treatment – were they really even enough to be considered "uniform buffers"?
  Response: **Perennial streams are defined as having flowing surface water all year long. This is a consistent designation across forest ownerships. The uniform buffers are termed that because they were amazingly consistent ribbons of trees around the streams on state lands. This can be seen on the aerial imagery in Fig 1 or in greater detail in the supplemental material with a prior paper: 10.1016/j.foreco.2021.119999.**
- 161: It would be helpful to start this sentence by stating if this watershed is a treatment watershed.
  Response: **Sentenced was revised as suggested.**
- 167: I feel like this sentence is written as if you have not just described the watersheds, rather than stating that these were within the watersheds you just described.
  Response: **This sentence was removed and the paragraph edited to be clearer.**

- 167: I do feel I need to question whether 30m is a long enough reach to make these measurements
  Response: **These reach lengths were selected based on EPA sampling strategy of the study reach being 11 times the width.**
- 189: Do these streams have any crayfish that could influence algae?
  Response: **We initially were interested in studying crayfish as part of this broader study, but only a few crayfish were able to be located during the pre-harvest sampling across all streams. No juga snails were present either.**
- 191: I don't think you need to list the physical characteristics that you measure, because you then go on to describe each of them.
  Response: **The list was deleted.**
- 193: Is this an n=4 measurements in a 30 m reach?
  Response: **Yes, we clarified that measurements were taken at transects and that transects for densiometer and other metrics were evenly spaced through the reach.**
- 200: I am assuming based on the sampling time selected in June that there was complete canopy closure at this time for deciduous trees, but I think it would be helpful to clarify this, particularly for the higher elevation sites
  Response: **Yes, the sampling in middle to late June occurred with full leaf out. The high elevation sites in the Oregon Coast Range are not very high - leaf out occurs generally in April.**
- 206: Did the downstream measurements go upstream of the site or downstream of the site?
  Response: **We clarified that we sampled at the most downstream section of each site. Site characteristics were measured upstream from the sampling locations.**
- 206: Do you ever address the hemispherical photo data later in the results or compare it with the densiometer data? I don't remember seeing that information.
  Response: **We graph the hemispherical data in Fig 2 because it showed clear characterization of changes in light at the streams pre- and post-harvest, which was relevant to algal dynamics. Hemispherical data are much more intensive to collect than densiometer data and therefore we only have hemiview data from 2008 and 2013. Densiometer data, which quantify canopy closure, were collected at each sampling time and location and the reach averages were analyzed for changes pre and post.**
- 211: I am not sure what you mean by "percent total site factor"
  Response: **That is a metric within the hemiview software that land managers frequently use and we revised the text to clarify this.**
- 212: Did you take all images on a cloudless day to enable the digital image analyzer, or does this analysis program allow you to account for things like cloud cover?
  Response: **We took all images at dawn or dusk and on cloudless days, which are common in summertime in western Oregon.**
- 213: This last sentence feels very out of place after a whole paragraph about canopy cover
  Response: **Yes it seemed out of place and we moved this sentence to earlier in the paragraph.**

- 222-226: I think it is worth mentioning here again that if you are focusing on nitrogen, phosphorus, and dissolved organic carbon as drivers, these all should be mentioned somewhere in your introduction
  Response: **We revised the Introduction to include discussion of all these analytes.**
- 229: I would like to see a little more description made about how the algae was collected   How were the site for algae collection selected within the stream reach? Why did the number of rocks vary per site? Were they in pools, riffles? Were they collected at the same location as the sediments? Did you calculate the area of the rocks that you used? What method did you use to calculate rock area?
  Response: **We revised the text to clarify our algal sampling protocol, including that rocks were randomly selected, that templates were used to limit the area scrubbed and that the total area scrubbed per composited sample was noted. These small streams are not easy to classify as distinct riffles or pools. Sediment samples were collected randomly within the same reach but not where rocks has been disturbed.**
- 242: I think it would be helpful if you clarified where these algal structure data were collected relative to the other samples for biomass
  Response: **We clarified that these data were randomly collected.**
- 245: I am wondering if it is problematic that your only taxonomic analysis occurred in the first year post-harvest and not in subsequent years as well give how much disturbance there likely was in the first year.
  Response: **While we would have liked to have the taxonomic information for each year, these analyses were expensive and time consuming. If there was a impact of the harvest, we would have expected to see it the first year, give the amount of hillslope disturbance.**
- 254: I think that you should cite all of the resources used for the diatom analyses, rather than as an "e.g."
  Response: **Corrected as suggested**.
- 257: I think that it would be better to put this additional sampling up by the section where you talk about biomass since it doesn't sound like you measured algal composition on these additional samples
  Response: **This section was moved as suggested.**
- 277: I think that you should not use KS2, but either write out the full name of the test or summarize what you are doing (or take out subtitle)
  Response: **We removed the abbreviation.**
- 279: I think you need a comma after "thinned" to distinguish it is a separate group from reference
  Response: **Added as suggested.**
- 298: I don't think that treatment group, harvest period, etc should be capitalized here
  Response: **Capitals removed.**
- 330: One question that I have is how you treated replicates within each of these statistical analyses – did you treat multiple samples from a stream as pseudoreplicates?

Response: **Pseudoreplication with field studies is a concern. Our multiple samples within a stream were composited for a site average per year. The annual data were then evaluated in PLS models, using non-parametric wild bootstrap and semi-parametric Wu jackknife for significance testing. These methods guard against the effects of heteroscedasticity and non-independent (non-i.i.d) data. We added 2 more references that might help clarify this: R. Liu. 1988. Bootstrap procedures under some non-I.I.D. models. Annals of Statistics 16: 1696-1708; Liu, R. Y. & Singh, K.1995. Using i.i.d. bootstrap inference for general non-i.i.d. models. Journal of Statistical Planning and Inference 43: 67-75.**

- 337: A question I now have looking at your discharge measurements – did you have any requirement for recent rain event when you did your sampling, or did you just sample regardless of flow conditions? This might be helpful to clarify in the methods
  Response: **We noted in the text that we had criteria to avoid rain events for our sampling. With year to year differences in overall weather, the flows varied at the time of sampling at the end of June.**
- 343: I think you should report the magnitude of the temperature increases in the text of the results section. Also, were these part of your statistical analyses?
  Response: **June temperatures increases post-harvest are now explicitly presented in Results. Temperatures were not included in our statistical analyses because summer temperature responses from the Trask River watershed treatments have been reported elsewhere (Reiter et al. 2020, Bladon et al 2018).**
- 345: If you are going to talk about temperature first, then I think this should be the first sub-panel in Figure 2, rather than the second (since you haven't yet referenced 2A).
  Response: **We also reversed the order of our discussion of temperature and light in the text.**
- Figure 2: I think this figure would be improved with better labeling. For example, for the treatments, it would be helpful if you had the width of the riparian buffer in parentheses next to the treatment name. Also, are the downstream sites here? If not, you might want to include them and/or specify this is only the headwater stream sites. I am also unclear as to why the light measurements do not have error bars. Didn't you take multiple measurements? Also, where is there a graph or table showing the difference in sediment among the sites? I think this would be very helpful to have graphically.
  Response: **We revised Fig 2 and included error bars. We duplicated the names of treatment groups within the figure, and included riparian widths for uniform and thinned sites. We could not include buffer widths for the variable treatment because they were not consistent Sediment data are included as a graph in new Fig 3.**
- Figure 2 caption: I think there are a number of issues with this caption. First, there is no indication of the sample size or statistics for each measure. Second, I personally prefer the use of standard error or confidence interval rather than standard deviation for comparing means. Didn't you calculate the 95% confidence intervals for each of these groups using the bootstrapping method? Couldn't you plot those here?
  Response: **We revised Fig 2 caption, clarified that these are only headwater sites. The sample sizes are detailed in the methods and differ for treatment groups or watersheds. For**

watersheds, we feel that the variability in data for the pre and post-harvest periods is best shown as standard deviation. If we were to show standard error, we would be presenting the error around the means, not of the data. And given our small sample sizes (n=4 or 6 years pre and post), the error bars for the standard deviation are almost identical to the 95% confidence intervals in our current bars if confidence intervals are calculated as standard error * 1.96. We added statements to the text that the current error bars are almost identical to 95% confidence intervals.

- Also, you say in the description for A that you calculated incoming radiation, but I don't think that is quite correct – usually radiation is measured using a light meter, but you just measured canopy coverage vs. no coverage.
  Response: **The light metric was calculated using industry standard software from our hemispherical images. Note that we are not reporting radiation but percent light. The annual canopy coverage data was collected using a densiometer and you are correct, that measure is only percent coverage or its inverse percent shade. We clarify this in the text.**
- Table 2: I think the bold text is a little hard to read – could you use an asterisk instead? I also found the symbols for the PLS model to be a little bit confusing.
  Response: **Asterisks were included for significance. The PLS model comparisons are not meant to be confusing; they show if the models were different where the lettering is unique before and after harvest (B/C). They also show the magnitude of the predicted model value across all treatments: A being largest value, C smaller, etc. So where the response was B/C, the values were smaller post-harvest.**
- Figure 3: I think it is important to make your labels consistent between Figure 2 and Figure 3.
  Response: **These have been revised. The collection period for the hemispherical data once pre and once post – and therefore the labels have to be different.**
- 365-374: I think that logically it makes more sense to talk about DOC, SRP, and DON in the same order in the text as they appear in the figure. I also think it is important to include your n somewhere in the text for reference.
  Response: **The order of discussing the panels is now sequential as suggested. We clarified that these samples are from annual sampling during early summer and the years are in the legends.**
- Figure 3 caption: I think it is also important for you to include more of your statistics in the figure caption as well, particularly the n, the p-value you used from one of your tests. Same comment about the use of 1 standard deviation here compared with the value of 95% CI or standard error.
  Response: **I know the type of figures you are suggesting – where lettering of significant differences are across the top of the bars. I tried this and while it could work for a single panel figure, many of our figures are multiple panels and the figures get too complicated. The lettering of significance has to be very small and its confusing with multiple panels. If we split our figures to be single panels, then we would have way too many figures. I included asterisks and hopefully they don't disappear in the complexity of images. We note in the caption that**

results of statistical tests for the headwater watersheds are in Table 3. See above for our response about type of error bars.

- 377-382: One question that I have is how these are varying from one year to the next post-harvest – is that what can explain the large variability in the error bars? I would predict that the DIN would initially be high, but then decrease as more vegetation grows in.
  Response: **We show each year's nitrate and ammonium concentrations in Table S4. Actually, nitrate appeared to increase with time since harvest, but because our sampling period covered only 4 yrs post harvest, we did not evaluate the significance of this trend. Vegetation recovery was limited over these 4 yrs, even though replanting of small conifer trees did occur in year 2 (as required by harvest regulations.) We do highlight and discuss the timing of max DIN in the text now.**
- Table 3: I think that you need to have a better description of epilithon here, as it does not indicate that what you are measuring is AFDM. I also think that generally, the information presented from the PLS would be better as a graph, rather than as a table, because you could visualize how different groups move from one to another. You couldn't do this for all variables, but maybe you put this table in your supplementary materials and make a graph with only the most interesting of the points you want to illustrate.
  Response: **We added 'biomass' to the column headings to clarify what are the metrics for epilithon. In the interest of space and presenting all the data, we feel that the table is the most concise presentation of the results. We added statistical results to the graphs where possible.**
- 394: You bring in the molar ratios of DIN: SRP here, but I think this is the first time that you talk about it – why might this be important to consider? If you think it is important, maybe highlight this point in your introduction.
  Response: **We revised to mention molar ratios in the Introduction.**
- Table 4: I am wondering if table 4 should be a fifth panel in the other panel about water quality – this is very hard to visualize looking at it in a table, and it seems that tables should be reserved for providing the output of statistical tests, rather than means and SD.
  Response: **We now present the molar ratios as part of figure 4.**
- Figure 4 – I think it would be helpful to indicate which downstream site is the reference and separate it out from the other three sites visually. Also, were each of these sites mixed in terms of the upstream harvesting, or could they also be grouped by the harvesting method?
  Response: **Now Figure 5. Good point. We now identify RK as the reference. Some sites were mixed in terms of harvest method, some were a single method. We include this information in the caption.**
- Figure 4 caption: This caption has the same issues as the other ones – there are no statistics provided or indication of n.
  Response: **Now figure 5 - The figure captions have been revised to be standard across the figures and the figure legends now clarify that what are presented are annual averages during early summer sampling, with the years noted. The statistics for downstream sites were conducted as treatment groups, not for individual sites, so including the statistics on a figure**

showing individual sites is rather complicated. Plus we have three different statistical tests to report.

- Figure 5: Same issue with consistent labeling with previous figures. Figure 5 caption: Same issues as prior captions

  Response: **Now figure 6. The figure captions have been revised to be standardized and figure legends clarified. We tried to include lettering of significant differences across the top of the bars and while it could work for a single panel figure. The lettering of significance has to be very small and its confusing with multiple panels. If we split our figures to be single panels, then we would have way too many figures. I included an asterisk (there is only one significant difference – its an Epilithon reference site). We note in the caption that significance presented is in Table 3**
- 437: I think it would be helpful if you moved this section with chlorophyll a correlations to a new paragraph, since it is referring to different analyses

  Response: **It is now a new paragraph.**
- 438: One thing that I think is a bit misleading here is that you correlate "light reaching the stream" with algal concentration – but the problem is that your canopy cover measurements were not taken at the same locations as your algae measurements. I think you need to clarify this point by saying "mean reach-scale canopy cover" to indicate that it is representative of the whole reach.

  Response: **Good point that these metrics are reach averages and we clarify this in the text.**
- Figure 7: I am not sure that I find the data presented here to be very convincing or helpful, and I think the tile study should be eliminated (or just mentioned briefly without a figure). It appears to me that the tiles were substantially lower, which suggests to me that either the incubation time was too low, or that there was an issue with the estimate of rock area potentially. It also is not as helpful as it could be because these measurements were only made once post-cutting, instead of clear cutting.

  Response: **Our comparison of biomass on tiles was removed. July samples of epilithon and chlorophyll a are now only in the Supplemental material and we briefly reference it in the discussion. We checked and the area of estimation was correctly calculated in the same manner as for all other estimates.**
- 445-449: It was a little unclear to me if the diatoms you discuss here were found pre-harvest, post-harvest, or both – I am particularly confused by the line starting on 448. It seems to me that this part of the results deserves much more attention and description. Also, rather than just stating the most common taxa, it seems like you should do some kind of indicator species analysis to have a better way of making a cutoff of important taxa. Also, could you give some indication of the type of diatoms these are in terms of their growth form in the table, and then use this as a basis of discussion in the discussion?

  Response: **We revised the sentence to clarify that diatoms were most common algae in the pre- and post-harvest periods and added additional information about growth forms of the dominant diatoms in the Methods, Results, Table, and Discussion. This algal taxa dataset is too small for meaningful ISA analysis.**

- 453: I am a little confused by the line that starts here because you say "they" and I am not sure to what group this refers.
  ***Response:* Sorry for the confusion. We revised and clarified this section.**
- Figure 8: there is the same issue with labeling and captioning here.
  ***Response:* Figure 7 now. Figures and captions are revised. However, we could not include statistical findings in this figure, because the statistics were only conducted on the treatment groups at downstream sites pre- and post-harvest, not individual sites.**
- 463: I am not sure if you can make the argument that light limitation has been eliminated in these streams, particularly in any of the streams besides the variable buffer, as these were the only streams that showed a real increase in the amount of light
  ***Response:* We recognize that light did not increase at all harvested sites. The light limitation statement refers to our expectations from the literature, not these specific streams.**
- 467: I think this sentence is a bit misleading because you had riparian buffers in all treatments, it is just that some were more variable and some more uniform
  ***Response:* We revised this sentence to explicitly note that all clearcut watershed had increases in DIN.**
- 461-471: I think that the first paragraph of the discussion could be a little bit stronger by increasing the organization and making it more succinct
  ***Response:* We revised this paragraph to make it stronger and more succinct.**
- 473: I know that a main point that is being made in this paper is the challenge of distinguishing statistical significance in landscape scale experiments, but this feels like it could be a concluding section
  ***Response:* We debated where best these paragraphs fit into the Discussion – thank you for your input.**
- 484: I think this paragraph should come before the first paragraph in this section, as this one starts broader and the last one is specific to your study
  ***Response:* Good suggestion, we switched the order of the paragraphs**
- 487: I think you could include more references here to support this statement
  ***Response:* We added more references.**
- 488: This sentence is not very clear, because I am not sure where the similarities end and the differences begin
  ***Response:* We revised this sentence to better distinguish what are similarities and differences.**
- 493: I think that this brings up some good points about the difficulties of field sampling, but I think in the context of this paper it strays a little bit far from the focus on algae
  ***Response:* We revised and with our focus being more than algae, we suggest that discussion of broader issues are relevant for this manuscript.**
- 500: I brought this up earlier, but I think that it is better to limit your discussion to forest harvest, rather than bringing in disturbance more generally
  ***Response:* Thank you - we revised this.**

- 503: You again bring up the multiple trophic levels aspect, but you don't really talk about what trophic levels you mean
  Response: **We removed the phrase multiple trophic levels. With numbered citations, it is not easy for readers and reviewers to see what studies are being referenced. The prior citations pointed to Trask macroinvertebrate and fish research findings.**
- 505: You might want to state why this is the case about nutrients
  Response: **We added citations and some text to clarify. However, the multiple reason for seasonality of nutrient dynamics are beyond the scope of this paper.**
- 511: It feels broken up to talk about being surprised by the finding of the lack of effect of riparian buffers here, and then moving paragraphs to continue talking about them. Also, it would be helpful for you to compare these values to the literature of studies that did not have any riparian buffer to support your claims.
  Response: **We revised, and now the discussion of riparian buffer influences on nutrients is in one paragraph.**
- 523: I think that these first two sentences get a little off topic, when it should focus more directly on downstream effects on DIN. In fact, I think you could shorten this paragraph substantially and add it onto the last paragraph.
  Response: **We moved this section up to the previous paragraph as suggested and revised and shorted the discussion**.
- 531: I like that you address a lack of effect on the other water chemistry, but I also think this paragraph could be written more concisely
  Response: **This paragraph was revised to be more concise as suggested.**
- 549: I am concerned that you say that increased nutrients have long been recognized to stimulate autotrophic responses in aquatic ecosystems, but only cite one reference. I think this would be a stronger statement if you included more references and focused only on headwater streams.
  Response: **There are many references for this and we included several more.**
- 557: So how can you explain the lack of response of chlorophyll in PH2 and PH3 if they have the required ratios of N:P?
  Response: **That is exactly one of our questions. We think that the lack of green algae in this system is part of why we did not see responses of chlorophyll.**
- 561: Trask River Watershed?
  Response: **Yes, thank you, we clarified this.**
- 565: The sentence starting here is a bit long and run-on-y. Also, I think that this argument would be stronger if you could provide evidence of to what temperature the stream would need to warm in order for the stream to shift from cold-water diatoms to filamentous algae
  Response: **Thank you, the sentence has been shortened and temperature more clearly addressed. Much remains unpublished about thermal thresholds for diatom taxa. However, the shift we observed in temperatures was apparently not enough to result in changes to the dominant diatom taxa.**

- 572-574: I think you should talk more about the specific species you found and how they may or may not have responded to nutrients
  Response: **We now include additional text and citations about the diatoms observed in these streams. An abundant diatom in our streams is considered to 'successfully compete at low resource (light and nutrients) and high physical stress levels' Biggs et al. 1998.**
- 581: Based on my comments above, I think this paragraph is more distracting than helpful to your paper.
  Response: **As suggested, we removed this paragraph**.
- 597: I am glad you did talk about grazers, but I am concerned that the role of grazers has been dismissed to easily, and more literature should be brought into this part of the discussion.
  Response: **We now include more literature and explicitly note that grazing snails were totally absent from these watersheds and that crayfish were rare. In these forested ecosystems without filamentous algae, the crayfish tend to be detritivores**.
- 621: Evidenced?
  Response: **Opps, thank you.**
- <u>Discussion generally</u>:
  - I think that there is not enough attention given to the role of light and grazers. There was an increase in nutrients, but light really only increased substantially in one site.
    Response: **In our revisions, we worked to emphasize the changes in light as a function of annual closure measurements. Tables 2 and 3 show that canopy closure was significantly different pre- to post- for the Clearcut variable group and significantly different pre to post at all three watersheds that comprise that treatment group. Grazers are discussed, and we note that they were not a major component of the aquatic food webs in these streams.**
  - I also think more attention could be played to the role of sediments in these streams.
    Response: **We included analyses of sediment because changes in sediment could impact algal standing stocks and chlorophyll *a*. Because instream sediment did not change after harvest, we noted that it did not likely have a role in suppressing epilithon responses.**
  - There is also no explanation for why chlorophyll a seems to mostly decline in the reference streams while remaining the same in the treatment streams. Another concern that I have is that even though they noted increases in nutrients and light, I wonder if the magnitude of these changes was enough to change the algae.
    Response: **These are good questions. Other studies have found algal responses with much less change in light and in nutrients. As to post-harvest declines in reference sites, we now include our speculations that chlorophyll a concentrations decreased in reference as well as treatment sites because the post-harvest period was slightly warmer, dryer with somewhat lower streamflows than during pre-harvest period.**
  - I also think that there does not seem to be an attempt to look at any of these factors as a function of time, which would be expected to change as new vegetation grows in and could account for variability.

Response: **We addressed this comment above and noted that highest nitrate concentrations did lag the harvest. However, four years post-harvest is not sufficient for time series or much revegetation regrowth and we did not conduct analyses of it.**

- Finally, I think one of the most interesting things in this paper was how variable the chlorophyll a was in these streams under pre-harvest conditions was highly variable – in fact much more so than in many of the post-streams.
  Response: **Yes, it is interesting how different these streams were in the pre-harvest period, even though they had many similar conditions, including harvest history, light, vegetation cover and proximity.**

While revising your submission, please upload your figure files to the Preflight Analysis and Conversion Engine (PACE) digital diagnostic tool, https://pacev2.apexcovantage.com/. PACE helps ensure that figures meet PLOS requirements. To use PACE, you must first register as a user. Registration is free. Then, login and navigate to the UPLOAD tab, where you will find detailed instructions on how to use the tool. If you encounter any issues or have any questions when using PACE, please email PLOS at figures@plos.org. Please note that Supporting Information files do not need this step.

Data Review URL

Click here to download Data Review URL http://doi.org/10.2737/RDS-2022-0001

Click here to download Data Review URL http://doi.org/10.2737/RDS-2022-0002

Data Review URL

Click here to download Data Review URL http://doi.org/10.2737/RDS-2022-0003
