# Supplementary material for: Stream nitrate enrichment and increased light yet no algal response following forest harvest and experimental manipulation of headwater riparian zones
Source: PLoS One. 2023 Apr 20;18(4):e0284590. doi: 10.1371/journal.pone.0284590 (PMC10118188; doi:10.1371/journal.pone.0284590)
Supplement: Attachment — Submitted filename: PONE-D-22-25727 Comments.docx [file pone.0284590.s007.docx]

**Summary of the Paper and Overall Comments**

Thank you for giving me the opportunity to review this paper submitted to PLOS One. I think that the dataset analyzed in this paper has many strengths, including the longevity of the research with multiple years of pre-harvest controls, numerous watershed pairings, consideration of spatial complexities when studying stream ecosystems, extensive complementary datasets from a multi-disciplinary team, consistent annual sampling design, and algal composition data reviewed by a co-author who leads the field in diatom identification. While I could not speak specifically to the nuances of the partial least squares regression method due to my lack of familiarity with this type of analysis, I recognize the strength of using multiple non-parametric statistical methods to analyze field data characterized by a high degree of natural variability. Consequently, these data offer an important contribution to our understanding of how forest management affects in-stream primary producers and I sincerely hope that these data are published. However, I did feel that there were numerous and substantial issues with the paper as written that lead me to recommend that this paper be categorized as needing “major revisions.” My concerns with the paper tend to fall into three categories. First, while there were many strengths in the methodologies used in this study, I do not feel that the authors provided enough detail on the specifics of how algae were collected from the stream in the methods and did not do enough to recognize the limitations of some of the methods that they used in the discussion. Second, while many of the figures were relatively visually clear, I found some inconsistencies with labeling to make it difficult to compare among figures. There was also a substantial amount of information that I felt was missing from the figure legends pertaining to the statistical analyses. I also disagreed with the use of standard deviations for error bars when the authors calculated 95% confidence intervals as part of their statistical analyses (and are more easily interpreted for statistical relevance in bar graphs). Finally, I felt that there were some substantial issues with the writing, particularly related to the framing of the data analysis and the literature used to support their findings. In particular, there were many areas of the writing that were too broad and drew from a broader literature source; focusing more specifically on riparian studies in headwater streams would allow the authors to make more direct comparisons and strengthen their claims.

**Specific Reviewer Comments**

- 34: This description does not seem to account for the “thinned” treatment – is there a better way to write a succinct description of the treatments to make it clearer?
- 39: I am not sure you discuss the importance of the algal community enough to include it as a major point in your abstract as written. I would like to see you spend more time talking about the algal species composition in the paper to justify it here.
- 49: This may be more of a stylistic comment, but I am not sure I like the use of sub-titles within the introduction in a non-review style paper
- 53: I think your argument about the importance of studying harvest would be strengthened if you could provide some kind of estimate of the percent of forests harvested in your study region, the US or globally
- 54: This statement would also be strengthen with some citations about both the diversity of aquatic organisms and the use of headwater streams as upstream sources of water for downstream communities
- 59: I think it would be helpful to have a more direct tie from the effects of harvest to the use of riparian buffers as a management strategy
- 62: Rather than saying “minimize algal blooms,” it seems this sentence should focus on the effect on light directly
- 64: This part of the sentence is a little unclear: “Changes to any or all of these functions and ecosystem services” – Changes due to what? Functions and ecosystem services provided by forested riparian areas?
- 70: I found this sentence to be very unclear – I was not sure what exactly you meant by “lower trophic levels of food webs” – Is this stream food webs or forest food webs? Is it primary producers or are herbivores also included? Is the focus here on removal of riparian vegetation or forest management at large? Because this is the first sentence in the paragraph, I am having a hard time figuring out the exact direction that this paragraph is going.
- 73: You talk about the functions of the riparian buffer, but think it would be helpful in the paragraph above to talk about how the function of the riparian buffer vary with width – then this sentence will make more sense
- 75: when you talk about stream parameter or process is of most interest, are you referring to protection of these processes as part of the establishment of riparian buffers?
- 75: The focus on nitrogen in this paragraph seems to have not been a logically led in by the initial sentences in this paragraph. It might be helpful to start with this sentence, and then take the information from the first two sentences to make them more specific to nitrogen, since this seems to be the focus
- 77: I would remove the information about fire-related disturbances, since you have narrowed to forest harvest in the first part of your intro
- 79: Again, I think focusing just on the effect of harvesting on nitrogen, rather than on “forest disturbance” would be more focused here
- 80: Will the general readership of PLOS one know the term “epilithon” or should it be defined?
- 80: Here is another place that I feel the writing is disorganized – the preceding sentences were about nitrogen, but then we jump to epilithon. It seems that this whole paragraph could be more focused by having a general subject of thinking about the effect of riparian buffers on abiotic conditions in streams, and then having a separate paragraph that talks bout the connection to the biotic
- 82: It seems like you should not include natural gaps in the canopy cover – it takes away the focus on riparian buffers to protect from harvest
- 83: It might make sense to first start by talking about the expected effects of nitrogen and light on biomass of epilithon, then get into the taxonomic effects
- 85: I have a problem with starting the sentence by saying that light changes composition of algae in the stream, but then supporting that with clearcutting (because there are many other abiotic factors that are affected by clearcutting)
- 87: Can you be more specific on the ways that diatoms and filamentous algae affect consumers? Talking about which group is more palatable and/or which consumers benefit or don’t benefit?
- 89: I think that the last sentence about not supporting the common paradigm is a bit bold, and think maybe there should be more support provided to support this
- 90: What do you mean by “this complexity of responses”?
- 91: I wonder if the issue is not so much the focus of resource managers and research on statistically significant findings, but rather the inflexibility of commonly used statistical tests to capture biological variability and significance in dynamic systems like streams
- 95: I think that you need to be a little clearer than stating “may not be supported” – Do you mean that the assumptions might not be met? That the methods might not be appropriate?
- 96: Can you be more specific in what you mean by “dueling statistics” Do you mean using multiple statistical approaches?
- 96: I think you lost me on this final sentence – it seems to relate more to how managers manage riparian vegetation, rather than its relationship to the discussion about statistics
- 101: I am not sure what you mean by “extent” of riparian buffers – do you mean width? Length? Quality?
- 101-104: This sentence is a little long, and might benefit from being shortened or turned into two sentences. Also, are you just measuring standing stock of algae or composition as well? If not measuring composition, I think that information can be removed from above.
- 104: Again, because you don’t specify what you mean by “extent,” in the first sentence, it is hard to know what you mean by “more removal” of riparian vegetation
- 105: I think it would be better to separate the effect on light and the effect on algal biomass
- 106: I would rephrase this part of the hypothesis to start with the effect you expect on nitrogen and then say why
- 108: I am a little confused here because you talk about nutrients and dissolved organic carbon, but the intro really just focuses on nitrogen. Which do you measure?
- 108: You don’t really discuss sediment at all in the intro, which I think is problematic to bring it in here in the hypothesis
- 111: Is this dissolved carbon or are you measuring other forms of carbon? I also think you could change this to be one sentence by saying “Do forested riparian buffers reduce increases in in-stream concentrations of dissolved organic carbon and nutrients in harvested forest watersheds?”
- 114: This question also does not line up with anything in the introduction or in the hypotheses, because you don’t mention temporal or spatial extent anywhere else.
- 116: Should this say “chlorophyll a”? I also don’t think you can bring in light here if you don’t mention light as something that you are directly measuring as a question.
- 120: Should there be a reference to a figure at the end of this sentence?
- 128: Rather than saying “prior disturbances, including…” I think you should just say “disturbance due to wildfire” because you don’t give examples of any other types of disturbances
- 129: I think it would be helpful to know the extent of this burning and logging within the watershed – ideally a map, but even just report of percentages would be helpful.
- 136: did you evaluate downstream effects in this study, or was this just an objective of the overall study?
- 138: Were there 12 treatment and 12 reference or 6 treatment and 6 reference streams?
- 140: Were there riparian buffers left in these? Since this is a key point in the study, it seems like it would be good to mention it here
- 146: I found it really difficult to read the legend on your figure. I am also not if you need both the satellite image and the map here. I also think it is problematic to introduce the reader to your treatment types for the first time in the Figure legend. I don’t know what you mean by “variable buffers’ or “uniform buffers” – a more specific description of the buffers that were left and the harvesting strategy would be helpful. Also in your figure legend, it would be helpful to give context for the 2013 year (first year post-harvest could be added).
- Table 1: I think it would look better in the harvest treatment to remove the underscore from the description. Do you think it is necessary to state the ownership of the watershed? You might instead just want to indicate if it was private or public. Also, is it possible to rename your watersheds so that all of the references are 1 and the clearcuts are the remaining numbers? I know this might not be possible if you are working in a large group. Did you run any statistics to compare the differences among your reference and treatment watersheds to make sure they were similar in some of the physical characteristics? Also, what is a “15m no riparian buffer?”
- 151: I am a little unclear as to whether these guidelines apply to the private or public lands or both
- 153: Can you provide a definition of “leave trees” in the prior sentence by using this term so that it is more familiar when you state what happened in the UM2 and UM3 watersheds
- 156-158: A few points of clarification about this treatment are needed – the first is how “perennial stream” is being defined by the logging company and if that is consistent with aquatic ecologist definition of the streams in this area. It is also unclear how even the 12m of buffer were in this treatment – were they really even enough to be considered “uniform buffers”?
- 161: It would be helpful to start this sentence by stating if this watershed is a treatment watershed.
- 167: I feel like this sentence is written as if you have not just described the watersheds, rather than stating that these were within the watersheds you just described.
- 167: I do feel I need to question whether 30m is a long enough reach to make these measurements
- 189: Do these streams have any crayfish that could influence algae?
- 191: I don’t think you need to list the physical characteristics that you measure, because you then go on to describe each of them.
- 193: Is this an n=4 measurements in a 30 m reach?
- 200: I am assuming based on the sampling time selected in June that there was complete canopy closure at this time for deciduous trees, but I think it would be helpful to clarify this, particularly for the higher elevation sites
- 206: Did the downstream measurements go upstream of the site or downstream of the site?
- 206: Do you ever address the hemispherical photo data later in the results or compare it with the densiometer data? I don’t remember seeing that information.
- 211: I am not sure what you mean by “percent total site factor”
- 212: Did you take all images on a cloudless day to enable the digital image analyzer, or does this analysis program allow you to account for things like cloud cover?
- 213: This last sentence feels very out of place after a whole paragraph about canopy cover
- 222-226: I think it is worth mentioning here again that if you are focusing on nitrogen, phosphorus, and dissolved organic carbon as drivers, these all should be mentioned somewhere in your introduction
- 229: I would like to see a little more description made about how the algae was collected How were the site for algae collection selected within the stream reach? Why did the number of rocks vary per site? Were they in pools, riffles? Were they collected at the same location as the sediments? Did you calculate the area of the rocks that you used? What method did you use to calculate rock area?
- 242: I think it would be helpful if you clarified where these algal structure data were collected relative to the other samples for biomass
- 245: I am wondering if it is problematic that your only taxonomic analysis occurred in the first year post-harvest and not in subsequent years as well give how much disturbance there likely was in the first year.
- 254: I think that you should cite all of the resources used for the diatom analyses, rather than as an “e.g.”
- 257: I think that it would be better to put this additional sampling up by the section where you talk about biomass since it doesn’t sound like you measured algal composition on these additional samples
- 277: I think that you should not use KS2, but either write out the full name of the test or summarize what you are doing (or take out subtitle)
- 279: I think you need a comma after “thinned” to distinguish it is a separate group from reference
- 298: I don’t think that treatment group, harvest period, etc should be capitalized here
- 330: One question that I have is how you treated replicates within each of these statistical analyses – did you treat multiple samples from a stream as pseudoreplicates?
- 337: A question I now have looking at your discharge measurements – did you have any requirement for recent rain event when you did your sampling, or did you just sample regardless of flow conditions? This might be helpful to clarify in the methods
- 343: I think you should report the magnitude of the temperature increases in the text of the results section. Also, were these part of your statistical analyses?
- 345: If you are going to talk about temperature first, then I think this should be the first sub-panel in Figure 2, rather than the second (since you haven’t yet referenced 2A).
- Figure 2: I think this figure would be improved with better labeling. For example, for the treatments, it would be helpful if you had the width of the riparian buffer in parentheses next to the treatment name. Also, are the downstream sites here? If not, you might want to include them and/or specify this is only the headwater stream sites. I am also unclear as to why the light measurements do not have error bars. Didn’t you take multiple measurements? Also, where is there a graph or table showing the difference in sediment among the sites? I think this would be very helpful to have graphically.
- Figure 2 caption: I think there are a number of issues with this caption. First, there is no indication of the sample size or statistics for each measure. Second, I personally prefer the use of standard error or confidence interval rather than standard deviation for comparing means. Didn’t you calculate the 95% confidence intervals for each of these groups using the bootstrapping method? Couldn’t you plot those here? Also, you say in the description for A that you calculated incoming radiation, but I don’t think that is quite correct – usually radiation is measured using a light meter, but you just measured canopy coverage vs. no coverage.
- Table 2: I think the bold text is a little hard to read – could you use an asterisk instead? I also found the symbols for the PLS model to be a little bit confusing.
- Figure 3: I think it is important to make your labels consistent between Figure 2 and Figure 3.
- 365-374: I think that logically it makes more sense to talk about DOC, SRP, and DON in the same order in the text as they appear in the figure. I also think it is important to include your n somewhere in the text for reference.
- Figure 3 caption: I think it is also important for you to include more of your statistics in the figure caption as well, particularly the n, the p-value you used from one of your tests. Same comment about the use of 1 standard deviation here compared with the value of 95% CI or standard error.
- 377-382: One question that I have is how these are varying from one year to the next post-harvest – is that what can explain the large variability in the error bars? I would predict that the DIN would initially be high, but then decrease as more vegetation grows in.
- Table 3: I think that you need to have a better description of epilithon here, as it does not indicate that what you are measuring is AFDM. I also think that generally, the information presented from the PLS would be better as a graph, rather than as a table, because you could visualize how different groups move from one to another. You couldn’t do this for all variables, but maybe you put this table in your supplementary materials and make a graph with only the most interesting of the points you want to illustrate.
- 394: You bring in the molar ratios of DIN: SRP here, but I think this is the first time that you talk about it – why might this be important to consider? If you think it is important, maybe highlight this point in your introduction.
- Table 4: I am wondering if table 4 should be a fifth panel in the other panel about water quality – this is very hard to visualize looking at it in a table, and it seems that tables should be reserved for providing the output of statistical tests, rather than means and SD.
- Figure 4 – I think it would be helpful to indicate which downstream site is the reference and separate it out from the other three sites visually. Also, were each of these sites mixed in terms of the upstream harvesting, or could they also be grouped by the harvesting method?
- Figure 4 caption: This caption has the same issues as the other ones – there are no statistics provided or indication of n.
- Figure 5: Same issue with consistent labeling with previous figures.
- Figure 5 caption: Same issues as prior captions
- 437: I think it would be helpful if you moved this section with chlorophyll a correlations to a new paragraph, since it is referring to different analyses
- 438: One thing that I think is a bit misleading here is that you correlate “light reaching the stream” with algal concentration – but the problem is that your canopy cover measurements were not taken at the same locations as your algae measurements. I think you need to clarify this point by saying “mean reach-scale canopy cover” to indicate that it is representative of the whole reach.
- Figure 7: I am not sure that I find the data presented here to be very convincing or helpful, and I think the tile study should be eliminated (or just mentioned briefly without a figure). It appears to me that the tiles were substantially lower, which suggests to me that either the incubation time was too low, or that there was an issue with the estimate of rock area potentially. It also is not as helpful as it could be because these measurements were only made once post-cutting, instead of clear cutting.
- 445-449: It was a little unclear to me if the diatoms you discuss here were found pre-harvest, post-harvest, or both – I am particularly confused by the line starting on 448. It seems to me that this part of the results deserves much more attention and description. Also, rather than just stating the most common taxa, it seems like you should do some kind of indicator species analysis to have a better way of making a cutoff of important taxa. Also, could you give some indication of the type of diatoms these are in terms of their growth form in the table, and then use this as a basis of discussion in the discussion?
- 453: I am a little confused by the line that starts here because you say “they” and I am not sure to what group this refers.
- Figure 8: there is the same issue with labeling and captioning here.
- 463: I am not sure if you can make the argument that light limitation has been eliminated in these streams, particularly in any of the streams besides the variable buffer, as these were the only streams that showed a real increase in the amount of light
- 467: I think this sentence is a bit misleading because you had riparian buffers in all treatments, it is just that some were more variable and some more uniform
- 461-471: I think that the first paragraph of the discussion could be a little bit stronger by increasing the organization and making it more succinct
- 473: I know that a main point that is being made in this paper is the challenge of distinguishing statistical significance in landscape scale experiments, but this feels like it could be a concluding section
- 484: I think this paragraph should come before the first paragraph in this section, as this one starts broader and the last one is specific to your study
- 487: I think you could include more references here to support this statement
- 488: This sentence is not very clear, because I am not sure where the similarities end and the differences begin
- 493: I think that this brings up some good points about the difficulties of field sampling, but I think in the context of this paper it strays a little bit far from the focus on algae
- 500: I brought this up earlier, but I think that it is better to limit your discussion to forest harvest, rather than bringing in disturbance more generally
- 503: You again bring up the multiple trophic levels aspect, but you don’t really talk about what trophic levels you mean
- 505: You might want to state why this is the case about nutrients
- 511: It feels broken up to talk about being surprised by the finding of the lack of effect of riparian buffers here, and then moving paragraphs to continue talking about them. Also, it would be helpful for you to compare these values to the literature of studies that did not have any riparian buffer to support your claims.
- 523: I think that these first two sentences get a little off topic, when it should focus more directly on downstream effects on DIN. In fact, I think you could shorten this paragraph substantially and add it onto the last paragraph.
- 531: I like that you address a lack of effect on the other water chemistry, but I also think this paragraph could be written more concisely
- 549: I am concerned that you say that increased nutrients have long been recognized to stimulate autotrophic responses in aquatic ecosystems, but only cite one reference. I think this would be a stronger statement if you included more references and focused only on headwater streams.
- 557: So how can you explain the lack of response of chlorophyll in PH2 and PH3 if they have the required ratios of N:P?
- 561: Trask River Watershed?
- 565: The sentence starting here is a bit long and run-on-y. Also, I think that this argument would be stronger if you could provide evidence of to what temperature the stream would need to warm in order for the stream to shift from cold-water diatoms to filamentous algae
- 572-574: I think you should talk more about the specific species you found and how they may or may not have responded to nutrients
- 581: Based on my comments above, I think this paragraph is more distracting than helpful to your paper.
- 597: I am glad you did talk about grazers, but I am concerned that the role of grazers has been dismissed to easily, and more literature should be brought into this part of the discussion.
- 621: Evidenced?
- Discussion generally: I think that there is not enough attention given to the role of light and grazers. There was an increase in nutrients, but light really only increased substantially in one site. I also think more attention could be played to the role of sediments in these streams. There is also no explanation for why chlorophyll a seems to mostly decline in the reference streams while remaining the same in the treatment streams. Another concern that I have is that even though they noted increases in nutrients and light, I wonder if the magnitude of these changes was enough to change the algae. I also think that there does not seem to be an attempt to look at any of these factors as a function of time, which would be expected to change as new vegetation grows in and could account for variability. Finally, I think one of the most interesting things in this paper was how variable the chlorophyll a was in these streams under pre-harvest conditions was highly variable – in fact much more so than in many of the post-streams.
