# Supplementary material for: Stream nitrate enrichment and increased light yet no algal response following forest harvest and experimental manipulation of headwater riparian zones
Source: PLoS One. 2023 Apr 20;18(4):e0284590. doi: 10.1371/journal.pone.0284590 (PMC10118188; doi:10.1371/journal.pone.0284590)
Supplement: Attachment — Submitted filename: PONE_response to reviewsDec22.docx [file pone.0284590.s008.docx]

PONE-D-22-25727

The paradox of stream nitrate enrichment and increased light yet no algal response following experimental manipulation of riparian zones and forested watersheds

PLOS ONE

Dear Dr. Johnson,

Thank you for submitting your manuscript to PLOS ONE. After careful consideration, we feel that it has merit but does not fully meet PLOS ONE’s publication criteria as it currently stands. Therefore, we invite you to submit a revised version of the manuscript that addresses the points raised during the review process.

I encourage to carefully consider all the comments of the reviewers, especially those from reviewers 2 and 3.

Please submit your revised manuscript by Dec 12 2022 11:59PM. If you will need more time than this to complete your revisions, please reply to this message or contact the journal office at [plosone@plos.org](mailto:plosone@plos.org). When you're ready to submit your revision, log on to <https://www.editorialmanager.com/pone/> and select the 'Submissions Needing Revision' folder to locate your manuscript file.

Please include the following items when submitting your revised manuscript:

- A rebuttal letter that responds to each point raised by the academic editor and reviewer(s). You should upload this letter as a separate file labeled 'Response to Reviewers'.
- A marked-up copy of your manuscript that highlights changes made to the original version. You should upload this as a separate file labeled 'Revised Manuscript with Track Changes'.
- An unmarked version of your revised paper without tracked changes. You should upload this as a separate file labeled 'Manuscript'.

If you would like to make changes to your financial disclosure, please include your updated statement in your cover letter. Guidelines for resubmitting your figure files are available below the reviewer comments at the end of this letter.

If applicable, we recommend that you deposit your laboratory protocols in protocols.io to enhance the reproducibility of your results. Protocols.io assigns your protocol its own identifier (DOI) so that it can be cited independently in the future. For instructions see: [https://journals.plos.org/plosone/s/submission-guidelines#loc-laboratory-protocols](https://nam04.safelinks.protection.outlook.com/?url=https%3A%2F%2Fjournals.plos.org%2Fplosone%2Fs%2Fsubmission-guidelines%23loc-laboratory-protocols&data=05%7C01%7Csherri.johnson%40oregonstate.edu%7C840e28fe26ef484d8c8708dab90105da%7Cce6d05e13c5e4d6287a84c4a2713c113%7C0%7C0%7C638025711598882592%7CUnknown%7CTWFpbGZsb3d8eyJWIjoiMC4wLjAwMDAiLCJQIjoiV2luMzIiLCJBTiI6Ik1haWwiLCJXVCI6Mn0%3D%7C3000%7C%7C%7C&sdata=V4vINfVXnk%2B80RJsV4csen18UUS8%2B%2BZssTJ0SJpmPuE%3D&reserved=0). Additionally, PLOS ONE offers an option for publishing peer-reviewed Lab Protocol articles, which describe protocols hosted on protocols.io. Read more information on sharing protocols at [https://plos.org/protocols?utm_medium=editorial-email&utm_source=authorletters&utm_campaign=protocols](https://nam04.safelinks.protection.outlook.com/?url=https%3A%2F%2Fplos.org%2Fprotocols%3Futm_medium%3Deditorial-email%26utm_source%3Dauthorletters%26utm_campaign%3Dprotocols&data=05%7C01%7Csherri.johnson%40oregonstate.edu%7C840e28fe26ef484d8c8708dab90105da%7Cce6d05e13c5e4d6287a84c4a2713c113%7C0%7C0%7C638025711599038822%7CUnknown%7CTWFpbGZsb3d8eyJWIjoiMC4wLjAwMDAiLCJQIjoiV2luMzIiLCJBTiI6Ik1haWwiLCJXVCI6Mn0%3D%7C3000%7C%7C%7C&sdata=0B25SvDzERGQmOQ3y77zA1bsgJmYaY8hOxY%2FNME%2B1dc%3D&reserved=0).

We look forward to receiving your revised manuscript.

Kind regards,

Clara Mendoza-Lera
Academic Editor
PLOS ONE

Journal Requirements:

When submitting your revision, we need you to address these additional requirements.

1. Please ensure that your manuscript meets PLOS ONE's style requirements, including those for file naming. The PLOS ONE style templates can be found at 
<https://journals.plos.org/plosone/s/file?id=wjVg/PLOSOne_formatting_sample_main_body.pdf> and 
<https://journals.plos.org/plosone/s/file?id=ba62/PLOSOne_formatting_sample_title_authors_affiliations.pdf>

***Response*: Corrected**

2. We note that the grant information you provided in the ‘Funding Information’ and ‘Financial Disclosure’ sections do not match. When you resubmit, please ensure that you provide the correct grant numbers for the awards you received for your study in the ‘Funding Information’ section.

***Response*: We revised Funding Information and Financial Disclosure. There were no grant numbers for the funding provided for this study**

- 3. We note that Figure 1 in your submission contain [map/satellite] images which may be copyrighted. All PLOS content is published under the Creative Commons Attribution License (CC BY 4.0), which means that the manuscript, images, and Supporting Information files will be freely available online, and any third party is permitted to access, download, copy, distribute, and use these materials in any way, even commercially, with proper attribution. For these reasons, we cannot publish previously copyrighted maps or satellite images created using proprietary data, such as Google software (Google Maps, Street View, and Earth). For more information, see our copyright guidelines: <http://journals.plos.org/plosone/s/licenses-and-copyright>.
   We require you to either (1) present written permission from the copyright holder to publish these figures specifically under the CC BY 4.0 license, or (2) remove the figures from your submission:
   a. You may seek permission from the original copyright holder of Figure 1 [#] to publish the content specifically under the CC BY 4.0 license.  We recommend that you contact the original copyright holder with the Content Permission Form (<http://journals.plos.org/plosone/s/file?id=7c09/content-permission-form.pdf>) and the following text:
  “I request permission for the open-access journal PLOS ONE to publish XXX under the Creative Commons Attribution License (CCAL) CC BY 4.0 (<http://creativecommons.org/licenses/by/4.0/>). Please be aware that this license allows unrestricted use and distribution, even commercially, by third parties. Please reply and provide explicit written permission to publish XXX under a CC BY license and complete the attached form.” Please upload the completed Content Permission Form or other proof of granted permissions as an "Other" file with your submission. 
  In the figure caption of the copyrighted figure, please include the following text: “Reprinted from [ref] under a CC BY license, with permission from [name of publisher], original copyright [original copyright year].”
   ***Response*: We removed any potential copyright question with our updated Fig 1. Our map was made by us using ArcGIS Pro with publicly available data layers of LiDAR terrain (https://www.oregongeology.org/lidar/) and with the hydrography layer derived from the LiDAR.** **The aerial image that was part B was removed. We had permission from Elsevier to cite this image which was previously published in our manuscript in Forest Ecology and Management, but Elsevier would only provide their signed copyright permission and was not able to sign your copyright permission form.**

**.**

**5. Review Comments to the Author**

**Reviewer #1:** This paper tests the response of algal biomass to different forest harvest, and riparian buffer practices. This study was conducted over ten-years and twelve streams, which is quite impressive and unusual. This work is interesting and high quality. While the work underling this manuscript is of good quality I have some questions about the statistics used to reach the conclusions of this work. Additionally, I think this manuscript could be more useful to other scientists and managers if it was shortened and simplified significantly. Please see my detailed comments below.

***Major issues:***
• Lines 257-263 and subsequent sections of results and discussion: This section describes the methods used to measure extra samples in July of 2016 and compare the measurements of algae on rocks to algae on tiles. Because the goals of this are not mentioned in the introduction (e.g., you do not have hypotheses stated in the introduction) this feels out of place here – particularly the comparison of between rocks and tiles. From the discussion section I understand that part of the motivation for this was to understand whether you were sampling too early in the season to see an effect of the treatments – but I don’t understand how the tiles fit into the goals of the present study. Additionally, the results from this don’t seem to really shed light on the findings of the main part of the study. I suggest you move all of this material to an appendix, and briefly reference the findings in the discussion.

***Response:* We removed the data from the sampling of tiles and our comparison of biomass on rocks in July is now in the Supplemental material. We briefly reference it in the discussion. We had included the tile comparison in year 4 post-harvest because we were surprised by not seeing any response in years 1-3. Tiles were added because many of the studies in the literature used tiles rather than sampling natural substrates and we had questions if our sampling of natural substrates might be influencing the lack of responses of algal biomass and chlorophyll *a* that we were observing. We added the July sampling to examine possible later summer responses, because the literature contains multiple examples where algal responses were studied only later in the summer.**

• Statistical methods: This paper uses multiple statistical methods to test the same questions, about how the forest harvest/ buffer treatments effects in-stream variables. The inclusion of multiple statistical methods add considerable length to the manuscript, and complexity to an experimental design that is quite simple. This manuscript would be much clearer if there was a single method used – and less statistical tests generally.

***Response:* We think the three methods are a strength of the paper, especially, where consensus among the methods occurs. We acknowledge that their importance was not clear enough in the original manuscript, so we have modified the discussion and the introduction to clarify the purpose of doing multiple statistical tests. The three analyses were applied to the question of pre-post differences with the belief that consensus among two or more of the methods gives a stronger result than any one method by itself. The use of multiple methods is further warranted given the data challenges: spatial and temporal dependency, small sample sizes and the possibility of not finding an acceptable PLS model given the more stringent quality criterion using the R^2^CV. The Kolmogorov-Smirnov 2 sample test (KS2) compares distributions of each pre-post pair. The BCa bootstrap calculates the 95% confidence intervals for the means, which can be compared for each pre-post pair. The PLS models are the only cases where all the data for a specific response are compared, and because of our stringent model acceptance criterion, not all PLS models were valid. We feel these three techniques provide value and are standard applications available in many statistical packages. PLS with Box-Cox transformations and wild bootstrap does add some complexity but allowed us to get better models and to deal with lack of independence in the data.**

• Line 307: Your hypotheses are all related to the effect of forest treatment across the watersheds which are essentially replicates. I don’t understand the need to look at changes in each watershed individually as you describe here.

***Response:*** **Although we started with a study design where watersheds within a treatment were planned to be replicates, the study sites within a treatment group showed variability in site conditions and responses and we were interested in exploring that variability. In addition, ‘real world’ implementation of treatments was not uniform, especially where no buffers were required (variable buffer sites). This led us to include examination of responses within each watershed. The evaluation of treatment categories is important for evaluation of forest plans and forest practices act, while the evaluation of individual watersheds addressed the variability observed in the forests and streams.**

• Line 437-439: Describes correlations between chlorophyll-a and light, I don’t see where these regressions are described in the methods.

***Response:* Thank you for pointing this out. We now include details about the correlations and statistics for them.**
• Figures: The goal of this manuscript is to investigate the instream responses to forest and riparian zone management. In the context of this, each stream is essentially a replicate – and I think it would make more sense to present them this way in the figures. Many of the figures show multiple bars for each of the streams. Presenting bar graphs grouped by treatments would fit better with your experimental design and would give the readers much less extraneous information. The figures in the present version could be moved to an appendix if you would like to keep that information available to a reader.

***Response:* The bars in the figures are consistently displayed and grouped by treatment so that readers can best see the variability of responses and which sites might be contributing to the treatment group response, rather than only presenting treatment averages. To help readers see the treatment groups, we used a specific fill pattern consistently for each treatment group. As you note below, watersheds are not true replicates, so presenting details by individual watershed can help readers understand what was** **influencing the observed responses.**
• Line 312-325. You describe the use of Wu’s jackknife to account for non-independence of measurements. I am more familiar with non-independence being addressed with random effects in mixed-effects models (e.g., as described in the books by Zuur 2009, or Wu 2009), than through bootstrapping. Reading Wu 1986 they seems to specifically caution against using their method in situations with non-independence. From Wu; “Our analysis of the bootstrap …. encounters similar difficulties in handling complex survey samples, where neither the independence nor the exchangeability assumption is satisfied (Rao and Wu (1985a)).” From my own exploration of your data it does appear that there is non-independence within dates and sites, with roughly half of the variation in the chlorophyll-a data explained by random effects for date and site. Please clarify if, and how the bootstrapping addresses these forms of non-independence in your data.

***Response:* Wu (1986) proposed a weighted bootstrap which did very well in the tests given the Wu’s Table 1, especially, compared to the other (unweighted) bootstrap. However, Liu (1988) improved on Wu’s weighted bootstrap with what became known as the wild bootstrap. Other researchers made further improvements and/or proved additional results, especially for non-independence. See, for example, the Davidson & Flachaire reference for the wild bootstrap. (R. Liu (1988), Bootstrap Procedures Under Some Non-I.I.D. Models, Annals of Statistics, 16, 4, 1696-1708).**

• Lines 484-496: This whole paragraph could be cut. I suspect any reader of this paper will understand that watersheds are not true replicates, and that environmental conditions are variable.

***Response:* Although readers understand that watersheds are not true replicates, many statistical analyses assume that the sites are replicates. We feel that a contribution of this paper, in addition to documenting responses to riparian harvest, is to suggest that multiple analyses could provide more solid foundation of statistical responses, given the multiple assumptions of statistical designs and that watersheds are not replicates and data are not normally distributed.**

• Line 581-589: This paragraph seems very tangential to the stated goals of this paper and could be cut entirely or moved to an appendix.

***Response:* As suggested, we removed tile comparison and this paragraph.**

• Line 612: The subheading for this section is “conclusions and management implications”. The management implications are not clear in the text of this section, or elsewhere in the paper. Please clarify what a manager should do with the information presented in this study.

***Response:* We have rephrased this paragraph to clarify what a manager should do with the information presented in the study.**
• Line 617-620: I am not convinced that doing multiple statistical tests of the same data provides better information or more certainty for managers and policy makers. At the very least including this extra information makes the paper longer, and harder for a reader to understand and I don’t think that the added value is really there. I think the paper would be better with a single type of analysis or with additional analyses relegated to the supplement.

***Response:* We revised these lines to more fully explain our reasoning for presenting multiple statistical tests. Please see above for our responses to the prior similar suggestion.**

Minor issues:
• Line 1: The use of the phrase “The paradox” doesn’t feel like it is lived up to by the body of the manuscript. I think the phrase sets an expectation that 1.) This is a commonly observed phenomena, and 2.) You provide evidence that explains how this happens. I suggest a simpler title: “No in-stream algal response to forest and riparian zone management despite increases in light and nitrate”

***Response:* Thank you for this thoughtful comment. We removed “paradox” from the manuscript including the title.**
• Line 39-41: I don’t really understand why the community being dominated by diatoms would explain the results.

***Response:* Sorry that it wasn’t clear. We revised the sentence to include that green algae were not abundant.**
• Line 41: Is the word “dynamics” needed here?

***Response:* No, it isn’t and the sentence was revised.**
• Line 59: The topic sentence suggests that this is a list of ecosystem services provided by riparian zones. Ecosystem services are the benefits to humans provided by an ecosystem. Much of this list is benefits of riparian zones to streams which are only loosely tied back to humans in the last sentence of this paragraph. I suggest dropping the phrase “ecosystem services” from this paragraph.

***Response:* Thank you for this suggestion. “Ecosystem services” were dropped and replaced by “ecosystem functions”.**
• Line 31: You use the word “paradigm” in the abstract to describe the expected increase in nutrients, light, and shift towards autotrophy. However, on lines 79-80, and lines 87-89 you list numerous studies have found contrary findings. If there are already more than half a dozen studies that contradicted what you describe as the current “paradigm” I am not sure it is correct to describe it as a paradigm at all.

***Response:* That is an interesting philosophical question about terminology. I wonder if we are using different definitions of paradigm? We looked up multiple definitions of paradigm and those definitions included: ‘a standard perspective or set of ideas; a pattern, especially an outstandingly clear or typical example or archetype’. In our field, I would say that this shift to autotrophy is being taught as a typical example (or paradigm), even though there are some published examples that contradict. Especially in our work with natural resource managers and teachers, we see it being used as an assumption and it shows up in classrooms, forest management plans and many gray literature documents. However, we accept your concern and have** **rephrased the title and revised the Abstract and Introduction to change how we mention paradigm.**
• Line 96: I am not sure what the phrase “dueling statistics” means.

***Response:* We are referring to the back and forth that occurring in the literature when authors rebut publications by other authors using differing analyses to yield differing results, followed by responses by original authors justifying the original analyses. There are multiple instances of this in literature around topics that have implications for resource management, such as the effects of logging on peak stream flows. Sorry for the jargon. We have revised these sentences and removed “dueling statistics”.**
• Line 104-117: The formatting of questions and hypotheses could be streamlined.

***Response:* Thank you for this suggestion. This section was streamlined.**
• Line 312: When you say “various measurements” in this sentence I don’t know if you mean measurements of different parameters within the same stream and date, or a single parameter among dates and streams.

***Response:* Thank you. We revised the sentence and clarified that sites were not independent.**
• Lines 485-486: I think there is an extra word in this sentence.

***Response:* Corrected**
• Line 502-504: Including self-citation of unpublished work is not necessary to make this point.

***Response:* These citations point to articles in the journal titled “*Forest Ecology and Management*” and are included here to support the statement about multiple trophic levels being studied at these same sites. We have included wording to make this connection more clear.**
• Line 499-502: Forest harvest increased nitrogen concentrations in both nitrogen limited and non-nitrogen limited ecosystems. If the response is the same why draw a distinction between nitrogen limited and non-limited?

***Response:* Revised to remove this distinction**
• Line 519-522: This should be modified slightly. The buffers could have taken up some nitrogen. Perhaps modify this sentence to say “Although not all transport of inorganic nitrogen…”

***Response:* Revised as suggested**
• When working with your data I had to refer back to the manuscript to figure out which sites had which treatment. It would be helpful if there were columns indicating treatments, and treatment periods in the data.

***Response:* The archived data in the USFS Research Data Archive are formatted as you describe. Tables 1, 3, and 5 had this information and it has been added to Table S4. Table 4 is now a figure.**

---------------------------------------------------------------------------------------------

**Reviewer #2:** The manuscript addresses a relevant issue about limiting primary productivity in headwater streams, often limited by light, facing deforestation. It has an exciting approach, including experimental deforestation and long-term monitoring, potentially responding to relevant ecological questions, and dissertating about environmental management.

I noticed a potential issue concerning the work's message that seems dissonant with the research findings—for example, assessing the standing stock of biomass or pigments is not a direct measurement of primary productivity. Net primary production is strictly defined as the difference between the energy fixed by autotrophs and their respiration, and it is most commonly equated to increments in biomass per unit of surface and time, e.g., g C m-2 d-1 (<https://www.sciencedirect.com/topics/earth-and-planetary-sciences/net-primary-production>).

- Could authors calculate NPP based on epileptic mass over time?

***Response:* We agree that reporting changes of biomass over time could be a valuable way to synthesize and inform changes in productivity. We acknowledge that algal stocks are not a proxy for productivity and we make this point more clear in text. Since we only have one measurement of biomass per year at each site, we are not able to calculate NPP based on changes in biomass per time. In streams, NPP is calculated from measurements of stream metabolism and respiration (*Methods in Stream Ecology*, Hauer FR, Lamberti GA, editors. Boston: Academic Press; 2017. doi:10.1016/B978-0-12-416558-8.00011-1) and is a very different metric than measurement of epilithic biomass or chlorophyll *a*. Although we do have data on in-stream metabolism (of which primary production is one component) and nutrient spiraling from the Trask River Watershed Study at a subset of the sampling times, we feel that the descriptions of metabolism, respiration, and nutrient spiraling and uptake rates are complex, lengthy subjects better suited to a separate publication.**

- NPP may not be associated with biomass or pigments. For example, epilithon/periphyton is a matrix formed by a stock of autotrophic and heterotrophic biomass. Whether the heterotrophic compartment decrease and the autotrophic one increases, the balance is no biomass gain, and other factors may influence autotrophic and heterotrophic proportion (Guariento et al., 2011, <https://link.springer.com/article/10.1007/s10452-011-9377-5>). In addition, it is known that primary producers can increase or decrease their cell proportion of pigments depending on light conditions (Ma et al., 2022, <https://doi.org/10.1016/j.biortech.2022.126777>).
  ***Response:* We agree and more fully describe epilithon as a matrix. We present chlorophyll *a* concentration to evaluate activity and greenness. We also note that autotrophic algal taxa did not become more common after harvest.**
- It is worth mentioning that monitoring data is precious, including the immediate and long-term impacts, and this research could show interesting time series. The way authors present 10 years of data pulled in before and after deforestation, using repeated measurements and ignoring system dynamics.

***Response:* Yes, it is challenging to conduct large scale studies over time. While deciding what to include in this paper, we explored year to year and variability of responses as a function of time since treatment. Only DIN showed some lags in responses and therefore we presented data by site and year in Table S4 for the two forms of dissolved inorganic nitrogen. However, with regard to time series we believe there are too little data for this approach. Our analyses provide validation statistics which we feel makes our results more robust; we added a reference that highlights the importance of cross-validation or test set validation (Breiman, L. 2001. Statistical Modeling: The Two Cultures, Statistical Science, 16, 3, 199-231), in addition to the existing Efron reference.**

- In addition, I believe that the manuscript should be restructured textually. For example, part of the assumptions is in the methods, and part of the methods is in the introduction. I find in the scientific literature a structure in which the introduction takes the scientific question and presents the hypotheses and the expected results.

***Response:* We agree that what you describe is optimal and we present our scientific questions and hypotheses in the Introduction. We double checked that methods are not in the Introduction. We mention some assumptions in the Data Analysis section because those are required to explain how we selected our analyses.**

- I also suggest including a sub-item about management and relocating textual sections accordingly.
  ***Response:* We have revised our statements about managers potential use of these findings, based on Reviewer #1’s comments.**
- Similarly to the first comment, it is unclear what processes were changed or not during the removal of the forest. What ecological processes and functions lead to changes and maintenance in the system? Is there evidence for what limitations of local N and P? Does species composition show any exciting pattern?
  ***Response:* We feel this paper provides foundational information about instream responses to forest harvest and clearly shows what changed and what did not change. We are not attempting to quantify the ecosystem processes here and we do not present primary production results. The rates and processes can be informative but in different ways than what we focus on in this manuscript. We also provide the taxonomic information for the most common species of diatoms and note that there were not substantial changes.**
- Finally, I believe the authors can tailor their questions to the results and respond to other ecological questions not addressed in this manuscript, such as biomass and species dynamics.

***Response:* We have revised the manuscript extensively, based on all three reviewer comments. We discuss epilithon biomass and algal species dynamics. Beyond that, I am not sure how we might “tailor questions to the results”? Or how or why we might “respond to ecological questions not addressed in this manuscript”? We revised our study questions and hypotheses in the Introduction and now they are more streamlined, as recommended, we fully discuss our finding related to these hypotheses in the Discussion section. We more fully address forest harvest effects on biomass and species dynamics for epilithon as well as responses of nutrients and carbon. Thank you for suggestions.**

----------------------------------------------------------------------------------------------

**Reviewer #3:** Thank you for giving me the opportunity to review this paper submitted to PLOS One. I think that the dataset analyzed in this paper has many strengths, including the longevity of the research with multiple years of pre-harvest controls, numerous watershed pairings, consideration of spatial complexities when studying stream ecosystems, extensive complementary datasets from a multi-disciplinary team, consistent annual sampling design, and algal composition data reviewed by a co-author who leads the field in diatom identification. While I could not speak specifically to the nuances of the partial least squares regression method due to my lack of familiarity with this type of analysis, I recognize the strength of using multiple non-parametric statistical methods to analyze field data characterized by a high degree of natural variability. Consequently, these data offer an important contribution to our understanding of how forest management affects in-stream primary producers and I sincerely hope that these data are published. However, I did feel that there were numerous and substantial issues with the paper as written that lead me to recommend that this paper be categorized as needing “major revisions.”

- My concerns with the paper tend to fall into three categories. First, while there were many strengths in the methodologies used in this study, I do not feel that the authors provided enough detail on the specifics of how algae were collected from the stream in the methods and did not do enough to recognize the limitations of some of the methods that they used in the discussion.

***Response:* We now more fully describe the sampling protocol for attached epilithon in the Methods, as you suggest. In the Discussion, we added text about the limitations of algae sampling so that readers would recognize that epilithon is of mixed origin, including autotrophic and heterotrophic materials, and spatially variable -hence the number of rocks that were sampled for each composite sample with multiple composite samples per reach.**

- Second, while many of the figures were relatively visually clear, I found some inconsistencies with labeling to make it difficult to compare among figures. There was also a substantial amount of information that I felt was missing from the figure legends pertaining to the statistical analyses. I also disagreed with the use of standard deviations for error bars when the authors calculated 95% confidence intervals as part of their statistical analyses (and are more easily interpreted for statistical relevance in bar graphs).

***Response:* Thank you for pointing out the inconsistencies. We clarified legends, regrouped panels and added figures as suggested (below). It would be nice to be able to incorporate the lettering of significance for watershed comparisons in each figure. However, we could not figure out how to include visual notation about significant differences at the treatment scale since what we are presenting are individual sites and because we have multiple significant tests. And the confidence intervals we calculated as part of the statistical analyses are for treatment groups. For watersheds, we feel that the variability in data for the pre and post-harvest periods is best shown as standard deviation. If we were to show standard error, we would be presenting the error around the means, not of the data. Interestingly, given our small sample sizes (n=4 or 6 years pre and post), the error bars for the standard deviation are almost identical to 95% confidence intervals because confidence intervals are calculated as standard error * 1.96. We added statements to the text that the current error bars are almost identical to 95% confidence intervals.**

- Finally, I felt that there were some substantial issues with the writing, particularly related to the framing of the data analysis and the literature used to support their findings. In particular, there were many areas of the writing that were too broad and drew from a broader literature source; focusing more specifically on riparian studies in headwater streams would allow the authors to make more direct comparisons and strengthen their claims.
  ***Response:* We appreciate your suggestions. We revised our framing of data analyses and narrowed the focus in the Introduction and Discussion to more specifically focus on harvesting and riparian studies.**

**Specific Reviewer Comments**

- 34: This description does not seem to account for the “thinned” treatment – is there a better way to write a succinct description of the treatments to make it clearer?

***Response:* Yes, good point and we revised to try to succinctly include treatment descriptions in the Abstract**

- 39: I am not sure you discuss the importance of the algal community enough to include it as a major point in your abstract as written. I would like to see you spend more time talking about the algal species composition in the paper to justify it here.

***Response:* As you suggest, we revised the sections about algal species composition within the paper and also reduced discussion of it in the Abstract.**

- 49: This may be more of a stylistic comment, but I am not sure I like the use of sub-titles within the introduction in a non-review style paper

***Response:* Subtitles were included to try to summarize main points of Introduction and Discussion. We feel they may be helpful to readers or we can remove them. We will defer to the Editor’s recommendations for this.**

- 53: I think your argument about the importance of studying harvest would be strengthened if you could provide some kind of estimate of the percent of forests harvested in your study region, the US or globally

***Response:* Good suggestion. We now include statements about the amount of forested lands across the US and percentage of forests where harvesting can occur to provide context for readers.**

- 54: This statement would also be strengthen with some citations about both the diversity of aquatic organisms and the use of headwater streams as upstream sources of water for downstream communities

***Response:* Yes, thank you for that suggestion. We added citations about aquatic biodiversity diversity and domestic use of headwater streams for drinking water.**

- 59: I think it would be helpful to have a more direct tie from the effects of harvest to the use of riparian buffers as a management strategy

***Response:* We added text about functions of riparian buffers and then we provide details in the paragraphs addressing management of riparian forests relative to these functions.**

- 62: Rather than saying “minimize algal blooms,” it seems this sentence should focus on the effect on light directly

***Response:* This sentence was revised as suggested.**

- 64: This part of the sentence is a little unclear: “Changes to any or all of these functions and ecosystem services” – Changes due to what? Functions and ecosystem services provided by forested riparian areas?

***Response:* Thank you for pointing out that this sentence was not clear. We revised it to and addressed ecosystem processes rather than services.**

- 70: I found this sentence to be very unclear – I was not sure what exactly you meant by “lower trophic levels of food webs” – Is this stream food webs or forest food webs? Is it primary producers or are herbivores also included? Is the focus here on removal of riparian vegetation or forest management at large? Because this is the first sentence in the paragraph, I am having a hard time figuring out the exact direction that this paragraph is going.

***Response:* We revised this sentence and narrowed the topic for this paragraph. We are focusing on aquatic ecosystems and responses to removal of riparian vegetation associated with forest harvest.**

- 73: You talk about the functions of the riparian buffer, but think it would be helpful in the paragraph above to talk about how the function of the riparian buffer vary with width – then this sentence will make more sense

***Response:* Good suggestion; revisions were included in previous paragraph.**

- 75: when you talk about stream parameter or process is of most interest, are you referring to protection of these processes as part of the establishment of riparian buffers?

***Response:* Yes, we revised as suggested.**

- 75: The focus on nitrogen in this paragraph seems to have not been a logically led in by the initial sentences in this paragraph. It might be helpful to start with this sentence, and then take the information from the first two sentences to make them more specific to nitrogen, since this seems to be the focus

***Response:* Good suggestion, we revised this paragraph to start broadly and then identify nutrient and sediment responses. (Nitrogen is not the only focus). Algal responses have been moved to their own paragraph.**

- 77: I would remove the information about fire-related disturbances, since you have narrowed to forest harvest in the first part of your intro

***Response:* We removed references pertaining to riparian disturbance by fire in the text.**

- 79: Again, I think focusing just on the effect of harvesting on nitrogen, rather than on “forest disturbance” would be more focused here

***Response:* We clarified that we are focusing on forest harvest and that we are studying responses of nutrients (N and P) as well as DOC, not just nitrogen.**

- 80: Will the general readership of PLOS one know the term “epilithon” or should it be defined?

***Response:* Good point; sentence was revised and algal biomass is used in the Introduction. We define epilithic algae/epilithon in the Methods and come back to the definition in the Discussion**.

- 80: Here is another place that I feel the writing is disorganized – the preceding sentences were about nitrogen, but then we jump to epilithon. It seems that this whole paragraph could be more focused by having a general subject of thinking about the effect of riparian buffers on abiotic conditions in streams, and then having a separate paragraph that talks bout the connection to the biotic

***Response:* Thank you, we revised and reorganized these paragraphs**

- 82: It seems like you should not include natural gaps in the canopy cover – it takes away the focus on riparian buffers to protect from harvest

***Response:* We removed natural gaps.**

- 83: It might make sense to first start by talking about the expected effects of nitrogen and light on biomass of epilithon, then get into the taxonomic effects

***Response:* We now start this paragraph referencing increased biomass.**

- 85: I have a problem with starting the sentence by saying that light changes composition of algae in the stream, but then supporting that with clearcutting (because there are many other abiotic factors that are affected by clearcutting)

***Response:* We revised this section to include nutrients and light, as supported by the citations.**

- 87: Can you be more specific on the ways that diatoms and filamentous algae affect consumers? Talking about which group is more palatable and/or which consumers benefit or don’t benefit?

***Response:* Because we are being encouraged to shorten this paper, we don’t feel that we should go into those details, which are off topic for this study. We include this sentence about food web responses to help readers have context that the type of algae matters.**

- 89: I think that the last sentence about not supporting the common paradigm is a bit bold, and think maybe there should be more support provided to support this

***Response:* We revised the Introduction and now mention paradigm as part of a citation about null findings being less frequently published.**

- 90: What do you mean by “this complexity of responses”?

***Response:* We added clarification that we are referring to multiple instream responses to forest harvest.**

- 91: I wonder if the issue is not so much the focus of resource managers and research on statistically significant findings, but rather the inflexibility of commonly used statistical tests to capture biological variability and significance in dynamic systems like streams

***Response:* Thank you for suggesting a more clear way to say this.**

- 95: I think that you need to be a little clearer than stating “may not be supported” – Do you mean that the assumptions might not be met? That the methods might not be appropriate?

***Response:* Yes, good suggestions. We revised as suggested.**

- 96: Can you be more specific in what you mean by “dueling statistics” Do you mean using multiple statistical approaches?

***Response:*** **Yes, we are referring to the back and forth that occurring in the literature when authors rebut publications by other authors using differing analyses to yield differing results, followed by responses by original authors justifying the original analyses. There are multiple instances of this in literature around topics that have implications for resource management, such as the effects of logging on peak stream flows. Sorry for the jargon.**

- 96: I think you lost me on this final sentence – it seems to relate more to how managers manage riparian vegetation, rather than its relationship to the discussion about statistics

***Response:* We revised this sentence and this section so that the paragraph addresses statistics more directly and not how managers deal with riparian vegetation. Thank you.**

- 101: I am not sure what you mean by “extent” of riparian buffers – do you mean width? Length? Quality?

***Response:* We removed ‘extent’ and replaced it with width.**

- 101-104: This sentence is a little long, and might benefit from being shortened or turned into two sentences. Also, are you just measuring standing stock of algae or composition as well? If not measuring composition, I think that information can be removed from above.

***Response:* We are evaluating community composition as well as standing stock of algae. We simplified this sentence.**

- 104: Again, because you don’t specify what you mean by “extent,” in the first sentence, it is hard to know what you mean by “more removal” of riparian vegetation

***Response:* We revised and clarified this sentence**

- 105: I think it would be better to separate the effect on light and the effect on algal biomass

***Response:* We think of these as related. It is generally well accepted that removal of riparian vegetation changes light to a stream and what we are exploring in this paper are the related responses - or not- of epilithon.**

- 106: I would rephrase this part of the hypothesis to start with the effect you expect on nitrogen and then say why

***Response:* We revised all the hypotheses as you suggest so that they all have similar structure.**

- 108: I am a little confused here because you talk about nutrients and dissolved organic carbon, but the intro really just focuses on nitrogen. Which do you measure?

***Response:* We measure nutrients and DOC in the streams. We added mention of responses of dissolved carbon to forest harvest in the Introduction.**

- 108: You don’t really discuss sediment at all in the intro, which I think is problematic to bring it in here in the hypothesis

***Response:* We now have expanded discussion of sediment responses in the Introduction.**

- 111: Is this dissolved carbon or are you measuring other forms of carbon? I also think you could change this to be one sentence by saying “Do forested riparian buffers reduce increases in in-stream concentrations of dissolved organic carbon and nutrients in harvested forest watersheds?”

***Response:* Thank you, we clarified that we are studying DOC. And because we simplified this section, we no longer have the question format.**

- 114: This question also does not line up with anything in the introduction or in the hypotheses, because you don’t mention temporal or spatial extent anywhere else.

***Response:* As suggested by previous reviewer, we removed the questions to simplify this final section of the Introduction**

- 116: Should this say “chlorophyll a”?

***Response:* Yes, corrected.**

- I also don’t think you can bring in light here if you don’t mention light as something that you are directly measuring as a question.

***Response:* This section of questions were deleted.**

- 120: Should there be a reference to a figure at the end of this sentence?

***Response:* Reference to Fig 1 added**

- 128: Rather than saying “prior disturbances, including…” I think you should just say “disturbance due to wildfire” because you don’t give examples of any other types of disturbances

***Response:* We removed ‘including’. The next sentences note additional disturbances from forest harvest and replanting and roads.**

- 129: I think it would be helpful to know the extent of this burning and logging within the watershed – ideally a map, but even just report of percentages would be helpful.

***Response:* We edited this slightly. The extent of burning and logging was 100%, hence the vegetation was 45-60 yr old at the start of the study.**

- 136: did you evaluate downstream effects in this study, or was this just an objective of the overall study?

***Response:* The study of headwater and downstream responses occurred across all of the Trask River Watershed studies (stream temperature, discharge, sediment, nutrients, algae, stream metabolism, insects, instream vertebrates)**.

- 138: Were there 12 treatment and 12 reference or 6 treatment and 6 reference streams?

***Response:* We revised this to remove confusion.**

- 140: Were there riparian buffers left in these? Since this is a key point in the study, it seems like it would be good to mention it here

***Response:* The next paragraphs go into extensive detail about where riparian buffers were retained.**

- 146: I found it really difficult to read the legend on your figure. I am also not if you need both the satellite image and the map here. I also think it is problematic to introduce the reader to your treatment types for the first time in the Figure legend. I don’t know what you mean by “variable buffers’ or “uniform buffers” – a more specific description of the buffers that were left and the harvesting strategy would be helpful. Also in your figure legend, it would be helpful to give context for the 2013 year (first year post-harvest could be added).

***Response:* We revised this figure and the legends should be clearer in the new upload. We provided context for why the photo from 2013. This figure serves multiple purposes and the paragraphs following the figure go into extensive detail about our study design. Because this figure serves to show the location of the study watershed in western US, we cite it at the beginning of Site Description, but we could remove the reference to Fig 1 from the beginning of the Study Description? However, wherever it is first cited, there will be the issue of more descriptions following the presentation of the figure.**

- Table 1: I think it would look better in the harvest treatment to remove the underscore from the description. Do you think it is necessary to state the ownership of the watershed? You might instead just want to indicate if it was private or public. Also, is it possible to rename your watersheds so that all of the references are 1 and the clearcuts are the remaining numbers? I know this might not be possible if you are working in a large group. Did you run any statistics to compare the differences among your reference and treatment watersheds to make sure they were similar in some of the physical characteristics? Also, what is a “15m no riparian buffer?”

***Response:* We revised this table and all others to remove the underscore in the treatment names. We include the missing word in the description of the thinned watershed. We changed the ownership columns also – thank you for that suggestion. We do not want to renumber the watersheds because these are the names being used across all of our Trask publications. We did not run statistics in advance of the decision for which watersheds would be harvested and which would be reference; these decisions were made at the beginning of the study and based on harvest guidelines, adjacent treatments, and with input from our research and management group. However, in our PLS analyses, the pre-post predicted responses can be compared within a given watershed as well as compared across all the watersheds for each response variable. Therefore one can compare the post-hoc lettering across watersheds and identify which watersheds have unique lettering and therefore are significantly different.**

- 151: I am a little unclear as to whether these guidelines apply to the private or public lands or both

***Response:* The guidelines differ for state and private forests and we clarified this in the text.**

- 153: Can you provide a definition of “leave trees” in the prior sentence by using this term so that it is more familiar when you state what happened in the UM2 and UM3 watersheds

***Response:* We removed the term leave trees and further explained that these were trees identified for wildlife and required to be left unharvested.**

- 156-158: A few points of clarification about this treatment are needed – the first is how “perennial stream” is being defined by the logging company and if that is consistent with aquatic ecologist definition of the streams in this area. It is also unclear how even the 12m of buffer were in this treatment – were they really even enough to be considered “uniform buffers”?

***Response:* Perennial streams are defined as having flowing surface water all year long. This is a consistent designation across forest ownerships. The uniform buffers are termed that because they were amazingly consistent ribbons of trees around the streams on state lands. This can be seen on the aerial imagery in Fig 1 or in greater detail in the supplemental material with a prior paper: 10.1016/j.foreco.2021.119999.**

- 161: It would be helpful to start this sentence by stating if this watershed is a treatment watershed.

***Response:* Sentenced was revised as suggested.**

- 167: I feel like this sentence is written as if you have not just described the watersheds, rather than stating that these were within the watersheds you just described.

***Response:* This sentence was removed and the paragraph edited to be clearer.**

- 167: I do feel I need to question whether 30m is a long enough reach to make these measurements

***Response:* These reach lengths were selected based on EPA sampling strategy of the study reach being 11 times the width.**

- 189: Do these streams have any crayfish that could influence algae?

***Response:* We initially were interested in studying crayfish as part of this broader study, but only a few crayfish were able to be located during the pre-harvest sampling across all streams. No juga snails were present either.**

- 191: I don’t think you need to list the physical characteristics that you measure, because you then go on to describe each of them.

***Response:* The list was deleted.**

- 193: Is this an n=4 measurements in a 30 m reach?

***Response:* Yes, we clarified that measurements were taken at transects and that transects for densiometer and other metrics were evenly spaced through the reach.**

- 200: I am assuming based on the sampling time selected in June that there was complete canopy closure at this time for deciduous trees, but I think it would be helpful to clarify this, particularly for the higher elevation sites

***Response:* Yes, the sampling in middle to late June occurred with full leaf out. The high elevation sites in the Oregon Coast Range are not very high - leaf out occurs generally in April.**

- 206: Did the downstream measurements go upstream of the site or downstream of the site?

***Response:* We clarified that we sampled at the most downstream section of each site. Site characteristics were measured upstream from the sampling locations.**

- 206: Do you ever address the hemispherical photo data later in the results or compare it with the densiometer data? I don’t remember seeing that information.

***Response:* We graph the hemispherical data in Fig 2 because it showed clear characterization of changes in light at the streams pre- and post-harvest, which was relevant to algal dynamics. Hemispherical data are much more intensive to collect than densiometer data and therefore we only have hemiview data from 2008 and 2013. Densiometer data, which quantify canopy closure, were collected at each sampling time and location and the reach averages were analyzed for changes pre and post.**

- 211: I am not sure what you mean by “percent total site factor”

***Response:* That is a metric within the hemiview software that land managers frequently use and we revised the text to clarify this.**

- 212: Did you take all images on a cloudless day to enable the digital image analyzer, or does this analysis program allow you to account for things like cloud cover?

***Response:* We took all images at dawn or dusk and on cloudless days, which are common in summertime in western Oregon.**

- 213: This last sentence feels very out of place after a whole paragraph about canopy cover

***Response:* Yes it seemed out of place and we moved this sentence to earlier in the paragraph.**

- 222-226: I think it is worth mentioning here again that if you are focusing on nitrogen, phosphorus, and dissolved organic carbon as drivers, these all should be mentioned somewhere in your introduction

***Response:* We revised the Introduction to include discussion of all these analytes.**

- 229: I would like to see a little more description made about how the algae was collected How were the site for algae collection selected within the stream reach? Why did the number of rocks vary per site? Were they in pools, riffles? Were they collected at the same location as the sediments? Did you calculate the area of the rocks that you used? What method did you use to calculate rock area?

***Response:* We revised the text to clarify our algal sampling protocol, including that rocks were randomly selected, that templates were used to limit the area scrubbed and that the total area scrubbed per composited sample was noted. These small streams are not easy to classify as distinct riffles or pools. Sediment samples were collected randomly within the same reach but not where rocks has been disturbed.**

- 242: I think it would be helpful if you clarified where these algal structure data were collected relative to the other samples for biomass

***Response:* We clarified that these data were randomly collected.**

- 245: I am wondering if it is problematic that your only taxonomic analysis occurred in the first year post-harvest and not in subsequent years as well give how much disturbance there likely was in the first year.

***Response:* While we would have liked to have the taxonomic information for each year, these analyses were expensive and time consuming. If there was a impact of the harvest, we would have expected to see it the first year, give the amount of hillslope disturbance.**

- 254: I think that you should cite all of the resources used for the diatom analyses, rather than as an “e.g.”

***Response:* Corrected as suggested**.

- 257: I think that it would be better to put this additional sampling up by the section where you talk about biomass since it doesn’t sound like you measured algal composition on these additional samples

***Response:* This section was moved as suggested.**

- 277: I think that you should not use KS2, but either write out the full name of the test or summarize what you are doing (or take out subtitle)

***Response:* We removed the abbreviation.**

- 279: I think you need a comma after “thinned” to distinguish it is a separate group from reference

***Response:* Added as suggested.**

- 298: I don’t think that treatment group, harvest period, etc should be capitalized here

***Response:* Capitals removed.**

- 330: One question that I have is how you treated replicates within each of these statistical analyses – did you treat multiple samples from a stream as pseudoreplicates?

***Response:* Pseudoreplication with field studies is a concern. Our multiple samples within a stream were composited for a site average per year. The annual data were then evaluated in PLS models, using non-parametric wild bootstrap and semi-parametric Wu jackknife for significance testing. These methods guard against the effects of heteroscedasticity and non-independent (non-i.i.d) data. We added 2 more references that might help clarify this: R. Liu. 1988. Bootstrap procedures under some non-I.I.D. models. Annals of Statistics 16: 1696-1708; Liu, R. Y. & Singh, K.1995. Using i.i.d. bootstrap inference for general non-i.i.d. models. Journal of Statistical Planning and Inference 43: 67-75.**

- 337: A question I now have looking at your discharge measurements – did you have any requirement for recent rain event when you did your sampling, or did you just sample regardless of flow conditions? This might be helpful to clarify in the methods

***Response:* We noted in the text that we had criteria to avoid rain events for our sampling. With year to year differences in overall weather, the flows varied at the time of sampling at the end of June.**

- 343: I think you should report the magnitude of the temperature increases in the text of the results section. Also, were these part of your statistical analyses?

***Response:* June temperatures increases post-harvest are now explicitly presented in Results. Temperatures were not included in our statistical analyses because summer temperature responses from the Trask River watershed treatments have been reported elsewhere (Reiter et al. 2020, Bladon et al 2018).**

- 345: If you are going to talk about temperature first, then I think this should be the first sub-panel in Figure 2, rather than the second (since you haven’t yet referenced 2A).

***Response:* We also reversed the order of our discussion of temperature and light in the text.**

- Figure 2: I think this figure would be improved with better labeling. For example, for the treatments, it would be helpful if you had the width of the riparian buffer in parentheses next to the treatment name. Also, are the downstream sites here? If not, you might want to include them and/or specify this is only the headwater stream sites. I am also unclear as to why the light measurements do not have error bars. Didn’t you take multiple measurements? Also, where is there a graph or table showing the difference in sediment among the sites? I think this would be very helpful to have graphically.

***Response:* We revised Fig 2 and included error bars. We duplicated the names of treatment groups within the figure, and included riparian widths for uniform and thinned sites. We could not include buffer widths for the variable treatment because they were not consistent Sediment data are included as a graph in new Fig 3.**

- Figure 2 caption: I think there are a number of issues with this caption. First, there is no indication of the sample size or statistics for each measure. Second, I personally prefer the use of standard error or confidence interval rather than standard deviation for comparing means. Didn’t you calculate the 95% confidence intervals for each of these groups using the bootstrapping method? Couldn’t you plot those here?

***Response:* We revised Fig 2 caption, clarified that these are only headwater sites. The sample sizes are detailed in the methods and differ for treatment groups or watersheds. For watersheds, we feel that the variability in data for the pre and post-harvest periods is best shown as standard deviation. If we were to show standard error, we would be presenting the error around the means, not of the data. And given our small sample sizes (n=4 or 6 years pre and post), the error bars for the standard deviation are almost identical to the 95% confidence intervals in our current bars if confidence intervals are calculated as standard error * 1.96. We added statements to the text that the current error bars are almost identical to 95% confidence intervals.**

- Also, you say in the description for A that you calculated incoming radiation, but I don’t think that is quite correct – usually radiation is measured using a light meter, but you just measured canopy coverage vs. no coverage.

***Response:* The light metric was calculated using industry standard software from our hemispherical images. Note that we are not reporting radiation but percent light. The annual canopy coverage data was collected using a densiometer and you are correct, that measure is only percent coverage or its inverse percent shade. We clarify this in the text.**

- Table 2: I think the bold text is a little hard to read – could you use an asterisk instead? I also found the symbols for the PLS model to be a little bit confusing.

***Response:* Asterisks were included for significance. The PLS model comparisons are not meant to be confusing; they show if the models were different where the lettering is unique before and after harvest (B/C). They also show the magnitude of the predicted model value across all treatments: A being largest value, C smaller, etc. So where the response was B/C, the values were smaller post-harvest.**

- Figure 3: I think it is important to make your labels consistent between Figure 2 and Figure 3.

***Response:* These have been revised. The collection period for the hemispherical data once pre and once post – and therefore the labels have to be different.**

- 365-374: I think that logically it makes more sense to talk about DOC, SRP, and DON in the same order in the text as they appear in the figure. I also think it is important to include your n somewhere in the text for reference.

***Response:* The order of discussing the panels is now sequential as suggested. We clarified that these samples are from annual sampling during early summer and the years are in the legends.**

- Figure 3 caption: I think it is also important for you to include more of your statistics in the figure caption as well, particularly the n, the p-value you used from one of your tests. Same comment about the use of 1 standard deviation here compared with the value of 95% CI or standard error.

***Response:* I know the type of figures you are suggesting – where lettering of significant differences are across the top of the bars. I tried this and while it could work for a single panel figure, many of our figures are multiple panels and the figures get too complicated. The lettering of significance has to be very small and its confusing with multiple panels. If we split our figures to be single panels, then we would have way too many figures. I included asterisks and hopefully they don’t disappear in the complexity of images. We note in the caption that results of statistical tests for the headwater watersheds are in Table 3. See above for our response about type of error bars.**

- 377-382: One question that I have is how these are varying from one year to the next post-harvest – is that what can explain the large variability in the error bars? I would predict that the DIN would initially be high, but then decrease as more vegetation grows in.

***Response:* We show each year’s nitrate and ammonium concentrations in Table S4. Actually, nitrate appeared to increase with time since harvest, but because our sampling period covered only 4 yrs post harvest, we did not evaluate the significance of this trend. Vegetation recovery was limited over these 4 yrs, even though replanting of small conifer trees did occur in year 2 (as required by harvest regulations.) We do highlight and discuss the timing of max DIN in the text now.**

- Table 3: I think that you need to have a better description of epilithon here, as it does not indicate that what you are measuring is AFDM. I also think that generally, the information presented from the PLS would be better as a graph, rather than as a table, because you could visualize how different groups move from one to another. You couldn’t do this for all variables, but maybe you put this table in your supplementary materials and make a graph with only the most interesting of the points you want to illustrate.

***Response:* We added ‘biomass’ to the column headings to clarify what are the metrics for epilithon. In the interest of space and presenting all the data, we feel that the table is the most concise presentation of the results. We added statistical results to the graphs where possible.**

- 394: You bring in the molar ratios of DIN: SRP here, but I think this is the first time that you talk about it – why might this be important to consider? If you think it is important, maybe highlight this point in your introduction.

***Response:* We revised to mention molar ratios in the Introduction.**

- Table 4: I am wondering if table 4 should be a fifth panel in the other panel about water quality – this is very hard to visualize looking at it in a table, and it seems that tables should be reserved for providing the output of statistical tests, rather than means and SD.

***Response:* We now present the molar ratios as part of figure 4.**

- Figure 4 – I think it would be helpful to indicate which downstream site is the reference and separate it out from the other three sites visually. Also, were each of these sites mixed in terms of the upstream harvesting, or could they also be grouped by the harvesting method?

***Response:* Now Figure 5. Good point. We now identify RK as the reference. Some sites were mixed in terms of harvest method, some were a single method. We include this information in the caption.**

- Figure 4 caption: This caption has the same issues as the other ones – there are no statistics provided or indication of n.

***Response:* Now figure 5 - The figure captions have been revised to be standard across the figures and the figure legends now clarify that what are presented are annual averages during early summer sampling, with the years noted. The statistics for downstream sites were conducted as treatment groups, not for individual sites, so including the statistics on a figure showing individual sites is rather complicated. Plus we have three different statistical tests to report.**

- Figure 5: Same issue with consistent labeling with previous figures. Figure 5 caption: Same issues as prior captions

***Response:* Now figure 6. The figure captions have been revised to be standardized and figure legends clarified. We tried to include lettering of significant differences across the top of the bars and while it could work for a single panel figure. The lettering of significance has to be very small and its confusing with multiple panels. If we split our figures to be single panels, then we would have way too many figures. I included an asterisk (there is only one significant difference – its an Epilithon reference site). We note in the caption that significance presented is in Table 3**

- 437: I think it would be helpful if you moved this section with chlorophyll a correlations to a new paragraph, since it is referring to different analyses

***Response:* It is now a new paragraph.**

- 438: One thing that I think is a bit misleading here is that you correlate “light reaching the stream” with algal concentration – but the problem is that your canopy cover measurements were not taken at the same locations as your algae measurements. I think you need to clarify this point by saying “mean reach-scale canopy cover” to indicate that it is representative of the whole reach.

***Response:* Good point that these metrics are reach averages and we clarify this in the text.**

- Figure 7: I am not sure that I find the data presented here to be very convincing or helpful, and I think the tile study should be eliminated (or just mentioned briefly without a figure). It appears to me that the tiles were substantially lower, which suggests to me that either the incubation time was too low, or that there was an issue with the estimate of rock area potentially. It also is not as helpful as it could be because these measurements were only made once post-cutting, instead of clear cutting.

***Response:* Our comparison of biomass on tiles was removed. July samples of epilithon and chlorophyll a are now only in the Supplemental material and we briefly reference it in the discussion. We checked and the area of estimation was correctly calculated in the same manner as for all other estimates.**

- 445-449: It was a little unclear to me if the diatoms you discuss here were found pre-harvest, post-harvest, or both – I am particularly confused by the line starting on 448. It seems to me that this part of the results deserves much more attention and description. Also, rather than just stating the most common taxa, it seems like you should do some kind of indicator species analysis to have a better way of making a cutoff of important taxa. Also, could you give some indication of the type of diatoms these are in terms of their growth form in the table, and then use this as a basis of discussion in the discussion?

***Response:* We revised the sentence to clarify that diatoms were most common algae in the pre- and post-harvest periods and added additional information about growth forms of the dominant diatoms in the Methods, Results, Table, and Discussion. This algal taxa dataset is too small for meaningful ISA analysis.**

- 453: I am a little confused by the line that starts here because you say “they” and I am not sure to what group this refers.

***Response:* Sorry for the confusion. We revised and clarified this section.**

- Figure 8: there is the same issue with labeling and captioning here.

***Response:* Figure 7 now. Figures and captions are revised. However, we could not include statistical findings in this figure, because the statistics were only conducted on the treatment groups at downstream sites pre- and post-harvest, not individual sites.**

- 463: I am not sure if you can make the argument that light limitation has been eliminated in these streams, particularly in any of the streams besides the variable buffer, as these were the only streams that showed a real increase in the amount of light

***Response:* We recognize that light did not increase at all harvested sites. The light limitation statement refers to our expectations from the literature, not these specific streams.**

- 467: I think this sentence is a bit misleading because you had riparian buffers in all treatments, it is just that some were more variable and some more uniform

***Response:* We revised this sentence to explicitly note that all clearcut watershed had increases in DIN.**

- 461-471: I think that the first paragraph of the discussion could be a little bit stronger by increasing the organization and making it more succinct

***Response:* We revised this paragraph to make it stronger and more succinct.**

- 473: I know that a main point that is being made in this paper is the challenge of distinguishing statistical significance in landscape scale experiments, but this feels like it could be a concluding section

***Response:* We debated where best these paragraphs fit into the Discussion – thank you for your input.**

- 484: I think this paragraph should come before the first paragraph in this section, as this one starts broader and the last one is specific to your study

***Response:* Good suggestion, we switched the order of the paragraphs**

- 487: I think you could include more references here to support this statement

***Response:* We added more references.**

- 488: This sentence is not very clear, because I am not sure where the similarities end and the differences begin

***Response:* We revised this sentence to better distinguish what are similarities and differences.**

- 493: I think that this brings up some good points about the difficulties of field sampling, but I think in the context of this paper it strays a little bit far from the focus on algae

***Response:* We revised and with our focus being more than algae, we suggest that discussion of broader issues are relevant for this manuscript.**

- 500: I brought this up earlier, but I think that it is better to limit your discussion to forest harvest, rather than bringing in disturbance more generally

***Response:* Thank you - we revised this.**

- 503: You again bring up the multiple trophic levels aspect, but you don’t really talk about what trophic levels you mean

***Response:* We removed the phrase multiple trophic levels. With numbered citations, it is not easy for readers and reviewers to see what studies are being referenced. The prior citations pointed to Trask macroinvertebrate and fish research findings.**

- 505: You might want to state why this is the case about nutrients

***Response:* We added citations and some text to clarify. However, the multiple reason for seasonality of nutrient dynamics are beyond the scope of this paper.**

- 511: It feels broken up to talk about being surprised by the finding of the lack of effect of riparian buffers here, and then moving paragraphs to continue talking about them. Also, it would be helpful for you to compare these values to the literature of studies that did not have any riparian buffer to support your claims.

***Response:* We revised, and now the discussion of riparian buffer influences on nutrients is in one paragraph.**

- 523: I think that these first two sentences get a little off topic, when it should focus more directly on downstream effects on DIN. In fact, I think you could shorten this paragraph substantially and add it onto the last paragraph.

***Response:* We moved this section up to the previous paragraph as suggested and revised and shorted the discussion**.

- 531: I like that you address a lack of effect on the other water chemistry, but I also think this paragraph could be written more concisely

***Response:* This paragraph was revised to be more concise as suggested.**

- 549: I am concerned that you say that increased nutrients have long been recognized to stimulate autotrophic responses in aquatic ecosystems, but only cite one reference. I think this would be a stronger statement if you included more references and focused only on headwater streams.

***Response:* There are many references for this and we included several more.**

- 557: So how can you explain the lack of response of chlorophyll in PH2 and PH3 if they have the required ratios of N:P?

***Response:* That is exactly one of our questions. We think that the lack of green algae in this system is part of why we did not see responses of chlorophyll.**

- 561: Trask River Watershed?

***Response:* Yes, thank you, we clarified this.**

- 565: The sentence starting here is a bit long and run-on-y. Also, I think that this argument would be stronger if you could provide evidence of to what temperature the stream would need to warm in order for the stream to shift from cold-water diatoms to filamentous algae

***Response:* Thank you, the sentence has been shortened and temperature more clearly addressed. Much remains unpublished about thermal thresholds for diatom taxa. However, the shift we observed in temperatures was apparently not enough to result in changes to the dominant diatom taxa.**

- 572-574: I think you should talk more about the specific species you found and how they may or may not have responded to nutrients

***Response:* We now include additional text and citations about the diatoms observed in these streams. An abundant diatom in our streams is considered to ‘successfully compete at low resource (light and nutrients) and high physical stress levels’ Biggs et al. 1998.**

- 581: Based on my comments above, I think this paragraph is more distracting than helpful to your paper.

***Response:* As suggested, we removed this paragraph***.*

- 597: I am glad you did talk about grazers, but I am concerned that the role of grazers has been dismissed to easily, and more literature should be brought into this part of the discussion.

***Response:* We now include more literature and explicitly note that grazing snails were totally absent from these watersheds and that crayfish were rare. In these forested ecosystems without filamentous algae, the crayfish tend to be detritivores**.

- 621: Evidenced?

***Response:* Opps, thank you.**

- Discussion generally:
  - I think that there is not enough attention given to the role of light and grazers. There was an increase in nutrients, but light really only increased substantially in one site.

***Response:* In our revisions, we worked to emphasize the changes in light as a function of annual closure measurements. Tables 2 and 3 show that canopy closure was significantly different pre- to post- for the Clearcut variable group and significantly different pre to post at all three watersheds that comprise that treatment group. Grazers are discussed, and we note that they were not a major component of the aquatic food webs in these streams.**

- I also think more attention could be played to the role of sediments in these streams.

***Response:* We included analyses of sediment because changes in sediment could impact algal standing stocks and chlorophyll *a*. Because instream sediment did not change after harvest, we noted that it did not likely have a role in suppressing epilithon responses.**

- There is also no explanation for why chlorophyll a seems to mostly decline in the reference streams while remaining the same in the treatment streams. Another concern that I have is that even though they noted increases in nutrients and light, I wonder if the magnitude of these changes was enough to change the algae.

***Response:* These are good questions. Other studies have found algal responses with much less change in light and in nutrients. As to post-harvest declines in reference sites, we now include our speculations that chlorophyll a concentrations decreased in reference as well as treatment sites because the post-harvest period was slightly warmer, dryer with somewhat lower streamflows than during pre-harvest period.**

- I also think that there does not seem to be an attempt to look at any of these factors as a function of time, which would be expected to change as new vegetation grows in and could account for variability.

***Response:* We addressed this comment above and noted that highest nitrate concentrations did lag the harvest. However, four years post-harvest is not sufficient for time series or much revegetation regrowth and we did not conduct analyses of it.**

- Finally, I think one of the most interesting things in this paper was how variable the chlorophyll a was in these streams under pre-harvest conditions was highly variable – in fact much more so than in many of the post-streams.

***Response:* Yes, it is interesting how different these streams were in the pre-harvest period, even though they had many similar conditions, including harvest history, light, vegetation cover and proximity.**

While revising your submission, please upload your figure files to the Preflight Analysis and Conversion Engine (PACE) digital diagnostic tool, [https://pacev2.apexcovantage.com/](https://nam04.safelinks.protection.outlook.com/?url=https%3A%2F%2Fpacev2.apexcovantage.com%2F&data=05%7C01%7Csherri.johnson%40oregonstate.edu%7C840e28fe26ef484d8c8708dab90105da%7Cce6d05e13c5e4d6287a84c4a2713c113%7C0%7C0%7C638025711599038822%7CUnknown%7CTWFpbGZsb3d8eyJWIjoiMC4wLjAwMDAiLCJQIjoiV2luMzIiLCJBTiI6Ik1haWwiLCJXVCI6Mn0%3D%7C3000%7C%7C%7C&sdata=KKM7SP8wC0JiMVFBUDgAY84oG92EmikyXylaqIxK520%3D&reserved=0). PACE helps ensure that figures meet PLOS requirements. To use PACE, you must first register as a user. Registration is free. Then, login and navigate to the UPLOAD tab, where you will find detailed instructions on how to use the tool. If you encounter any issues or have any questions when using PACE, please email PLOS at [figures@plos.org](mailto:figures@plos.org). Please note that Supporting Information files do not need this step.
