# Supplementary material for: Stream nitrate enrichment and increased light yet no algal response following forest harvest and experimental manipulation of headwater riparian zones
Source: PLoS One. 2023 Apr 20;18(4):e0284590. doi: 10.1371/journal.pone.0284590 (PMC10118188; doi:10.1371/journal.pone.0284590)
Supplement: Attachment — Submitted filename: Response to Reviewers_PONE_March2023.docx [file pone.0284590.s010.docx]

**Response to Reviewers Letter**

PONE-D-22-25727R1

Stream nitrate enrichment and increased light yet no algal response following forest harvest and experimental manipulation of headwater riparian zones

PLOS ONE

Dear Dr. Johnson,

Thank you for submitting your manuscript to PLOS ONE. After careful consideration, we feel that it has merit but does not fully meet PLOS ONE’s publication criteria as it currently stands. Therefore, we invite you to submit a revised version of the manuscript that addresses the points raised during the review process.

Please submit your revised manuscript by Apr 14 2023 11:59PM. If you will need more time than this to complete your revisions, please reply to this message or contact the journal office at [plosone@plos.org](mailto:plosone@plos.org). When you're ready to submit your revision, log on to https://www.editorialmanager.com/pone/ and select the 'Submissions Needing Revision' folder to locate your manuscript file.

Please include the following items when submitting your revised manuscript:

- A rebuttal letter that responds to each point raised by the academic editor and reviewer(s). You should upload this letter as a separate file labeled 'Response to Reviewers'.
- A marked-up copy of your manuscript that highlights changes made to the original version. You should upload this as a separate file labeled 'Revised Manuscript with Track Changes'.
- An unmarked version of your revised paper without tracked changes. You should upload this as a separate file labeled 'Manuscript'.

If you would like to make changes to your financial disclosure, please include your updated statement in your cover letter. Guidelines for resubmitting your figure files are available below the reviewer comments at the end of this letter.

If applicable, we recommend that you deposit your laboratory protocols in protocols.io to enhance the reproducibility of your results. Protocols.io assigns your protocol its own identifier (DOI) so that it can be cited independently in the future. For instructions see: [https://journals.plos.org/plosone/s/submission-guidelines#loc-laboratory-protocols](https://gcc02.safelinks.protection.outlook.com/?url=https%3A%2F%2Fjournals.plos.org%2Fplosone%2Fs%2Fsubmission-guidelines%23loc-laboratory-protocols&data=05%7C01%7C%7C52c97a66b9d34c93092808db199dc27f%7Ced5b36e701ee4ebc867ee03cfa0d4697%7C0%7C0%7C638131938149720049%7CUnknown%7CTWFpbGZsb3d8eyJWIjoiMC4wLjAwMDAiLCJQIjoiV2luMzIiLCJBTiI6Ik1haWwiLCJXVCI6Mn0%3D%7C3000%7C%7C%7C&sdata=BLh8abYvIB1Y0kbhXExjQ%2BgOtSWeo3pbuQCoT3dwM8U%3D&reserved=0). Additionally, PLOS ONE offers an option for publishing peer-reviewed Lab Protocol articles, which describe protocols hosted on protocols.io. Read more information on sharing protocols at [https://plos.org/protocols?utm_medium=editorial-email&utm_source=authorletters&utm_campaign=protocols](https://gcc02.safelinks.protection.outlook.com/?url=https%3A%2F%2Fplos.org%2Fprotocols%3Futm_medium%3Deditorial-email%26utm_source%3Dauthorletters%26utm_campaign%3Dprotocols&data=05%7C01%7C%7C52c97a66b9d34c93092808db199dc27f%7Ced5b36e701ee4ebc867ee03cfa0d4697%7C0%7C0%7C638131938149720049%7CUnknown%7CTWFpbGZsb3d8eyJWIjoiMC4wLjAwMDAiLCJQIjoiV2luMzIiLCJBTiI6Ik1haWwiLCJXVCI6Mn0%3D%7C3000%7C%7C%7C&sdata=aZAHCIw9%2BaQauApdpuJ1aJ2jxtrdMT2FN7EK75TQ%2FRE%3D&reserved=0).

We look forward to receiving your revised manuscript.

Kind regards,

Clara Mendoza-Lera
Academic Editor
PLOS ONE

Journal Requirements:

Please review your reference list to ensure that it is complete and correct. If you have cited papers that have been retracted, please include the rationale for doing so in the manuscript text, or remove these references and replace them with relevant current references. Any changes to the reference list should be mentioned in the rebuttal letter that accompanies your revised manuscript. If you need to cite a retracted article, indicate the article’s retracted status in the References list and also include a citation and full reference for the retraction notice.

[Note: HTML markup is below. Please do not edit.]

Reviewers' comments:

Reviewer's Responses to Questions

**Comments to the Author**

1. If the authors have adequately addressed your comments raised in a previous round of review and you feel that this manuscript is now acceptable for publication, you may indicate that here to bypass the “Comments to the Author” section, enter your conflict of interest statement in the “Confidential to Editor” section, and submit your "Accept" recommendation.

Reviewer #4: All comments have been addressed

2. Is the manuscript technically sound, and do the data support the conclusions?

The manuscript must describe a technically sound piece of scientific research with data that supports the conclusions. Experiments must have been conducted rigorously, with appropriate controls, replication, and sample sizes. The conclusions must be drawn appropriately based on the data presented.

Reviewer #4: Yes

3. Has the statistical analysis been performed appropriately and rigorously?

Reviewer #4: Yes

4. Have the authors made all data underlying the findings in their manuscript fully available?

The [PLOS Data policy](https://gcc02.safelinks.protection.outlook.com/?url=http%3A%2F%2Fwww.plosone.org%2Fstatic%2Fpolicies.action%23sharing&data=05%7C01%7C%7C52c97a66b9d34c93092808db199dc27f%7Ced5b36e701ee4ebc867ee03cfa0d4697%7C0%7C0%7C638131938149720049%7CUnknown%7CTWFpbGZsb3d8eyJWIjoiMC4wLjAwMDAiLCJQIjoiV2luMzIiLCJBTiI6Ik1haWwiLCJXVCI6Mn0%3D%7C3000%7C%7C%7C&sdata=waVbI28aPUptRs48E%2BJPTmunGq7WS2KN6CAeDi75vy8%3D&reserved=0) requires authors to make all data underlying the findings described in their manuscript fully available without restriction, with rare exception (please refer to the Data Availability Statement in the manuscript PDF file). The data should be provided as part of the manuscript or its supporting information, or deposited to a public repository. For example, in addition to summary statistics, the data points behind means, medians and variance measures should be available. If there are restrictions on publicly sharing data—e.g. participant privacy or use of data from a third party—those must be specified.

Reviewer #4: Yes

5. Is the manuscript presented in an intelligible fashion and written in standard English?

PLOS ONE does not copyedit accepted manuscripts, so the language in submitted articles must be clear, correct, and unambiguous. Any typographical or grammatical errors should be corrected at revision, so please note any specific errors here.

Reviewer #4: Yes

6. Review Comments to the Author

Please use the space provided to explain your answers to the questions above. You may also include additional comments for the author, including concerns about dual publication, research ethics, or publication ethics. (Please upload your review as an attachment if it exceeds 20,000 characters)

Reviewer #4: Dear authors.
The manuscript titled “Stream nitrate enrichment and increased light yet no algal response following forest harvest and experimental manipulation of headwater riparian zones” aims to analyze changes in epilithic biofilm, chlorophyll a, nutrients, and sediment deposition in headwater streams under pre and post-harvest comparing with reference sites and analyzing the effects in downstream sites on the watershed. Although the researchers found a significant increase in light and nutrients (in this case DIN), no significant differences were found on the tested epilithic biofilm and chlorophyll a, possibly related to a co-limitation driven by the low phosphorous concentration.
It seems to me that the statistical approaches are quite accurate to try to find the relationships at different levels, either treating the watersheds as treatments or analyzing each one given the great variability that each watershed has. That is why it seems to me that the responses of the authors to the previous reviews make it clear why the use of multiple statistical analyzes is adequate for this type of situation and I agree that the authors mention that the lack of response from the variables mentioned to environmental changes is not always reported in previous studies. This gives great importance to this article being published since it can serve as a starting point for the following researchers that can give a more powerful response to decision-makers in the management and management of hydrobiological resources.
Solo adjunto algunos errores tipográficos en los manuscritos .pfd.

RESPONSE FROM AUTHORS: We appreciate your review of our paper.

7. PLOS authors have the option to publish the peer review history of their article ([what does this mean?](https://gcc02.safelinks.protection.outlook.com/?url=https%3A%2F%2Fjournals.plos.org%2Fplosone%2Fs%2Feditorial-and-peer-review-process%23loc-peer-review-history&data=05%7C01%7C%7C52c97a66b9d34c93092808db199dc27f%7Ced5b36e701ee4ebc867ee03cfa0d4697%7C0%7C0%7C638131938149720049%7CUnknown%7CTWFpbGZsb3d8eyJWIjoiMC4wLjAwMDAiLCJQIjoiV2luMzIiLCJBTiI6Ik1haWwiLCJXVCI6Mn0%3D%7C3000%7C%7C%7C&sdata=VDBpUybCH6bizY0oltxYGcAbh%2Fy6U9WY51xDQ%2B00bPY%3D&reserved=0)). If published, this will include your full peer review and any attached files.


If you choose “no”, your identity will remain anonymous but your review may still be made public.


**Do you want your identity to be public for this peer review?** For information about this choice, including consent withdrawal, please see our [Privacy Policy](https://gcc02.safelinks.protection.outlook.com/?url=https%3A%2F%2Fwww.plos.org%2Fprivacy-policy&data=05%7C01%7C%7C52c97a66b9d34c93092808db199dc27f%7Ced5b36e701ee4ebc867ee03cfa0d4697%7C0%7C0%7C638131938149720049%7CUnknown%7CTWFpbGZsb3d8eyJWIjoiMC4wLjAwMDAiLCJQIjoiV2luMzIiLCJBTiI6Ik1haWwiLCJXVCI6Mn0%3D%7C3000%7C%7C%7C&sdata=AefyQ7%2BD4yDg5DK1R%2FIGVH5q%2Bxep7Tswh3deIYzxxPU%3D&reserved=0).

Reviewer #4: No

[NOTE: If reviewer comments were submitted as an attachment file, they will be attached to this email and accessible via the submission site. Please log into your account, locate the manuscript record, and check for the action link "View Attachments". If this link does not appear, there are no attachment files.]

While revising your submission, please upload your figure files to the Preflight Analysis and Conversion Engine (PACE) digital diagnostic tool, [https://pacev2.apexcovantage.com/](https://gcc02.safelinks.protection.outlook.com/?url=https%3A%2F%2Fpacev2.apexcovantage.com%2F&data=05%7C01%7C%7C52c97a66b9d34c93092808db199dc27f%7Ced5b36e701ee4ebc867ee03cfa0d4697%7C0%7C0%7C638131938149720049%7CUnknown%7CTWFpbGZsb3d8eyJWIjoiMC4wLjAwMDAiLCJQIjoiV2luMzIiLCJBTiI6Ik1haWwiLCJXVCI6Mn0%3D%7C3000%7C%7C%7C&sdata=HfygORgnHirBpGU20IEBW%2B34lAT4DnMbQqHZj%2B4G8wg%3D&reserved=0). PACE helps ensure that figures meet PLOS requirements. To use PACE, you must first register as a user. Registration is free. Then, login and navigate to the UPLOAD tab, where you will find detailed instructions on how to use the tool. If you encounter any issues or have any questions when using PACE, please email PLOS at [figures@plos.org](mailto:figures@plos.org). Please note that Supporting Information files do not need this step.

**From Carl Williams, PLOS ONE:**

PONE-D-22-25727R1
Stream nitrate enrichment and increased light yet no algal response following forest harvest and experimental manipulation of headwater riparian zones
PLOS ONE

Dear Dr. Johnson,

Thank you for submitting your work to PLOS ONE.

After the Academic Editor processed the decision on your paper, we received comments from another reviewer who had also been approached for advice on your work.

I have included the reviewer comments below for your review. Please address the comments raised by this reviewer as part of your revisions and include a response to this review as part of your Response to Reviewers document. The Academic Editor is aware of these additional review comments and will evaluate your response to them as part of the continuing review process when you submit your revision.

Please let me know if you have any questions or concerns

Regards,
Carl Williams
PLOS ONE

---------------------

1. Recommendation
Accept

2. Is the manuscript technically sound, and do the data support the conclusions? (Answer options: Yes, No, Partly)
Yes

3. Has the statistical analysis been performed appropriately and rigorously?
Yes

4. Does the manuscript adhere to the PLOS Data Policy?
Yes

5. Is the manuscript presented in an intelligible fashion and written in standard English?
Yes

6. Review Comments to the Author (minimum 200 characters)
The authors chose to change the message about algal productivity to biomass and did not calculate productivity which was more interesting for the research. The response indicates that the authors are unable or do not know how to calculate productivity from biomass over time. However, the manuscript has a lot of proactive changes making the focus on algae biomass and not activity. It would be interesting to include further discuss the factors that may influence the maintenance of algal biomass, as there is no doubt that solar radiation increased in the stream channel. Developing ideas around sheer stress on algae biomass and herbivory can help the discussion. Overall, the manuscript is suitable for publication after they change it following the review.
The manuscript now has clear proactive changes focusing on algae biomass rather than activity. The authors did not calculate productivity but show the interesting maintenance of algae biomass, although there is a clear increase in solar radiation in stream channels after deforestation. Developing ideas around sheer stress and herbivory on algae biomass could help the topic of discussion.

**RESPONSE FROM AUTHORS:** Thank you for your review. We did not feel that our once per year data on standing stocks supported calculations of instream productivity and we focused on biomass and Chlorophyll a. We did measure instream productivity in a subset of sites each year using diel measurements of instream dissolved oxygen and calculations of re-aeration, which are intended for future publication with Dr. Argerich as the lead author. Therefore it does not make sense to us to publish rough estimates of productivity, using methods of calculation that we are not comfortable with.

In this version, we added text in the Discussion on grazing (herbivory) on algae with the recognition that these headwater streams did not have much green algae, but the biofilms were predominantly composed of diatoms. Sheer stress is now mentioned. While is an important factor for algal growth, in these headwater streams with similar channel slopes and relatively similar stream discharge levels at the time of our annual sampling as well as prior to sampling, it seems a bit of a stretch for this study to delve into those dynamics.

7. PLOS authors have the option to publish the peer review history of their article (<https://gcc02.safelinks.protection.outlook.com/?url=https%3A%2F%2Fjournals.plos.org%2Fplosone%2Fs%2Feditorial-and-peer-review-process%23loc-peer-review-history&data=05%7C01%7C%7Cd69ff6e4234b4e0a11cf08db23eb29fc%7Ced5b36e701ee4ebc867ee03cfa0d4697%7C0%7C0%7C638143265154366507%7CUnknown%7CTWFpbGZsb3d8eyJWIjoiMC4wLjAwMDAiLCJQIjoiV2luMzIiLCJBTiI6Ik1haWwiLCJXVCI6Mn0%3D%7C3000%7C%7C%7C&sdata=8uXLiPrzNnWykkkH6np%2FuGPEauVC%2FYjBtWvVZRw0yVA%3D&reserved=0>). If published, this will include your full peer review and any attached files. Do you want your identity to be public for this peer review? (Answer options: Yes, No)
No

__________________________________________________
In compliance with data protection regulations, you may request that we remove your personal registration details at any time.  (Use the following URL: <https://gcc02.safelinks.protection.outlook.com/?url=https%3A%2F%2Fwww.editorialmanager.com%2Fpone%2Flogin.asp%3Fa%3Dr&data=05%7C01%7C%7Cd69ff6e4234b4e0a11cf08db23eb29fc%7Ced5b36e701ee4ebc867ee03cfa0d4697%7C0%7C0%7C638143265154366507%7CUnknown%7CTWFpbGZsb3d8eyJWIjoiMC4wLjAwMDAiLCJQIjoiV2luMzIiLCJBTiI6Ik1haWwiLCJXVCI6Mn0%3D%7C3000%7C%7C%7C&sdata=HnsbNSIZshJH%2F3lSbeTl2Dmr%2Bk5efWTxnstjw7cbG%2FY%3D&reserved=0)>. Please contact the publication office if you have any questions.
